# Supplementary material for: Connecting omics signatures and revealing biological mechanisms with iLINCS
Source: Nat Commun. 2022 Aug 9;13:4678. doi: 10.1038/s41467-022-32205-3 (PMC9362980; doi:10.1038/s41467-022-32205-3)
Supplement: Supplementary file 1 — Supplementary Information [file 41467_2022_32205_MOESM1_ESM.pdf]

Connecting omics signatures and revealing biological mechanisms with iLINCS

Marcin Pilarczyk[1,2§], Mehdi Fazel-Najafabadi[1,2§], Michal Kouril[2,3§], Behrouz Shamsaei[1,2§], Juozas Vasiliauskas[1,2], Wen Niu[1,2], Naim Mahi[1,2], Lixia Zhang[1,2], Nicholas A Clark[1,2], Yan Ren[1,2], Shana White[1,2], Rashid Karim[1,4], Huan Xu[1,2], Jacek Biesiada[1], Mark F. Bennett[1,2], Sarah E Davidson[1], John F Reichard[1,2], Kurt Roberts[1], Vasileios Stathias[2,5], Amar Koleti[2,5], Dusica Vidovic[2,5], Daniel J.B. Clarke[2,6], Stephan C. Schurer[2,5], Avi Ma'ayan[2,6], Jarek Meller[1,2,4], Mario Medvedovic[1,2,*]

Supplemental Results

Contents

Supplemental Figure 1: Interactive visualization tools in iLINCS. 2

Supplemental Figure 2: iLINCS architecture. 3

Supplemental Results 1: Comparison of iLINCS and clue.io query results 3

Table S1: Top 20 CPs returned by the clue.io 4

Table S2: Top 20 CPs returned by the iLINCS 4

Supplemental Results 2: Benchmarking methods for connectivity analysis and comparisons to other web resources 4

Table S3: Area under the ROC curve (AUC) for six competing connectivity analysis methods across 10 pairs of cancer cell lines. 5

Table S4: Comparing the scope and functionality of iLINCS with other resources. 5

Supplemental Results 3: Utility and scope of applications of the iLINCS data and analytics 6

Supplemental Results 4: MOA analysis of the whole-genome sirolimus signatures. 7

Supplemental Results 5: Analysis of LuminalA and Her2E vs normal tissue proteomic signatures. 7

Supplemental Figure 1: Interactive visualization tools in iLINCS. A) Interactive heatmaps: As a gold standard graphical display for visualizing high-dimensional data and relationships, interactive heatmaps are used throughout iLINCS via several different applications: Native Shiny heatmap, Java script based Morpheus and Clustergrammer. Diversity of heatmap apps provide for diversity of functionalities and it facilitates use of iLINCS in different configurations of network speed vs the computer speed. B) Interactive volcano plots are used for visualizing and selecting informative (eg differentially expressed) genes/proteins in a signature (unadjusted two-tailed p-values displayed); C) Interactive scatter plots are used to visualize relationships between two signatures and identifying genes/proteins driving the "connectivity"; D) Interactive box plots are used to visualize differential distribution of gene/proteins in different samples and up- and down-regulated genes/proteins in different signatures. In this example the distribution of expression levels of the ESR1 genes in 58 HER2-enriched and 231 Luminal A samples derived from independent patients is depicted; E) Interactive GSEA plots serve to visualize strength of connectivity and identify genes/proteins driving the connectivity between a gene list and a signature; F) Interactive 3D scatter plots are used to visualize high-dimensional relationship in dimensionality reduction analysis (PCA and t-SNE); G) Connected 2D scatter plots for visualizing high-dimensional relationship in dimensionality reduction analysis (PCA and t-SNE); H) Interactive pathway visualizations allows for exploring the relationships between signatures and pathways. The nodes are colored according to the differential expression of corresponding genes with blue representing negative and yellow positive values; and K) Interactive network visualizations is used to integrate signatures with the global protein-protein interaction network. The size of the node corresponds to the level of statistical significance (larger nodes are more statistically significant), and color to the level of differential expression on the log scale (green represents negative numbers and red positive numbers).

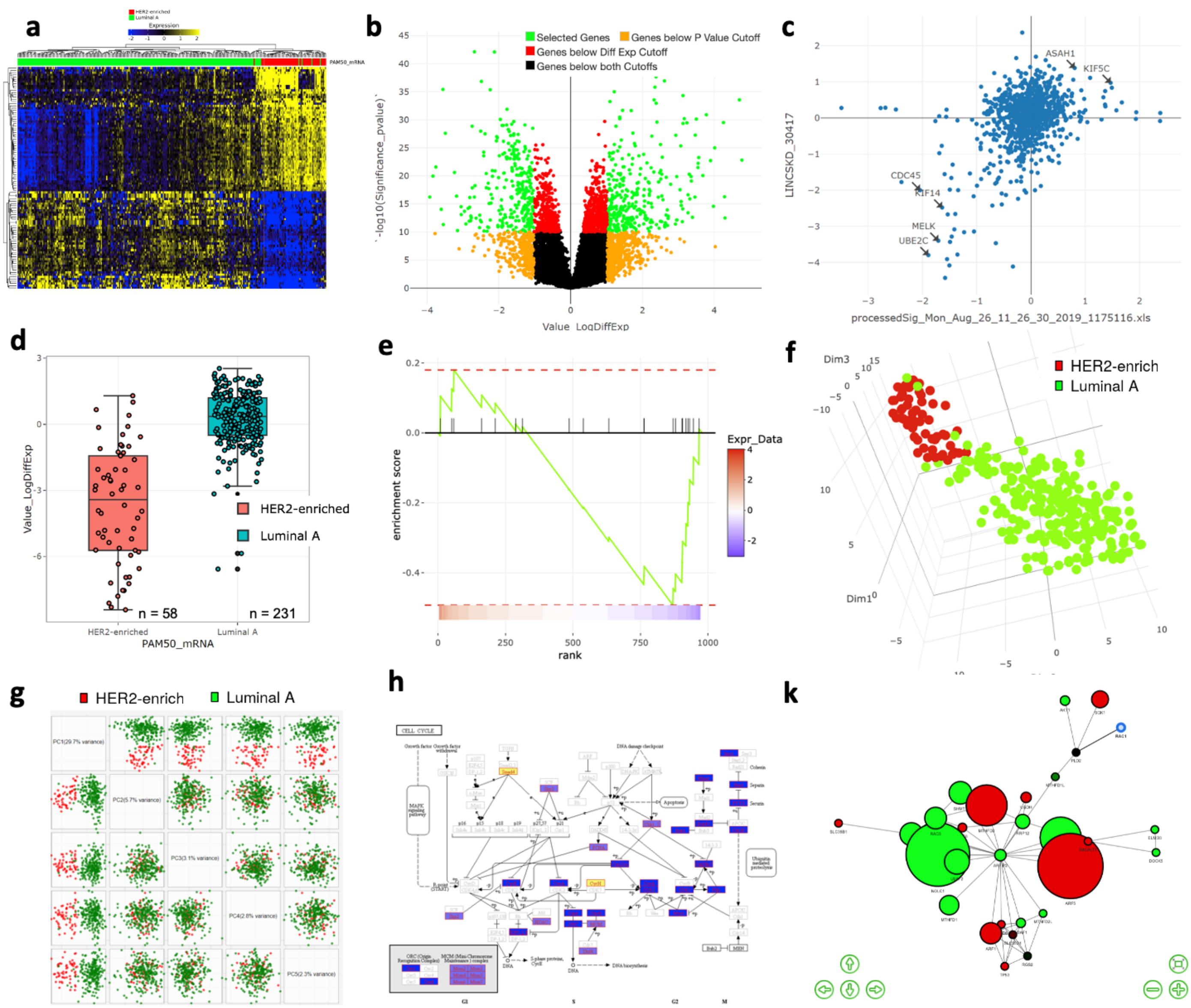

Supplemental Figure 2: iLINCS architecture. iLINCS is based on software stack: MySQL, MongoDB, R, NodeJS, and AngularJS. MySQL and MongoDB backend databases contain pre-processed genomics datasets, signatures and their connections, and all associated metadata. For external users, the database is accessed through a powerful API, which can be explored and tested with the Swagger UI interface. The iLINCS API was created in nodeJS using ExpressJS and Loopback frameworks. The API is documented and can be explored through swagger UI interface, which allows users to explore available API models and methods. The iLINCS internal analytical engine is written in R utilizing a range of specialized R packages. The iLINCS API is based on nodeJS using ExpressJS and Loopback frameworks. The nodeJS API connects with the R analytical engine using the openCPU framework which provides HTTP API for executing R functions. The user interfaces are written in Java Script using AngularJS framework. iLINCS facilitates submission of signatures and intermediate analysis results via redirection APIs to a range of third-party task specific bioinformatics web tools and services.

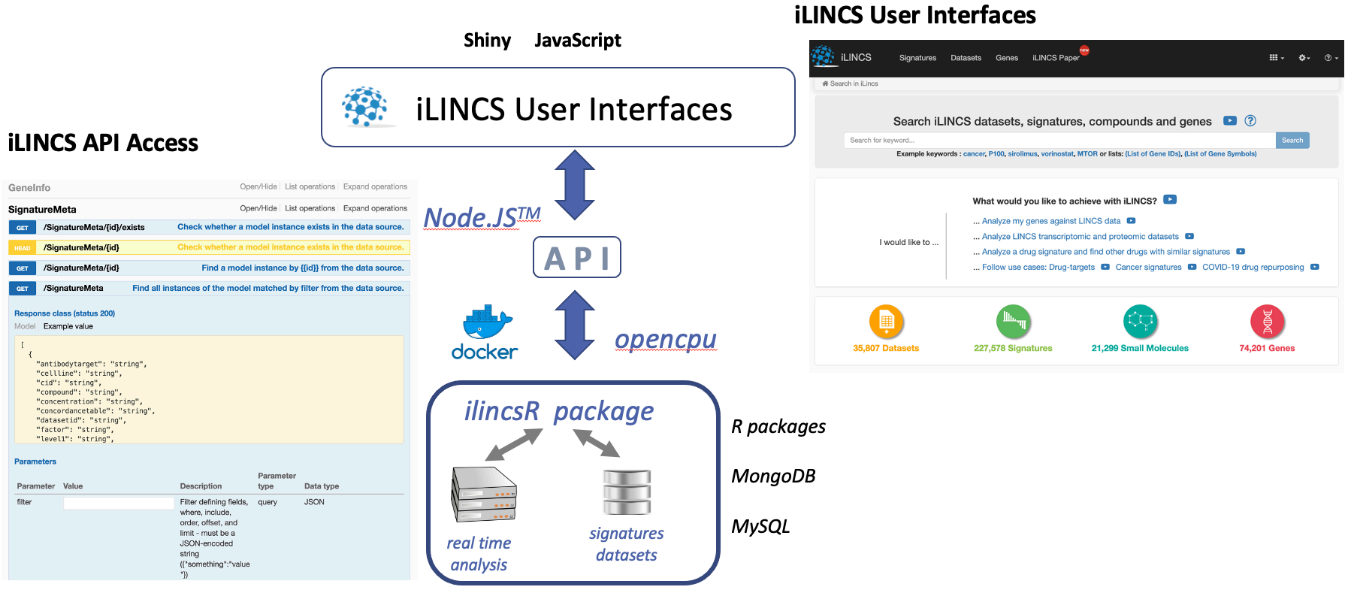

Supplemental Results 1: Comparison of iLINCS and clue.io query results

iLINCS and clue.io query tools were compared based on the "connected" chemical perturbagens (CPs) returned by the query with the MTOR CRISPR consensus gene signature (CGS) used in the Use Case 1 of the main text (Fig 2A). For submission to the clue.io, the signature is summarized as the list of 50 most up-regulated and the 50 most down-regulated genes. The default clue.io connectivity metric, median τ across all cell-lines, was used to identify 20 most connected CPs and results, along with results obtained from iLINCS for the same CPs are shown in Table S1. The clu.io query was executed on September 12, 2019. Most of the connected CPs are also deemed significant by iLINCS analysis. Interestingly, none of the CPs implicated by clue.io, but not picked up as connected by iLINCS target the core elements of the mTOR signaling. On the other hand, all top 20 signatures (lowest pValue) implicated by iLINCS (Table S2) are known to target core elements of mTOR signaling (mTOR, PI3K and AKT proteins). This includes four perturbagens not deemed connected by clue.io (highlighted). Two CPs that show in the iLINCS results, but were missed by clue.io are not in the Touchstone set used by clue.io [7] (is_touchstone=0). Finally, 6 CPs are missing connectivity information in clue.io because they were part of the Phase II LINCS L1000 dataset (GSE70138), which are included in iLINCS, but not used by clue.io. The CPs in red were missing MOA information in the released data, and we established their MOA by manual literature and online databases searches.

In summary, clue.io and iLINCS provide qualitatively similar results with connected CPs targeting mTOR pathway. Some differences were introduced by the different statistical measure of similarity used by the two system, but the biggest

differences most likely came from the scope of the query, where clue.io searches over a space of 2,837 Touchstone CPs and iLINCS searches over a space of 15,349 CPs with at least one high quality signature.

Table S1: Top 20 CPs returned by the clue.io (unadjusted two-tailed p-values based on weighted correlation t-statistics displayed in the table)

Perturbagen ID	Perturbagen Name	ilincsMoa	is_touchstone	median_tau	iLINCS	pValue
BRD-K67566344	KU-0063794	MTOR inhibitor	1	97.35	Connected	3.83E-89
BRD-K84937637	sirolimus	MTOR inhibitor	1	97.31	Connected	7.21E-55
BRD-A84045418	calpeptin	Calpain inhibitor	1	97.23	Connected	2.08E-33
BRD-K12184916	NVP-BEZ235	MTOR inhibitor	1	96.02	Connected	6.94E-161
BRD-K71726959	BRD-K71726959	CDK inhibitor	1	95.33	Not Connected	
BRD-K27305650	LY-294002	MTOR inhibitor	1	95.32	Connected	3.35E-45
BRD-K92577649	GBR-13069	Dopamine uptake inhibitor	1	95.14	Not Connected	
BRD-K30677119	PP-30	RAF inhibitor	1	94.71	Connected	3.48E-18
BRD-K99818283	PIK-90	PI3K inhibitor	1	94.42	Connected	1.27E-54
BRD-K69932463	AZD-8055	MTOR inhibitor	1	93.54	Connected	1.17E-218
BRD-A62025033	temsirolimus	MTOR inhibitor	1	93.49	Connected	3.46E-125
BRD-K77008974	WYE-354	MTOR inhibitor	1	92.05	Connected	4.03E-19
BRD-K06593056	LE-135	Retinoid receptor agonist	1	91.85	Not Connected	
BRD-K21350491	phenamil	TRPV antagonist	1	91.54	Not Connected	
BRD-K64835161	BRD-K64835161	CLK inhibitor,DYRK inhibitor	1	91.53	Connected	1.18E-04
BRD-A77299732	salubrinal	Eukaryotic translation initiation facto	1	91.47	Not Connected	
BRD-K13800121	parecoxib	Cyclooxygenase inhibitor	1	90.63	Connected	5.08E-06
BRD-K67868012	PI-103	PI3K inhibitor,MTOR inhibitor	1	90.59	Connected	7.37E-144
BRD-K71879491	tretinoin	Retinoid receptor agonist	1	90.33	Not Connected	
BRD-A75409952	wortmannin	PI3K inhibitor	1	90.30	Connected	2.14E-110

Table S2: Top 20 CPs returned by the iLINCS (unadjusted two-tailed p-values based on weighted correlation t-test displayed in the table)

Perturbagen ID	Perturbagen Name	ilincsMoa	is_touchstone	median_tau	iLINCS	pValue
BRD-K02708799	GSK 1059615	PI3K inhibitor	0		Connected	0
BRD-A79768653	sirolimus	MTOR inhibitor	1	83.07	Connected	1.6E-293
BRD-K69932463	AZD-8055	MTOR inhibitor	1	93.54	Connected	1.2E-218
BRD-K12184916	NVP-BEZ235	MTOR inhibitor	1	96.02	Connected	6.9E-161
BRD-K59317601	MLN-0128	MTOR inhibitor			Connected	4.6E-144
BRD-K67868012	PI-103	PI3K inhibitor,MTOR inhibitor	1	90.59	Connected	7.4E-144
BRD-K94012289	936890-98-1	MTOR inhibitor	1		Connected	9.4E-137
BRD-K40175214	torin-1	MTOR inhibitor,PI3K inhibitor	1	71.90	Connected	1.9E-135
BRD-A45498368	WYE-125132	MTOR inhibitor	1	79.43	Connected	4.3E-127
BRD-A62025033	temsirolimus	MTOR inhibitor	1	93.49	Connected	3.5E-125
BRD-K52911425	GDC-0941	PI3K inhibitor	1	82.75	Connected	5.2E-125
BRD-A75409952	wortmannin	PI3K inhibitor	1	90.30	Connected	2.1E-110
BRD-K13154216	Everolimus	MTOR inhibitor			Connected	4.4E-109
BRD-K42898655	Temsirolimus	MTOR inhibitor			Connected	8E-104
BRD-K99023089	AZD5363	AKT inhibitor			Connected	3.2E-100
BRD-K09078998	Ridaforolimus	MTOR inhibitor			Connected	8.83E-97
BRD-K63068307	ZSTK-474	PI3K inhibitor	1	78.95	Connected	7.61E-94
BRD-K67566344	KU-0063794	MTOR inhibitor	1	97.35	Connected	3.83E-89
BRD-K72636697	SCHEMBL17052537	MTOR inhibitor	0		Connected	2.91E-86
BRD-A25736793	Everolimus	MTOR inhibitor			Connected	4.02E-79

Supplemental Results 2: Benchmarking methods for connectivity analysis and comparisons to other web resources

We performed a limited benchmarking study of several methods for "connecting" transcriptional signatures with the objective to identify optimal methods for pre-computed connections between signatures in the iLINCS libraries, and for

connectivity analysis of newly created and submitted signatures. The choice of the methods were motivated by previous studies[7,9-11] and included Extreme and Weighted correlations, the Connectivity Score used by the Connectivity Map, and the correlation based on signed log significance, which was motivated by our previous results in the context of enrichment analysis[12]. The methods were benchmarked with respect to their ability to "connect" a chemical perturbagen signature in one cancer cell line with the equivalent signature in another cell line for five cell lines with most signatures in the LINCS L1000 dataset (A375, A549, PC3, VCAP and MCF7). Only signatures designated as "gold" and generated using the "epsilon" version of L1000 probes (GEO CMap LINCS User Guide v2.1) were used in the analysis. Two signatures that share the same treatment (same small molecule, same dose and duration) in two different cell lines were considered to be true positive signature pair. True negative signature pairs were defined as pairs of signatures from different cell lines treated with different chemical perturbagen. For each 10 different cell line combinations, the number of "true positive pairs" ranged between 5,793 and 16,477 (the average number is 10,145). To calculate ROC curves, 50,000 "true negative pairs" randomly selected from all true negative pairs for a given cell line combination. Following six methods were compared:

WeightedCor: Weighted Pearson's correlation between MODZ scores, weighted by the logarithm of the product of the p-values. This score was shown to be best in correlating genome wide signatures of related biological states [11].

Cor: Pearsons's correlation between MODZ scores of the two signatures.

ExtremeCorLobP_100: Extreme Correlation of Signed Log P-values utilizing top 100 up- and down-regulated genes. The signed significance of the ith gene is defined as

$$s_i = \text{sign}(d_i) * (-\log_{10}(p_i)), \text{ for } i = 1, \dots, N,$$

and the signed significance signature is $\boldsymbol{s}=(s_1,\dots,s_N)$. The extreme signed signature $\boldsymbol{e}=(e_1,\dots,e_N)$ is then constructing by setting the signed significances of all genes other than the top 100 and bottom 100 to zero:

$$e_i = \begin{Bmatrix} s_i, if\ s_i \geq s^{100} \text{ or } s_i \leq s^{-100} \\ 0, \text{otherwise} \end{Bmatrix}$$

Where s^{100} is the 100th most positive s_i and s^{-100} is the 100th most negative s_i. The extreme Pearson correlation between two signatures is then calculated as the standard Pearson's correlation between the extreme signed significance signatures.

ExtremeWeightedCor_100: Extreme Weighted Pearson's Correlations calculating weighted Pearson's correlation (see **WeightedCor**) after setting MODZ's of all but top 100 up- and down-regulated genes to zero (see **ExtremeCorLobP_100)**

CMap_100: Connectivity score as described in the Connectivity Map publication [7] using the top 100 up- and down-regulated genes as the query.

Overall, all six methods performed very well on this task with average AUC's ranging from 0.946 to 0.926 (Table S3). The decision was made to use the Extreme Correlation of Signed Log P-values utilizing top 100 up- and down-regulated genes as the method for pre-computed connections, since it showed the best performance. For real-time connectivity analysis of submitted and newly created signatures, we decided to use Weighted Correlations since they were almost as good as the best method, but much easier to compute in the real time. To facilitate the fast computations of weighted correlations, the algorithm was implemented in C and added to the backend R package using the *Rcpp* package [13].

Table S3: Area under the ROC curve (AUC) for six competing connectivity analysis methods across 10 pairs of cancer cell lines.

Method	A375xA549	A375xMCF7	A375xPC3	A375xVCAP	A549xMCF7	A549xPC3	A549xVCAP	MCF7xPC3	MCF7xVCAP	PC3xVCAP	Average
ExtremeCorLogP_100	0.951	0.952	0.957	0.930	0.947	0.947	0.926	0.957	0.947	0.948	0.946
ExtremeWeightedCor_100	0.946	0.947	0.951	0.925	0.944	0.944	0.921	0.953	0.944	0.946	0.942
WeightedCor	0.945	0.947	0.952	0.924	0.943	0.943	0.918	0.954	0.941	0.943	0.941
ExtremeCor_100	0.942	0.946	0.947	0.921	0.941	0.941	0.914	0.952	0.936	0.939	0.938
Cor	0.938	0.943	0.945	0.914	0.936	0.937	0.903	0.949	0.925	0.929	0.932
CMap_100	0.935	0.940	0.941	0.906	0.931	0.933	0.895	0.945	0.915	0.921	0.926

Table S4: Comparing the scope and functionality of iLINCS with other resources. There are numerous online analysis tools that cover different aspects of cancerLINCS functionality: access to a vast amount of data[2,5,6,14,15], "connectivity

analysis" with LINCS signatures[5,7], user friendly web interfaces for analysis of primary transcriptomic data[16], and more targeted collections of cancers omics datasets[1,3,4], various aspects of systems biology analysis of omics signatures[17-23], and interactive visualizations[24,25]. However, *iLINCS* platform is unique in bringing together all different aspects of a comprehensive signature analysis platform.

	GUI based analysis	*API*	*Signature Creation*	*Systems Biology Analysis*	*Connectivity Analysis*	*Multi-omic Analysis*	*Bulk Transcriptomic Datasets*	*Single Cell RNA-seq Datasets*	*Proteomic Datasets*
iLINCS	**Yes**	**Yes**	**Yes**	**Yes**	**Yes**	**Yes**	**Yes**	**Limited**	**Yes**
cBioPortal[1]	**Yes**	**Yes**	**No**	**No**	**No**	**No**	**Yes**	**No**	**No**
GDC[2]	**Yes**	**Yes**	**No**	**No**	**No**	**No**	**Yes**	**No**	**No**
Expression Atlas[3]	**Limited**	**Limited**	**Limited**	**No**	**No**	**No**	**Yes**	**Limited**	**No**
CancerSEA[4]	**Yes**	**No**	**No**	**No**	**No**	**No**	**No**	**Limited**	**No**
BioJupies[5]	**No**	**No**	**Yes**	**Yes**	**Yes**	**No**	**Yes**	**No**	**No**
GEO[6]	**Yes**	**Limited**	**Limited**	**No**	**No**	**No**	**Yes**	**Limited**	**Limited**
clue.io[7]	**Yes**	**Limited**	**No**	**No**	**Yes**	**No**	**No**	**No**	**No**
WebMeV[8]	**Yes**	**No**	**Yes**	**Yes**	**No**	**No**	**Yes**	**No**	**No**

Supplemental Results 3: Utility and scope of applications of the iLINCS data and analytics

The utility of re-using omics datasets in public domain is self-evident. Thousands of high impact papers have demonstrated that such analyses can yield important insight. The conceptual utility of the CMAP analyses at this point is also evidenced by the referenced usage of CMAP data in the peer reviewed publication. The original Science 2006, CMAP paper has been referenced more than 4,000 times and the most recent Cell 2017 paper describing L1000 dataset has been referenced more than 1,000 times. We collected and analyzed the disease-related topics of the studies referencing the CMAP papers using the Dimensions platform (https://www.dimensions.ai/). The results indicate a broad utility of the CMAP approach in terms the of diseases studied with cancers being the most studied, but still representing less only about a third of all use cases (CMAP references in Supplemental Figure 3). We also summarized in the same way topics of 114 peer reviewed

Supplemental Figure 3. Top 8 disease categories associated with publications referencing the two primary CMAP papers (CMAP references) and publications referencing iLINCS.

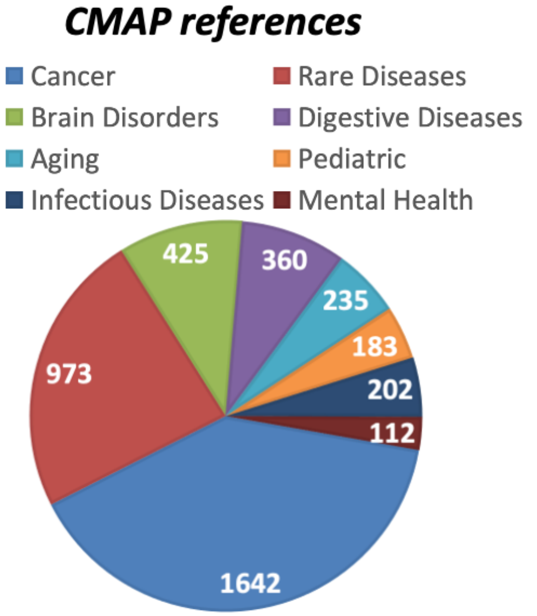

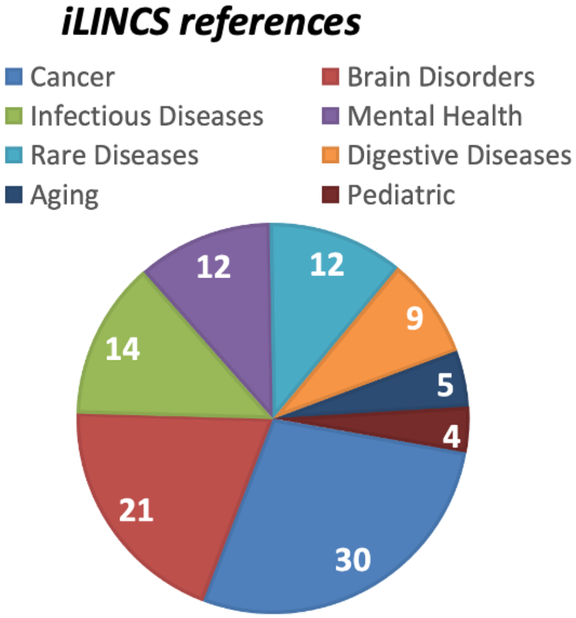

publications referencing the use of iLICNS (iLINCS references in Supplemental Figure 3). The distribution of disease use cases closely resembled the general CMAP distribution, with cancers representing even smaller fraction of all use cases.

Supplemental Results 4: MOA analysis of the whole-genome sirolimus signatures.

To verify that the reduced representation of global transcriptome offered by the L1000 "landmark genes", we repeated the analysis in Use Case 1 with the sirolimus signature generated on the whole-genome microarray in the original CMAP dataset [26] (Supplemental Figure 4A). The enrichment analysis of 191 highly statistically significant genes (p-value<5e-10) failed was not enriched for KEGG mTOR pathway genes (Enrichr p-value=0.43). We correlated the sirolimus signature with LINCS CGSes (Supplemental Figure 4B) and established that mTOR signaling pathway genes dominated the most positively correlated CGSes(Supplemental Figure 4C). The pathway enrichment analysis of genes perturbed in the top 100 most positively correlated CGSes showed statistically significant enrichment of mTOR pathway genes (Enrichr p-value=5.4e-9). All Enrichr results are given in Supplemental Table ST4.

Supplemental Figure 4. Analysis of whole-genome sirolimus signature. A) The iLINCS whole genome signature generated by the original CMAP project; B) 192 differentially expressed (unadjusted two-tailed p-value<10^{-10}) genes used for enrichment analysis; C) Top 5 most perturbed genes in top 100 most positively correlated CGSes.

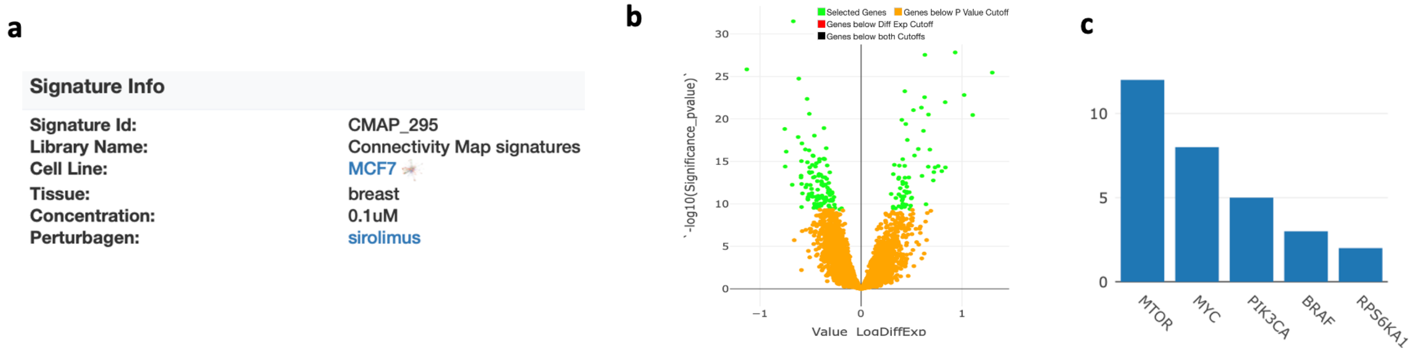

Supplemental Results 5: Analysis of LuminalA and Her2E vs normal tissue proteomic signatures (p-values displayed in the table are unadjusted, two-tailed, empirical Bayes t-test p-values)

Here we contrast the effectiveness of the LuminalA and Her2E signatures described in Use Case 2, with the corresponding signatures constructed by comparing LuminalA and Her2E samples with normal breast tissue. Table S5 shows the top 12 most significantly differentially expressed probes (unadjusted, two-tailed, empirical Bayes t-test p-value<1e-10) in the direct comparison. As noted in the main text, the direct comparison elicits the known drivers of both LuminalA (ER-alpha) and Her2E tumors (HER2), and therapeutic targets currently used in clinic. In the comparison of tumor samples to normal

Table S5. Proteomic signatures constructed by directly comparing LuminalA and Her2E tumors, and by comparing LuminalA and Her2E samples to Normal breast tissue.

LuminalA vs Her2E				LuminalA vs Normal				Her2E vs Normal			
Probe	Gene	Log2 Diff Exp	p-value	Probe	Gene	Log2 Diff Exp	p-value	Probe	Gene	Log2 Diff Exp	p-value
ER-alpha	ESR1	2.8	1.3E-25	Chk1	CHEK1	-0.9	1.2E-36	Chk1	CHEK1	-0.8	7.9E-15
HER2_pY1248	ERBB2	-1.3	3.6E-23	MSH2	MSH2	-1.1	3.8E-36	Caveolin-1	CAV1	-1.5	2.0E-14
HER2	ERBB2	-1.6	2.9E-22	S6_pS240_S244	RPS6	-1.3	3.4E-26	MSH2	MSH2	-0.9	1.1E-13
Bcl-2	BCL2	1.3	2.5E-20	Src_pY416	SRC,LYN,FYN,L	-1.0	1.9E-24	GSK3-alpha-b	GSK3A,GSK3B	0.6	6.3E-12
PR	PGR	2.9	8.2E-20	JAB1	COPS5	-0.6	5.2E-23	DIRAS3	DIRAS3	-0.6	9.8E-12
EGFR_pY1068	EGFR	-1.2	2.1E-19	PI3K-p85	PIK3R1	0.7	1.5E-21	c-Met	MET	-0.4	1.3E-11
ASNS	ASNS	-0.6	1.0E-16	SF2	SRSF1	-0.5	2.0E-20	HER3_pY1289	ERBB3	0.7	2.0E-11
FoxM1	FOXM1	-0.5	8.6E-16	Beclin	BECN1	-0.7	2.4E-18	Chk2_pT68	CHEK2	0.7	2.1E-11
Cyclin_B1	CCNB1	-1.0	5.8E-15	GAPDH	GAPDH	-1.1	2.8E-18	Syk	SYK	1.0	2.8E-11
GATA3	GATA3	1.0	4.8E-12	Bax	BAX	0.6	5.2E-18	JAB1	COPS5	-0.7	3.3E-11
G6PD	G6PD	-0.5	1.3E-11	Bap1-c-4	BAP1	-0.6	7.9E-18	Ku80	XRCC5	0.7	7.4E-11
4E-BP1_pS65	EIF4EBP1	-0.3	9.9E-11	c-Abl	ABL1	0.5	2.2E-17	Bcl2A1	BCL2A1	-0.6	2.4E-10

tissue, these two proteins do not reach statistical significance (unadjusted, two-tailed, empirical Bayes t-test p-value of 1e-10) and are not among the 12 most differentially expressed proteins. Instead, the top differentially expressed proteins seem to be more generic cancer related genes, including three proteins downregulated in both LuminalA and Her2E samples (highlighted).

Reference List

1 Gao, J. *et al.* Integrative analysis of complex cancer genomics and clinical profiles using the cBioPortal. *Sci. Signal.* **6**, pl1-pl1 (2013).

2 Grossman, R. L. *et al.* Toward a Shared Vision for Cancer Genomic Data. *N Engl J Med* **375**, 1109-1112, doi:10.1056/NEJMp1607591 (2016).

3 Papatheodorou, I. *et al.* Expression Atlas update: from tissues to single cells. *Nucleic Acids Res* **48**, D77-D83, doi:10.1093/nar/gkz947 (2020).

4 Yuan, H. *et al.* CancerSEA: a cancer single-cell state atlas. *Nucleic Acids Res* **47**, D900-D908, doi:10.1093/nar/gky939 (2019).

5 Torre, D., Lachmann, A. & Ma'ayan, A. BioJupies: Automated Generation of Interactive Notebooks for RNA-Seq Data Analysis in the Cloud. *Cell Syst* **7**, 556-561 e553, doi:10.1016/j.cels.2018.10.007 (2018).

6 Barrett, T. *et al.* NCBI GEO: archive for high-throughput functional genomic data. *Nucleic Acids Res* **37**, D885-890, doi:10.1093/nar/gkn764 (2009).

7 Subramanian, A. *et al.* A Next Generation Connectivity Map: L1000 Platform and the First 1,000,000 Profiles. *Cell* **171**, 1437-1452 e1417, doi:10.1016/j.cell.2017.10.049 (2017).

8 Wang, Y. E., Kutnetsov, L., Partensky, A., Farid, J. & Quackenbush, J. WebMeV: A Cloud Platform for Analyzing and Visualizing Cancer Genomic Data. *Cancer Res* **77**, e11-e14, doi:10.1158/0008-5472.CAN-17-0802 (2017).

9 Iwata, M., Sawada, R., Iwata, H., Kotera, M. & Yamanishi, Y. Elucidating the modes of action for bioactive compounds in a cell-specific manner by large-scale chemically-induced transcriptomics. *Scientific Reports* **7**, 40164, doi:10.1038/srep40164

https://www.nature.com/articles/srep40164#supplementary-information (2017).

10 Cheng, J. *et al.* in *Pac. Symp. Biocomput.* 5-16 (World Scientific).

11 Engreitz, J. *et al.* Content-based microarray search using differential expression profiles. *BMC Bioinformatics* **11**, 603 (2010).

12 Sartor, M. A., Leikauf, G. D. & Medvedovic, M. LRpath: a logistic regression approach for identifying enriched biological groups in gene expression data. *Bioinformatics* **25**, 211-217, doi:10.1093/bioinformatics/btn592 (2009).

13 Eddelbuettel, D. *et al.* Rcpp: Seamless R and C++ integration. *Journal of Statistical Software* **40**, 1-18 (2011).

14 Athar, A. *et al.* ArrayExpress update–from bulk to single-cell expression data. *Nucleic acids research* **47**, D711-D715 (2019).

15 Lachmann, A. *et al.* Massive mining of publicly available RNA-seq data from human and mouse. *Nat Commun* **9**, 1366, doi:10.1038/s41467-018-03751-6 (2018).

16 Al Mahi, N., Najafabadi, M. F., Pilarczyk, M., Kouril, M. & Medvedovic, M. GREIN: An interactive web platform for re-analyzing GEO RNA-seq data. *Scientific reports* **9**, 7580 (2019).

17 Kuleshov, M. V. *et al.* Enrichr: a comprehensive gene set enrichment analysis web server 2016 update. *Nucleic Acids Res* **44**, W90-97, doi:10.1093/nar/gkw377 (2016).

18 Dennis, G., Jr. *et al.* DAVID: Database for Annotation, Visualization, and Integrated Discovery. *Genome Biol* **4**, P3 (2003).

19 Chen, J., Bardes, E. E., Aronow, B. J. & Jegga, A. G. ToppGene Suite for gene list enrichment analysis and candidate gene prioritization. *Nucleic Acids Res* **37**, W305-311, doi:10.1093/nar/gkp427 (2009).

20 Fabregat, A. *et al.* The Reactome pathway Knowledgebase. *Nucleic Acids Res* **44**, D481-487, doi:10.1093/nar/gkv1351 (2016).

21 Kanehisa, M., Furumichi, M., Tanabe, M., Sato, Y. & Morishima, K. KEGG: new perspectives on genomes, pathways, diseases and drugs. *Nucleic Acids Res* **45**, D353-D361, doi:10.1093/nar/gkw1092 (2017).

22 Warde-Farley, D. *et al.* The GeneMANIA prediction server: biological network integration for gene prioritization and predicting gene function. *Nucleic Acids Res* **38**, W214-220, doi:10.1093/nar/gkq537 (2010).

23 Clarke, D. J. B. *et al.* eXpression2Kinases (X2K) Web: linking expression signatures to upstream cell signaling networks. *Nucleic Acids Res* **46**, W171-W179, doi:10.1093/nar/gky458 (2018).

24 Freudenberg, J. M., Joshi, V. K., Hu, Z. & Medvedovic, M. CLEAN: CLustering Enrichment ANalysis. *BMC Bioinformatics* **10**, 234 (2009).

25 *Morpheus*, <https://software.broadinstitute.org/morpheus> (

26 Lamb, J. *et al.* The Connectivity Map: using gene-expression signatures to connect small molecules, genes, and disease. *Science* **313**, 1929-1935 (2006).

Supplemental Methods

Table of Contents

Perturbation signatures creation and analysis 11

Perturbation signatures 11

Constructing signatures with iLINCS 11

Signature connectivity analysis....11

Perturbagen connectivity analysis 14

iLINCS signature libraries 15

LINCS L1000 signature libraries 15

LINCS targeted proteomics signatures 16

Disease related signatures....16

ENCODE transcription factor binding signatures....16

Connectivity Map Signatures....17

DrugMatrix signatures 17

Transcriptional signatures from EBI Expression Atlas....17

Cancer therapeutics response signatures....17

Pharmacogenomics transcriptional signatures 17

Analytical tools, web applications and web resources 17

Gene and protein expression dataset collections 18

Perturbation signatures creation and analysis

Perturbation signatures

All pre-computed perturbation signatures in iLINCS, as well as signatures created using an iLINCS dataset, consist of a pair of vectors (**d**,**p**): the vector of differential expressions between the perturbed/treated samples and baseline samples **d**=(d_1,…,d_N), and the vector of associated p-values **p**=(p_1,…,p_N), where N is the number of genes or proteins in the signature. Query signatures submitted by the user can also consist of only log-scale differential expressions without p-values (***d***), lists of up- and down-regulated genes ($\mathbf{gl}_{\mathbf{up}}$,$\mathbf{gl}_{\mathbf{down}}$), and a single list of genes (**gl**). Identities of the genes in iLINCS are established with *Entrez* Gene identifiers[1] and associated gene symbols.

Constructing signatures with iLINCS

A signature in iLINCS is constructed by specifying the "treatment" and "baseline" sets of samples. Depending on the data type, iLINCS uses an appropriate statistical model to calculate $\log_2$-scale differential expressions (***d***), and the associated p-values (***p***).

For microarray gene expression datasets, TCGA RNA-seq data pre-processed using RSEM methodology[2], TCGA copy number variation (CNV) datasets pre-processed using the GISTIC 2.0 methodology [3], and RPPA protein expression datasets [4], iLINCS uses the empirical Bayes linear model for two sample group comparison (ie, empirical Bayes t-test) as implemented in the *limma* package[5]. Complete workflow for constructing a signatures and connectivity analysis on the constructed signature using iLINCS user interfaces (UI) is depicted in the Use Case 3 Supplemental Workflow. The same analyses can be performed using iLINCS API and the examples are provided *usingIlincsApis* RStudio notebook freely accessible from our github repository (https://github.com/uc-bd2k/ilincsAPI).

For GEO RNA-seq datasets pre-processed by the GEO RNA-seq Experiments Interactive Navigator (*GREIN)*[6] platform, iLINCS constructs the signature (**d**,**p**) by differential gene expression analysis of "treatment" and "baseline" samples using the negative binomial statistical model as implemented in the *edgeR* [7] via the connection with *GREIN*.

Signature connectivity analysis

Depending on the exact type of the query signature, the connectivity analysis with libraries of pre-computed iLINCS signatures are computed using different connectivity metrics. The choice of the similarity metric to be used in different contexts was driven by benchmarking six different methods (Supplementary Result 2) and computational considerations.

*Query with a pre-computed (***d**,**p***) iLINCS signature*

If the query signature (**d**,**p**) is selected from iLINCS libraries of pre-computed signatures, the connectivity with all other iLINCS signatures is also pre-computed using the extreme Pearson's correlation[8,9] of signed significances of all genes. The signed significance of the ith gene is defined as

$$ss_i = \text{sign}(d_i) * (-\log_{10}(p_i)),\ \ for\ \ i = 1, \dots, N, \qquad (1)$$

and the signed significance signature is **ss**=(ss_1,…,ss_N). The extreme signed signature **e**=(e_1,…,e_N) is then constructing by setting the signed significances of all genes other than the top 100 and bottom 100 to zero:

$$e_i = \begin{Bmatrix} ss_i, if\ ss_i \geq ss^{100}\ or\ ss_i \leq ss^{-100} \\ 0, \text{otherwise} \end{Bmatrix} \qquad (2)$$

Where ss^{100} is the 100th most positive ss_i and ss^{-100} is the 100th most negative ss_i. The extreme Pearson correlation between two signatures, ($\boldsymbol{d}_1$,$\boldsymbol{p}_1$) and ($\boldsymbol{d}_2$,$\boldsymbol{p}_2$), is then calculated as the standard Pearson's correlation between corresponding extreme signed signature $\boldsymbol{e}_1$ and $\boldsymbol{e}_2$:

$$r(\mathbf{e}_1, \mathbf{e}_2) = \frac{\sum_{i=1}^{N}[(e_{1i}-\bar{e}_1)(e_{2i}-\bar{e}_2)]}{\sqrt{\sum_{i=1}^{N}[(e_{1i}-\bar{e}_1)^2]\sum_{i=1}^{n}[(e_{2i}-\bar{e}_2)^2]}} \qquad (3)$$

where

$$\bar{e_1} = \sum_{i=1}^{N} \frac{e_{1i}}{N} \; and \; \bar{e_2} = \sum_{i=1}^{N} \frac{e_{2i}}{N} \qquad (4)$$

The statistical significance of r is calculated as:

$$\text{p} - \text{value}(r) = \text{Probability}\left(|t_{N-2}| > r\sqrt{\frac{N-2}{1-r^2}} \right) \qquad (5)$$

Where t_{N-2} the random variable distributed as t-distribution with N-2 degrees of freedom. The association between the query signature and another iLINCS signature is visualized in iLINCS by an interactive scatter plot of log differential expression levels created by *ggplot2 and plotly* packages as illustrated in Fig 1. Complete workflow for connectivity analysis of pre-computed iLINCS signatures using iLINCS GUI is depicted in the Use Case 1 Supplemental Workflow and the examples for performing the same analyses with iLINCS API are provided in the *usingIlincsApis* RStudio notebook.

Fig 1

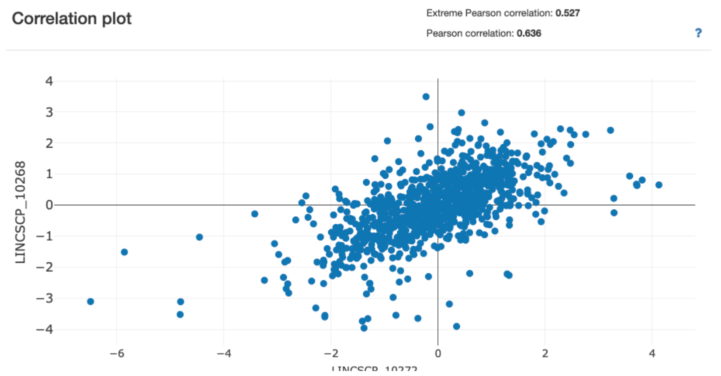

*Query with a user submitted or newly created (**d**,**p**) signature*

If the query signature (**d**,**p**) is created from an iLINCS dataset, or directly uploaded by the user, the connectivity with a precomputed iLINCS signature, (**d**^{L},**p**^{L}), is calculated as the weighted correlation[10] between the vectors of log-differential expressions:

$$r_{\boldsymbol{w}}(\mathbf{d}\;,\mathbf{d}_L) = \frac{\sum_{i=1}^{N}[w_i(d_i-\bar{d})-(d_i^L-\bar{d}^L)]}{\sqrt{\sum_{i=1}^{N}[w_i(d_i-\bar{d})^2]\sum_{i=1}^{N}\left[w_i\left(d_i^L-\bar{d}^L\right)^2\right]}} \qquad (5)$$

with the vector of weights **w**=(w_1,…,w_N) defined as:

$$w_i = -log_{10}(p_i) - log_{10}\left(p_i^L\right) \qquad (6)$$

$$\bar{d} = \sum_{i=1}^{N} \frac{d_i}{N} \; and \; \bar{d}^L = \sum_{i=1}^{N} \frac{{d_i}^L}{N} \qquad (7)$$

The statistical significance of $r_{\boldsymbol{w}}$ is calculated by replacing r with $r_{\boldsymbol{w}}$ in equation (5). The association between the submitted query signature and an iLINCS signature is visualized by an interactive scatter plot of log differential expression levels created by *ggplot2 and plotly* packages as already illustrated in Fig 1. The example of performing a query with a signature created by analysis of an iLINCS dataset using iLINCS GUI is provided in the Supplemental Use Case 3 and the examples for performing the same analyses with iLINCS API are provided in the *usingIlincsApis* RStudio notebook. For a brief description of how to submit a (**d**,**p**) signatures using iLINCS GUI, see the section Submitting signatures for analysis below. Examples of submitting a signature via API are provided in *usingIlincsApis* RStudio notebook.

Query with a user submitted signature **d** *consisting of only log differential expression levels*

When the user-uploaded signature consists of only log differential expression levels (without p-values), the connectivity with iLINCS signatures is again calculated as the weighted correlation[10] between the vectors of log-differential expressions, but the weights for the correlation are based only on the p-values of the iLINCS signatures:

$$w_i = -log_{10}(p_i^L) \qquad (8)$$

The association between the submitted query signature and an iLINCS signature is visualized by an interactive scatter plot of log differential expression levels created by *ggplot2 and plotly* packages as illustrated in Fig 1. Performing the analysis using iLINCS UI and API is the same as in the previous case.

Query with a user submitted (**gl**$_{up}$,**gl**$_{down}$) *signature*

If the query signature uploaded by the user consists of the lists of up- and down-regulated genes ***(*****gl**$_{up}$,**gl**$_{down}$***)***, the connectivity is calculated by assigning -1 to down-regulated and +1 to upregulated genes:

$$d_i = \begin{Bmatrix} 1, if\ gene\ i \in \boldsymbol{gl}_{up} \\ 1, if\ gene\ i \in \boldsymbol{gl}_{down} \end{Bmatrix} \qquad (9)$$

and calculating weighted Pearson's correlation between such vector and iLINCS signatures using in the same way as in the previous section, when user submitted signature ***d*** consisted of only log differential expression levels without p-values. The calculated statistical significance of the correlation in this case is equivalent to the t-test for the difference between differential expression measures of iLINCS signatures between up- and down-regulated genes. The association between the submitted query signature and an iLINCS signature is visualized by an interactive box-plot of log differential expression levels of the iLINCS signature for up- and down- regulated lists of genes created by *ggplot2 and plotly* packages as illustrated in Fig 2. For a brief description of how to submit a *(***gl**$_{up}$,**gl**$_{down}$*)* signatures using iLINCS GUI, see the section Submitting signatures for analysis below. Examples of submitting a signature via API are provided in *usingIlincsApis* RStudio notebook.

Fig 2

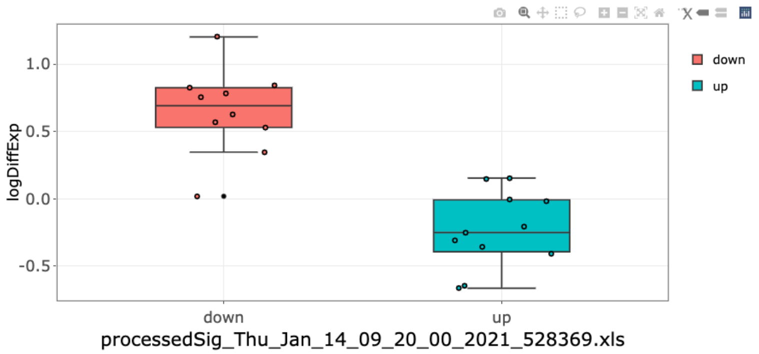

Query with a user submitted $\boldsymbol{gl}$ *signature*

If the query signature is uploaded by the user in a form of a gene list **gl**, the connectivity with an iLINCS signatures (**d**^{L},**p**^{L}) is calculated by treating the list of submitted genes as a gene set, and performing gene set enrichment analysis of the iLINCS signature using the Random Set analysis [11]. Suppose that $\boldsymbol{gl}$ consists of n genes $\mathbf{gl} = (g_1, \ldots, g_n)$. Then the Random Set Z statistics measuring the enrichment of differentially expressed gene from (**d**^{L},**p**^{L}) in **gl** is defined as:

$$z = \frac{\frac{\sum_{i:g_i \in gl} w_i}{n} - \frac{\sum_{i=1}^{N} w_i}{N}}{\sigma} \qquad (10)$$

Where

$$w_i = -\log_{10}(p_i^L) \tag{11}$$

and

$$\sigma = \sqrt{\frac{1}{n}\left(\frac{N-n}{N-1}\right)\left\{\left(\frac{\sum_{i=1}^{N}(d_i^L)^2}{N}\right) - \left(\frac{\sum_{i=1}^{N} d_i^L}{N}\right)^2\right\}} \tag{12}$$

The statistical significance of z is calculated as:

$$\mathrm{p-value}(z) = \mathrm{Probability}(Z > z) \tag{13}$$

Where Z is the standard Normal random variable with mean equal to zero and standard deviation equal to one. The association between the submitted query signature and an iLINCS signature is visualized by an interactive Gene Set Enrichment Analysis (GSEA) plot [12] created with *ggplot2 and plotly* packages as illustrated in Fig 3. For a brief description of how to submit a $\mathbf{gl}$ signatures using iLINCS GUI, see the section Submitting signatures for analysis below. Examples of submitting a signature via API are provided in *usingIlincsApis* RStudio notebook.

Fig 3

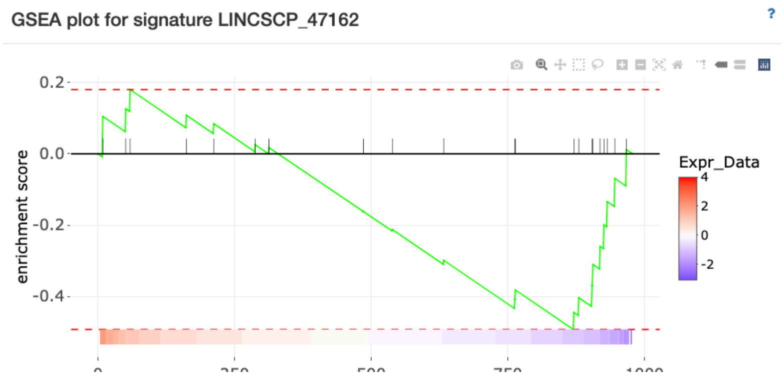

Perturbagen connectivity analysis

The perturbagen connectivity analysis compares the query signature to *all* signatures for a given perturbagen as a group, thus extending the pair-wise connectivity analysis to account for diversity of responses in different cellular contexts. This is accomplished by performing the enrichment analysis of individual connectivity scores between the query signature and set of all L1000 signatures of a perturbagen (for all cell lines, time points and concentrations). The analysis establishes whether the connectivity scores as a set are "unusually" high in comparison to all positive and all negative connectivity scores between the query signature and all iLINCS signatures of the same type based on the Random Set analysis[11].

Suppose that $S = \{\mathbf{s}_\mathrm{i} = (\mathbf{d}_i^L, \mathbf{p}_i^L), i = 1, \dots, T\}$ are all iLINCS perturbation signatures of the same type, such as, for example, L1000 chemical perturbagen (CP) signatures. Suppose that the subset of these signature $C = \{\mathbf{s}_j, j \in K\}\ where\ K \subseteq \{1, \dots, T\}\ and\ |C| = t$ correspond to the perturbagen of interest. Suppose that $CS = \{cs_i = (r_i\ \ , pv_i\ \ ), i = 1, \dots, T\}$ are connectivity scores (r_i) and associated p-values (pv_i) between the query signature and all signatures in S. We defined the positive and negative connectivity significant scores as

$$lpv_i^+ = \begin{Bmatrix} -\log_{10}\left(\frac{pv_i}{2}\right), \text{if gene } r_i > 0 \\ -\log_{10}\left(\frac{1-pv_i}{2}\right), \text{if } gene\ r_i \leq 0 \end{Bmatrix} \tag{14}$$

$$lpv_i^- = \begin{cases} -\log_{10}\left(\frac{pv_i}{2}\right), \text{if gene } r_i < 0 \\ -\log_{10}\left(\frac{1-pv_i}{2}\right), \text{if gene } r_i \geq 0 \end{cases} \quad (15)$$

respectively.

The positive (pc^+) and negative (pc^-) perturbagen connectivity Random Set enrichment is then calculated by replacing w_i with lpv_i^+ and lpv_i^-, respectively, *n* with *t*, *N* with *T* and the $\mathbf{lg}$ list of genes with the set *C* of signatures in equations (10), (11) and (12). Use of the iLINCS GUI to perform perturbagen connectivity analysis is demonstrated in the Supplemental Use Case 3 and examples of using API are provided in *usingIlincsApis* RStudio notebook.

Submitting signatures for analysis

Fig 4. iLINCS GUI for submitting a signature

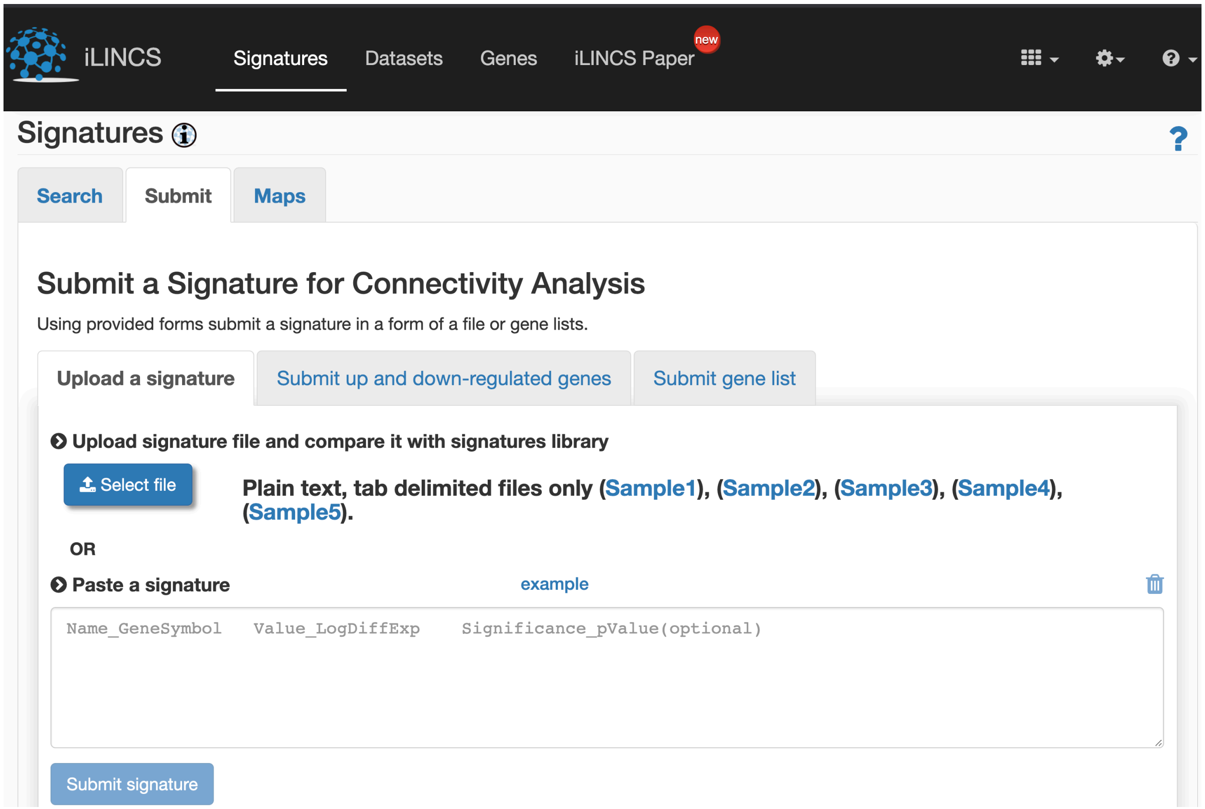

Users can submit signatures for the analysis using the iLINCS GUI or API. The procedures for submitting signatures via API are provided in the *usingIlincsApis* RStudio notebook. iLINCS GUI for submitting signatures is accessed by selecting the “Signatures” workflow and then the “Submit” tab (Fig 4). The signatures can be submitted by either uploading text files, or pasting data into provide boxes. The interface for submitting a signature in the form of a vector of log differential expression and p-values (**d**,**p**), or just a vector of differential expressions, can be accessed by selecting the first tab titled “Upload a signature”. The interface to submit a signature in the form of the lists of up- and down-regulated genes can be accessed by selecting the second tab titled “Submit up and down-regulated genes”. The interface to submit a signature in the form of a simple set of genes is accessed by selecting the third tab titled “Submit gene list”.

iLINCS signature libraries

LINCS L1000 signature libraries

For all LINCS L1000 signature libraries (Consensus gene knockdown signatures (CGS), Overexpression gene signatures and Chemical perturbation signatures) the signatures are constructed by combining the Level 4, population control

signature replicates from two released GEO datasets (GSE92742 and GSE70138) into the Level 5 moderated Z scores (MODZ) by calculating weighted averages as described in the primary publication for the L1000 Connectivity Map dataset [13]. Suppose that $\{\mathbf{z}_1, .., \mathbf{z}_R\}$ is a set of R level 4 replicates of z-score signature. To construct the vector of differential expressions ($\mathbf{d}$) levels for the corresponding level 5 ($\mathbf{d}, \mathbf{p}$) signature, we first calculate the weights associated with each replicate $\{w_1, \dots, w_R\}$ based on their Spearman's correlations with the rest of the replicates:

$$\widetilde{w}_i = \sum_{j \in \{1,\dots,R\}\ and\ i \neq j} \text{Spearman}\left(\mathbf{z}_i, \mathbf{z}_j\right), \dots, i = 1, .., R \quad (16)$$

and

$$w_i = \frac{\widetilde{w}_i}{\sum_{j=1}^{R} \widetilde{w}_j} \quad (17)$$

Moderated Z (MODZ) scores are then calculated as the weighted average of the replicates:

$$\mathbf{d} = \mathbf{MODZ} = \sum_{i=1}^{R} w_i \boldsymbol{z}_i \quad (18)$$

The corresponding p-values are calculated by comparing MODZ of each gene to zero using the Empirical Bayes weighted t-test with the same weights used for calculating MODZs as implemented in the *limma* package. The shRNA and CRISPR knock-down signatures targeting the same gene were further aggregated into Consensus gene signatures (CGSes)[13] using the same procedure and treating now the level 5 MODZ scores of perturbation with a single shRNA or CRISPR guide as the experimental replicate.

Only signatures showing evidence of being reproducible by having the 75th quantile of pairwise Spearman's correlations of level 4 replicates (Broad institute distil_cc_q75 quality control metric[13]) greater than 0.2 are included in the iLINCS library.

LINCS targeted proteomics signatures

LINCS targeted proteomics signatures iLINCS library contains signatures of chemical perturbations assayed by the quantitative targeted mass spectrometry proteomics P100 assay measuring levels of 96 phosphopeptides and GCP assay against ~60 probes that monitor combinations of post-translational modifications on histones[14]. The level 4 Differential Quantification (DIFF) Z-score normalized experimental replicates were downloaded from https://panoramaweb.org/LINCS. The differential expression vector ($\mathbf{d}$) is calculated as a simple average of experimental replicates and the vector of p-values is calculated by comparing the average expression levels for each probe to zero using the simple t-test.

Disease related signatures

Transcriptional signatures in this library are constructed by comparing sample groups within the collection of curated public domain transcriptional dataset (GEO DataSets collection)[15]. For each dataset, we selected each factor with at least two levels. For each pair of level with at least 2 microarray replicates, a signature was constructed by calculating $\log_2$ differential expressions and associated p-values for all genes using Empirical Bayes linear model implemented in the *limma* package[5]. Signatures were subsequently curated to exclude some obviously not interesting results, such as comparisons between technical replicates of biological samples obtained from the same individual.

ENCODE transcription factor binding signatures

ENCODE transcription factor binding signatures is the only signature library in iLINCS not consisting of differential expression levels of genes and proteins. Instead, ENCODE signature consist of genome-wide transcription factor (TF) binding signatures constructed by applying the TREG methodology to ENCODE ChiP-seq[16]. Each signature consists of scores and probabilities of regulation by the given TF in the specific context (cell line and treatment) for each gene in the genome. The details of constructing these signatures are provide in the *TREG* paper[16].

Connectivity Map Signatures

Transcriptional signatures of perturbagen activity constructed based on the version 2 of the original Connectivity Map dataset using Affymetrix expression arrays[17]. Each signature consists of differential expressions and associated p-values for all genes when comparing perturbagen treated cell lines with appropriate controls calculated using the Empirical Bayes linear model as implemented in the R *limma* package. The details of iLICNS Connectivity Map signature library construction have been described in a previous publication[17].

DrugMatrix signatures

DrugMatrix signature library consists of toxicogenomic signatures of over 600 different therapeutic, industrial, or environmental chemicals at both non-toxic and toxic doses[18] in different rat tissues using GE Codelink rat array and the Affymetrix whole genome 230 2.0 rat GeneChip array. DrugMatrix is maintained by the National Toxicology Program (NTP)[19]. DrugMatrix was downloaded from the NTP web site (https://ntp.niehs.nih.gov/data/drugmatrix/) and signatures are created by extracting the treatment metadata, and already created genome-wide differential gene expression levels and associated p-values.

Transcriptional signatures from EBI Expression Atlas

Expression Atlas signature library consitst of all mouse, rat and human differential expression signatures and associated p-values from manually curated comparisons in the Expression Atlas[20]. The latest atlas (atlas-latest-data.tar.gz) was downloaded from https://www.ebi.ac.uk/gxa/download.html on May, 19 2020, and pre-computed signatures (sample metadata, log differential expression levels and associated p-values) were extracted for all available signatures.

Cancer therapeutics response signatures

Cancer therapeutics response signatures were created by combining transcriptional data with drug sensitivity data from the Cancer Therapeutics Response Portal (CTRP) project[21]. Signatures were created separately for each tissue/cell lineage in the dataset by comparing gene expression between the five cell lines of that lineage that were most and five that were least sensitive to a given drug area as measured by the concentration-response curve (AUC) using two-sample t-test.

Pharmacogenomics transcriptional signatures

Signatures in the iLINICS Pharmacogenomics transcriptional signatures library were created by calculating differential gene expression levels and associated p-value between cell-lines treated with anti-cancer drugs and the corresponding controls in two separate projects: The NCI Transcriptional Pharmacodynamics Workbench (NCI-TPW)[22] and the Plate-seq project dataset[23]. For the Plate-seq project, the raw sequencing data was downloaded from GEO and SRA (GSE97460) and processed with the GREIN processing pipeline [6]. The signatures were constructed by differential expression analysis between drug treated samples (for a specific drug, concentration, cell line combination) and corresponding cell line specific DMSO control samples using the negative binomial statistical model as implemented in the *edgeR* [7]. For the NCI-TPW signatures, Affymetrix microarray data was downloaded from GEO (GSE116438) and signatures were constructed by differential expression analysis between drug treated samples (for a specific drug-concentration-cell line-time point combination) and the cell line specific control untreated samples using the Empirical Bayes linear model as implemented in the *limma* package[5].

Analytical tools, web applications and web resources

Signatures analytics in iLINCS is facilitated via native R, Java, JavaScript and Shiny applications, and via API connections to external web application and services. Brief listing of analysis and visualization tools is provided here. The overall structure of iLINCS is described in the Supplemental Results.

Gene list enrichment analysis is facilitated by directly submitting lists of gene to any of the three prominent enrichment analysis web tools: Enrichr[24], DAVID[25], ToppGene[26]. The manipulation and selection of list of signature genes is facilitated via an interactive volcano plot JavaScript application (shown in Supplemental Workflow 3).

Pathway analysis is facilitated through general purpose enrichment tool (Enrichr, DAVID, ToppGene). The enrichment analysis of Reactome pathways is also facilitated via connection to Reactome online tool[27]. Pathway analyses are also implemented in internal R routines for SPIA analysis[28] of KEGG pathways and general visualization of signatures in the context of KEGG pathways using the KEGG API[29].

In performing all tests, the list of genes identified in iLINCS analysis is submitted to individual tools. The gene list to be submitted for the analysis can be identified in four different ways:

- Differentially expressed genes or proteins identified from pre-computed signatures, user submitted, or newly created signature (iLINCS GUI use demonstrated in the Supplemental Use Case 2 and 3)
- Co-expressed genes identified via Morpheus cluster analysis of a set of signatures in the "Group analysis" iLINCS workflow (iLINCS GUI use demonstrated in the Supplemental Use Case 3)
- List of genes genetically perturbed in the selected sets of signatures (iLINCS GUI use demonstrated in the Supplemental Use Case 2)
- List of genes chemically perturbed in the selected sets of signatures (iLINCS GUI use demonstrated in the Supplemental Use Case 2)

Network analysis is facilitated by submitting lists of genes to Genemania[30]. The gene lists used in such analysis are created in the same way as the gene lists used for *Pathway analysis.*

Heatamap visualizations are facilitated by native iLINCS applications: Java based FTreeView[31], modified version of the JavaScript based Morpheus[32], a Shiny based HeatMap application utilizing the *ComplexHeatmap* package [33], and by connection to the web application Clustergrammer[34] (iLINCS GUI use demonstrated in the Supplemental Use Case 3) .

Dimensionality reduction analysis (PCA and t-SNE[35]) and visualization of high-dimensional relationship via interactive 2D and 3D scatter plots is facilitated via internal iLINCS Shiny applications. t-SNE analysis is performed using the *Rtsne* package[36] and PCA analysis is performed using the *prcomp* function in the *stats* package [37]. The visualization of the reduced dimensionality data vectors is implemented using the using *plotly* [38] and pairs3D R packages.

Interactive box-plots, scatter plots, GSEA plots, bar charts and pie charts used throughout iLINCS are implemented using R *ggplot*[39] and *plotly*[38].

Additional analysis are provided by connection X2K Web[40] (inference of upstream regulatory networks from signature genes), L1000FWD[41] (connectivity with signatures constructed using characteristic dimension methodology), STITCH[42] (visualization of drug-target networks), piNET[43] (visualization of gene-to-pathway relationships for signature genes).

Additional information about drugs, genes and proteins are provided by links to, LINCS Data Portal[44], ScrubChem[45], PubChem[46], Harmonizome[47], GeneCards[48], and several other only databases.

Gene and protein expression dataset collections

iLINCS backend databases provide access to more than 11,000 pre-processed gene and protein expression datasets that can be used to create and analyze gene and expression protein signatures. Datasets are thematically organized into eight collections with some datasets assigned to multiple collections. User can search all datasets or browse datasets by collection.

LINCS collection: Datasets generated by the LINCS data and signature generation centers[49]

TCGA collection: Gene expression (RNASeqV2), protein expression (RPPA), and copy number variation data generated by TCGA project[50]

GDS collection: A curated collection of GEO Gene Datasets (GDS)[15]

Cancer collection: An ad-hoc collection of cancer related genomics and proteomic datasets

Toxicogenomics collection: An ad-hoc collection of toxicogenomics datasets

RPPA collection: An ad-hoc collection of proteomic datasets generated by Reverse Phase Protein Array assay[4]

GREIN collection: Complete collection of preprocessed human, mouse and rat RNA-seq data in GEO provided by the GEO RNA-seq Experiments Interactive Navigator (GREIN)[6]

Reference collection: An ad-hoc collection of important gene expression datasets.

Reference List

1 Maglott, D., Ostell, J., Pruitt, K. D. & Tatusova, T. Entrez Gene: gene-centered information at NCBI. *Nucleic Acids Res.* **33**, D54-D58 (2005).
2 Li, B. & Dewey, C. N. RSEM: accurate transcript quantification from RNA-Seq data with or without a reference genome. *BMC Bioinformatics* **12**, 323, doi:10.1186/1471-2105-12-323 (2011).
3 Mermel, C. H. *et al.* GISTIC2.0 facilitates sensitive and confident localization of the targets of focal somatic copy-number alteration in human cancers. *Genome Biology* **12**, R41, doi:10.1186/gb-2011-12-4-r41 (2011).
4 Tibes, R. *et al.* Reverse phase protein array: validation of a novel proteomic technology and utility for analysis of primary leukemia specimens and hematopoietic stem cells. *Mol Cancer Ther* **5**, 2512-2521, doi:10.1158/1535-7163.MCT-06-0334 (2006).
5 Smyth, G. K. in *Bioinformatics and Computational Biology Solutions using R and Bioconductor* (eds R. Gentleman *et al.*) 397-420 (Springer, 2005).
6 Mahi, N. A., Najafabadi, M. F., Pilarczyk, M., Kouril, M. & Medvedovic, M. GREIN: An Interactive Web Platform for Re-analyzing GEO RNA-seq Data. *Sci Rep* **9**, 7580, doi:10.1038/s41598-019-43935-8 (2019).
7 Robinson, M. D., McCarthy, D. J. & Smyth, G. K. edgeR: a Bioconductor package for differential expression analysis of digital gene expression data. *Bioinformatics* **26**, 139-140, doi:10.1093/bioinformatics/btp616 (2010).
8 Iwata, M., Sawada, R., Iwata, H., Kotera, M. & Yamanishi, Y. Elucidating the modes of action for bioactive compounds in a cell-specific manner by large-scale chemically-induced transcriptomics. *Scientific Reports* **7**, 40164, doi:10.1038/srep40164 (2017).
9 Cheng, J. *et al.* in *Pac. Symp. Biocomput.* 5-16 (World Scientific).
10 Engreitz, J. *et al.* Content-based microarray search using differential expression profiles. *BMC Bioinformatics* **11**, 603 (2010).
11 Newton, M. A., Quinatan, F. A., den Boon, J. A., Sengupta, S. & Ahlquist, P. Random-set methods identify distinct aspects of the enrichment signal in gene-set analysis. *The Annals of Applied Statistics* **1**, 85-106 (2007).
12 Subramanian, A. *et al.* Gene set enrichment analysis: a knowledge-based approach for interpreting genome-wide expression profiles. *Proc Natl Acad Sci U S A* **102**, 15545-15550, doi:10.1073/pnas.0506580102 (2005).
13 Subramanian, A. *et al.* A Next Generation Connectivity Map: L1000 Platform and the First 1,000,000 Profiles. *Cell* **171**, 1437-1452 e1417, doi:10.1016/j.cell.2017.10.049 (2017).
14 Abelin, J. G. *et al.* Reduced-representation Phosphosignatures Measured by Quantitative Targeted MS Capture Cellular States and Enable Large-scale Comparison of Drug-induced Phenotypes. *Mol Cell Proteomics* **15**, 1622-1641, doi:10.1074/mcp.M116.058354 (2016).
15 Barrett, T. *et al.* NCBI GEO: archive for high-throughput functional genomic data. *Nucleic Acids Res* **37**, D885-890, doi:10.1093/nar/gkn764 (2009).
16 Chen, J. *et al.* Genome-wide signatures of transcription factor activity: connecting transcription factors, disease, and small molecules. *PLoS computational biology* **9**, e1003198, doi:10.1371/journal.pcbi.1003198 (2013).
17 Freudenberg, J. M., Sivaganesan, S., Phatak, M., Shinde, K. & Medvedovic, M. Generalized random set framework for functional enrichment analysis using primary genomics datasets. *Bioinformatics* **27**, 70-77, doi:10.1093/bioinformatics/btq593 (2011).
18 Ganter, B. *et al.* Development of a large-scale chemogenomics database to improve drug candidate selection and to understand mechanisms of chemical toxicity and action. *J Biotechnol* **119**, 219-244, doi:10.1016/j.jbiotec.2005.03.022 (2005).

19 Auerbach, S. DrugMatrix® and ToxFX® Coordinator National Toxicology Program. *National Toxicology Program: Dept of Health and Human Services*.
20 Papatheodorou, I. *et al.* Expression Atlas: gene and protein expression across multiple studies and organisms. *Nucleic Acids Res* **46**, D246-D251, doi:10.1093/nar/gkx1158 (2018).
21 Rees, M. G. *et al.* Correlating chemical sensitivity and basal gene expression reveals mechanism of action. *Nat Chem Biol* **12**, 109-116, doi:10.1038/nchembio.1986 (2016).
22 Monks, A. *et al.* The NCI Transcriptional Pharmacodynamics Workbench: A Tool to Examine Dynamic Expression Profiling of Therapeutic Response in the NCI-60 Cell Line Panel. *Cancer Res* **78**, 6807-6817, doi:10.1158/0008-5472.CAN-18-0989 (2018).
23 Bush, E. C. *et al.* PLATE-Seq for genome-wide regulatory network analysis of high-throughput screens. *Nat Commun* **8**, 105, doi:10.1038/s41467-017-00136-z (2017).
24 Kuleshov, M. V. *et al.* Enrichr: a comprehensive gene set enrichment analysis web server 2016 update. *Nucleic Acids Res* **44**, W90-97, doi:10.1093/nar/gkw377 (2016).
25 Dennis, G., Jr. *et al.* DAVID: Database for Annotation, Visualization, and Integrated Discovery. *Genome Biol* **4**, P3 (2003).
26 Chen, J., Bardes, E. E., Aronow, B. J. & Jegga, A. G. ToppGene Suite for gene list enrichment analysis and candidate gene prioritization. *Nucleic Acids Res* **37**, W305-311, doi:10.1093/nar/gkp427 (2009).
27 Fabregat, A. *et al.* The Reactome pathway Knowledgebase. *Nucleic Acids Res* **44**, D481-487, doi:10.1093/nar/gkv1351 (2016).
28 Tarca, A. L. *et al.* A novel signaling pathway impact analysis. *Bioinformatics* **25**, 75-82, doi:10.1093/bioinformatics/btn577 (2009).
29 Kanehisa, M., Furumichi, M., Tanabe, M., Sato, Y. & Morishima, K. KEGG: new perspectives on genomes, pathways, diseases and drugs. *Nucleic Acids Res* **45**, D353-D361, doi:10.1093/nar/gkw1092 (2017).
30 Warde-Farley, D. *et al.* The GeneMANIA prediction server: biological network integration for gene prioritization and predicting gene function. *Nucleic Acids Res* **38**, W214-220, doi:10.1093/nar/gkq537 (2010).
31 Freudenberg, J. M., Joshi, V. K., Hu, Z. & Medvedovic, M. CLEAN: CLustering Enrichment ANalysis. *BMC Bioinformatics* **10**, 234 (2009).
32 *Morpheus*, <https://software.broadinstitute.org/morpheus> (
33 Gu, Z., Eils, R. & Schlesner, M. Complex heatmaps reveal patterns and correlations in multidimensional genomic data. *Bioinformatics* **32**, 2847-2849, doi:10.1093/bioinformatics/btw313 (2016).
34 Fernandez, N. F. *et al.* Clustergrammer, a web-based heatmap visualization and analysis tool for high-dimensional biological data. *Sci Data* **4**, 170151, doi:10.1038/sdata.2017.151 (2017).
35 Maaten, L. v. d. & Hinton, G. Visualizing data using t-SNE. *Journal of machine learning research* **9**, 2579-2605 (2008).
36 Krijthe, J., van der Maaten, L. & Krijthe, M. J. (GitHub, 2018).
37 R: A Language and Environment for Statistical Computing (R Foundation for Statistical Computing, Vienna, Austria, 2016).
38 Sievert, C. *et al.* plotly: Create Interactive Web Graphics via 'plotly. js'. *R package version* **4**, 110 (2017).
39 Wickham, H. *ggplot2: elegant graphics for data analysis*. (Springer, 2016).
40 Clarke, D. J. B. *et al.* eXpression2Kinases (X2K) Web: linking expression signatures to upstream cell signaling networks. *Nucleic Acids Res* **46**, W171-W179, doi:10.1093/nar/gky458 (2018).
41 Wang, Z., Lachmann, A., Keenan, A. B. & Ma'ayan, A. L1000FWD: fireworks visualization of drug-induced transcriptomic signatures. *Bioinformatics* **34**, 2150-2152, doi:10.1093/bioinformatics/bty060 (2018).
42 Kuhn, M. *et al.* STITCH 2: an interaction network database for small molecules and proteins. *Nucleic Acids Res* **38**, D552-556, doi:10.1093/nar/gkp937 (2010).
43 Meller, J. *et al.* piNET: a versatile web platform for downstream analysis and visualization of proteomics data. *Nucleic Acids Research* **48**, W85-W93, doi:10.1093/nar/gkaa436 (2020).
44 Koleti, A. *et al.* Data Portal for the Library of Integrated Network-based Cellular Signatures (LINCS) program: integrated access to diverse large-scale cellular perturbation response data. *Nucleic Acids Res* **46**, D558-D566, doi:10.1093/nar/gkx1063 (2018).
45 Harris, J. B. in *Bioinformatics and Drug Discovery* 37-47 (Springer, 2019).

46 Kim, S. *et al.* PubChem Substance and Compound databases. *Nucleic Acids Res* **44**, D1202-1213, doi:10.1093/nar/gkv951 (2016).
47 Rouillard, A. D. *et al.* The harmonizome: a collection of processed datasets gathered to serve and mine knowledge about genes and proteins. *Database (Oxford)* **2016**, doi:10.1093/database/baw100 (2016).
48 Safran, M. *et al.* GeneCards Version 3: the human gene integrator. *Database (Oxford)* **2010**, baq020, doi:10.1093/database/baq020 (2010).
49 Keenan, A. B. *et al.* The Library of Integrated Network-Based Cellular Signatures NIH Program: System-Level Cataloging of Human Cells Response to Perturbations. *Cell Syst* **6**, 13-24, doi:10.1016/j.cels.2017.11.001 (2018).
50 Consortium, T. Comprehensive molecular portraits of human breast tumours. *Nature* **490**, 61-70 (2012).

Quality control assessment of iLINCS data and analysis procedures

Table of Contents

Quality Control of Signature Libraries* *22
LINCS L1000 signatures 22
Comparison of LINCS signatures hosted by iLINCS to those released by in GEO (GSE92742 and GSE70138) 22
The accuracy of iLINCS signatures in the backend database and retrieved through iLINCS API 23
Comparison of LINCS signatures hosted by iLINCS to those hosted by clue.io 23
Quality Control of Omics Datasets* *23
Microarray datasets 23
RNA-seq datasets 24
The accuracy and the consistence of iLINCS analysis results* *25
Accuracy of connectivity analysis 25
Accuracy of pre-computed connectivity scores 25
The accuracy and the consistency of connectivity scores calculated for newly created and user-submitted signatures 25
Accuracy of analysis of iLINCS datasets to create a signature 27

Quality Control of Signature Libraries

LINCS L1000 signatures

The vast majority of omics signatures in iLINCS are derived from the LINCS L1000 data. iLINCS signatures were constructed by following the Broad Institute strategy outlined the original publication (details in Methods and Supplemental Methods). The "level 4" z scores data was downloaded from GEO and MODZ "level 5" signatures were calculated as a weighted average across level 4 replicated plates (https://clue.io/connectopedia/data_levels)[1]. In our internal test, connectivity analysis of the same treatments (perturbagen, time, and concentration matched) across different cell lines for level 5 signatures consistently yields the area under the ROC curve of 0.95, or greater (Supplemental Results 2: Benchmarking methods for connectivity analysis and comparisons to other web resources). This indicates that overall precision of LINCS L1000 signatures in iLINCS in correctly connecting biologically similar signatures is very high. Here we perform technical tests of accuracy of the signatures in iLINCS by comparing them to the released Level 5 signatures in GEO. We perform this comparison by comparing off-line results all signatures produced by our pipeline to signatures released in GEO, and by randomly selecting and downloading 100 signatures via iLINCS API and confirming again that they are identical to the signatures released in GEO. We also perform a direct connectivity analysis of iLICNS with LINCS signatures via Broad clue.io system.

Comparison of LINCS signatures hosted by iLINCS to those released by in GEO (GSE92742 and GSE70138)

To assess the accuracy of our processing and the accuracy of the signatures in the database, we compared iLINCS CP signatures with those released to GEO by the Broad institute. It is important to notice that in our processing we used the latest Broad algorithm to computing the MODZs (https://github.com/cmap/cmapM). As for the datasets released in GEO,

a portion of the older dataset (GSE92742) used an older version of the pipeline while the new dataset (GSE70138) used the latest pipeline which is identical to our pipeline. This was reflected in the results of our comparisons. For signatures in the second release (GSE70138), 100% signatures from iLINCS were identical to corresponding Level 5 signatures in GEO. For signatures in the pilot phase data release (GSE92742), 7.5% signatures had the correlation <0.99, which could be considered as slightly different and is consistent with the use of a slightly different processing pipeline.

The accuracy of iLINCS signatures in the backend database and retrieved through iLINCS API

To make sure that the L1000 signatures we computed were accurately represented in the iLINCS backend databases and in the connectivity analysis, we Use API to download signatures from iLINCS and correlate with signatures in the GEO. A random set of 100 signatures was downloaded from iLINCS using APIs and compared to the GEO signatures. The comparison was made using three measures of similarity, Pearson's correlation, total absolute deviation and maximum absolute deviation. If $\boldsymbol{s}_1 = \left(g_{1,1}, \dots, g_{978,1}\right)\ and\ \boldsymbol{s}_2 = \left(g_{1,2}, \dots, g_{978,2}\right)$ are two signatures where $g_{i,k}$ is the MODZ score for jth gene and ith signatures, then the vector of absolute deviation is defined as $\mathbf{ad} = (\left|g_{1,1} - g_{2,1}\right|, \dots, \left|g_{1,978} - g_{2,978}\right|)$, the total absolute deviation is defined as $adt = \sum_{i=1}^{978} ad_i$ and maximum absolute deviation is defined as $adm = \max\left(ad_1, \dots, ad_{978}\right)$. The results for this analysis is provided in the Supplementary QC Table showing perfect correspondence between signatures in iLINCS and signatures released in GEO.

Comparison of LINCS signatures hosted by iLINCS to those hosted by clue.io

To compare LINCS signatures and connectivity methods from iLINCS to clue.io, we selected 100 random iLINCS L1000 CP (chemical perturbagen) signatures and used them to query clue.io. For each iLINCS signature we extracted a list of the 100 most up-regulated and the 100 most down-regulated landmark genes and submitted these gene lists to clue.io via their L1000 query API. We then assessed each signature's "self-connectivity" by the rank of its connectivity score with the corresponding clue.io signature among all clue.io signatures. A rank of 1 indicates that the iLINCS signature was most connected to its corresponding clue.io signature. Of the 100 signatures tested, all except one had virtually perfect association with clue.io signatures (Supplemental QC table). 98 signatures had a perfect "self-connectivity" rank of 1, one (ASG001_PC3_6H:BRD-A19500257-001-04-7:0.08) had rank of 3 (out of >1 million) which is still virtually perfect association. One signature (LJP006_HEPG2_24H:M01) showed poor association with the corresponding clue.io signature. We assessed the quality of this signature in iLINCS by comparing it to the corresponding signature in the released GEO dataset (GSE70138) and verified that iLINCS signature is identical to the GEO signature. Overall, these results indicate that L1000 signatures in iLINCS are accurate and consistent with the L1000 signatures in clue.io.

Quality Control of Omics Datasets

The omics datasets in iLINCS are uploaded by two distinct processes: 1) The batch processing of microarray and RNA-seq data in GEO and 2) By curated processing of individual datasets. The vast majority of the datasets in iLINCS were processed the first, batch processing approach. In the situations when individual datasets are processed and curated (second approach), each such dataset is associated with a unique processing and uploading script, and the data and metadata are inspected and compared with the original source after it is uploaded. Here we report a systematic quality control we performed for more than 11,000 datasets that were processed using batch processing of microarray and RNA-seq datasets. The quality control of these iLINCS datasets was performed by comparing them to results obtained by other group's independent processing efforts. The QC of microarray GEO GDS datasets data and metadata was performed by comparing them to EBI Expression Atlas[2] and RNA-seq datasets were compared to the recount2 project datasets[3].

Microarray datasets

We assessed systematically the quality and consistency of the GDS datasets in iLINCS by re-creating EBI Expression Atlas signatures[2] using the GDS datasets in iLINCS and iLINCS API. Through semi-automated search and curation, we identified 150 single factor Expression Atlas signatures that could be perfectly matched to a microarray dataset in the iLINCS collection. The signatures, datasets and matching factors and sample labels are provided in the Supplemental QC Table. This allowed us to re-construct automatically the equivalent signatures directly from the microarray datasets using iLICNS

API. Each of the newly created signature was submitted for the connectivity analysis against all Expression Atlas signatures in iLINCS and the ability to recover the correct signature was assessed. In all 150 cases, the correct signature was highly connected to the corresponding Expression Atlas signature and the correct signatures was ranked as the number 1, most connected among all 5,646 Expression Atlas signatures in iLINCS. The fact that the signatures created directly from uploaded GDS datasets are perfect match for EBI Expression Atlas signatures which were constructed by curation and independent analysis of the same datasets indicates that both metadata and expression data for corresponding microarray datasets in iLINCS are consistent with the data deposited in GEO and validates our processing and uploading GEO GDS datasets to iLINCS. Furthermore, this results also validates accuracy of the analysis procedures used to create signatures in iLINCS.

RNA-seq datasets

We assessed systematically the consistency of the *GREIN collection* of preprocessed human, mouse and rat RNA-seq data from GEO pre-processed by the GEO RNA-seq Experiments Interactive Navigator (GREIN)[4] by comparing them to datasets pre-processed by the recount2 project [3]. Despite vastly different processing pipelines, the data exhibited very high level of reproducibility across two collections indicating that GREIN collection is accurate representation of the GEO RNA-seq data.

The comparison between GREIN and recount datasets was performed for common human datasets in both collections. Since recount datasets provides quantifications for individual "runs" and aggregating individual runs into sample specific would require additional manipulations, we focus 18,626 samples from 968 GEO datasets which had one run per sample. The consistency of the processed data was assessed by calculating pairwise Pearson's correlations between all sample GREIN profiles with all recount profiles. A pair of GREIN and recount profiles $(\mathbf{g}_i, \mathbf{r}_j)$ is designated as true positive (TP) if

Fig 1

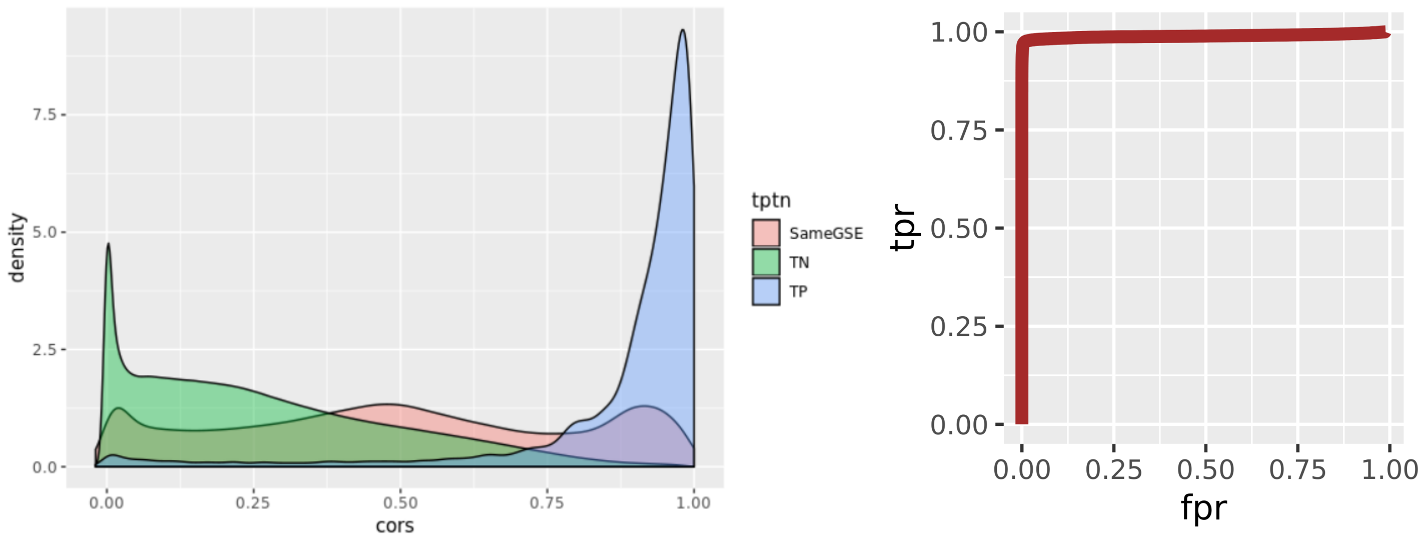

they correspond to the same sample, true negative (TN) if they come from different GEO series, and the pairs corresponding to different samples within the same series as "SameGSE". The distribution of Person's correlations is shown in Fig 1. It indicates very strict separation between the correlations between TP and TN samples. This is also clearly indicated in the associated ROC curves for separating TP from TN samples (Fig1 B). False positive rate (fpr) for each TP pair $(\boldsymbol{g}_i, \boldsymbol{r}_i)$ was calculated as the proportion of TN pairs with higher correlation than $cor(\mathbf{g}_i, \mathbf{r}_i)$. The area under the ROC curve was 0.98 indicating close to perfect separation.

The accuracy and the consistence of iLINCS analysis results

To establish that the accuracy of iLINCS analysis results we implemented the equivalent methodology off-line and cross-references the results produced by iLINCS with results produced by independently implemented methods off-line. We focused on two main aspects of the analyses offered by iLINCS: 1) The connectivity analysis between pre-computed and user submitted signatures; and 2) The methods for creating an omics signature from iLINCS datasets.

Accuracy of connectivity analysis

To systematically and reproducibly assess the accuracy of pre-computed connections between iLINCS signatures and accuracy of the connectivity analysis of newly created and user submitted signatures, we wrote a qulity control (QC) R script (https://github.com/uc-bd2k/ilincsAPI/tree/master/qc) that implements connectivity analysis methods used by iLINCS, performs off-line connectivity analysis, and compares the results to iLINCS results.

Accuracy of pre-computed connectivity scores

The pre-computed connections between all iLINCS signatures are based on the extreme Pearson's correlation of signed significances (Supplemental Methods). For a randomly selected iLINCS signatures, the QC R script uses iLICNS API to identify connected signatures and the value of the extreme Pearson's correlation. Then it again uses iLINCS API to download the query and a randomly selected set of connected signatures and calculates extreme Pearson's correlations off-line. Finally, it compares the correlations returned by the iLINCS query to the off-line calculated correlations. Below is the example output produced by the QC R script for the analysis of iLINCS CP signature LINCSCP_113267 and five randomly selected connected signatures. Results were identical to within what is expected by precision of the data stored in the iLINCS database.

```
[1] For signature: LINCSCP_85346 calculated extreme correlation is:-0.330399839537796 iLINCS query results is: -0.3304

[1] For signature: LINCSCP_14449 calculated extreme correlation is:-0.308913198637522 iLINCS query results is: -0.308913

[1] For signature: LINCSCP_85348 calculated extreme correlation is:-0.297887956989956 iLINCS query results is: -0.297888

[1] For signature: LINCSCP_85349 calculated extreme correlation is:-0.279814638299072 iLINCS query results is: -0.279815

[1] For signature: LINCSCP_209947 calculated extreme correlation is:-0.277738762299301 iLINCS query results is: -0.277739
```

The accuracy and the consistency of connectivity scores calculated for newly created and user-submitted signatures

The user-submitted signature can come in the form of a table of log differential gene expressions and pvalues, (***d*,*p***), a table of only log differential gene expressions (***d***), and the lists of up- and down-regulated genes ($\boldsymbol{gl_{up}},\boldsymbol{gl_{down}}$). We used QC R script to submit the same signature (LINCSCP_113267) for the analysis via iLINCS API in these three forms and compared the results produced by iLINCS to off-line computations by QC R script.

*The accuracy of weighted correlations connectivity analysis for a (**d,p**) signature*

If the query signature is created from an iLINCS dataset, or directly uploaded by the user in the form of a table of log differential gene expressions and pvalues, (**d**,**p**) (Supplemental methods), the connectivity with all iLINCS signatures is calculated as the weighted correlation between the two vectors of log-differential expressions (Supplemental methods Eq 3). For this scenario, we used that same signatures as in previous section (LINCSCP_113267) and submitted it for analysis via iLINCS API as a user submitted signature. The results returned by iLINCS were compared to off-line weighted correlation calculated by QC R script off-line for five randomly selected connected signatures. The weighted correlation coefficient against 5 other signatures is calculated offline and compared with iLINCS results. Results were again identical to within what is expected by precision of the data stored in the iLINCS database.

```
[1] For signature: LINCSCP_85346 calculated weighted correlation is:-0.473201749262842 iLINCS query results
is: -0.4732018163

[1] For signature: LINCSCP_85348 calculated weighted correlation is:-0.447326879788148 iLINCS query results
is: -0.4473269829

[1] For signature: LINCSCP_14449 calculated weighted correlation is:-0.437788573887979 iLINCS query results
is: -0.4377884536

[1] For signature: LINCSCP_11625 calculated weighted correlation is:-0.435372189868698 iLINCS query results
is: -0.435372125

[1] For signature: LINCSCP_85349 calculated weighted correlation is:-0.429771138405948 iLINCS query results
is: -0.4297711733
```

*The accuracy of weighted correlations connectivity analysis for a (**p**) signature*

If the query signature is directly uploaded by the user in the form of a table of log differential gene expressions (***d***) (Supplemental methods), the connectivity with all iLINCS signatures is calculated as the weighted correlation between the two vectors of log-differential expressions with weights being calculated only using p-values of iLINCS signatures (Supplemental methods). We again used that same signatures as in previous section (LINCSCP_113267) and submitted it for analysis via iLINCS API as a user submitted signature consisting only of log differential expressions. The results returned by iLINCS were compared to off-line weighted correlation calculated by QC R script off-line for five randomly selected connected signatures. The weighted correlation coefficient against 5 other signatures is calculated offline and compared with iLINCS results. Results were again identical to within what is expected by precision of the data stored in the iLINCS database.

```
[1] For signature: LINCSCP_11631 calculated weighted correlation is:-0.507595884030785 iLINCS query results
is: -0.5075960534

[1] For signature: LINCSCP_209947 calculated weighted correlation is:-0.500765362989446 iLINCS query results
is: -0.5007654212

[1] For signature: LINCSCP_85346 calculated weighted correlation is:-0.495213570689653 iLINCS query results
is: -0.4952136282

[1] For signature: LINCSCP_11633 calculated weighted correlation is:-0.490393202955426 iLINCS query results
is: -0.4903931816

[1] For signature: LINCSCP_14449 calculated weighted correlation is:-0.481937171739761 iLINCS query results
is: -0.4819370293
```

The accuracy of queries with up- and down-regulated gene lists ($\mathbf{gl}_{up},\mathbf{gl}_{down}$)

If the query signature is directly uploaded by the user in the form of the lists of up- and down-regulated genes ($\boldsymbol{gl}_{up},\boldsymbol{gl}_{down}$) (Supplemental methods), the connectivity with all iLINCS signatures is calculated by assigning -1 to down-regulated and +1 to upregulated genes:

$$d_i = \begin{Bmatrix} 1, \text{if gene } i \in \mathbf{gl}_{up} \\ 1, \text{if gene } i \in \mathbf{gl}_{down} \end{Bmatrix}$$

and calculating weighted Pearson's correlation between such vector and iLINCS signatures in the same way as in the previous section, when user submitted signature ***d*** consisted of only log differential expression levels without p-values. We again used that same signatures as in previous section (LINCSCP_113267), extracted 100 most up- and down-regulated genes and submitted it for analysis via iLINCS API as a user submitted signature. The results returned by iLINCS were compared to off-line weighted correlation calculated by QC R script for five randomly selected connected signatures. Results were again identical to within what is expected by precision of the data stored in the iLINCS database.

```
[1] "For signature: LINCSCP_4770 calculated correlation is:0.594277981689531 iLINCS query results is:
0.5942780464"
```

```
[1] "For signature: LINCSCP_19362 calculated correlation is:0.557703722767745 iLINCS query results is: 0.5577038697"

[1] "For signature: LINCSCP_32084 calculated correlation is:0.555662560888544 iLINCS query results is: 0.5556625943"

[1] "For signature: LINCSCP_918 calculated correlation is:0.552018761801338 iLINCS query results is: 0.5520187776"

[1] "For signature: LINCSCP_26496 calculated correlation is:0.542683999823284 iLINCS query results is: 0.5426839885"
```

Accuracy of analysis of iLINCS datasets to create a signature

The accuracy of the differential expression analysis of an iLICNS dataset to create an omics signature is also demonstrated by the results presented in the Microarray datasets qc section. In the process of validating that iLINCS GDS datasets can be used to accurately re-create signatures that were independently created by the EBI Expression Atlas project, we also validated the accuracy of the analysis procedure used by iLINCS to perform the differential expression analysis between

Reference List

1 Subramanian, A. *et al.* A Next Generation Connectivity Map: L1000 Platform and the First 1,000,000 Profiles. *Cell* **171**, 1437-1452 e1417, doi:10.1016/j.cell.2017.10.049 (2017).

2 Papatheodorou, I. *et al.* Expression Atlas: gene and protein expression across multiple studies and organisms. *Nucleic Acids Res* **46**, D246-D251, doi:10.1093/nar/gkx1158 (2018).

3 Collado-Torres, L. *et al.* Reproducible RNA-seq analysis using recount2. *Nat Biotech* **35**, 319-321, doi:10.1038/nbt.3838 (2017).

4 Mahi, N. A., Najafabadi, M. F., Pilarczyk, M., Kouril, M. & Medvedovic, M. GREIN: An Interactive Web Platform for Re-analyzing GEO RNA-seq Data. *Scientific Reports* **9**, 7580, doi:10.1038/s41598-019-43935-8 (2019).

Use case 1: Detecting and modulating aberrant mTOR pathway signaling

We use mTOR signaling as the prototypical example of using iLINCS to identify chemical perturbagens capable of modulating a known signaling pathway driving the disease process (A and B), using iLINCS in establishing MOA of a chemical perturbagen (C, D, E) and in detecting aberrant signaling in the diseased tissue.

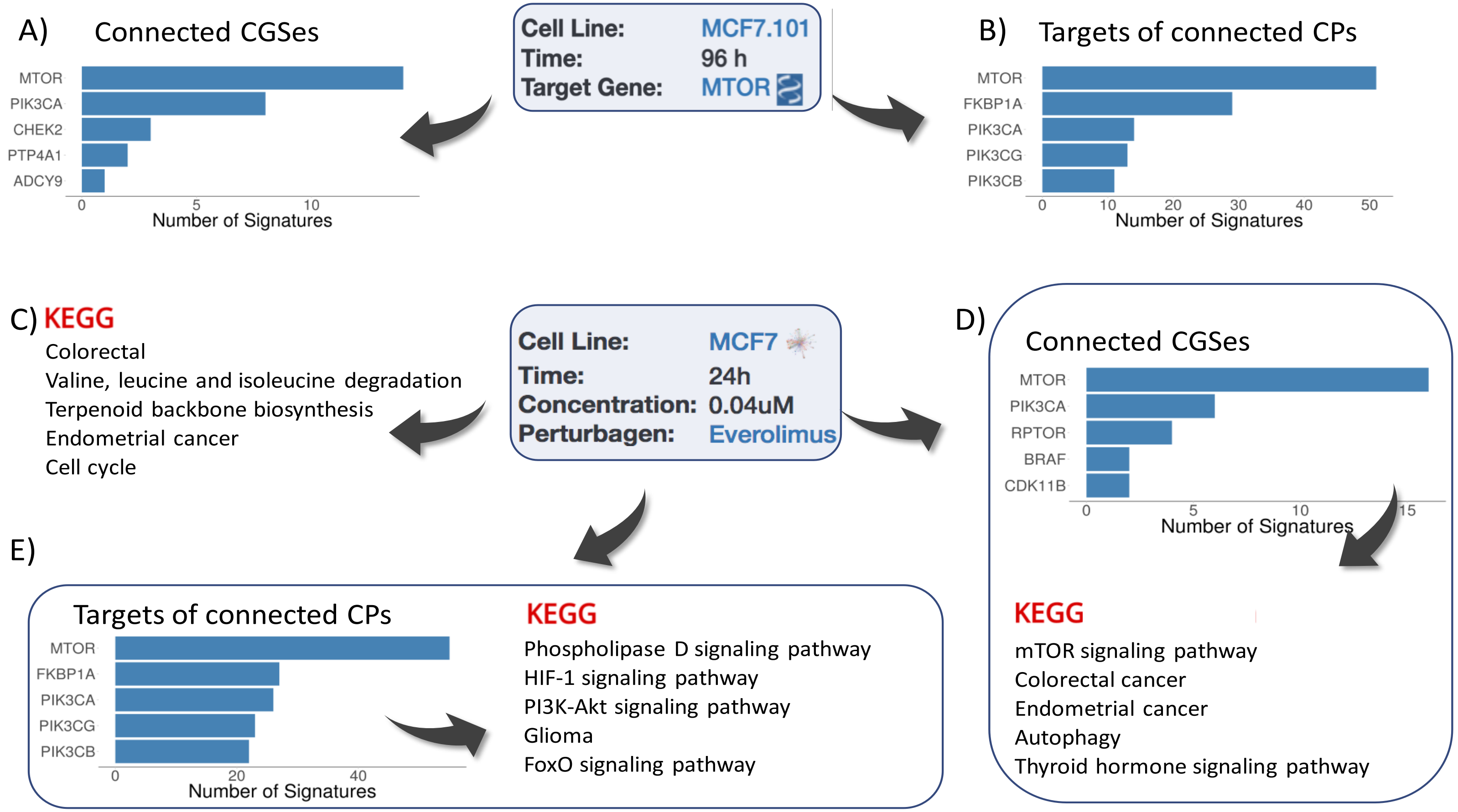

Use case 1 Part 1: Identifying chemical perturbagens emulating genetic perturbation of MTOR protein

First, we search through iLINCS libraries for Consensus Genes Signatures (CGSes) of MTOR knock-down and use the CRISPR CGS in MCF-7 cell line as the query signature. The connectivity analysis identifies 258 LINCS CGSes and 831 CP Signatures with statistically significant correlation with the query signature. Top 100 most connected CGSes are dominated by the signatures of genetic perturbations of MTOR and PIK3CA genes (Fig 2A), whereas all top 5 most frequent inhibition targets of CPs among top 100 most connected CP signatures are MTOR and PIK3 proteins (Fig 2B). Results clearly indicate that the query MTOR CGS is highly specific and sensitive to perturbation of the mTOR pathway and effectively identifies chemical perturbagens capable of inhibiting mTOR signaling. The connected CP signatures also include several chemical perturbagens with highly connected signatures that have not been known to target mTOR signaling providing additional candidate inhibitors.

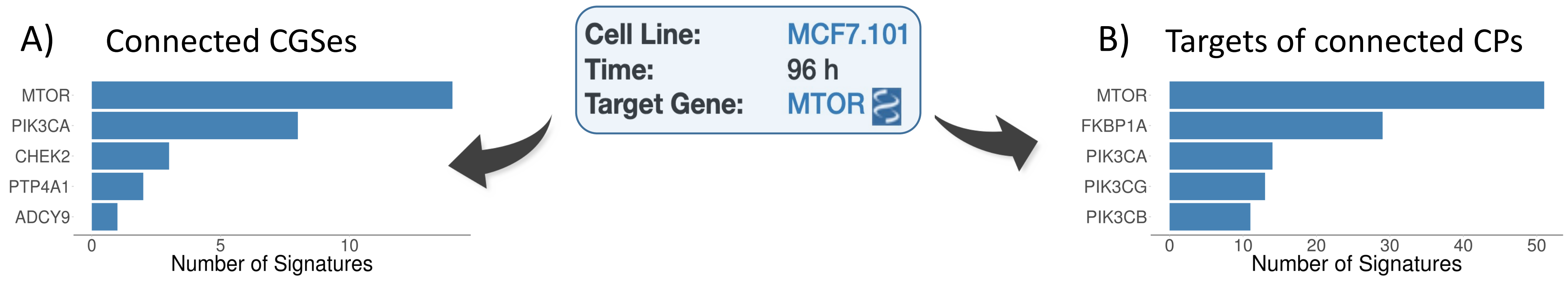

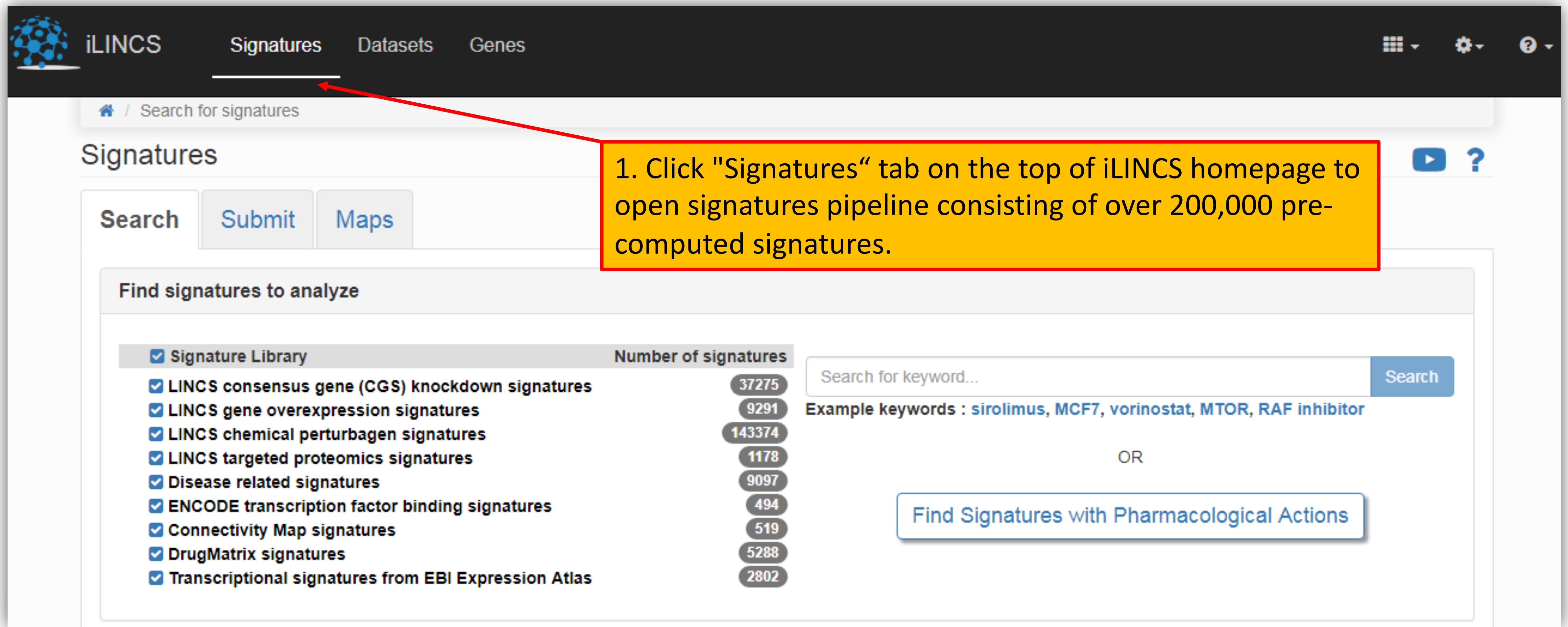

1. Click "Signatures“ tab on the top of iLINCS homepage to open signatures pipeline consisting of over 200,000 pre-computed signatures.

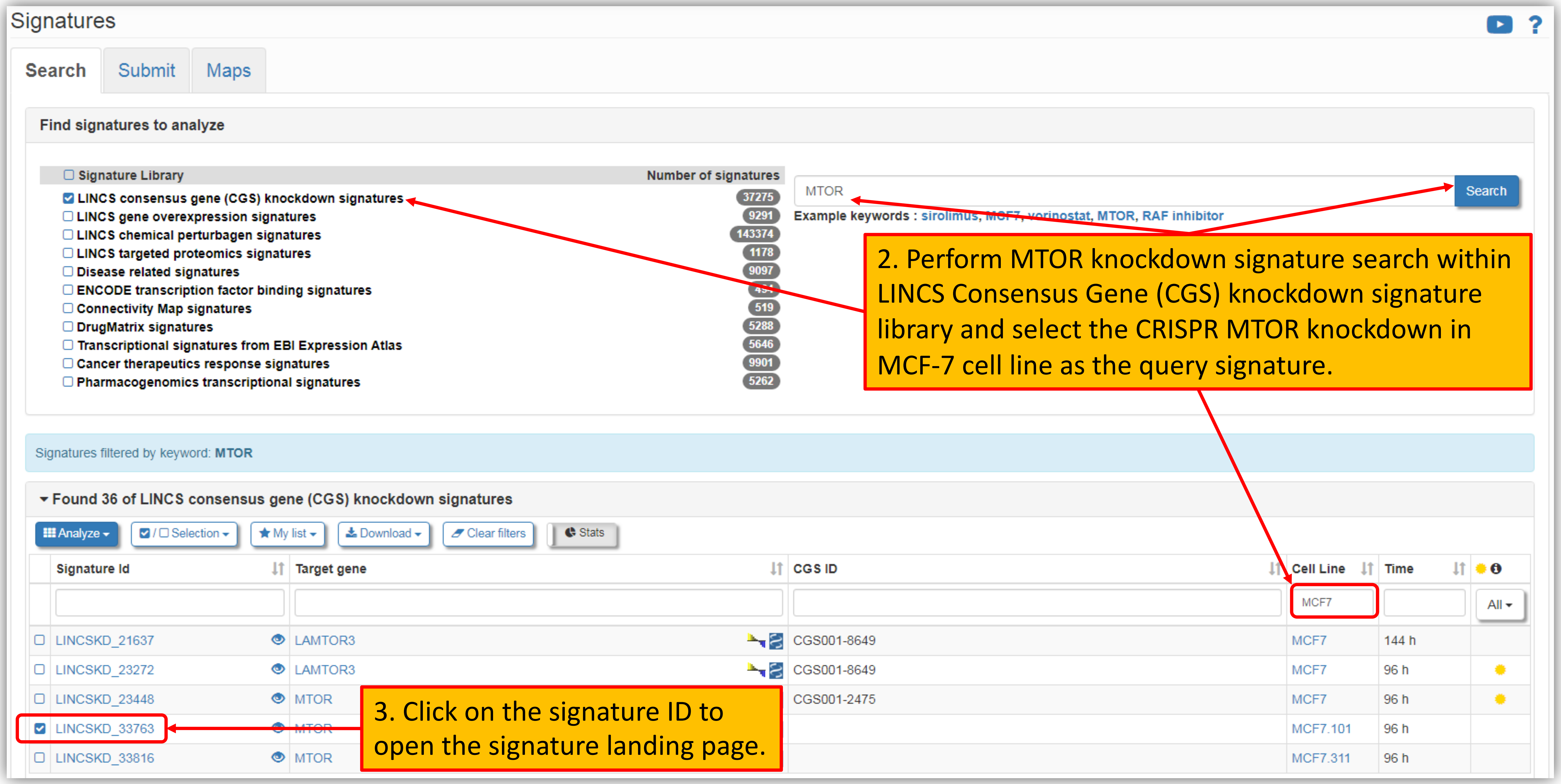
Signatures
Search
Submit
Maps
Find signatures to analyze
Signature Library
Number of signatures
LINCS consensus gene (CGS) knockdown signatures 37275
LINCS gene overexpression signatures 9291
LINCS chemical perturbagen signatures 143374
LINCS targeted proteomics signatures 1178
Disease related signatures 9097
ENCODE transcription factor binding signatures
Connectivity Map signatures 519
DrugMatrix signatures 5288
Transcriptional signatures from EBI Expression Atlas 5646
Cancer therapeutics response signatures 9901
Pharmacogenomics transcriptional signatures 5262
MTOR
Search
Example keywords : sirolimus, MCF7, vorinostat, MTOR, RAF inhibitor
2. Perform MTOR knockdown signature search within LINCS Consensus Gene (CGS) knockdown signature library and select the CRISPR MTOR knockdown in MCF-7 cell line as the query signature.
Signatures filtered by keyword: MTOR
Found 36 of LINCS consensus gene (CGS) knockdown signatures
Analyze
Selection
My list
Download
Clear filters
Stats
Signature Id
Target gene
CGS ID
Cell Line
Time
MCF7
All
LINCSKD_21637 LAMTOR3 CGS001-8649 MCF7 144 h
LINCSKD_23272 LAMTOR3 CGS001-8649 MCF7 96 h
LINCSKD_23448 MTOR CGS001-2475 MCF7 96 h
LINCSKD_33763 MTOR MCF7.101 96 h
LINCSKD_33816 MTOR MCF7.311 96 h
3. Click on the signature ID to open the signature landing page.

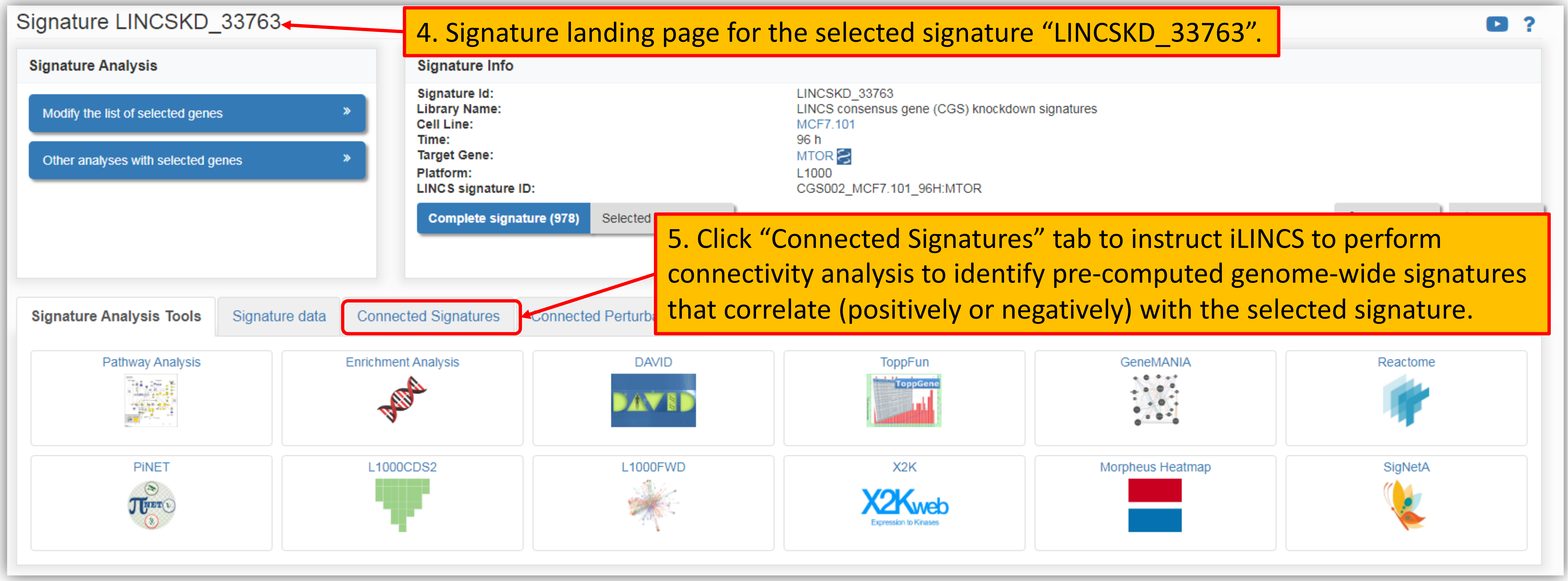
Signature LINCSKD_33763
4. Signature landing page for the selected signature “LINCSKD_33763”.
Signature Analysis
Modify the list of selected genes
Other analyses with selected genes
Signature Info
Signature Id: LINCSKD_33763
Library Name: LINCS consensus gene (CGS) knockdown signatures
Cell Line: MCF7.101
Time: 96 h
Target Gene: MTOR
Platform: L1000
LINCS signature ID: CGS002_MCF7.101_96H:MTOR
Complete signature (978)
Selected
5. Click “Connected Signatures” tab to instruct iLINCS to perform connectivity analysis to identify pre-computed genome-wide signatures that correlate (positively or negatively) with the selected signature.
Signature Analysis Tools
Signature data
Connected Signatures
Connected Perturb
Pathway Analysis
Enrichment Analysis
DAVID
ToppFun
GeneMANIA
Reactome
PiNET
L1000CDS2
L1000FWD
X2K
Morpheus Heatmap
SigNetA
X2Kweb
Expression to Kinases

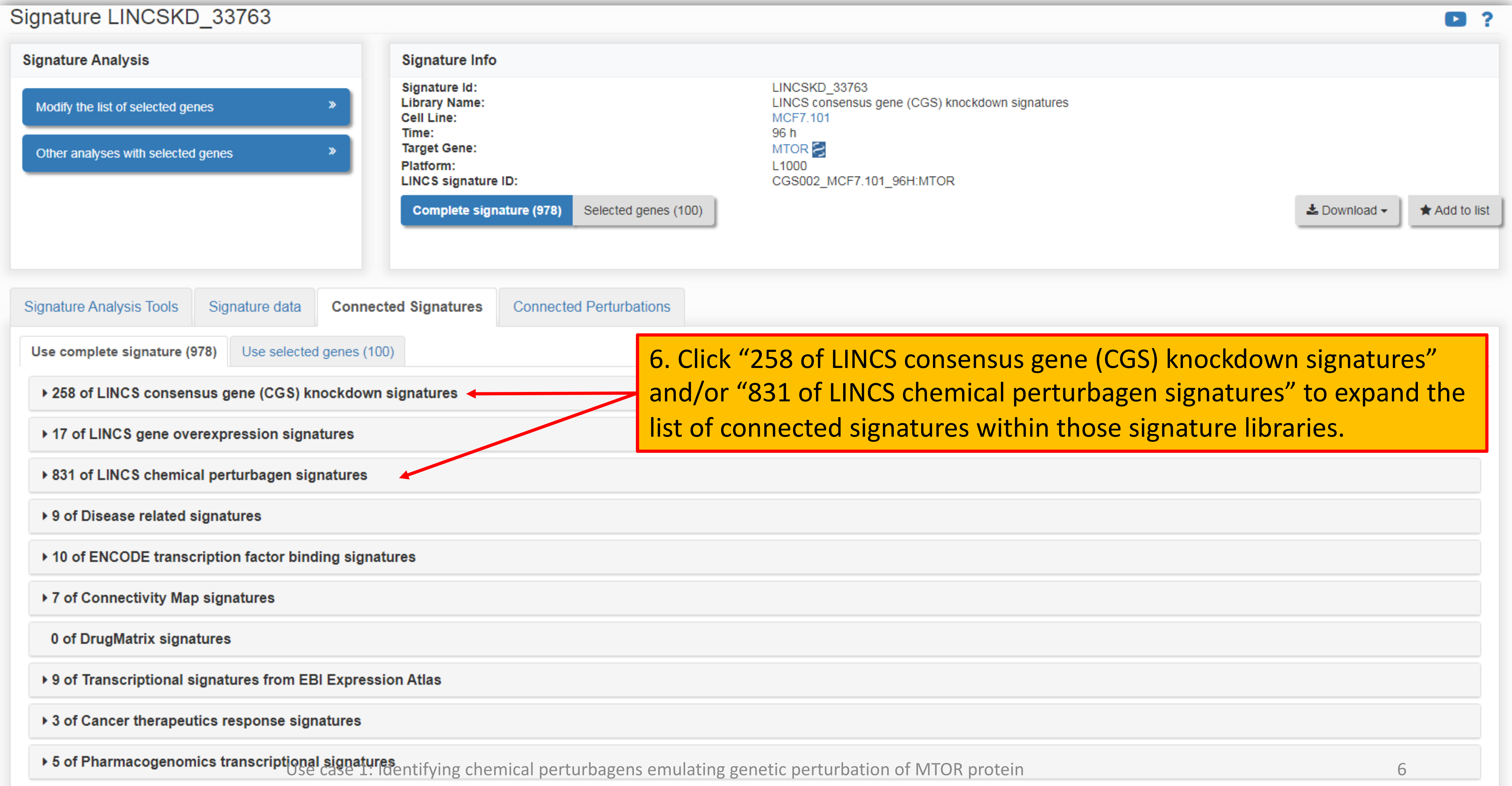

6. Click "258 of LINCS consensus gene (CGS) knockdown signatures" and/or "831 of LINCS chemical perturbagen signatures" to expand the list of connected signatures within those signature libraries.

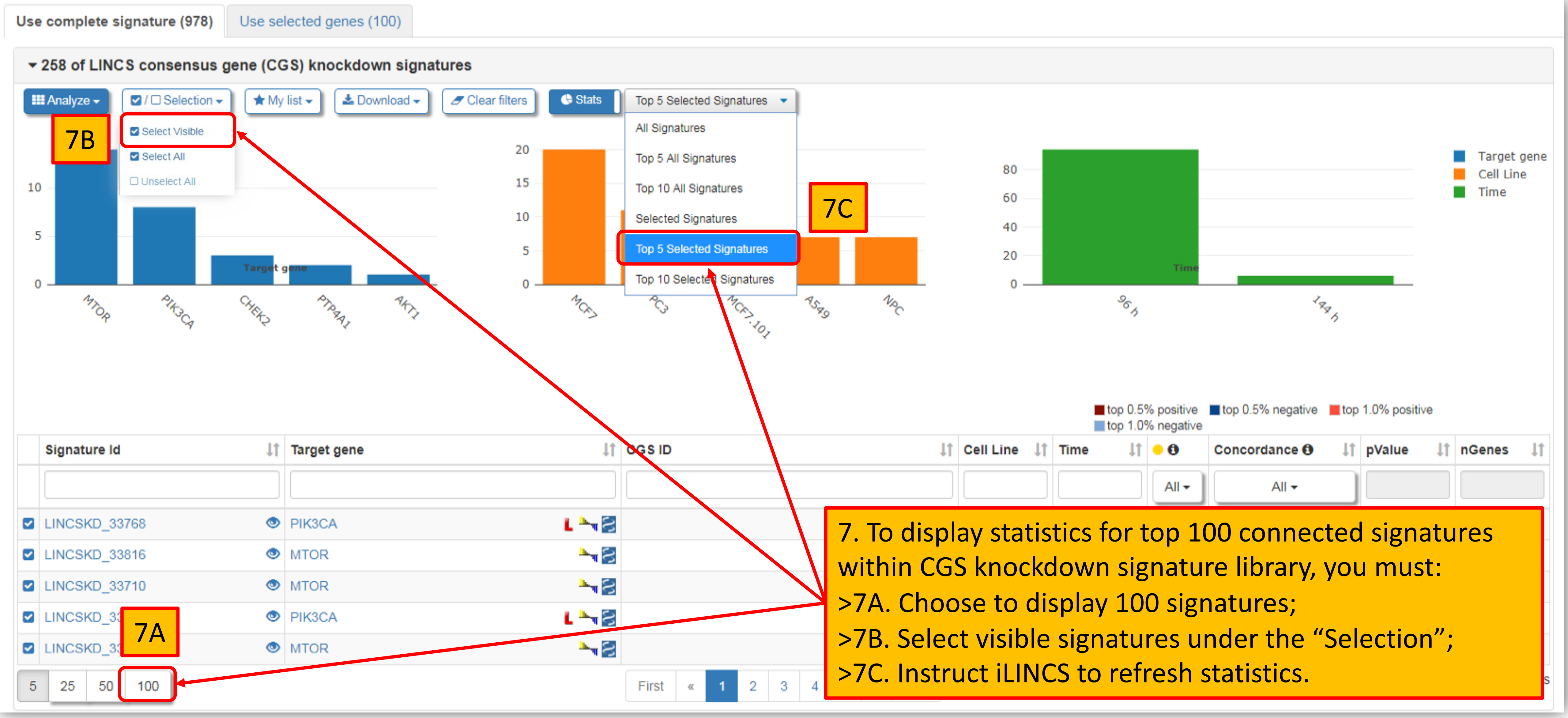
Use complete signature (978)
Use selected genes (100)
258 of LINCS consensus gene (CGS) knockdown signatures
Analyze
Selection
My list
Download
Clear filters
Stats
Top 5 Selected Signatures
Select Visible
Select All
Unselect All
All Signatures
Top 5 All Signatures
Top 10 All Signatures
Selected Signatures
Top 5 Selected Signatures
Top 10 Selected Signatures
7A
7B
7C
Target gene
Cell Line
Time
MTOR
PIK3CA
CHEK2
PTP4A1
AKT1
MCF7
PC3
MCF7.101
A549
NPC
96 h
144 h
top 0.5% positive
top 0.5% negative
top 1.0% positive
top 1.0% negative
Signature Id
Target gene
CGS ID
Cell Line
Time
Concordance
pValue
nGenes
All
LINCSKD_33768
PIK3CA
LINCSKD_33816
MTOR
LINCSKD_33710
MTOR
PIK3CA
MTOR
5
25
50
100
First
«
1
2
3
4
7. To display statistics for top 100 connected signatures within CGS knockdown signature library, you must:
>7A. Choose to display 100 signatures;
>7B. Select visible signatures under the “Selection”;
>7C. Instruct iLINCS to refresh statistics.

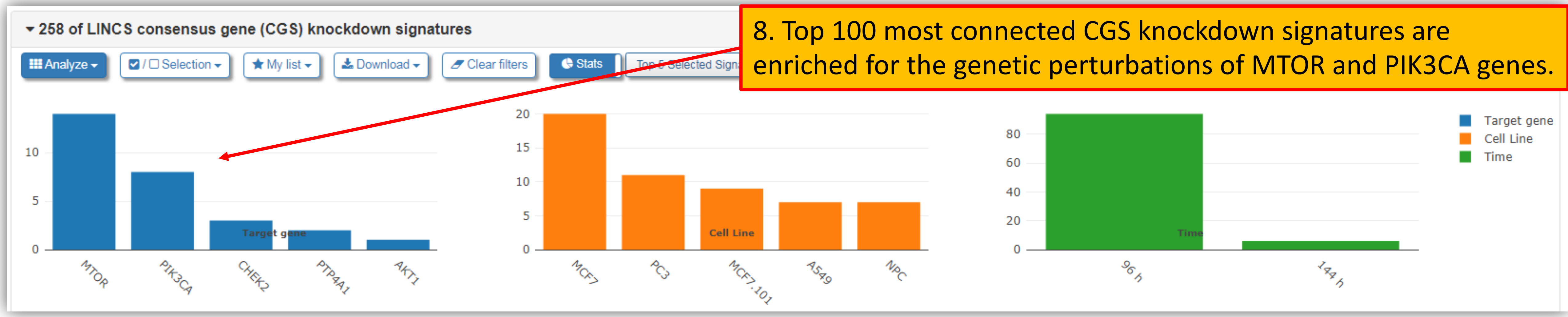

8. Top 100 most connected CGS knockdown signatures are enriched for the genetic perturbations of MTOR and PIK3CA genes.

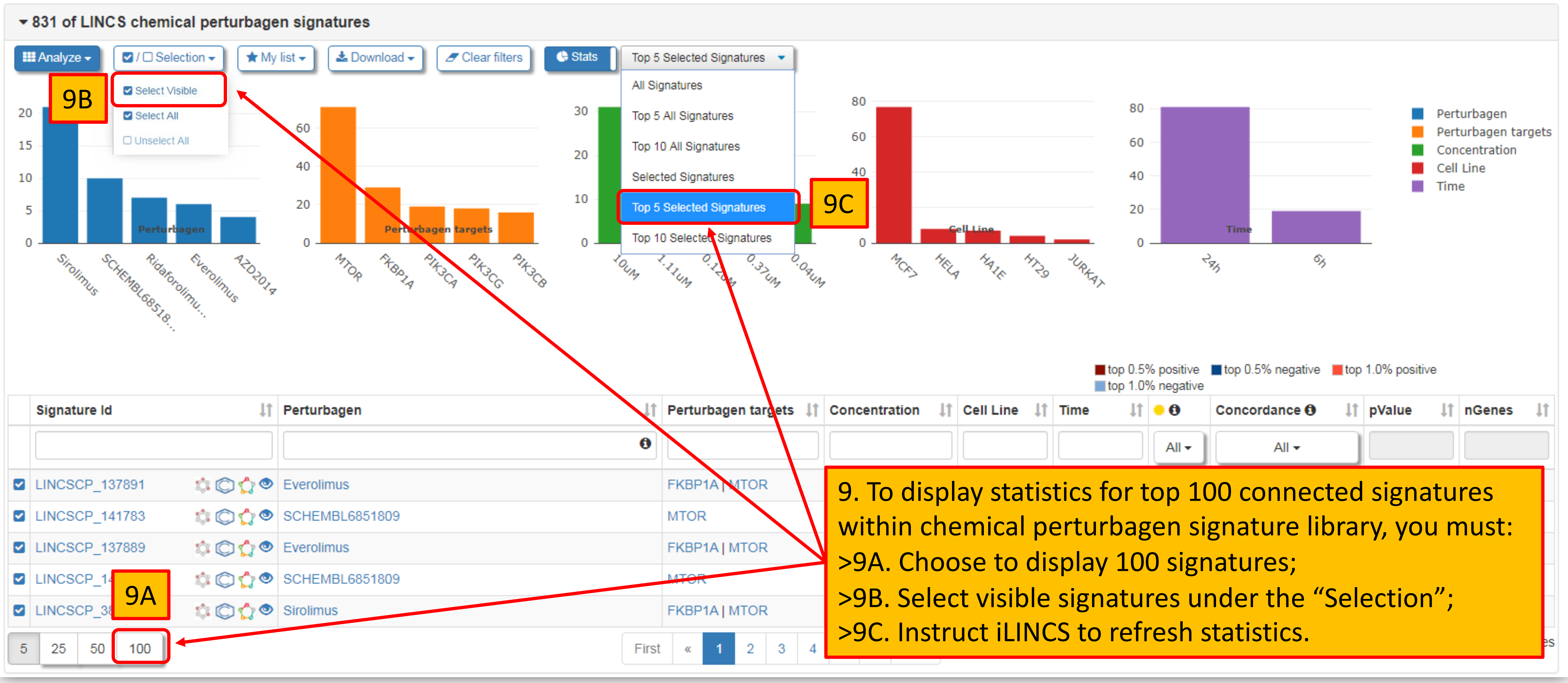
831 of LINCS chemical perturbagen signatures
Analyze
Selection
My list
Download
Clear filters
Stats
Top 5 Selected Signatures
Select Visible
Select All
Unselect All
All Signatures
Top 5 All Signatures
Top 10 All Signatures
Selected Signatures
Top 5 Selected Signatures
Top 10 Selected Signatures
9A
9B
9C
Perturbagen
Perturbagen targets
Concentration
Cell Line
Time
Sirolimus
SCHEMBL68518...
Ridaforolimu...
Everolimus
AZD2014
MTOR
FKBP1A
PIK3CA
PIK3CG
PIK3CB
10uM
1.11uM
0.37uM
0.04uM
MCF7
HELA
HA1E
HT29
JURKAT
24h
6h
top 0.5% positive
top 0.5% negative
top 1.0% positive
top 1.0% negative
Signature Id
Perturbagen
Perturbagen targets
Concentration
Cell Line
Time
Concordance
pValue
nGenes
LINCSCP_137891
Everolimus
FKBP1A | MTOR
LINCSCP_141783
SCHEMBL6851809
MTOR
LINCSCP_137889
Everolimus
FKBP1A | MTOR
SCHEMBL6851809
MTOR
Sirolimus
FKBP1A | MTOR
5
25
50
100
First
«
1
2
3
4
9. To display statistics for top 100 connected signatures within chemical perturbagen signature library, you must:
>9A. Choose to display 100 signatures;
>9B. Select visible signatures under the “Selection”;
>9C. Instruct iLINCS to refresh statistics.

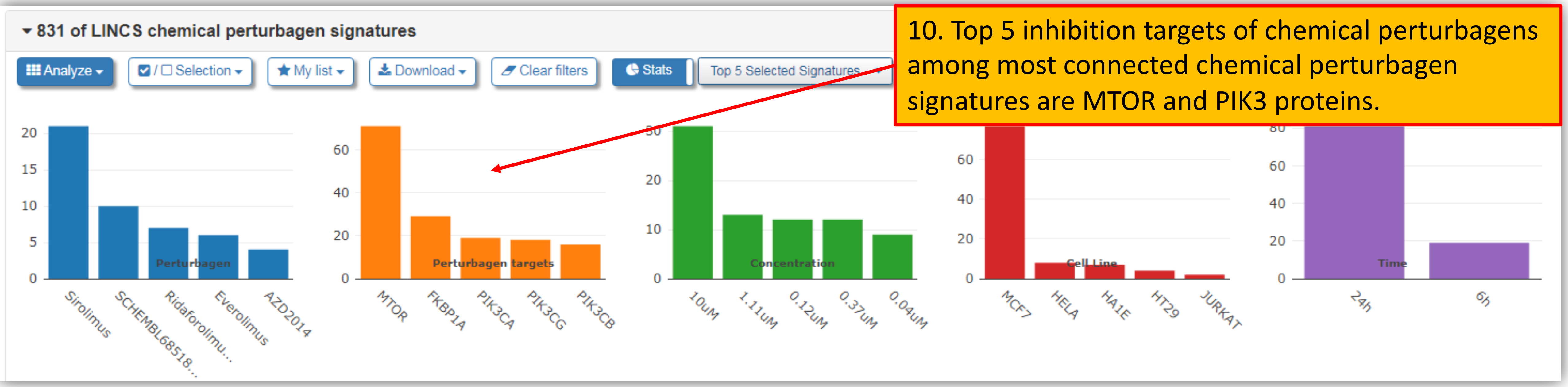

831 of LINCS chemical perturbagen signatures
Analyze
/ Selection
My list
Download
Clear filters
Stats
Top 5 Selected Signatures
10. Top 5 inhibition targets of chemical perturbagens among most connected chemical perturbagen signatures are MTOR and PIK3 proteins.
Perturbagen
Sirolimus
SCHEMBL68518...
Ridaforolimu...
Everolimus
AZD2014
Perturbagen targets
MTOR
FKBP1A
PIK3CA
PIK3CG
PIK3CB
Concentration
10uM
1.11uM
0.12uM
0.37uM
0.04uM
Cell Line
MCF7
HELA
HA1E
HT29
JURKAT
Time
24h
6h

Use case 1 Part 2: Mechanism of action analysis via connection to genetic perturbation signatures

Transcriptional signature of the chemical perturbagens often carry only an echo of such effects since the proteins directly targeted by the chemical and associated signaling proteins are not transcriptionally changed. iLINCS offers the solution for this problem by connecting the CP signatures to LINCS CGSes and follow-up systems biology analysis of genes whose CGSes are highly correlated with the CP signature. This is demonstrated by the analysis of one of the CP signatures of the 24 hour, 0.04μM treatment of the MCF-7 cell line with the mTOR inhibitor Everolimus (Fig 2CDE). Traditional pathway enrichment analysis of the transcriptional signatures via iLINCS connection to Enrichr (Fig 2C) fails to identify the mTOR pathway as being affected. In the next step, we first connect the CP signature to LINCS CGSes and then perform pathway enrichment analysis of genes with correlated CGSes. This analysis correctly identifies mTOR signaling pathway as the top most affected pathway (Fig 2D). Similarly, connectivity analysis with other CP signatures followed by the enrichment analysis of protein targets of connected CPs again identifies the Pi3k-Akt signaling pathway as one of the most enriched (Fig 2E).

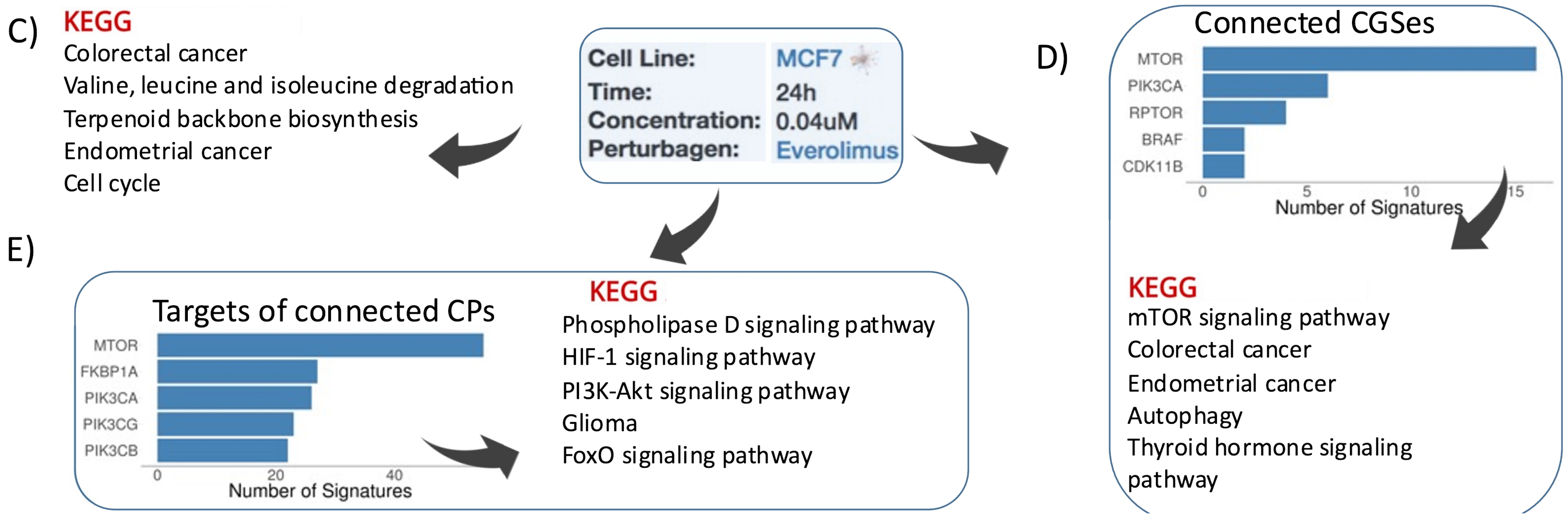

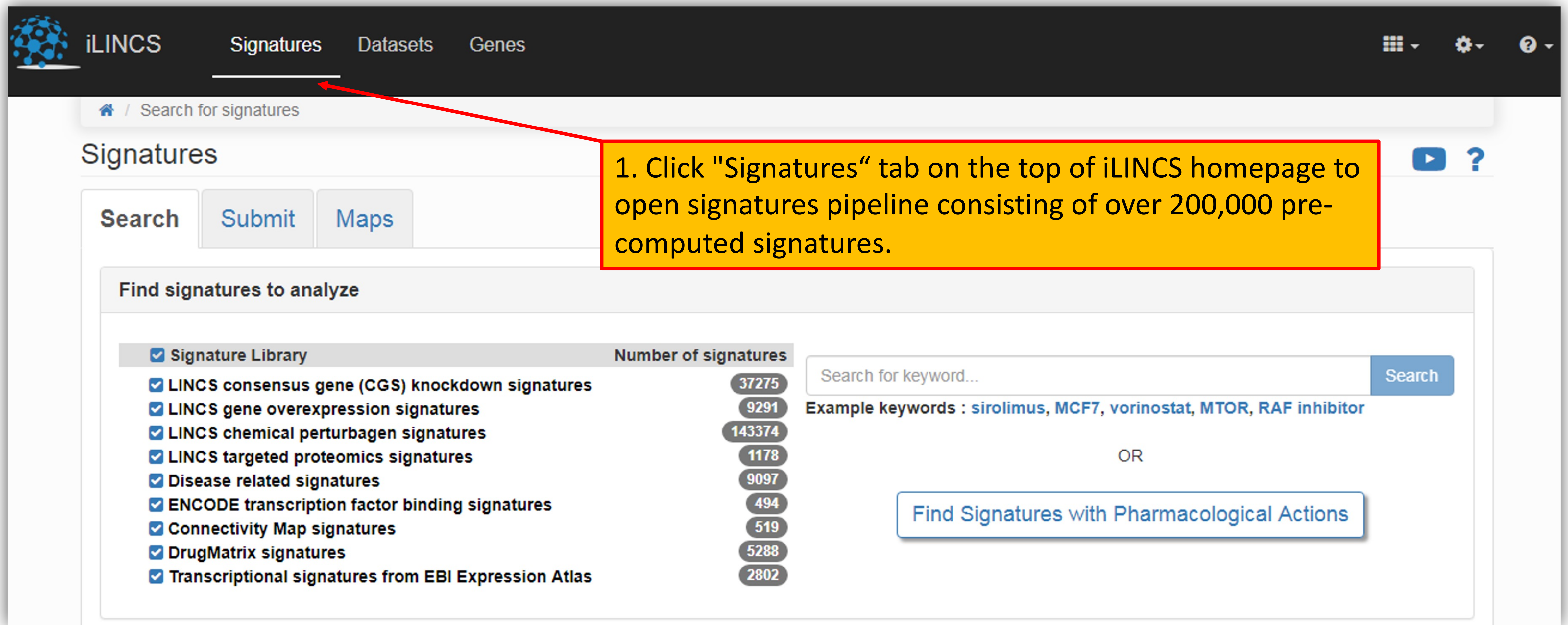
iLINCS
Signatures
Datasets
Genes
Search for signatures
Signatures
Search
Submit
Maps
1. Click "Signatures“ tab on the top of iLINCS homepage to open signatures pipeline consisting of over 200,000 pre-computed signatures.
Find signatures to analyze
Signature Library
Number of signatures
LINCS consensus gene (CGS) knockdown signatures 37275
LINCS gene overexpression signatures 9291
LINCS chemical perturbagen signatures 143374
LINCS targeted proteomics signatures 1178
Disease related signatures 9097
ENCODE transcription factor binding signatures 494
Connectivity Map signatures 519
DrugMatrix signatures 5288
Transcriptional signatures from EBI Expression Atlas 2802
Search for keyword...
Search
Example keywords : sirolimus, MCF7, vorinostat, MTOR, RAF inhibitor
OR
Find Signatures with Pharmacological Actions

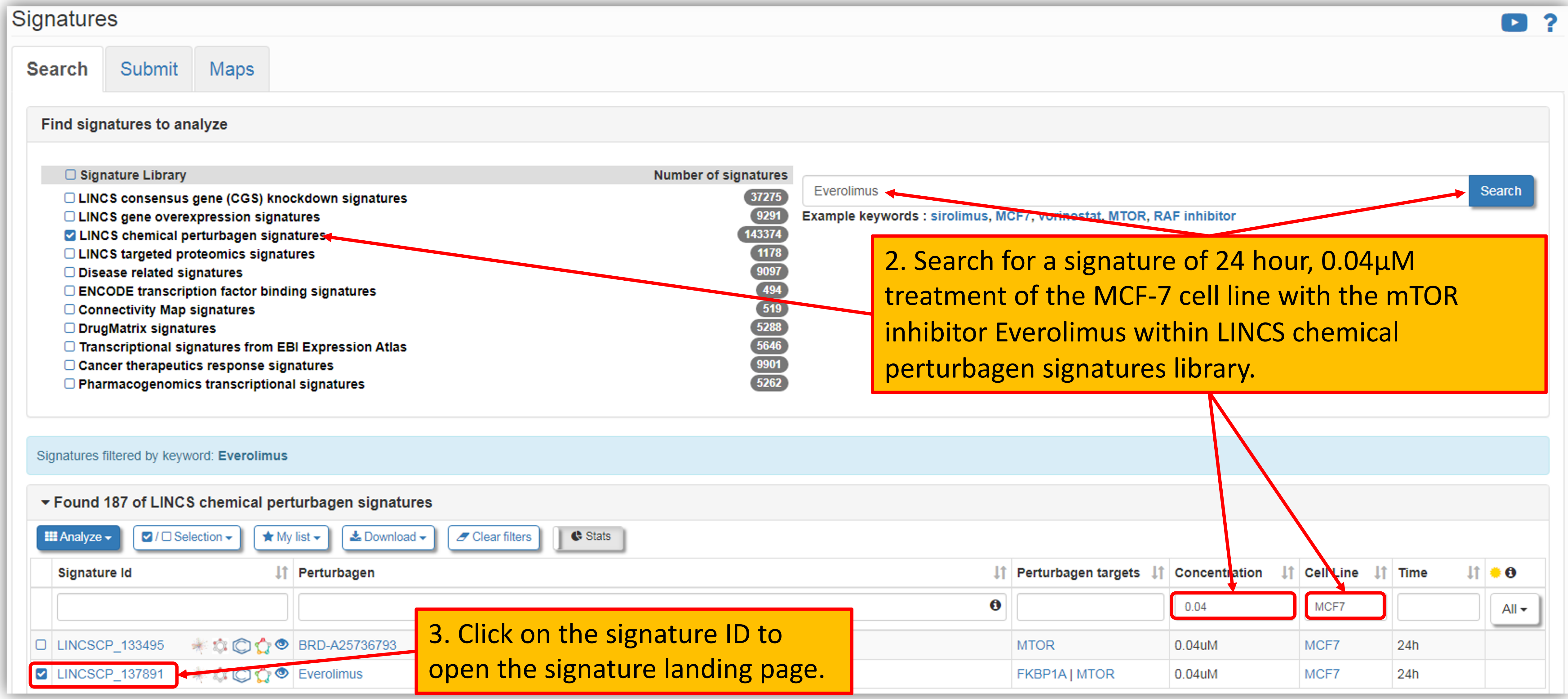
Signatures
Search
Submit
Maps
Find signatures to analyze
Signature Library
Number of signatures
LINCS consensus gene (CGS) knockdown signatures 37275
LINCS gene overexpression signatures 9291
LINCS chemical perturbagen signatures 143374
LINCS targeted proteomics signatures 1178
Disease related signatures 9097
ENCODE transcription factor binding signatures 494
Connectivity Map signatures 519
DrugMatrix signatures 5288
Transcriptional signatures from EBI Expression Atlas 5646
Cancer therapeutics response signatures 9901
Pharmacogenomics transcriptional signatures 5262
Everolimus
Search
Example keywords : sirolimus, MCF7, vorinostat, MTOR, RAF inhibitor
2. Search for a signature of 24 hour, 0.04µM treatment of the MCF-7 cell line with the mTOR inhibitor Everolimus within LINCS chemical perturbagen signatures library.
Signatures filtered by keyword: Everolimus
Found 187 of LINCS chemical perturbagen signatures
Analyze
Selection
My list
Download
Clear filters
Stats
Signature Id
Perturbagen
Perturbagen targets
Concentration
Cell Line
Time
0.04
MCF7
All
LINCSCP_133495
BRD-A25736793
MTOR
0.04uM
MCF7
24h
LINCSCP_137891
Everolimus
FKBP1A | MTOR
0.04uM
MCF7
24h
3. Click on the signature ID to open the signature landing page.

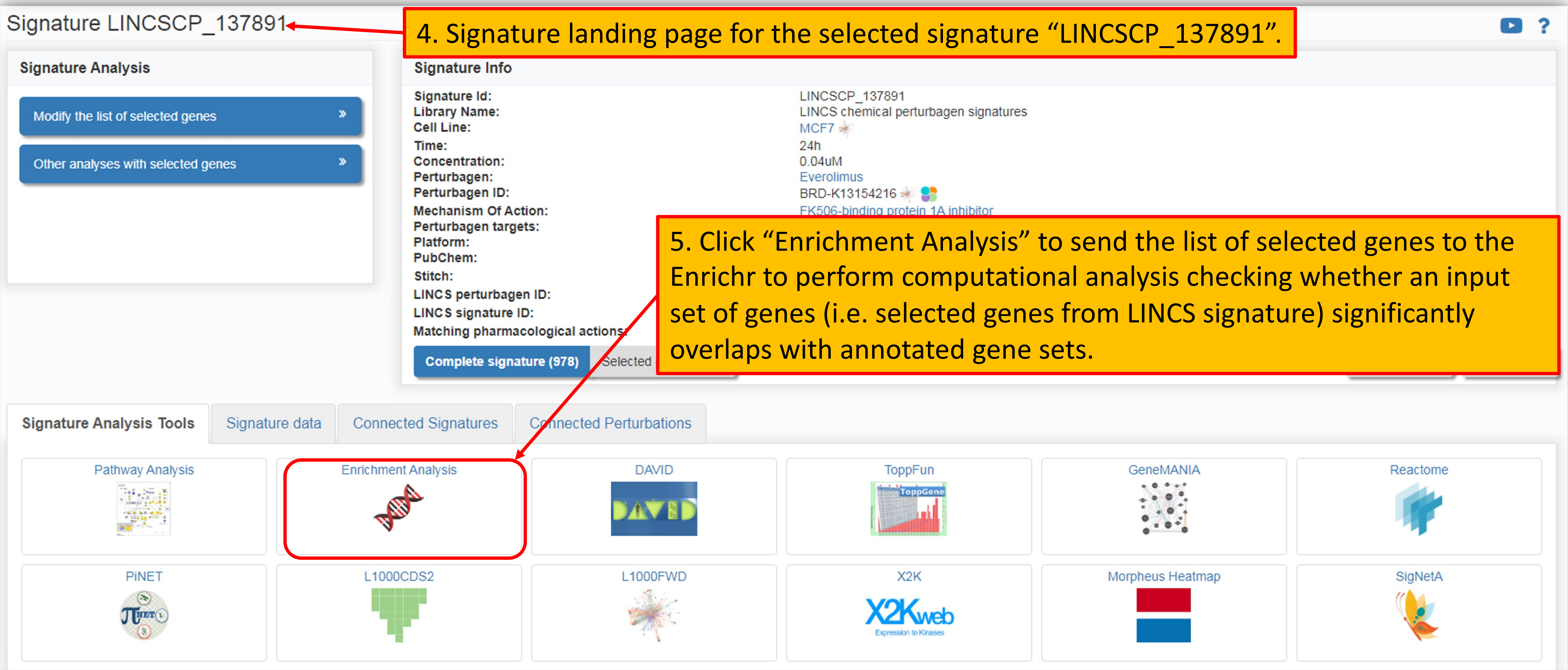
Signature LINCSCP_137891
4. Signature landing page for the selected signature “LINCSCP_137891”.
Signature Analysis
Modify the list of selected genes
Other analyses with selected genes
Signature Info
Signature Id: LINCSCP_137891
Library Name: LINCS chemical perturbagen signatures
Cell Line: MCF7
Time: 24h
Concentration: 0.04uM
Perturbagen: Everolimus
Perturbagen ID: BRD-K13154216
Mechanism Of Action: FK506-binding protein 1A inhibitor
Perturbagen targets:
Platform:
PubChem:
Stitch:
LINCS perturbagen ID:
LINCS signature ID:
Matching pharmacological actions:
Complete signature (978)
Selected
5. Click “Enrichment Analysis” to send the list of selected genes to the Enrichr to perform computational analysis checking whether an input set of genes (i.e. selected genes from LINCS signature) significantly overlaps with annotated gene sets.
Signature Analysis Tools
Signature data
Connected Signatures
Connected Perturbations
Pathway Analysis
Enrichment Analysis
DAVID
ToppFun
ToppGene
GeneMANIA
Reactome
PiNET
L1000CDS2
L1000FWD
X2K
X2Kweb
Expression to Kinases
Morpheus Heatmap
SigNetA

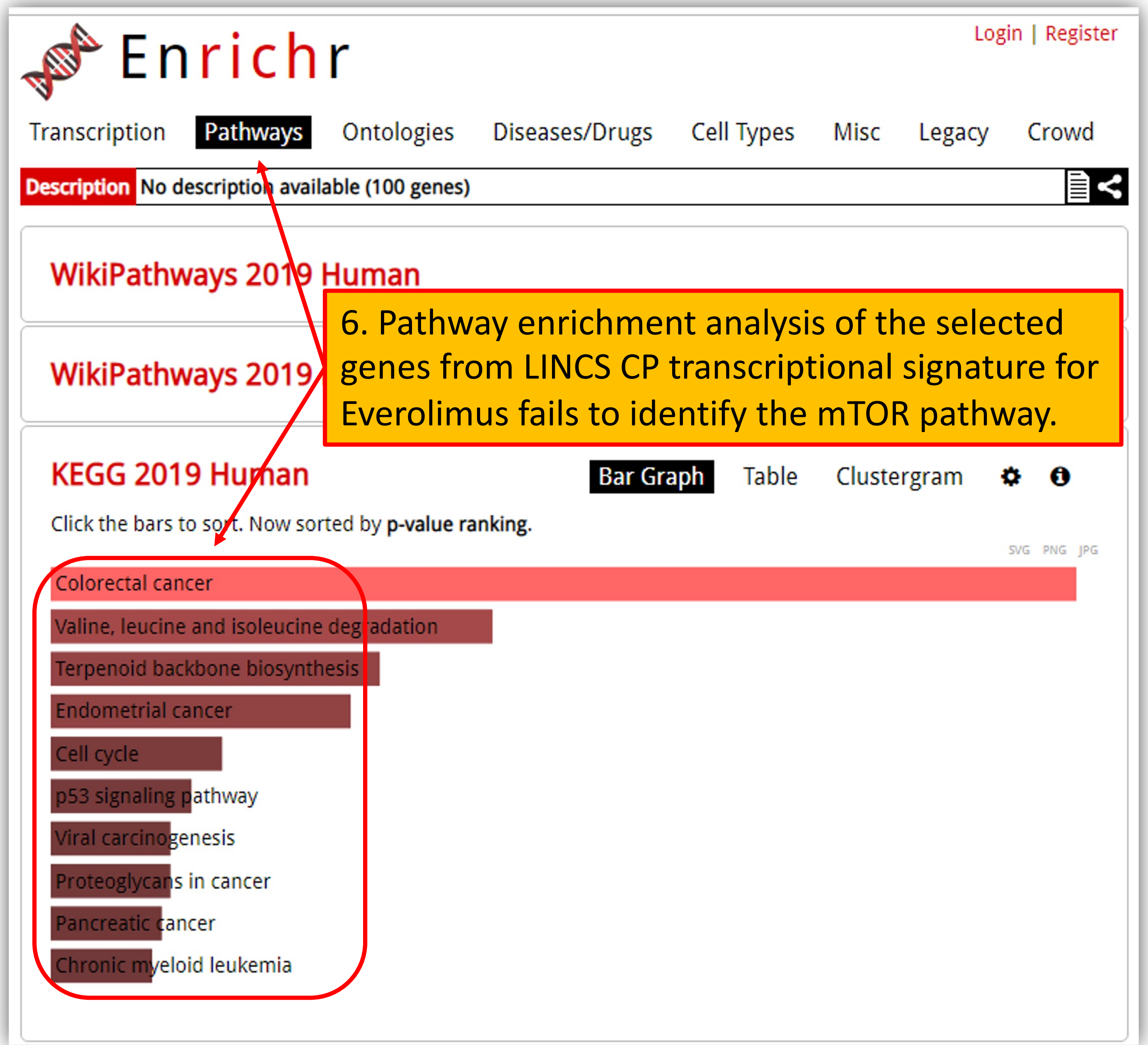
Enrichr
Login | Register
Transcription
Pathways
Ontologies
Diseases/Drugs
Cell Types
Misc
Legacy
Crowd
Description
No description available (100 genes)
WikiPathways 2019 Human
WikiPathways 2019
6. Pathway enrichment analysis of the selected genes from LINCS CP transcriptional signature for Everolimus fails to identify the mTOR pathway.
KEGG 2019 Human
Bar Graph
Table
Clustergram
Click the bars to sort. Now sorted by p-value ranking.
SVG
PNG
JPG
Colorectal cancer
Valine, leucine and isoleucine degradation
Terpenoid backbone biosynthesis
Endometrial cancer
Cell cycle
p53 signaling pathway
Viral carcinogenesis
Proteoglycans in cancer
Pancreatic cancer
Chronic myeloid leukemia

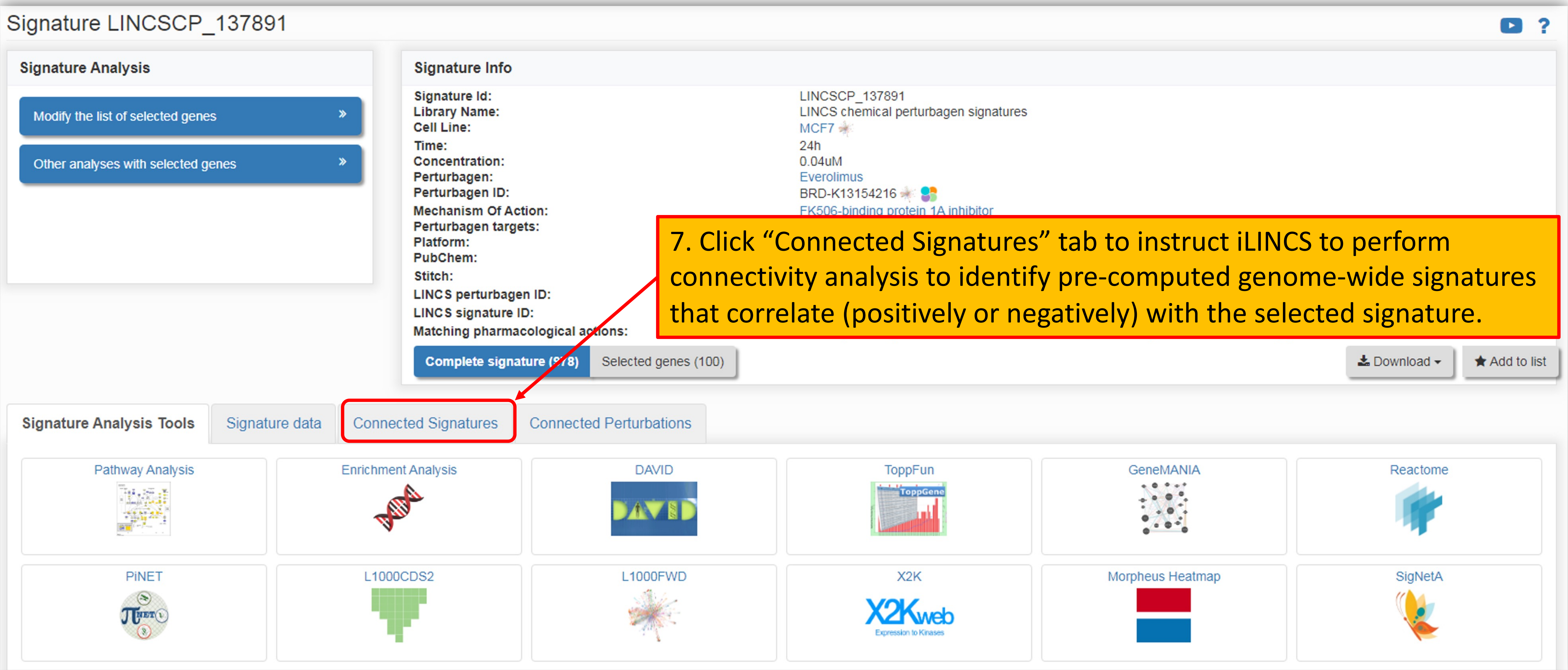
Signature LINCSCP_137891
Signature Analysis
Modify the list of selected genes
Other analyses with selected genes
Signature Info
Signature Id: LINCSCP_137891
Library Name: LINCS chemical perturbagen signatures
Cell Line: MCF7
Time: 24h
Concentration: 0.04uM
Perturbagen: Everolimus
Perturbagen ID: BRD-K13154216
Mechanism Of Action: FK506-binding protein 1A inhibitor
Perturbagen targets:
Platform:
PubChem:
Stitch:
LINCS perturbagen ID:
LINCS signature ID:
Matching pharmacological actions:
7. Click “Connected Signatures” tab to instruct iLINCS to perform connectivity analysis to identify pre-computed genome-wide signatures that correlate (positively or negatively) with the selected signature.
Complete signature (978)
Selected genes (100)
Download
Add to list
Signature Analysis Tools
Signature data
Connected Signatures
Connected Perturbations
Pathway Analysis
Enrichment Analysis
DAVID
ToppFun
GeneMANIA
Reactome
PiNET
L1000CDS2
L1000FWD
X2K
Morpheus Heatmap
SigNetA
X2Kweb
Expression to Kinases

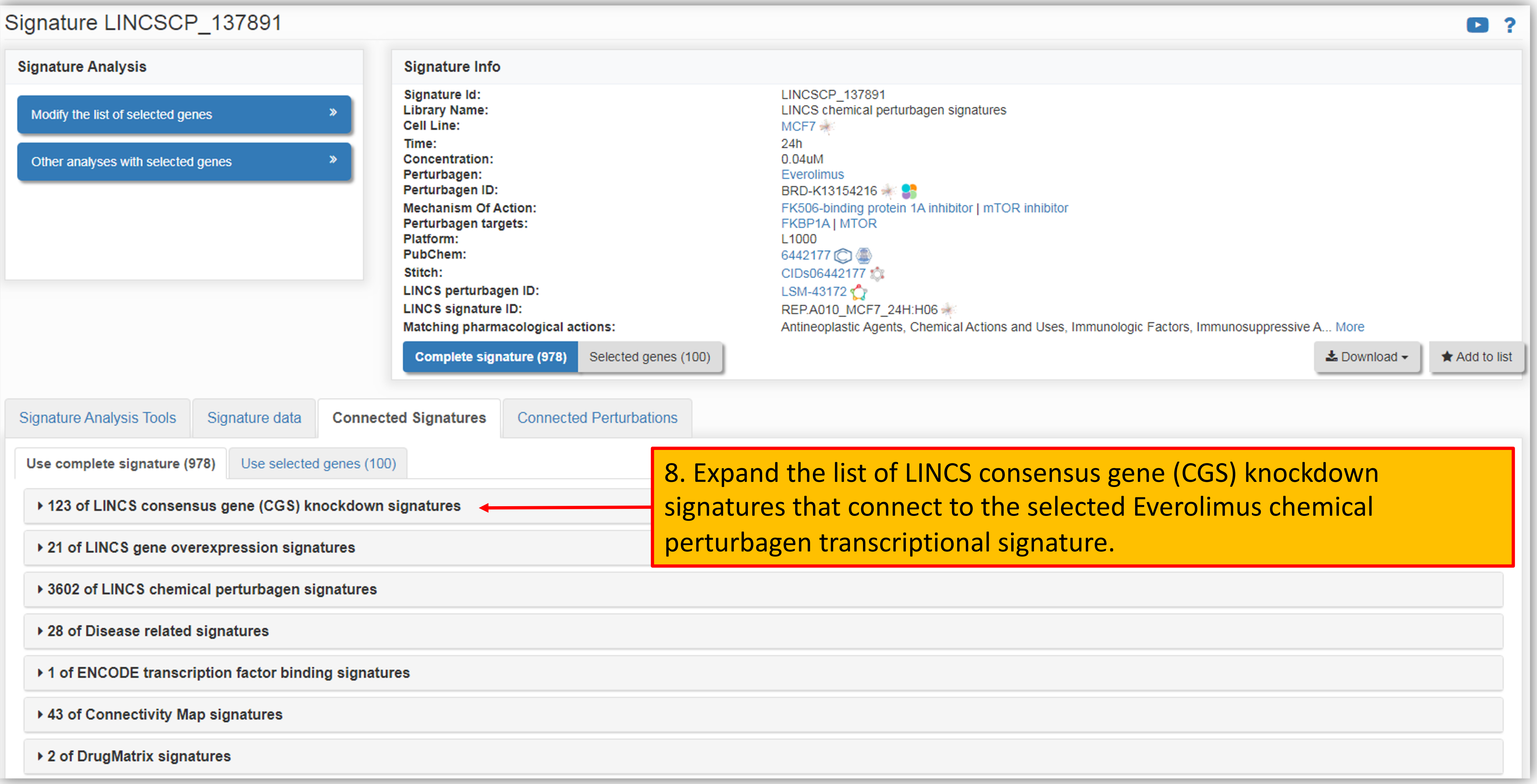
Signature LINCSCP_137891
Signature Analysis
Modify the list of selected genes
Other analyses with selected genes
Signature Info
Signature Id: LINCSCP_137891
Library Name: LINCS chemical perturbagen signatures
Cell Line: MCF7
Time: 24h
Concentration: 0.04uM
Perturbagen: Everolimus
Perturbagen ID: BRD-K13154216
Mechanism Of Action: FK506-binding protein 1A inhibitor | mTOR inhibitor
Perturbagen targets: FKBP1A | MTOR
Platform: L1000
PubChem: 6442177
Stitch: CIDs06442177
LINCS perturbagen ID: LSM-43172
LINCS signature ID: REP.A010_MCF7_24H:H06
Matching pharmacological actions: Antineoplastic Agents, Chemical Actions and Uses, Immunologic Factors, Immunosuppressive A... More
Complete signature (978)
Selected genes (100)
Download
Add to list
Signature Analysis Tools
Signature data
Connected Signatures
Connected Perturbations
Use complete signature (978)
Use selected genes (100)
123 of LINCS consensus gene (CGS) knockdown signatures
21 of LINCS gene overexpression signatures
3602 of LINCS chemical perturbagen signatures
28 of Disease related signatures
1 of ENCODE transcription factor binding signatures
43 of Connectivity Map signatures
2 of DrugMatrix signatures
8. Expand the list of LINCS consensus gene (CGS) knockdown signatures that connect to the selected Everolimus chemical perturbagen transcriptional signature.

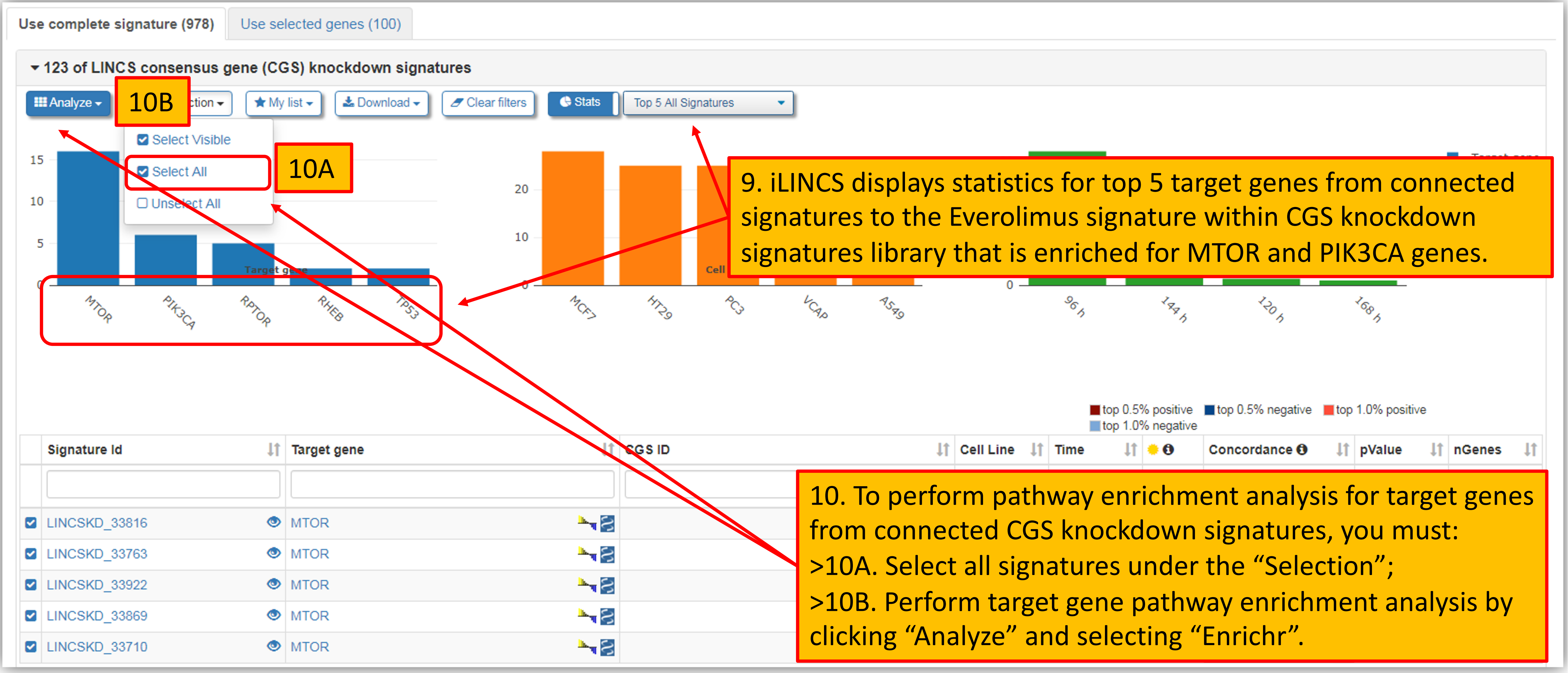

Use complete signature (978)
Use selected genes (100)
123 of LINCS consensus gene (CGS) knockdown signatures
Analyze
10B
My list
Download
Clear filters
Stats
Top 5 All Signatures
Select Visible
Select All
Unselect All
10A
9. iLINCS displays statistics for top 5 target genes from connected signatures to the Everolimus signature within CGS knockdown signatures library that is enriched for MTOR and PIK3CA genes.
MTOR
PIK3CA
RPTOR
RHEB
TP53
MCF7
HT29
PC3
VCAP
A549
96 h
144 h
120 h
168 h
top 0.5% positive
top 0.5% negative
top 1.0% positive
top 1.0% negative
Signature Id
Target gene
CGS ID
Cell Line
Time
Concordance
pValue
nGenes
LINCSKD_33816
LINCSKD_33763
LINCSKD_33922
LINCSKD_33869
LINCSKD_33710
10. To perform pathway enrichment analysis for target genes from connected CGS knockdown signatures, you must:
>10A. Select all signatures under the “Selection”;
>10B. Perform target gene pathway enrichment analysis by clicking “Analyze” and selecting “Enrichr”.

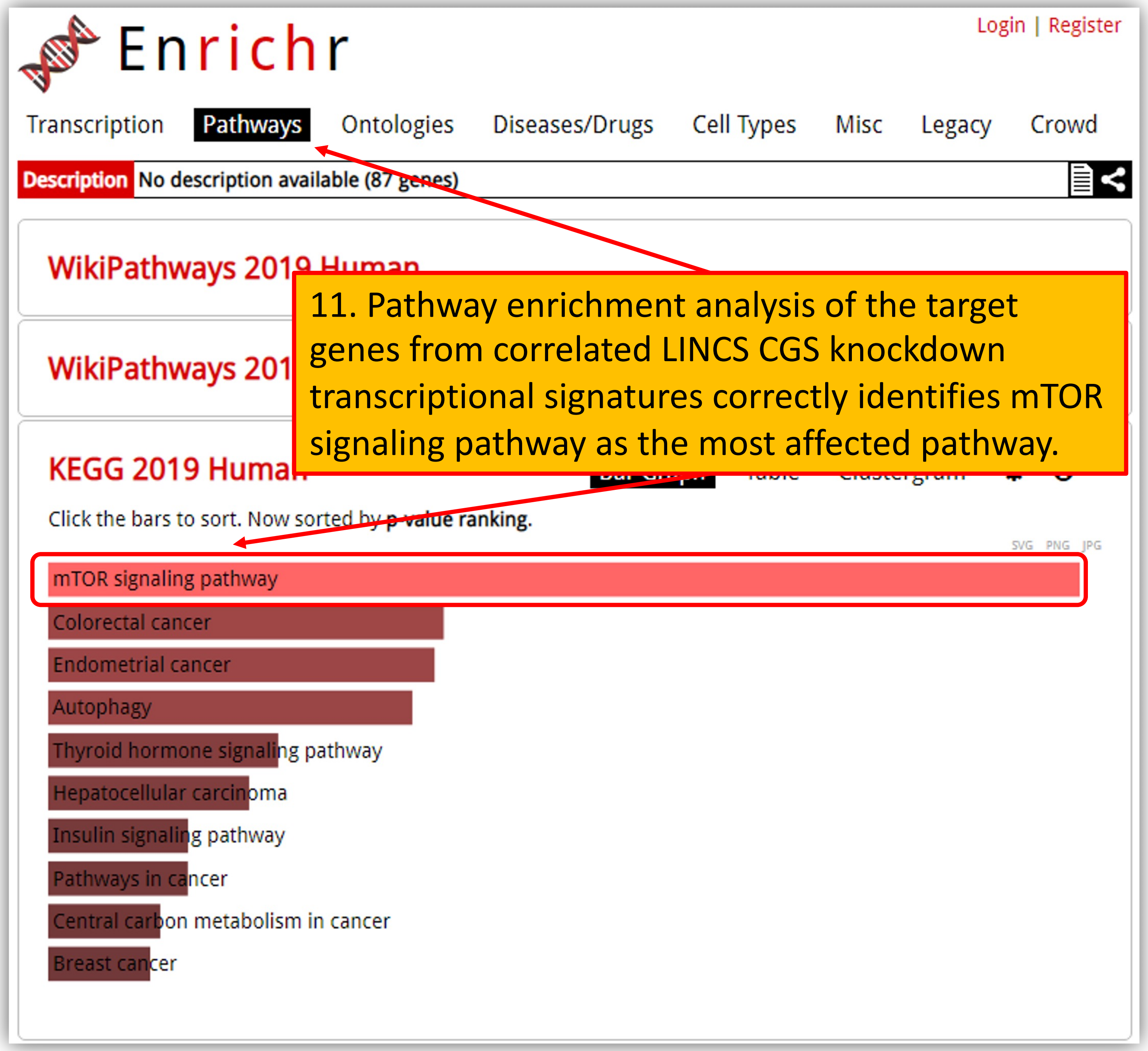

11. Pathway enrichment analysis of the target genes from correlated LINCS CGS knockdown transcriptional signatures correctly identifies mTOR signaling pathway as the most affected pathway.

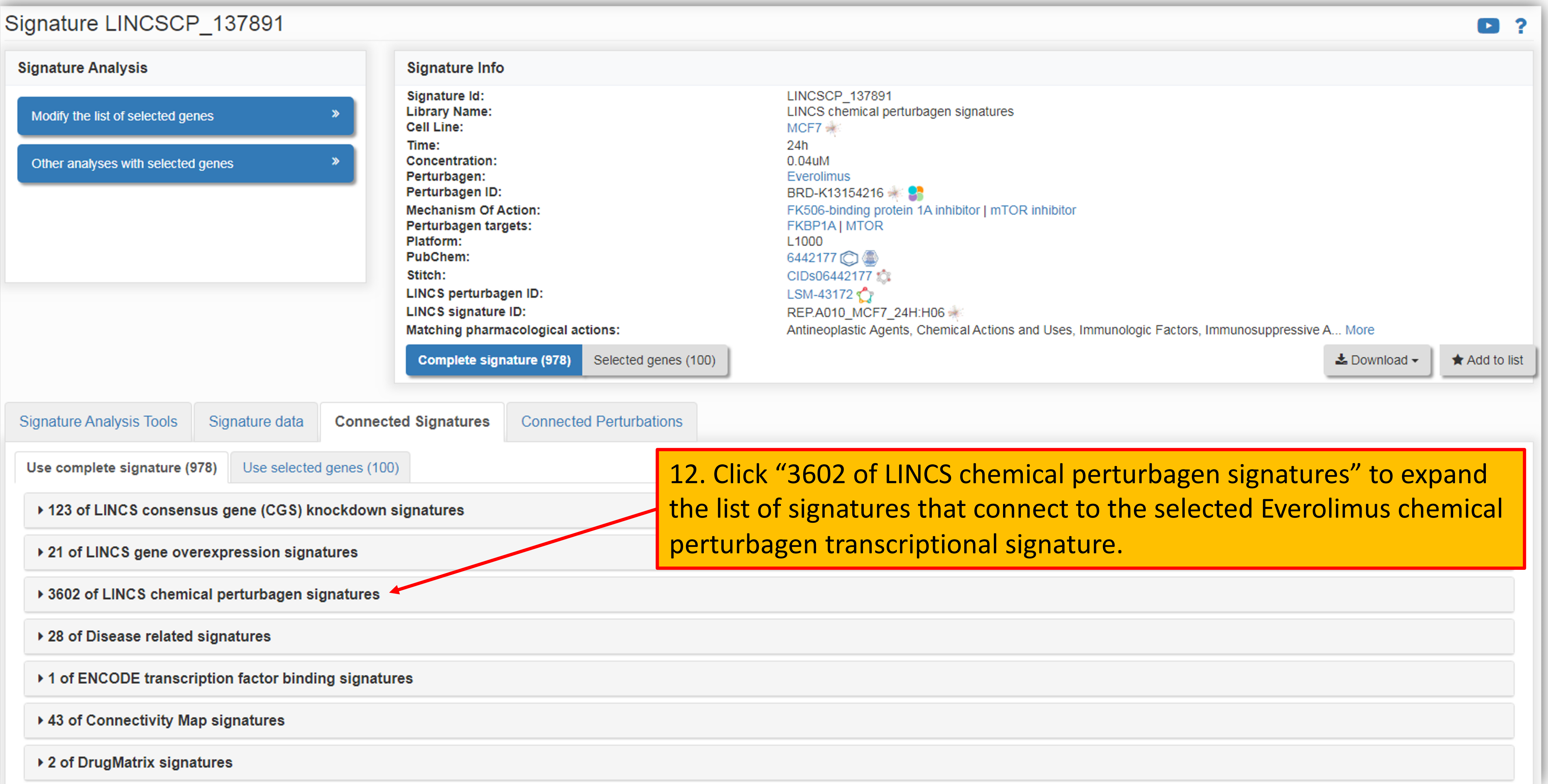

12. Click "3602 of LINCS chemical perturbagen signatures" to expand the list of signatures that connect to the selected Everolimus chemical perturbagen transcriptional signature.

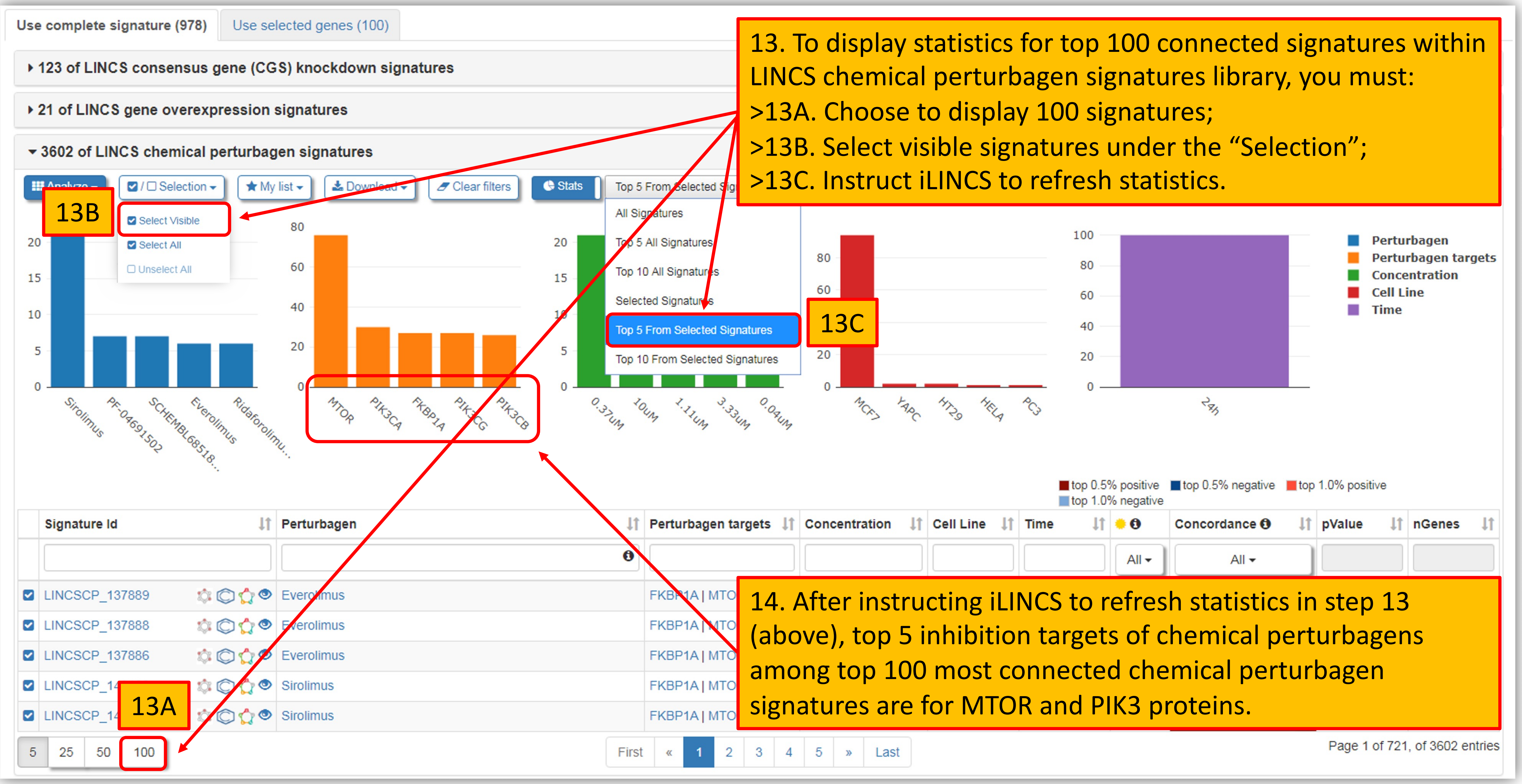

Use complete signature (978)
Use selected genes (100)
123 of LINCS consensus gene (CGS) knockdown signatures
21 of LINCS gene overexpression signatures
3602 of LINCS chemical perturbagen signatures
Selection
My list
Download
Clear filters
Stats
Select Visible
Select All
Unselect All
All Signatures
Top 5 All Signatures
Top 10 All Signatures
Selected Signatures
Top 5 From Selected Signatures
Top 10 From Selected Signatures
13. To display statistics for top 100 connected signatures within LINCS chemical perturbagen signatures library, you must:
>13A. Choose to display 100 signatures;
>13B. Select visible signatures under the "Selection";
>13C. Instruct iLINCS to refresh statistics.
13A
13B
13C
Sirolimus
PF-04691502
Everolimus
MTOR
PIK3CA
FKBP1A
PIK3CB
0.37uM
10uM
1.11uM
3.33uM
0.04uM
MCF7
YAPC
HT29
HELA
PC3
24h
Perturbagen
Perturbagen targets
Concentration
Cell Line
Time
top 0.5% positive
top 0.5% negative
top 1.0% positive
top 1.0% negative
Signature Id
Perturbagen
Perturbagen targets
Concentration
Cell Line
Time
Concordance
pValue
nGenes
LINCSCP_137889
LINCSCP_137888
LINCSCP_137886
Everolimus
Sirolimus
14. After instructing iLINCS to refresh statistics in step 13 (above), top 5 inhibition targets of chemical perturbagens among top 100 most connected chemical perturbagen signatures are for MTOR and PIK3 proteins.
First
Last
Page 1 of 721, of 3602 entries

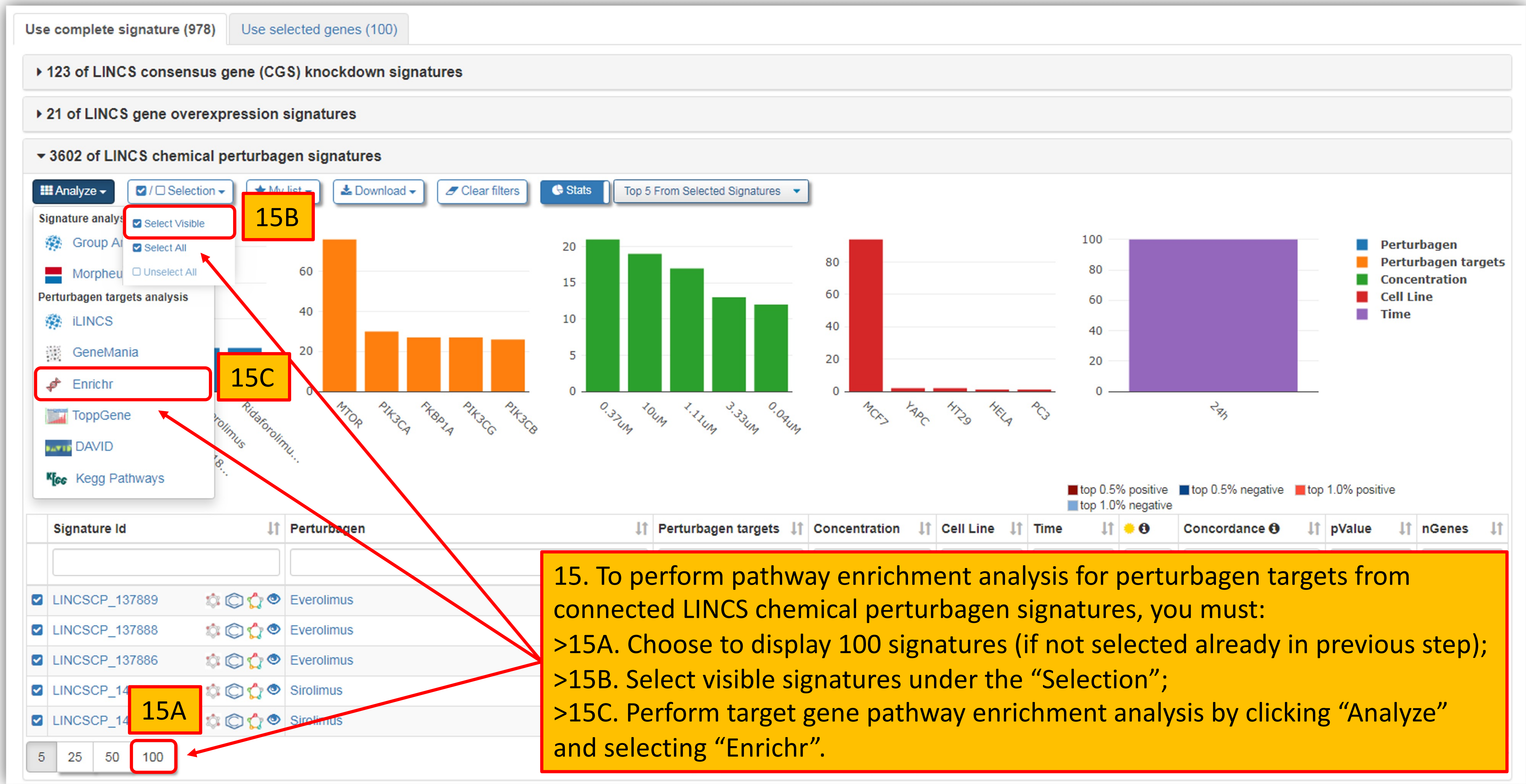

15. To perform pathway enrichment analysis for perturbagen targets from connected LINCS chemical perturbagen signatures, you must:

>15A. Choose to display 100 signatures (if not selected already in previous step);

>15B. Select visible signatures under the “Selection”;

>15C. Perform target gene pathway enrichment analysis by clicking “Analyze” and selecting “Enrichr”.

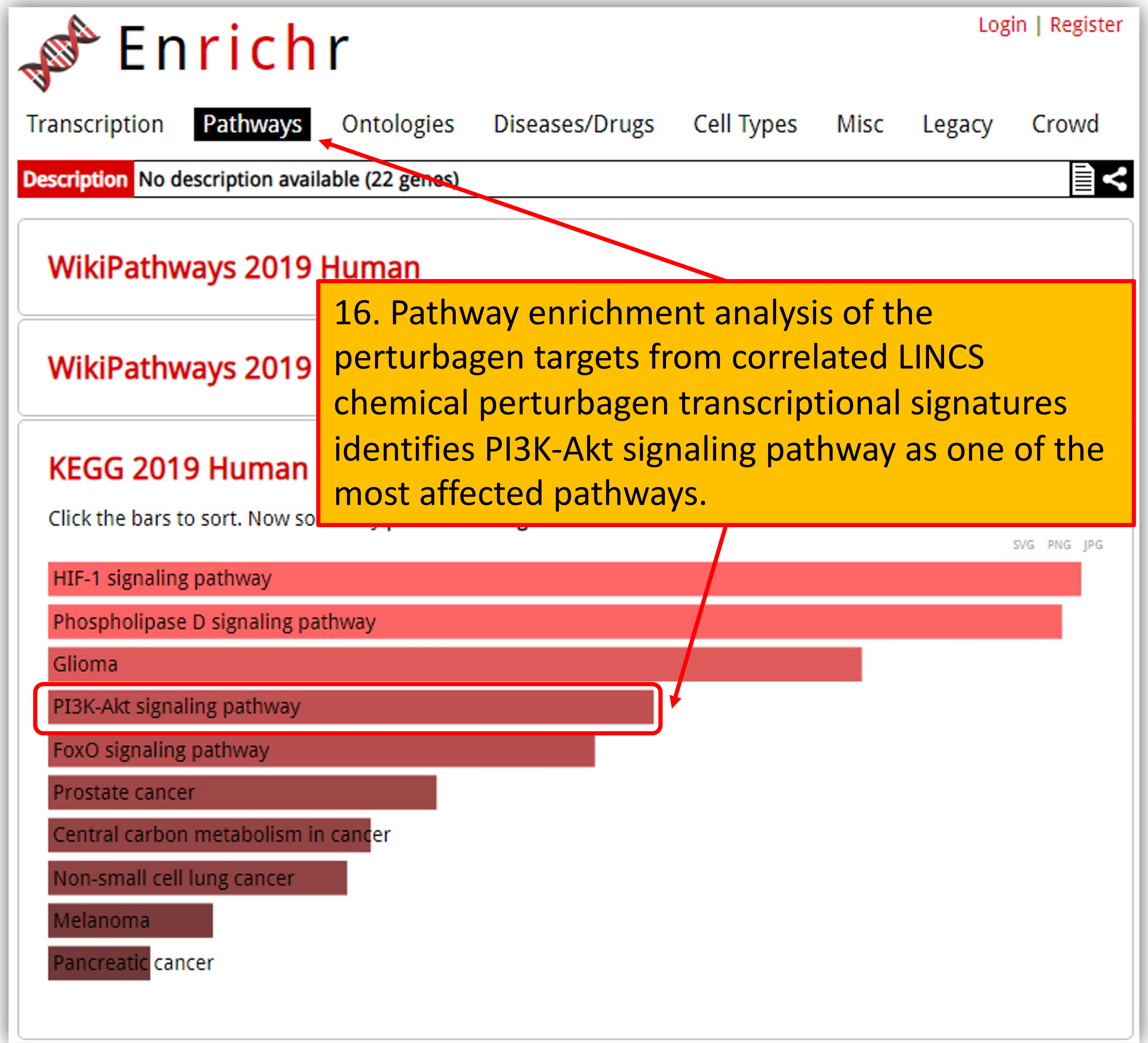
Enrichr
Login | Register
Transcription
Pathways
Ontologies
Diseases/Drugs
Cell Types
Misc
Legacy
Crowd
Description
No description available (22 genes)
WikiPathways 2019 Human
WikiPathways 2019
KEGG 2019 Human
Click the bars to sort. Now so
16. Pathway enrichment analysis of the perturbagen targets from correlated LINCS chemical perturbagen transcriptional signatures identifies PI3K-Akt signaling pathway as one of the most affected pathways.
SVG
PNG
JPG
HIF-1 signaling pathway
Phospholipase D signaling pathway
Glioma
PI3K-Akt signaling pathway
FoxO signaling pathway
Prostate cancer
Central carbon metabolism in cancer
Non-small cell lung cancer
Melanoma
Pancreatic cancer

Use case 2: Proteo-genomics analysis of cancer driver events in breast cancer

The use case describes the analysis of TCGA breast cancer RNA-seq and RPPA data using the iLINCS "Datasets" workflow to construct and analyze the differential gene and protein expression signatures contrasting Luminal A and Her2 enriched (Her2E) breast tumors. The analysis results are depicted in the Figure. A) Most differentially expressed proteins in the proteomics signatures constructed by comparing RPPA profiles of Her2E and Luminal A BRC samples; B) Gene expression profile of the genes corresponding to proteins in A) based on RNA-seq data; C) Top 100 CP signatures most connected with the transcriptional signature constructed by comparing RNA-seq profiles of Her2E and Luminal A samples; D) Selected chemical perturbagens and their targets for CP signatures in C).

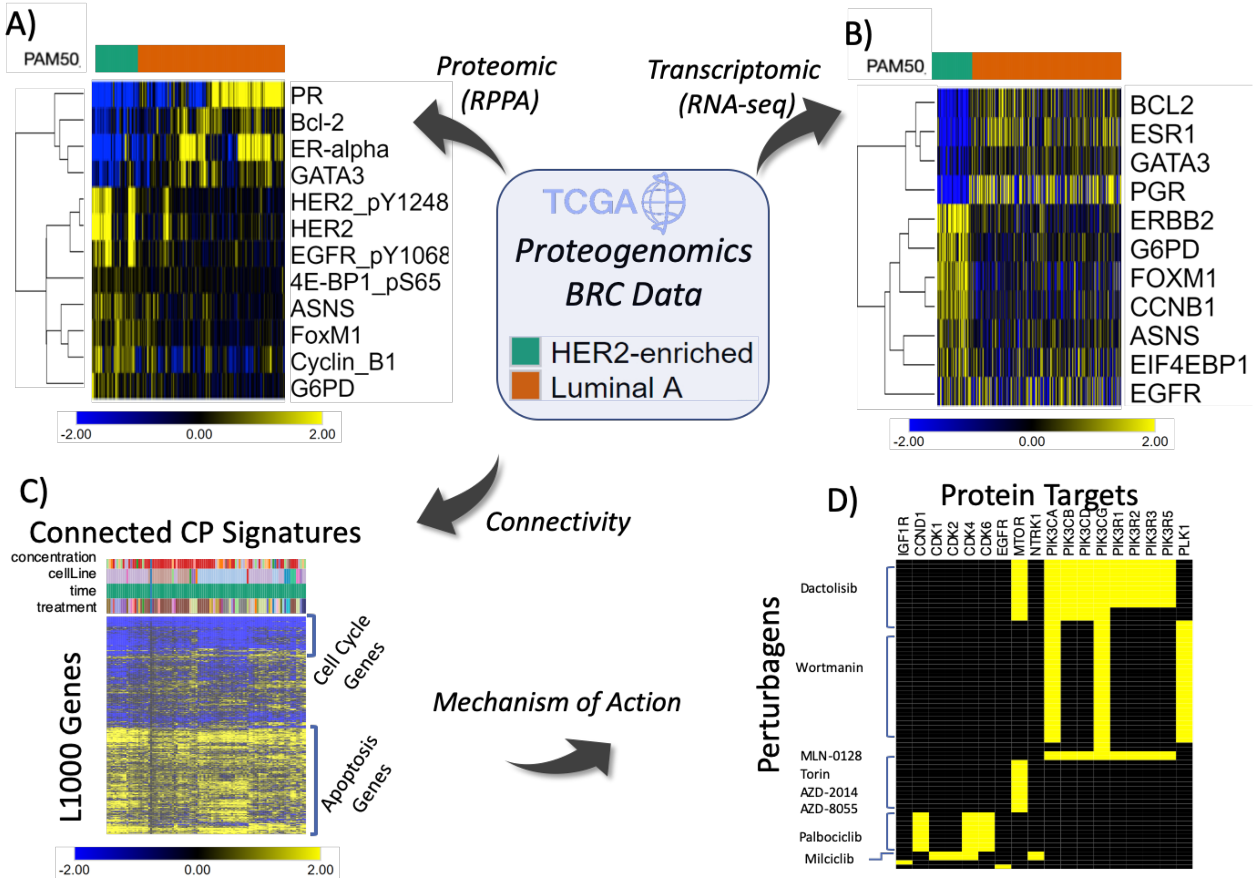

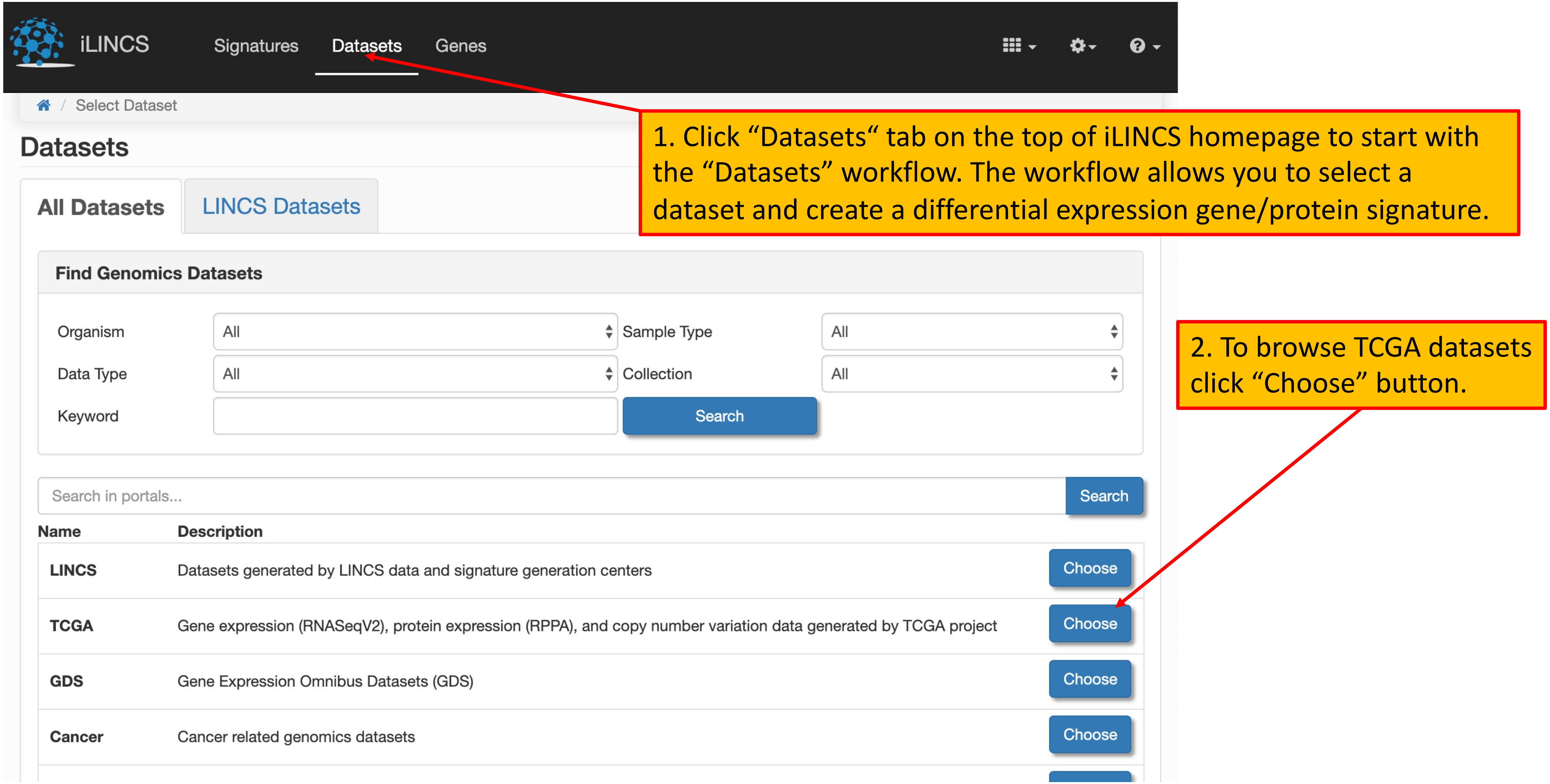
iLINCS
Signatures
Datasets
Genes
Select Dataset
Datasets
All Datasets
LINCS Datasets
Find Genomics Datasets
Organism
All
Sample Type
All
Data Type
All
Collection
All
Keyword
Search
Search in portals...
Search
Name
Description
LINCS
Datasets generated by LINCS data and signature generation centers
Choose
TCGA
Gene expression (RNASeqV2), protein expression (RPPA), and copy number variation data generated by TCGA project
Choose
GDS
Gene Expression Omnibus Datasets (GDS)
Choose
Cancer
Cancer related genomics datasets
Choose
1. Click "Datasets" tab on the top of iLINCS homepage to start with the "Datasets" workflow. The workflow allows you to select a dataset and create a differential expression gene/protein signature.
2. To browse TCGA datasets click "Choose" button.

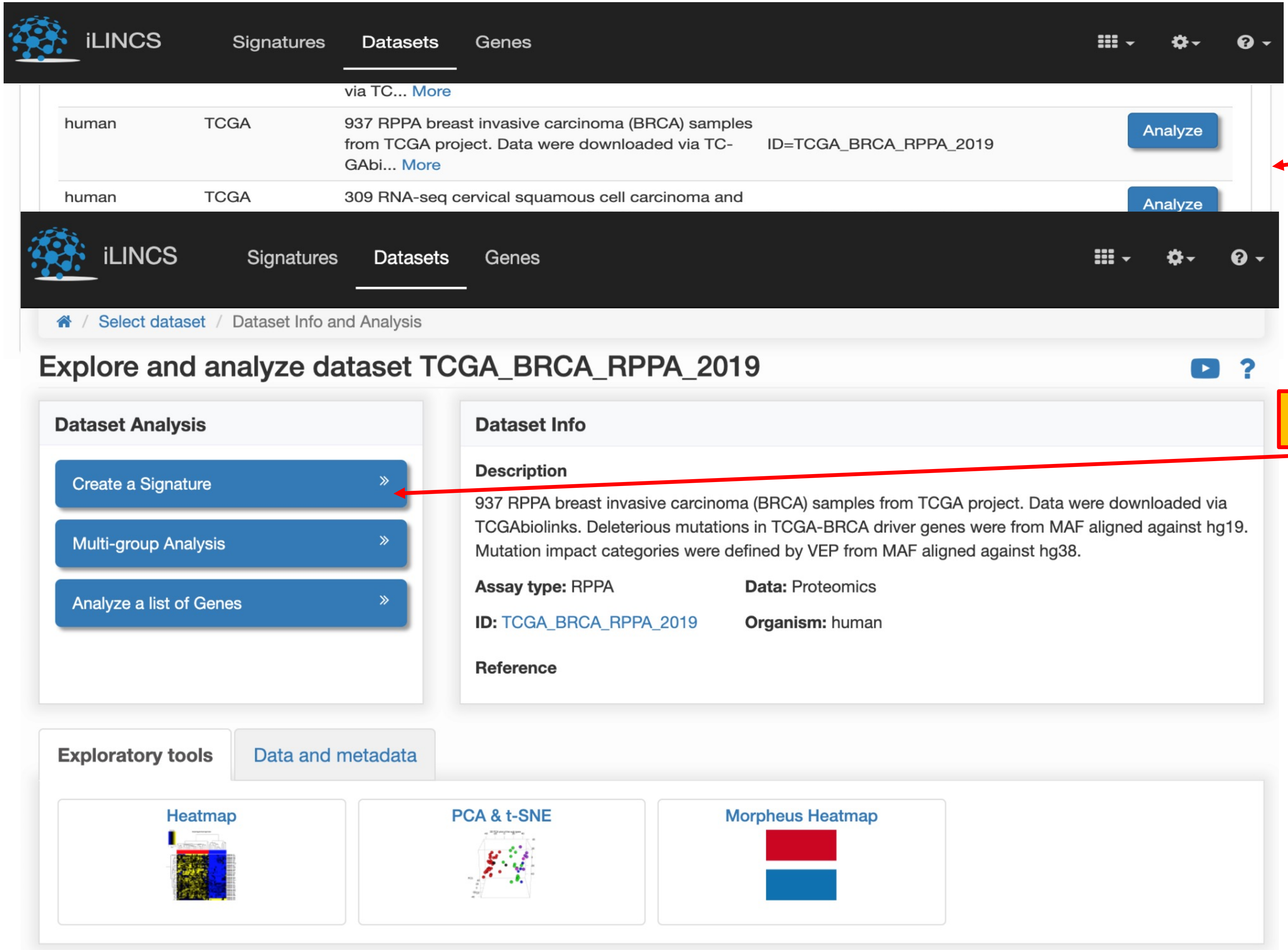

3. Select the BRC RPPA dataset for the analysis.

4. To create signature.

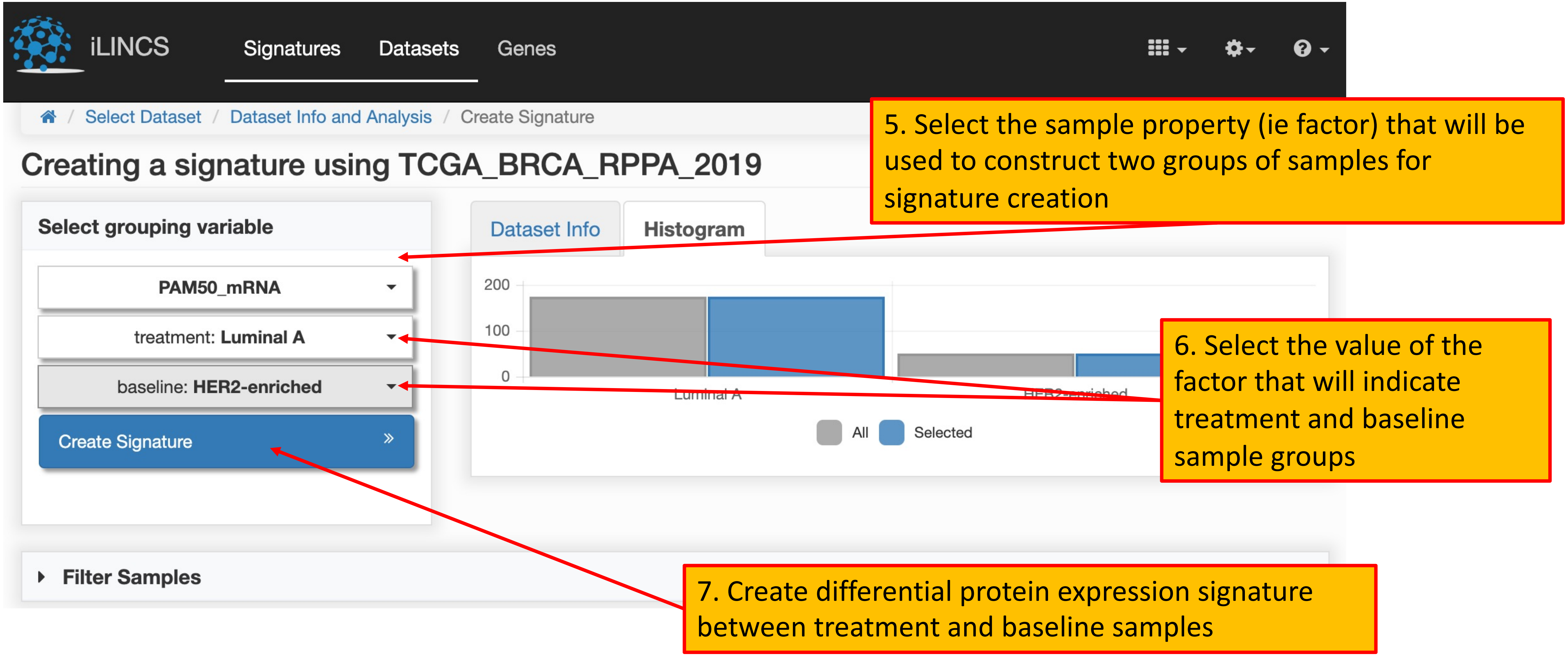
iLINCS
Signatures
Datasets
Genes
Select Dataset / Dataset Info and Analysis / Create Signature
Creating a signature using TCGA_BRCA_RPPA_2019
Select grouping variable
PAM50_mRNA
treatment: Luminal A
baseline: HER2-enriched
Create Signature
Dataset Info
Histogram
200
100
0
Luminal A
All
Selected
Filter Samples
5. Select the sample property (ie factor) that will be used to construct two groups of samples for signature creation
6. Select the value of the factor that will indicate treatment and baseline sample groups
7. Create differential protein expression signature between treatment and baseline samples

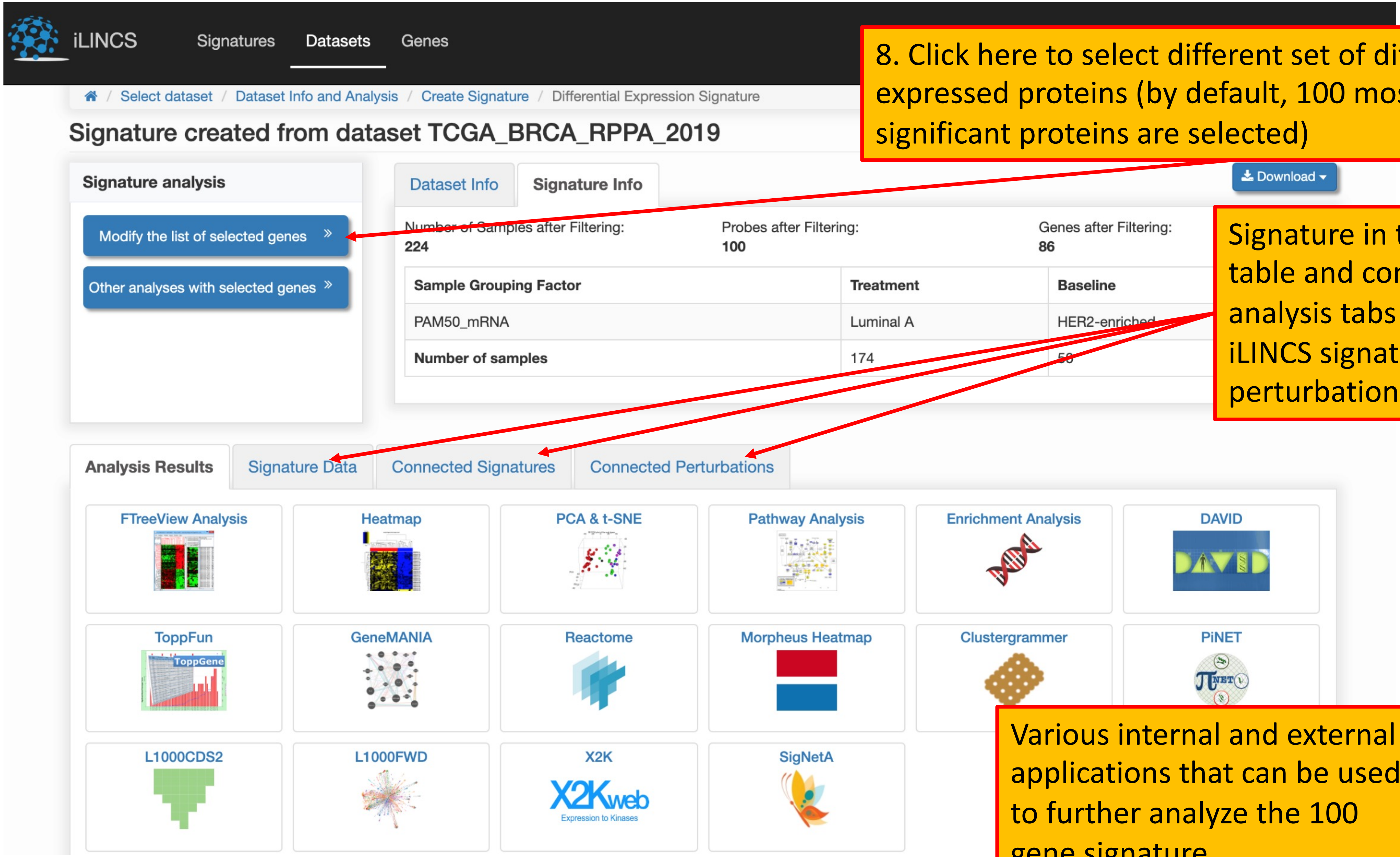

8. Click here to select different set of differentially expressed proteins (by default, 100 most statistically significant proteins are selected)

Signature in the form of the table and connectivity analysis tabs to connect to iLINCS signatures and perturbations

Various internal and external applications that can be used to further analyze the 100 gene signature

Use Case 3: Proteo-genomics analysis of cancer driver events in breast cancer

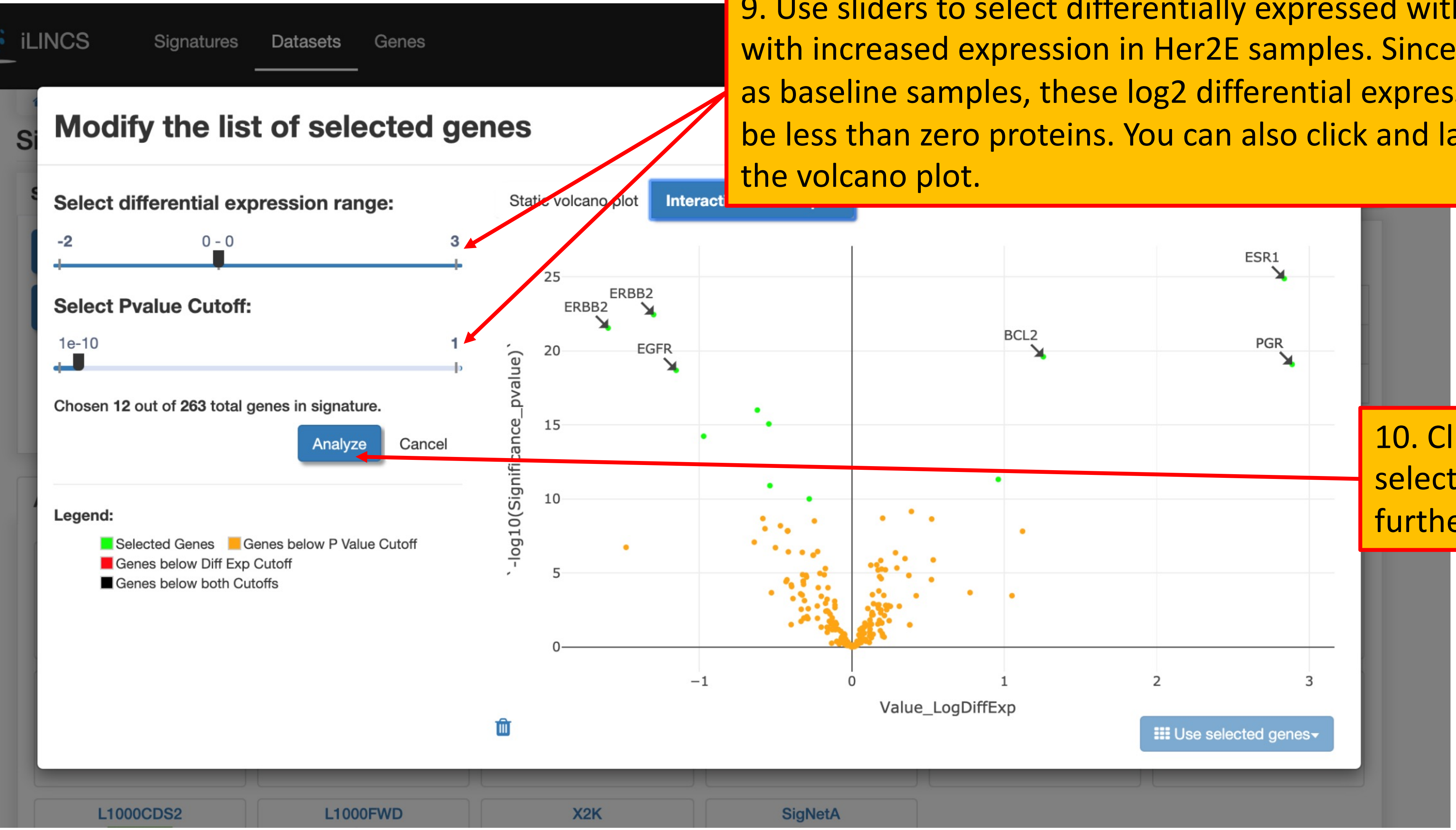

9. Use sliders to select differentially expressed with p-value<10e-10 that with increased expression in Her2E samples. Since Her2E samples were used as baseline samples, these log2 differential expression of these proteins will be less than zero proteins. You can also click and label individual proteins in the volcano plot.

10. Click analyze to use the selected 12 proteins in further analyses

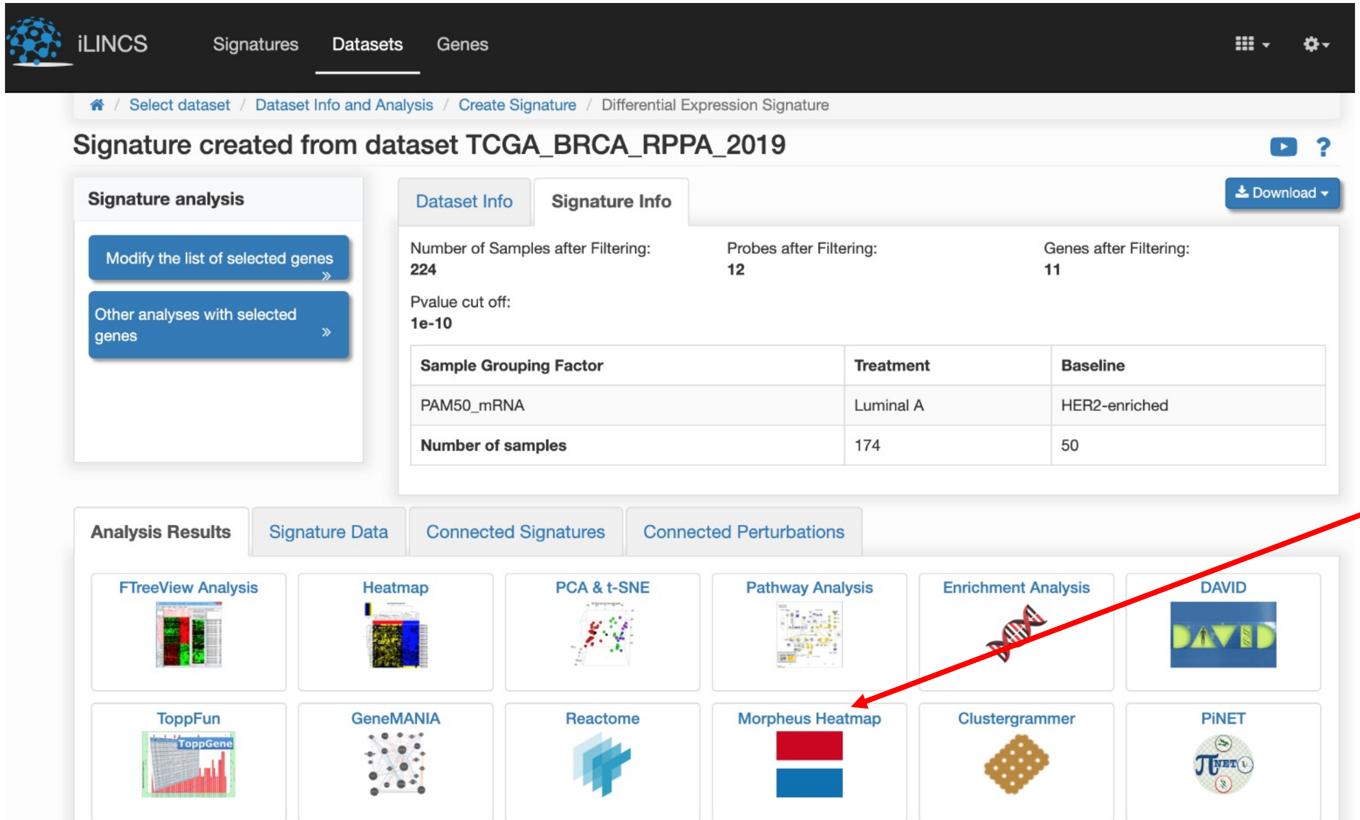

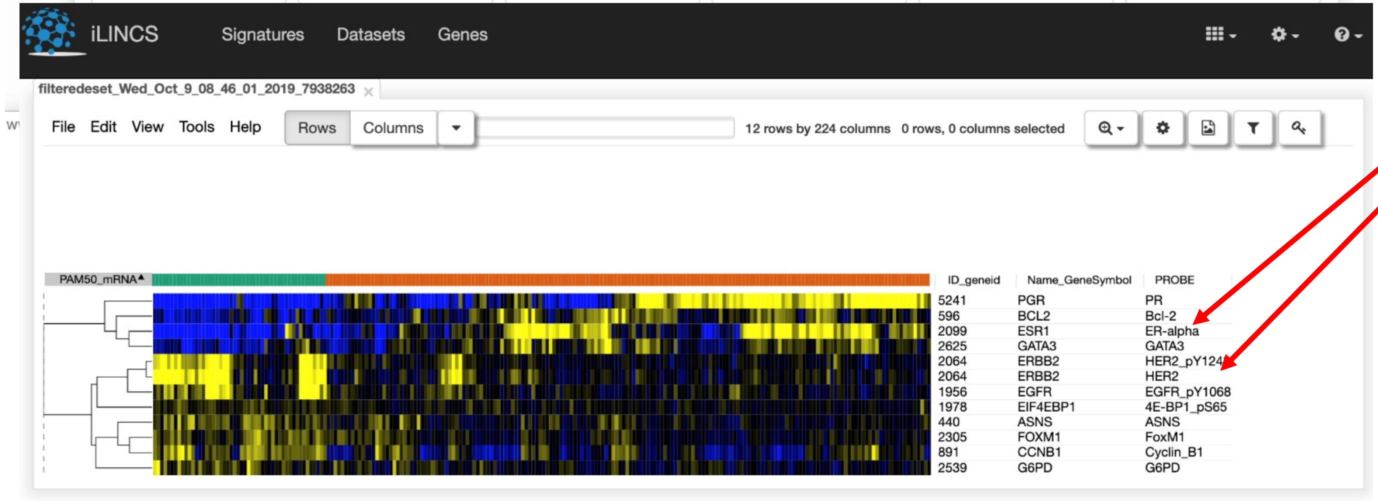

11. Click to view the heatmap of the 12 proteins in the analysis of the corresponding RNA-seq datasest

Most differentially expressed proteins are known drivers of Her2E and Luminal A BRC subtypes

Use Case 3: Proteo-genomics analysis of cancer driver events in breast cancer

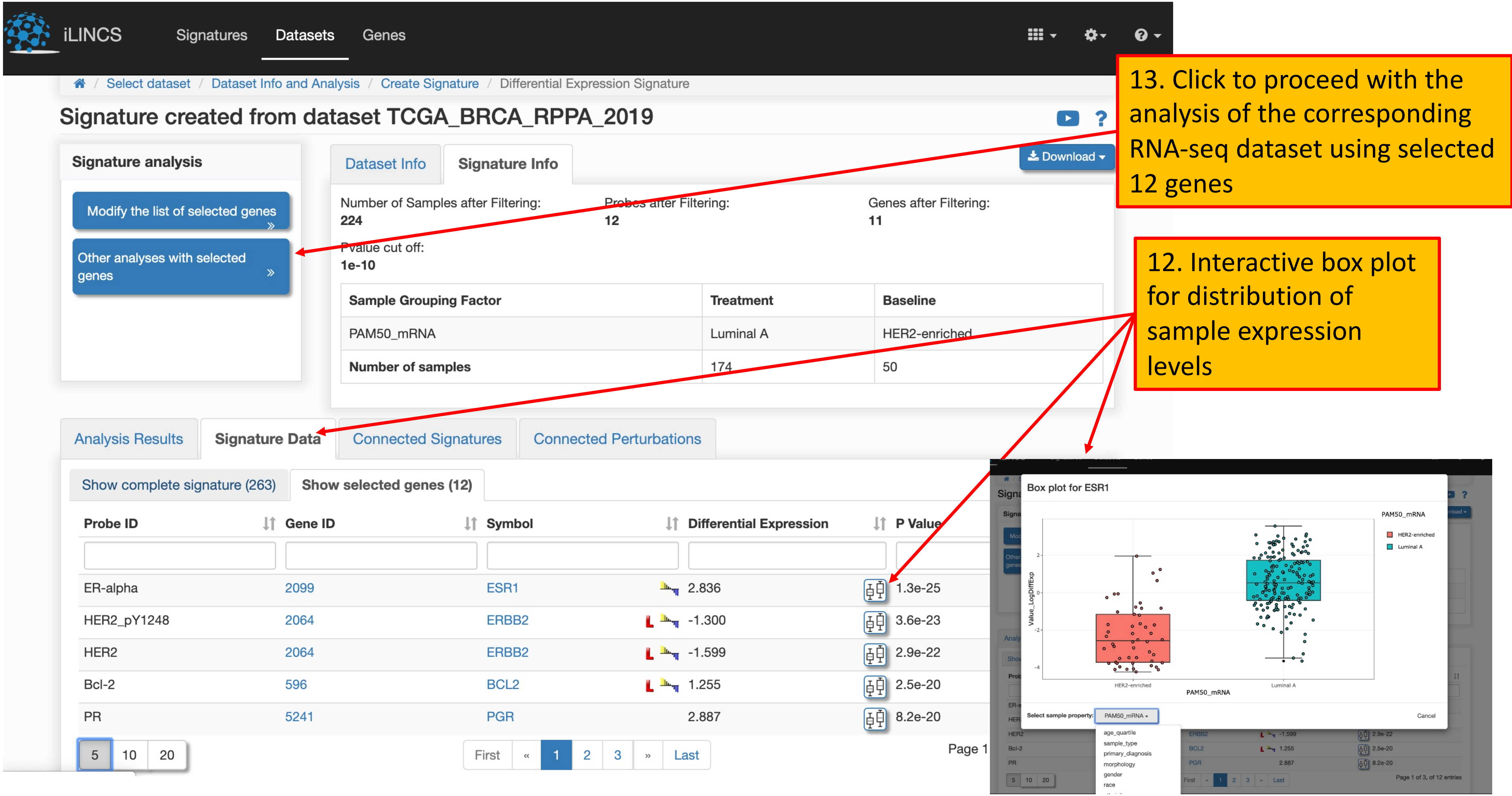

Use Case 3: Proteo-genomics analysis of cancer driver events in breast cancer

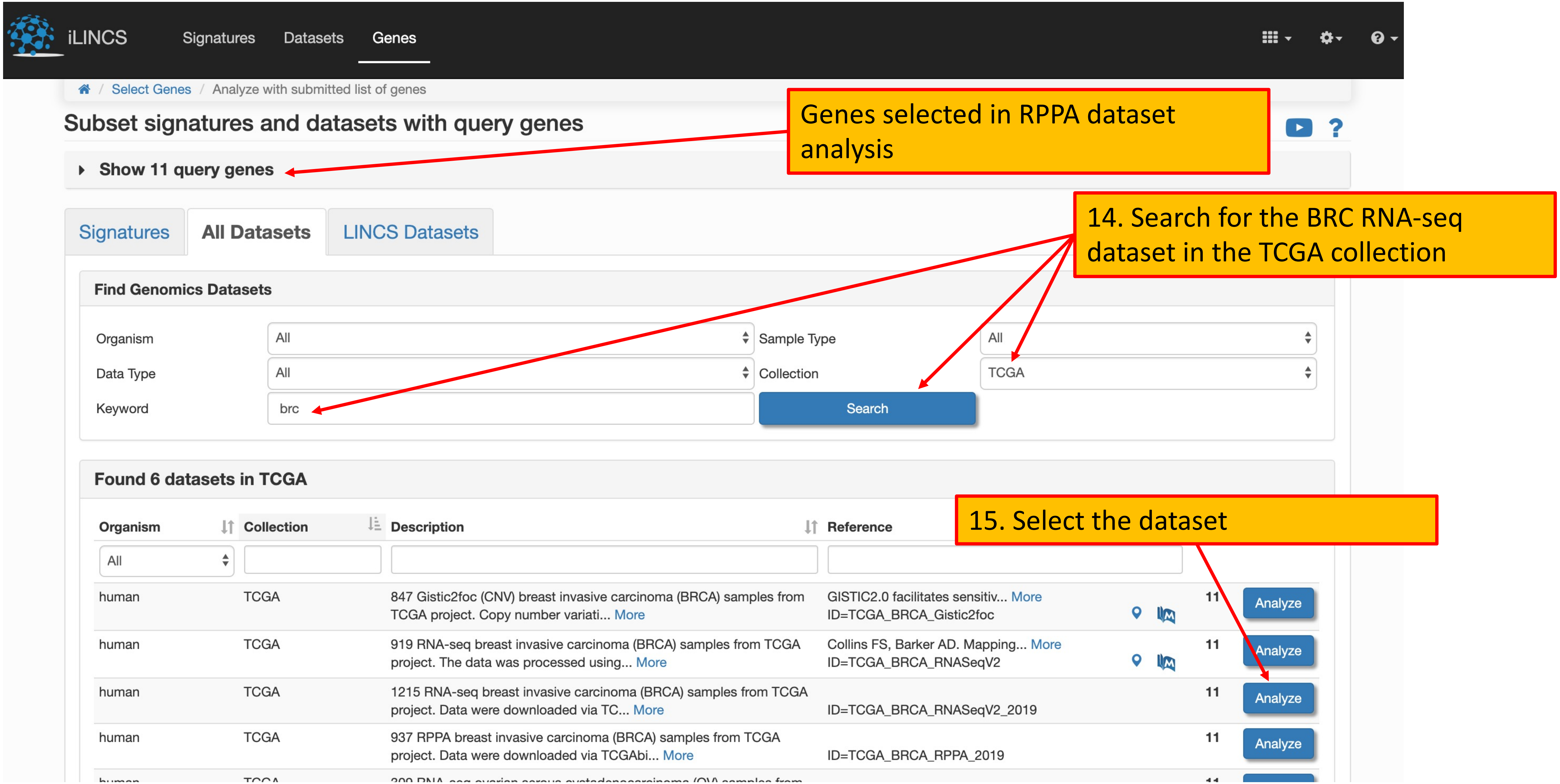
iLINCS
Signatures
Datasets
Genes
Select Genes / Analyze with submitted list of genes
Subset signatures and datasets with query genes
Show 11 query genes
Genes selected in RPPA dataset analysis
Signatures
All Datasets
LINCS Datasets
14. Search for the BRC RNA-seq dataset in the TCGA collection
Find Genomics Datasets
Organism
All
Sample Type
All
Data Type
All
Collection
TCGA
Keyword
brc
Search
Found 6 datasets in TCGA
Organism
Collection
Description
Reference
15. Select the dataset
All
human
TCGA
847 Gistic2foc (CNV) breast invasive carcinoma (BRCA) samples from TCGA project. Copy number variati... More
GISTIC2.0 facilitates sensitiv... More
ID=TCGA_BRCA_Gistic2foc
11
Analyze
human
TCGA
919 RNA-seq breast invasive carcinoma (BRCA) samples from TCGA project. The data was processed using... More
Collins FS, Barker AD. Mapping... More
ID=TCGA_BRCA_RNASeqV2
11
Analyze
human
TCGA
1215 RNA-seq breast invasive carcinoma (BRCA) samples from TCGA project. Data were downloaded via TC... More
ID=TCGA_BRCA_RNASeqV2_2019
11
Analyze
human
TCGA
937 RPPA breast invasive carcinoma (BRCA) samples from TCGA project. Data were downloaded via TCGAbi... More
ID=TCGA_BRCA_RPPA_2019
11
Analyze

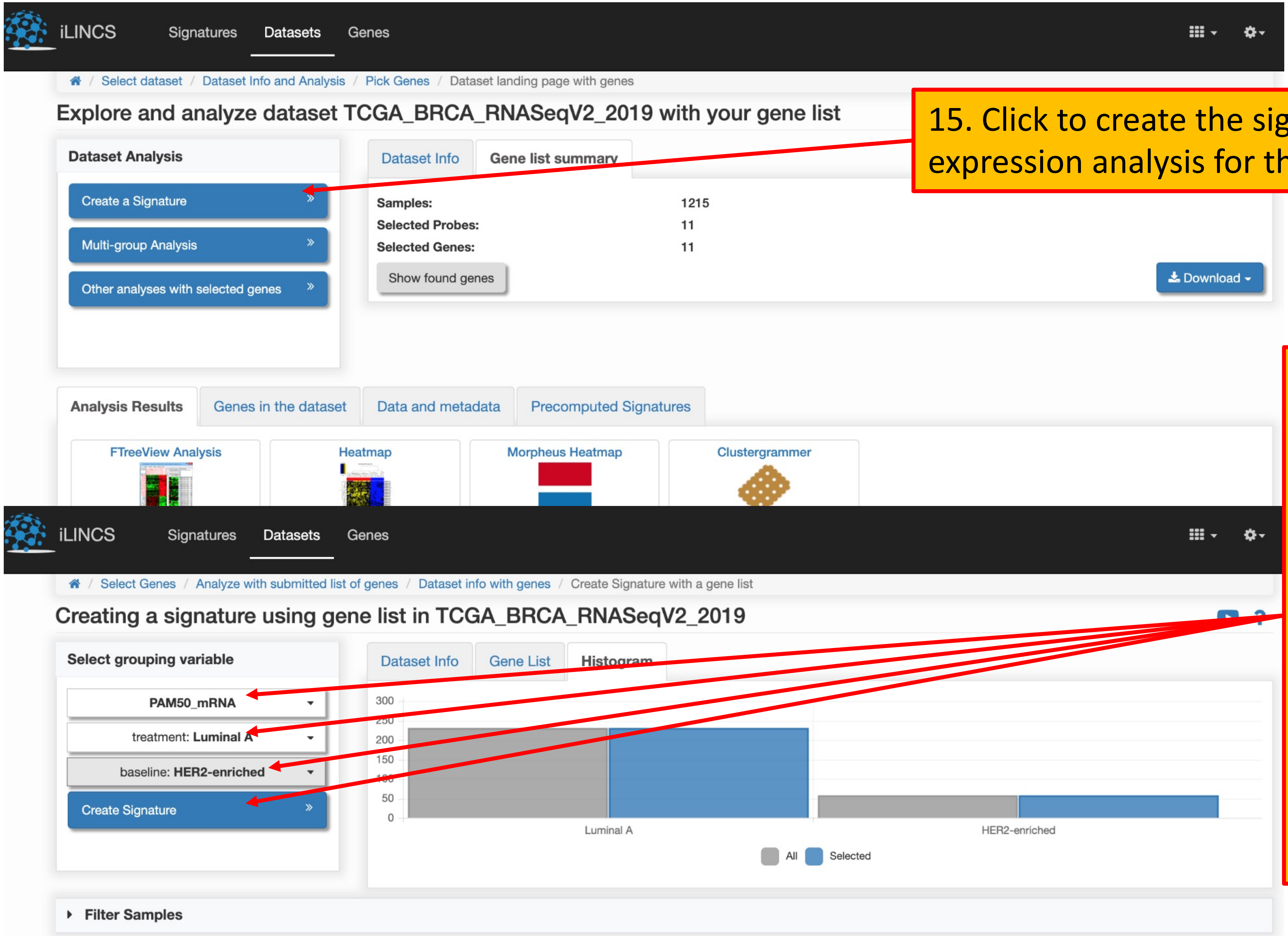

15. Click to create the signature (ie perform differential expression analysis for the selected 11 genes)

16. Select the sample property (ie factor) that will be used to construct two groups of samples for signature creation

17. Select the value of the factor that will indicate treatment and baseline sample groups

18. Perform differential gene expression analysis between treatment and baseline samples

19. View the gene by gene analysis results

20. View the hetamap

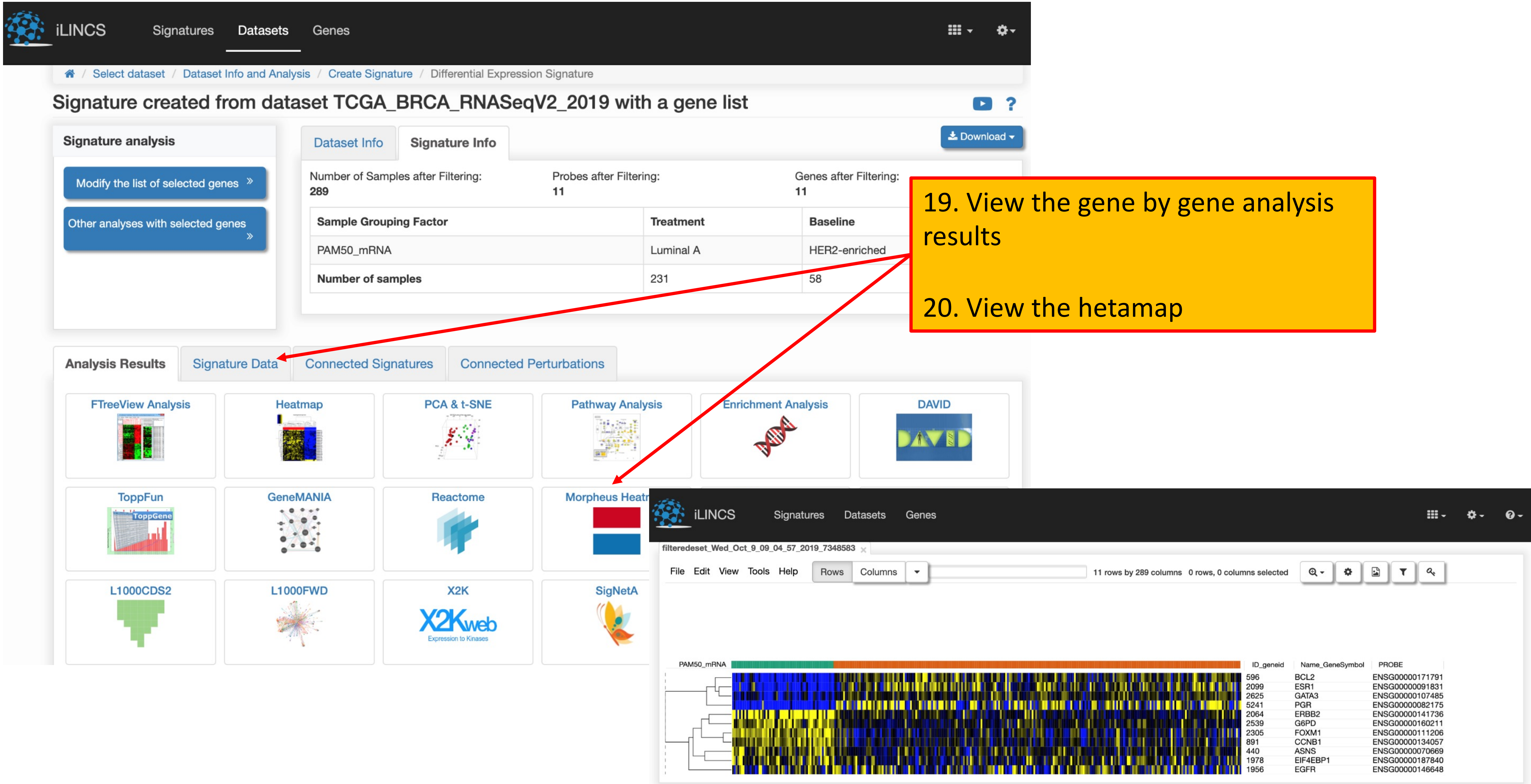

Use Case 3: Proteo-genomics analysis of cancer driver events in breast cancer

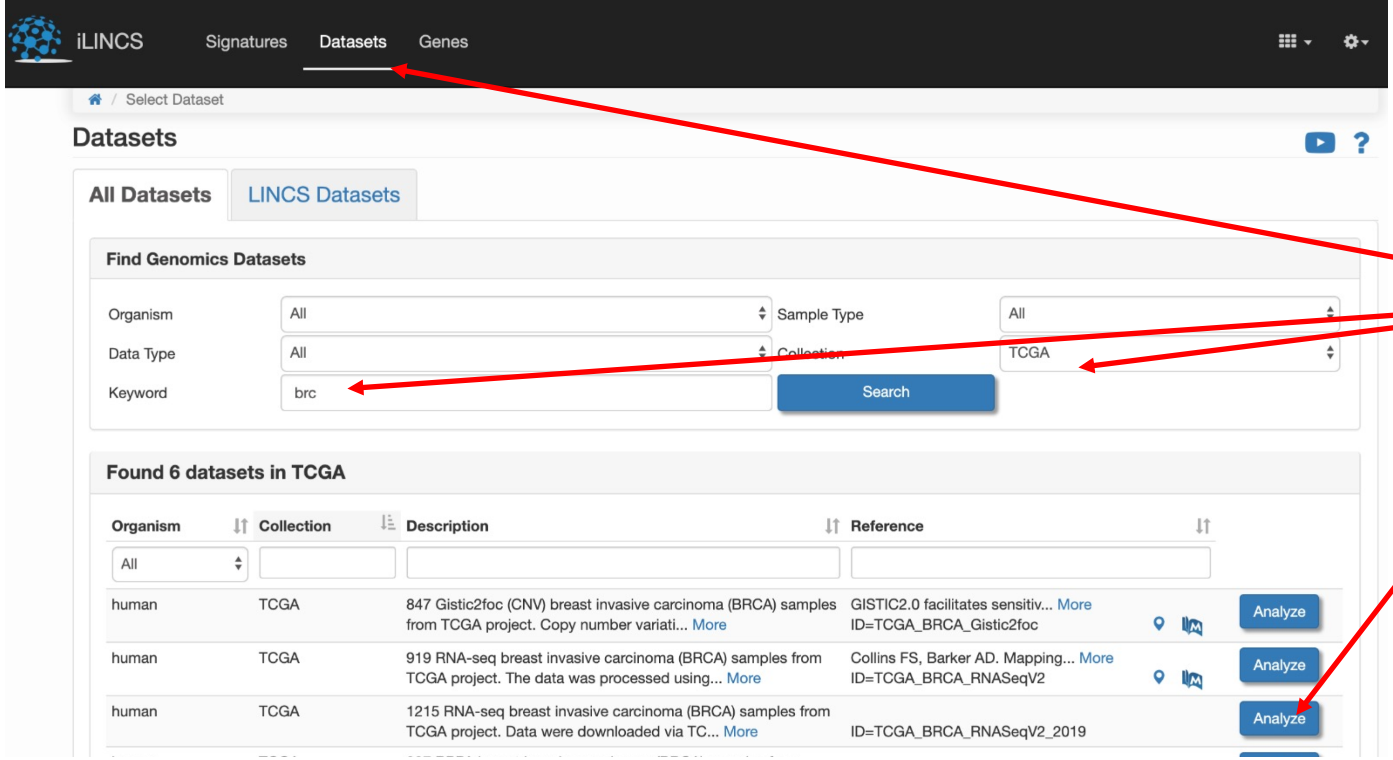

21. Start with the Datasets workflow

22. Search Datasets for BRC in the TCGA collection

23. Select the BRC RNA-seq dataset for the analysis

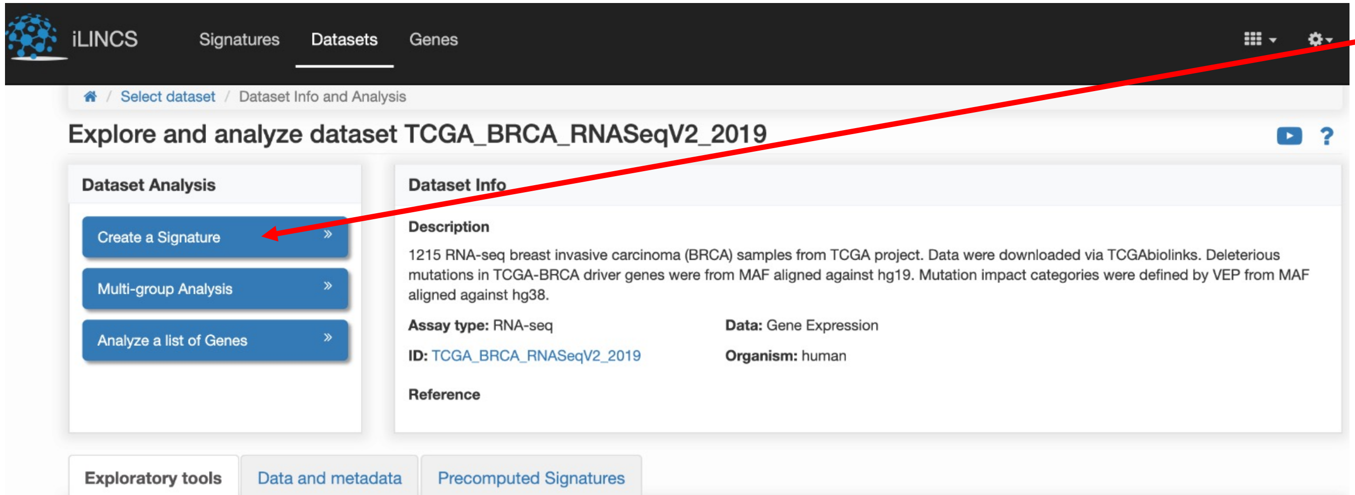

24. Initiate the full signature creation

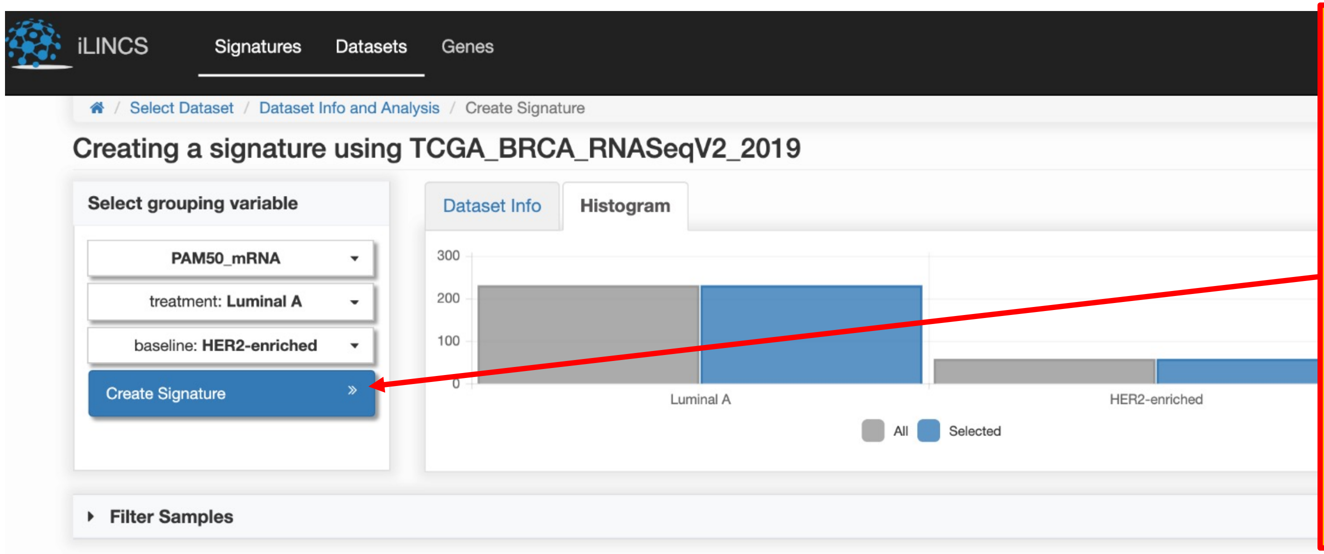

25. Select the sample property (ie factor) that will be used to construct two groups of samples for signature creation

26. Select the value of the factor that will indicate treatment and baseline sample groups

27. Perform differential gene expression analysis between treatment and baseline samples

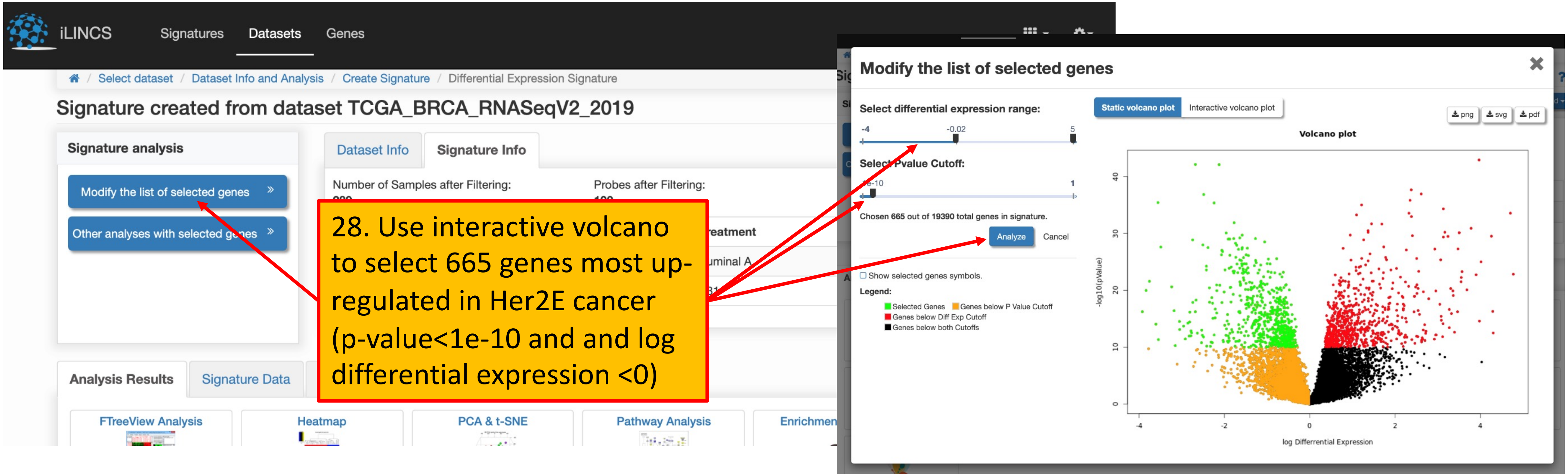

28. Use interactive volcano to select 665 genes most up-regulated in Her2E cancer (p-value<1e-10 and and log differential expression <0)

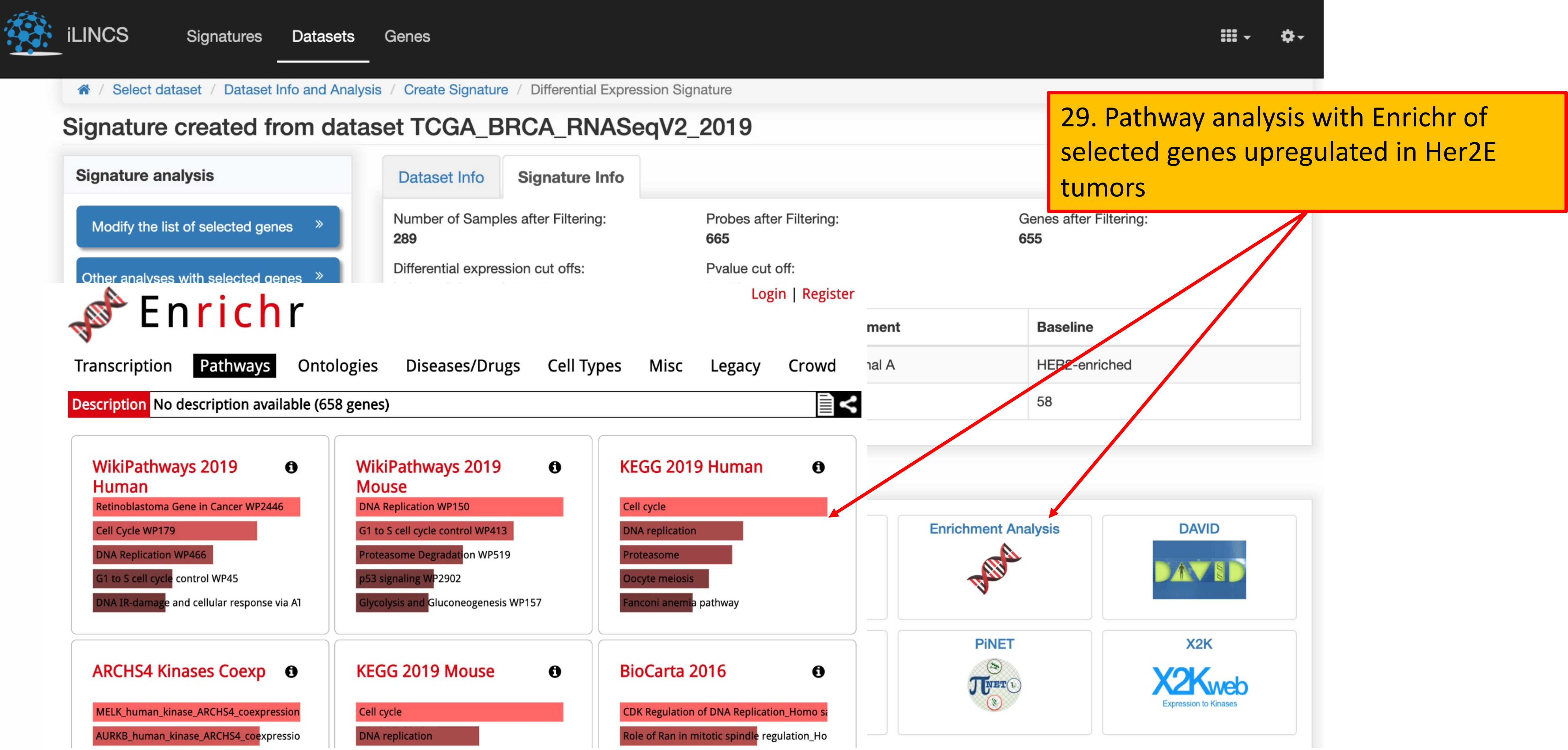
iLINCS
Signatures
Datasets
Genes
Select dataset / Dataset Info and Analysis / Create Signature / Differential Expression Signature
Signature created from dataset TCGA_BRCA_RNASeqV2_2019
Signature analysis
Modify the list of selected genes
Other analyses with selected genes
Dataset Info
Signature Info
Number of Samples after Filtering:
289
Probes after Filtering:
665
Genes after Filtering:
655
Differential expression cut offs:
Pvalue cut off:
Baseline
HER2-enriched
58
Enrichment Analysis
DAVID
PiNET
X2K
X2Kweb
Expression to Kinases
Enrichr
Login | Register
Transcription
Pathways
Ontologies
Diseases/Drugs
Cell Types
Misc
Legacy
Crowd
Description
No description available (658 genes)
WikiPathways 2019 Human
Retinoblastoma Gene in Cancer WP2446
Cell Cycle WP179
DNA Replication WP466
G1 to S cell cycle control WP45
WikiPathways 2019 Mouse
DNA Replication WP150
G1 to S cell cycle control WP413
Proteasome Degradation WP519
p53 signaling WP2902
Glycolysis and Gluconeogenesis WP157
KEGG 2019 Human
Cell cycle
DNA replication
Proteasome
Oocyte meiosis
Fanconi anemia pathway
ARCHS4 Kinases Coexp
MELK_human_kinase_ARCHS4_coexpression
KEGG 2019 Mouse
Cell cycle
DNA replication
BioCarta 2016
29. Pathway analysis with Enrichr of selected genes upregulated in Her2E tumors

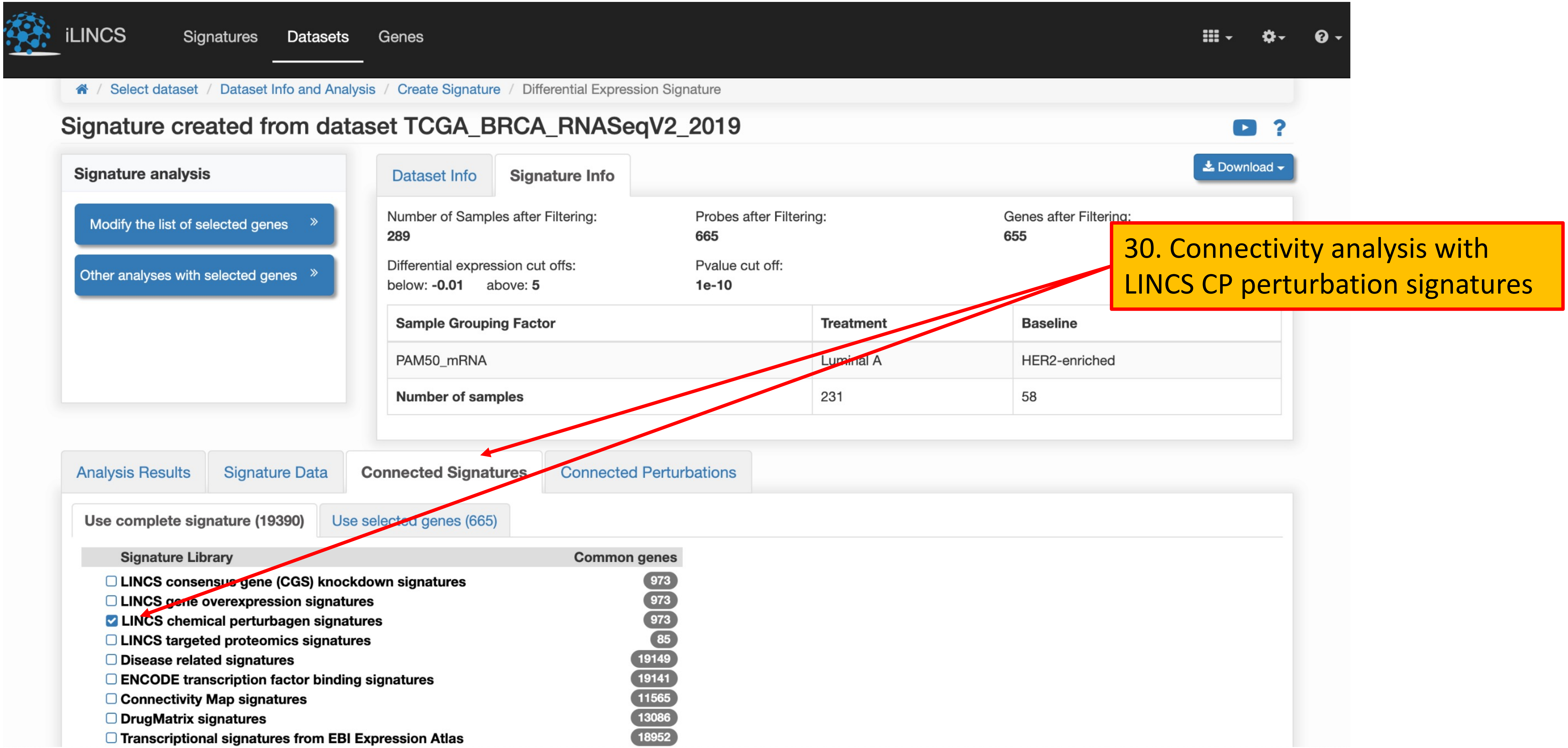

iLINCS
Signatures
Datasets
Genes
Select dataset / Dataset Info and Analysis / Create Signature / Differential Expression Signature
Signature created from dataset TCGA_BRCA_RNASeqV2_2019
Signature analysis
Modify the list of selected genes
Other analyses with selected genes
Dataset Info
Signature Info
Download
Number of Samples after Filtering: 289
Probes after Filtering: 665
Genes after Filtering: 655
Differential expression cut offs: below: -0.01 above: 5
Pvalue cut off: 1e-10
Sample Grouping Factor
Treatment
Baseline
PAM50_mRNA
Luminal A
HER2-enriched
Number of samples
231
58
Analysis Results
Signature Data
Connected Signatures
Connected Perturbations
Use complete signature (19390)
Use selected genes (665)
Signature Library
Common genes
LINCS consensus gene (CGS) knockdown signatures 973
LINCS gene overexpression signatures 973
LINCS chemical perturbagen signatures 973
LINCS targeted proteomics signatures 85
Disease related signatures 19149
ENCODE transcription factor binding signatures 19141
Connectivity Map signatures 11565
DrugMatrix signatures 13086
Transcriptional signatures from EBI Expression Atlas 18952
30. Connectivity analysis with LINCS CP perturbation signatures

The connectivity analysis provides the sorted list of most statistically significantly correlated signatures.

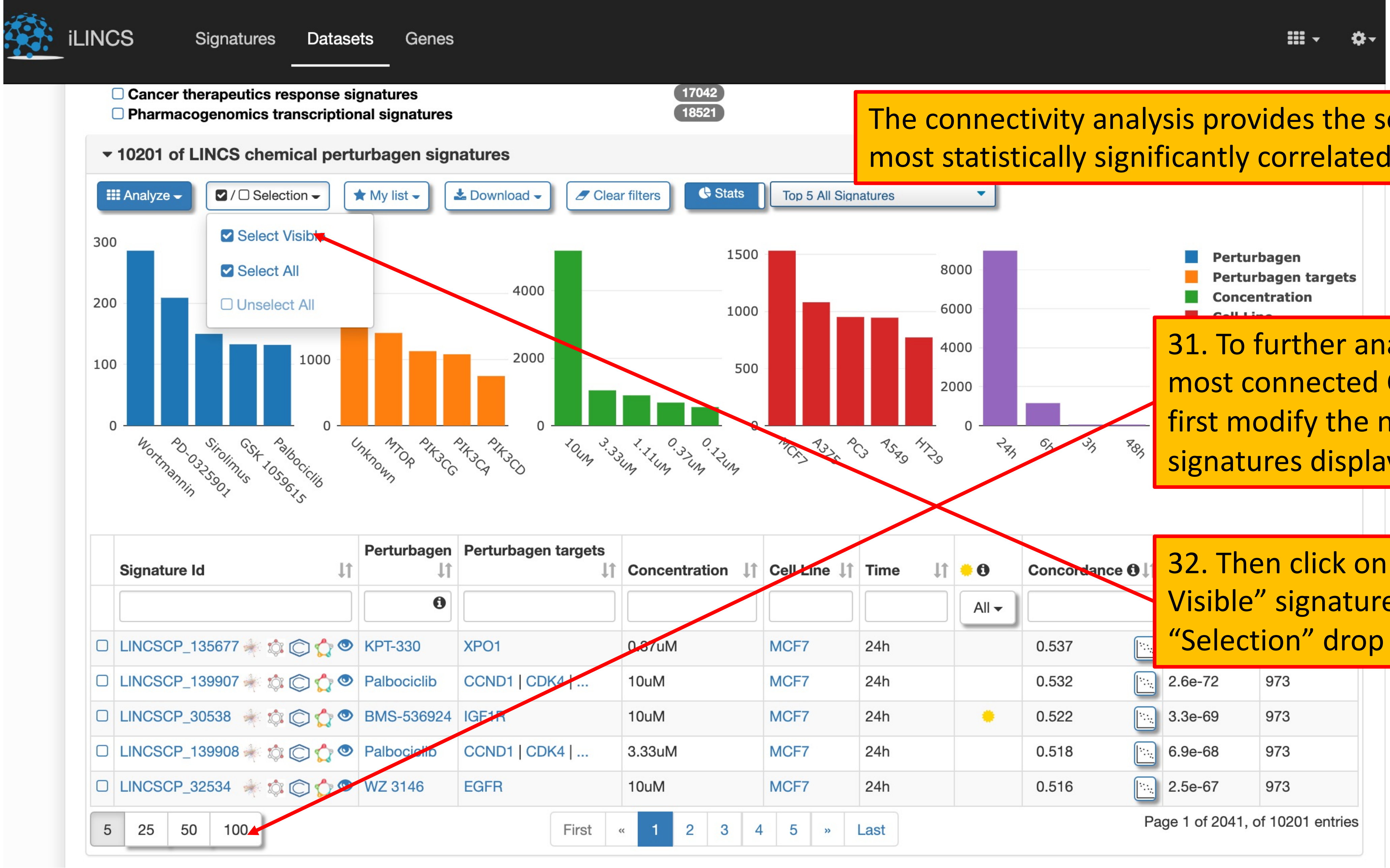

31. To further analyze top 100 most connected CP signatures, first modify the number of signatures displayed.

32. Then click on “Select Visible” signatures in the “Selection” drop down menu

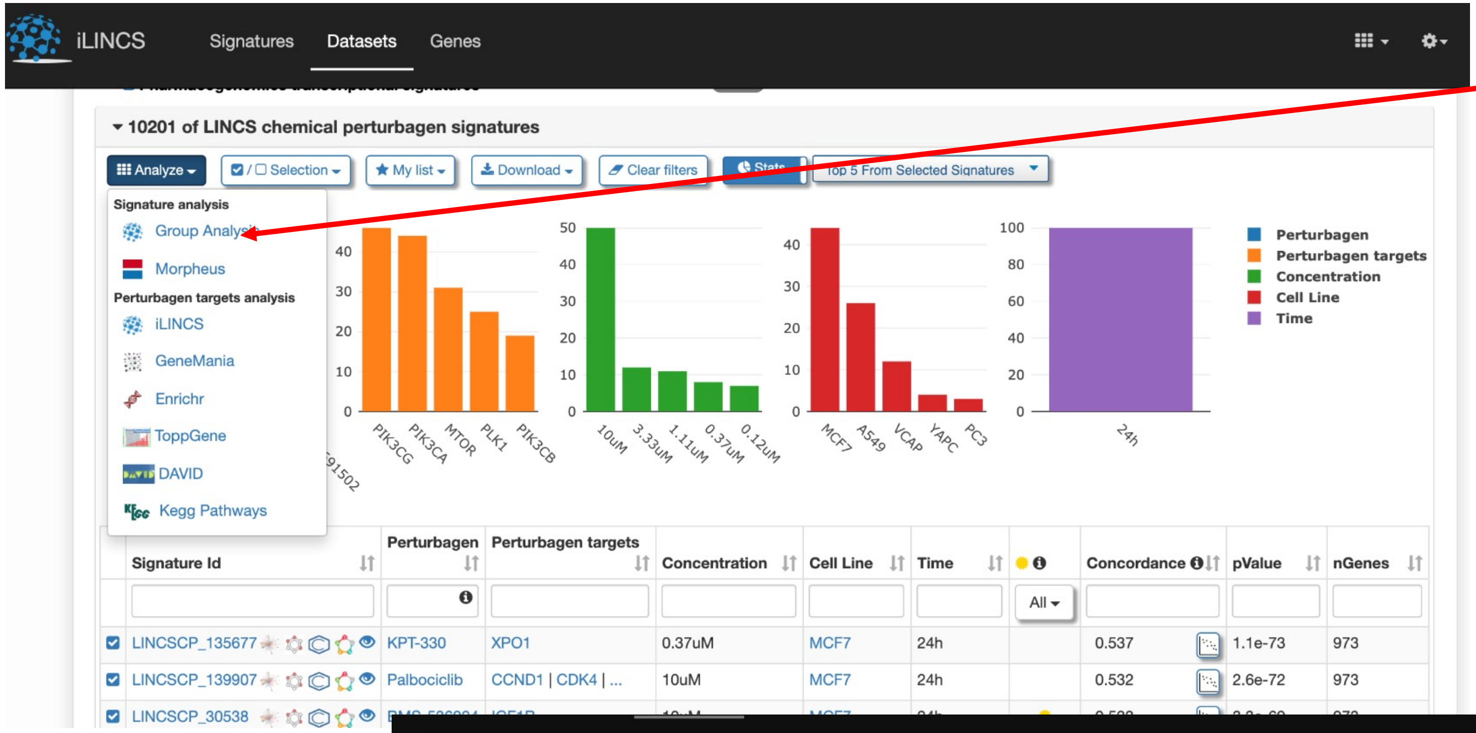

32. Select “Group Analysis” on the “Analysis” drop-down menu.

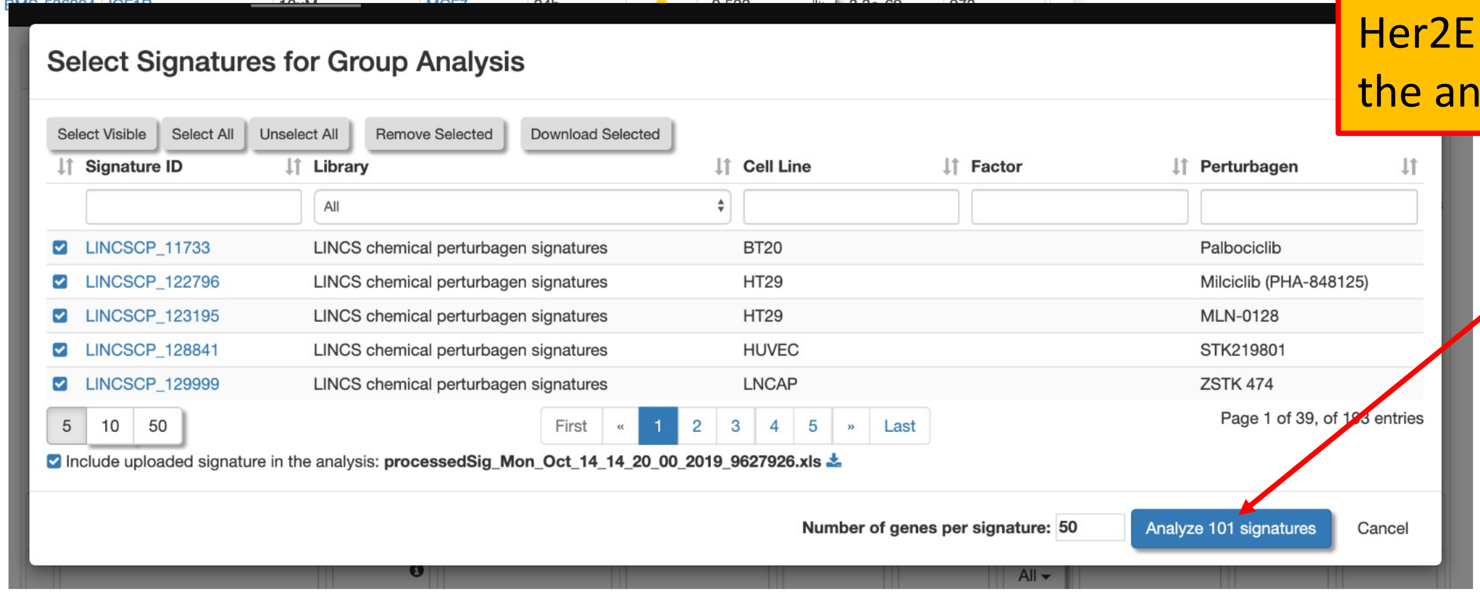

33. Confirm the selected signatures and the default number of most differentially expressed genes to be used from each signature. The number of signature is 101, because the original Luminal A vs Her2E signature is also included in the analysis.

34. To view the heatmap of expression changes in 101 signatures.

35. To perform pathway enrichment analysis for a set of co-expressed genes, select the cluster.

36. Right click on the (invisible in this case) column of selected gene symbols, end select “Export” on the context specific menu.

37. Submit the genes to Enrichr

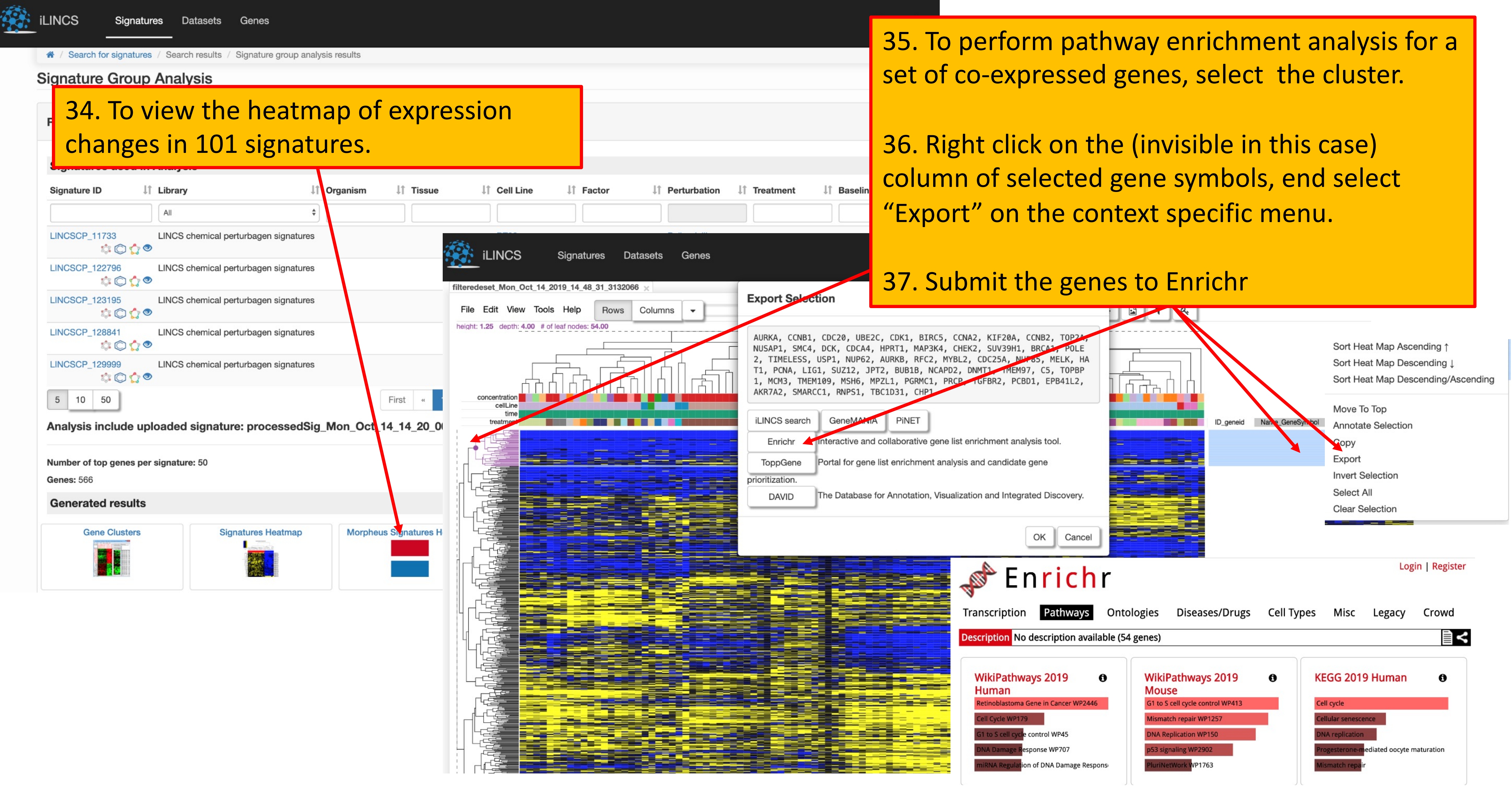

38. To view the heatmap of protein targets of corresponding perturbages.

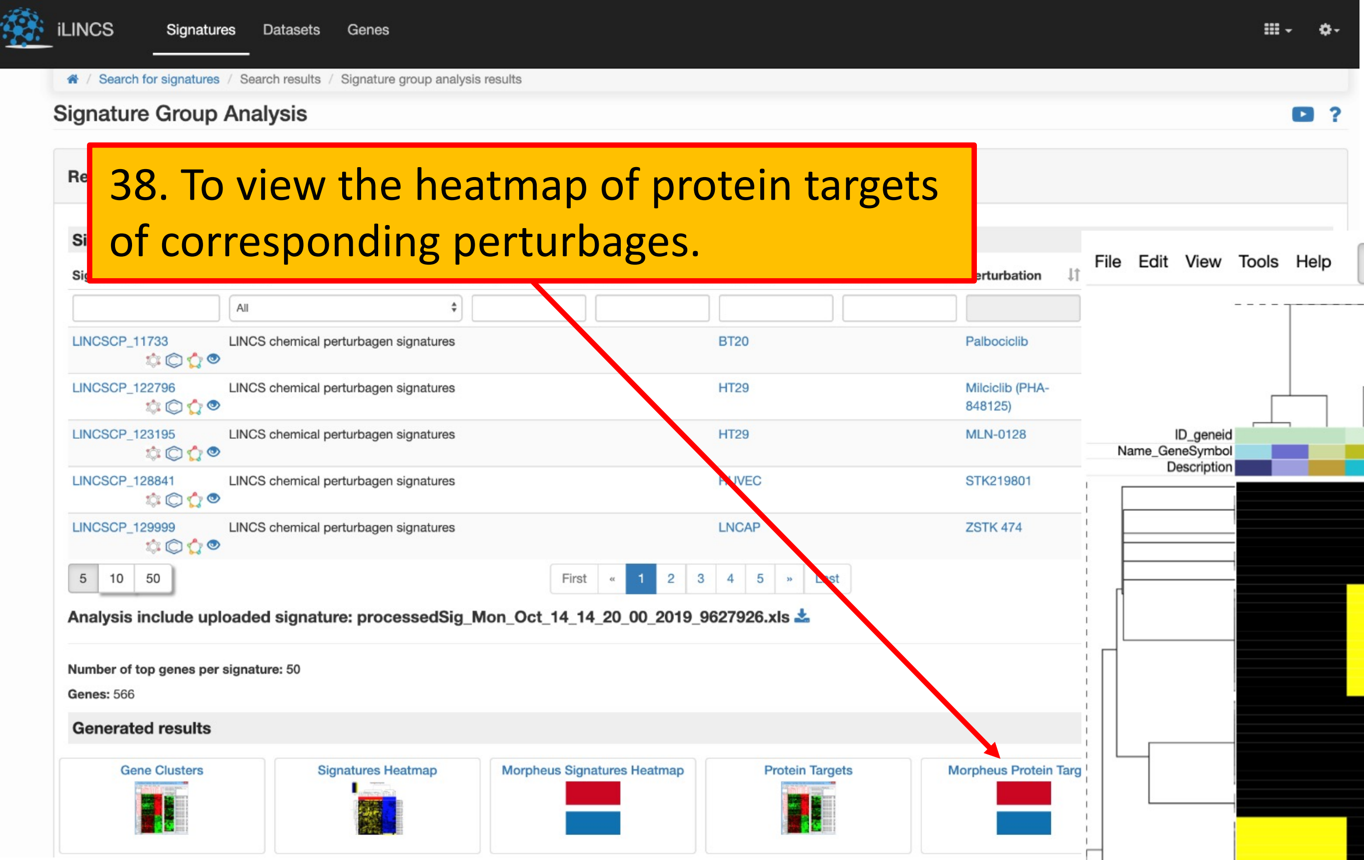

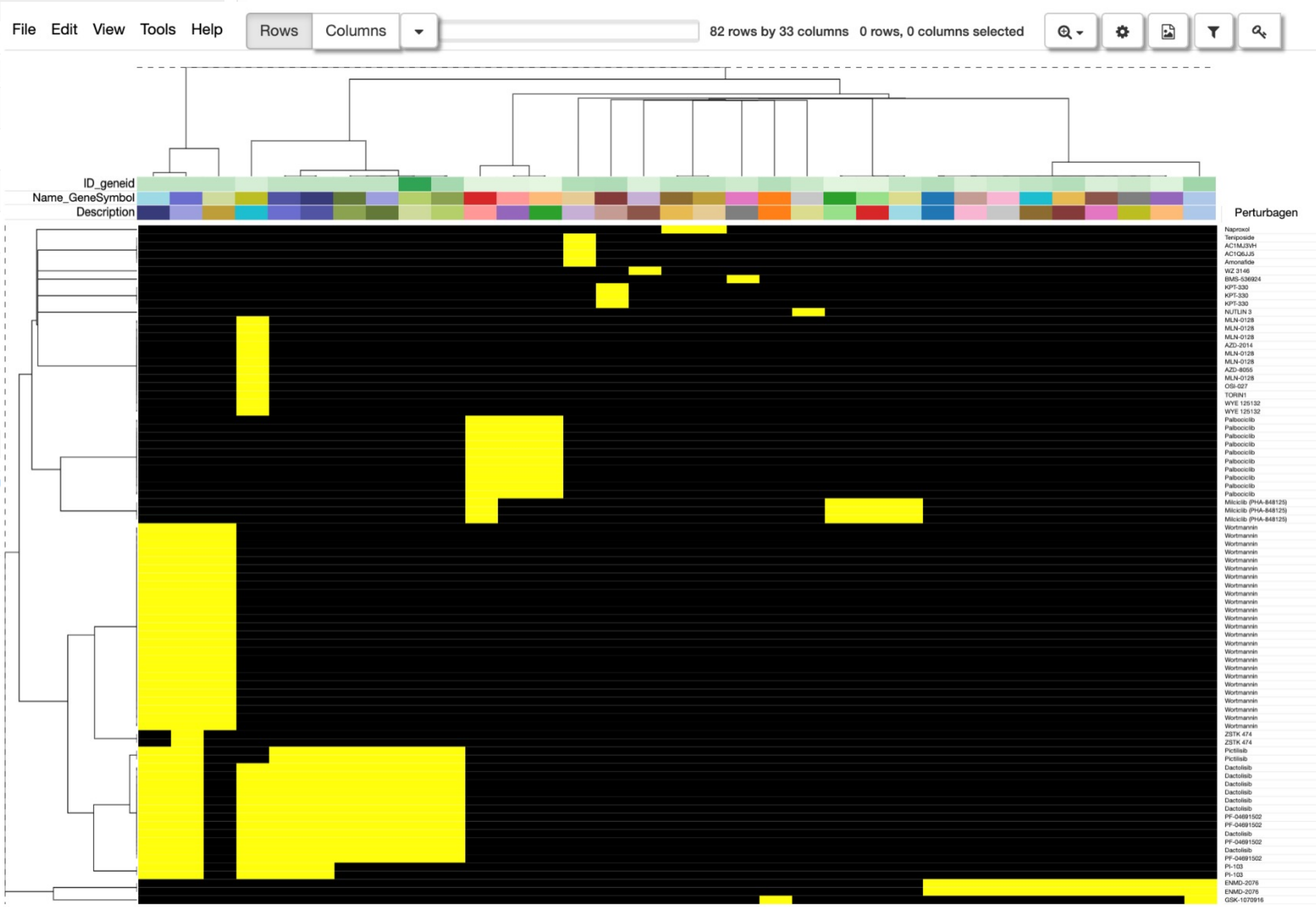

Use case 3: Drug repurposing for COVID-19

In this use case, we use iLINCS to construct a SARS-CoV-2 infection signature by re-analyzing a GEO dataset GSE147507. The signature was constructed by differential gene expression analysis of infected vs mock-infected A549 cell line genetically modified to express ACE2 gene which is required for viral entry into the cell. The resulting signature comprises many upregulated chemokines and other immune system related genes, including the EGR1 transcription factor that regulates inflammation and immune system response, and TNF and NK-kappa B signaling as the two pathways most enriched for upregulated genes (A). CMAP analysis against the LINCS gene over-expression signatures identifies the signature of LYN tyrosine kinase as the most positively correlated with the SARS-CoV-2 infection signature (B). The enrichment analysis of over-expressed genes with positively correlated signatures, drived by the over-expression signatures of the six NF-kappa B pathway regulators (LYN, TIRAP, BCL10, LTBR, TNF, CD40), identified NF-kappa B signaling pathway as the most enriched (B). This confirms mechanistically the gene expression enrichment analysis of the infection signature. CDK inhibitors and the drug Avlodicip are identified as the potential therapeutic strategies based on their ability to reverse the infection signature (C). suggested as a potential candidate for drug repurposing 75.

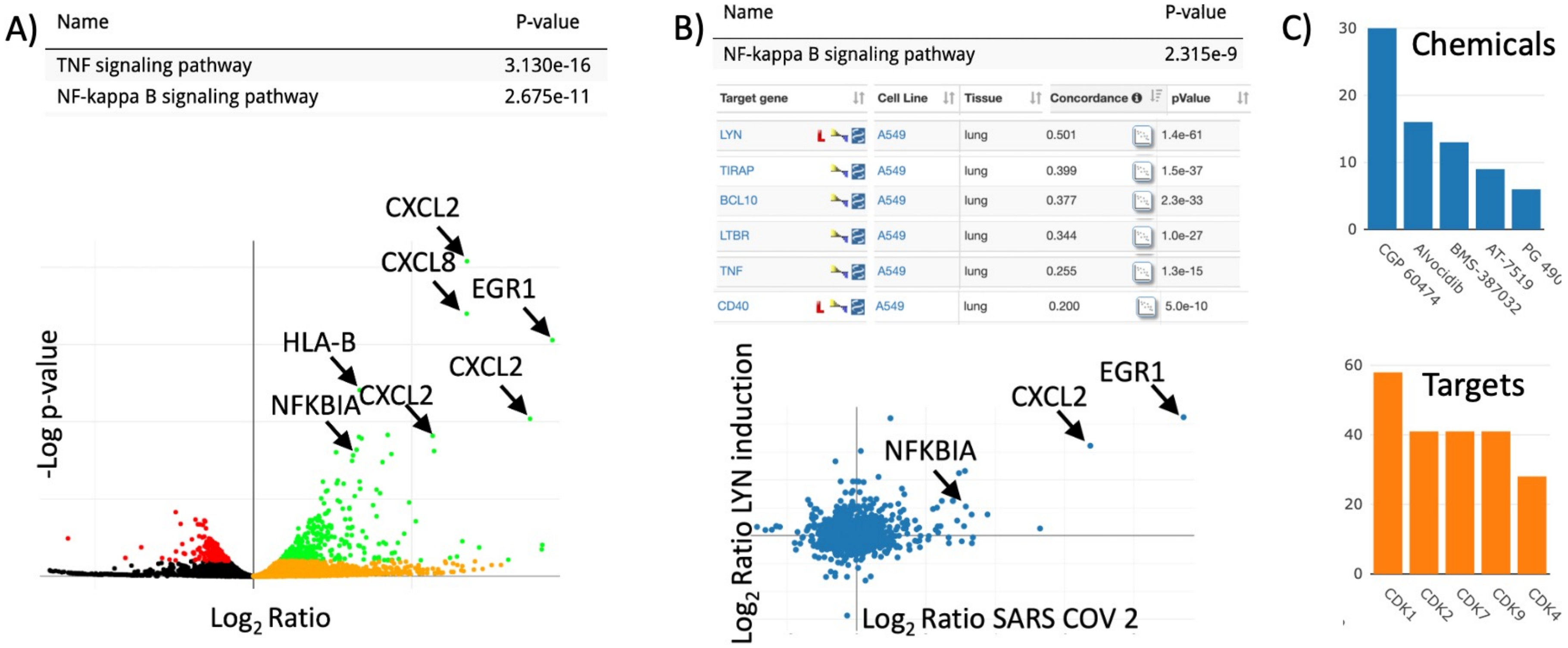

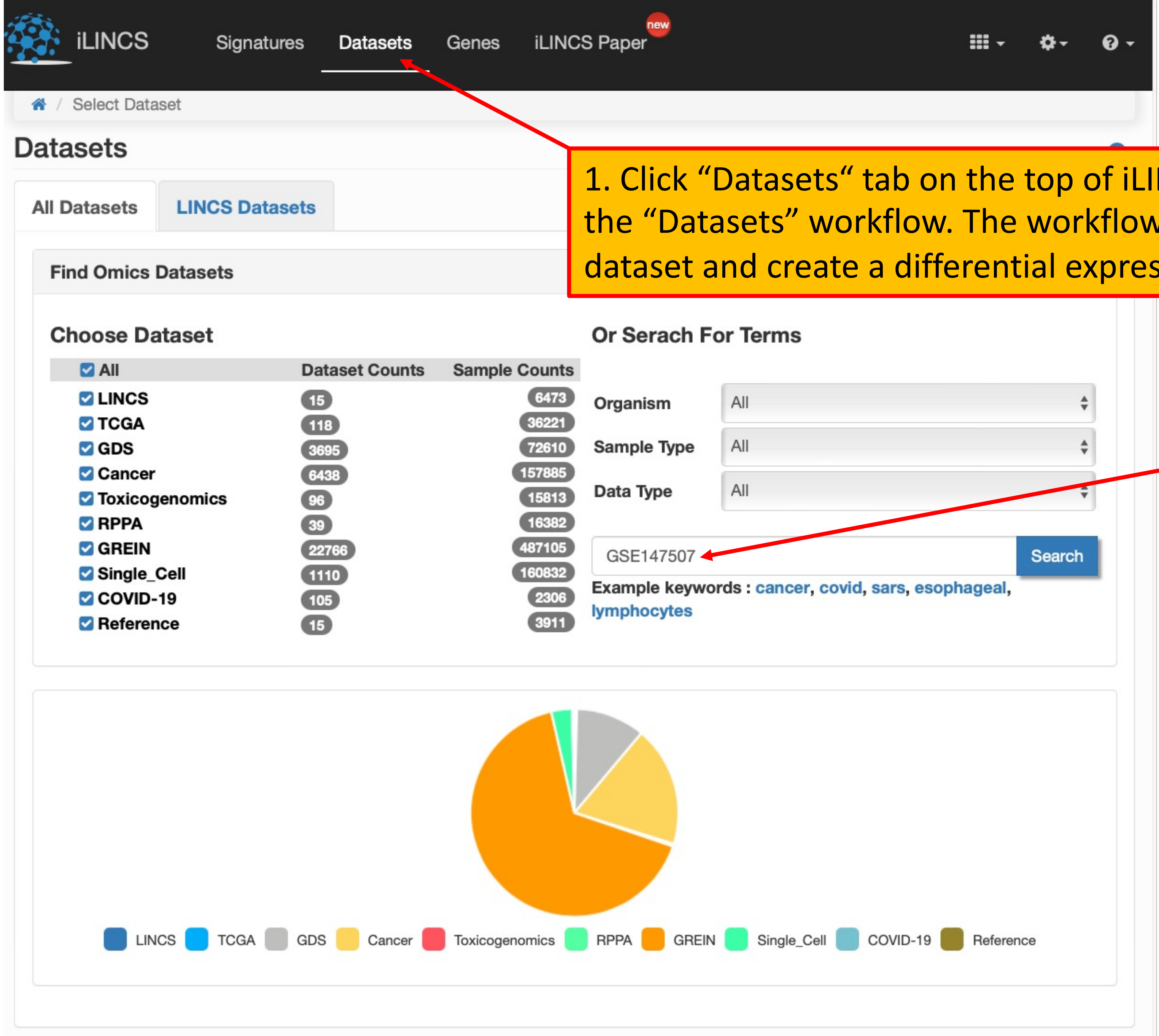

1. Click “Datasets“ tab on the top of iLINCS homepage to start with the “Datasets” workflow. The workflow allows you to select a dataset and create a differential expression gene/protein signature.

2. Search for GSE147507.

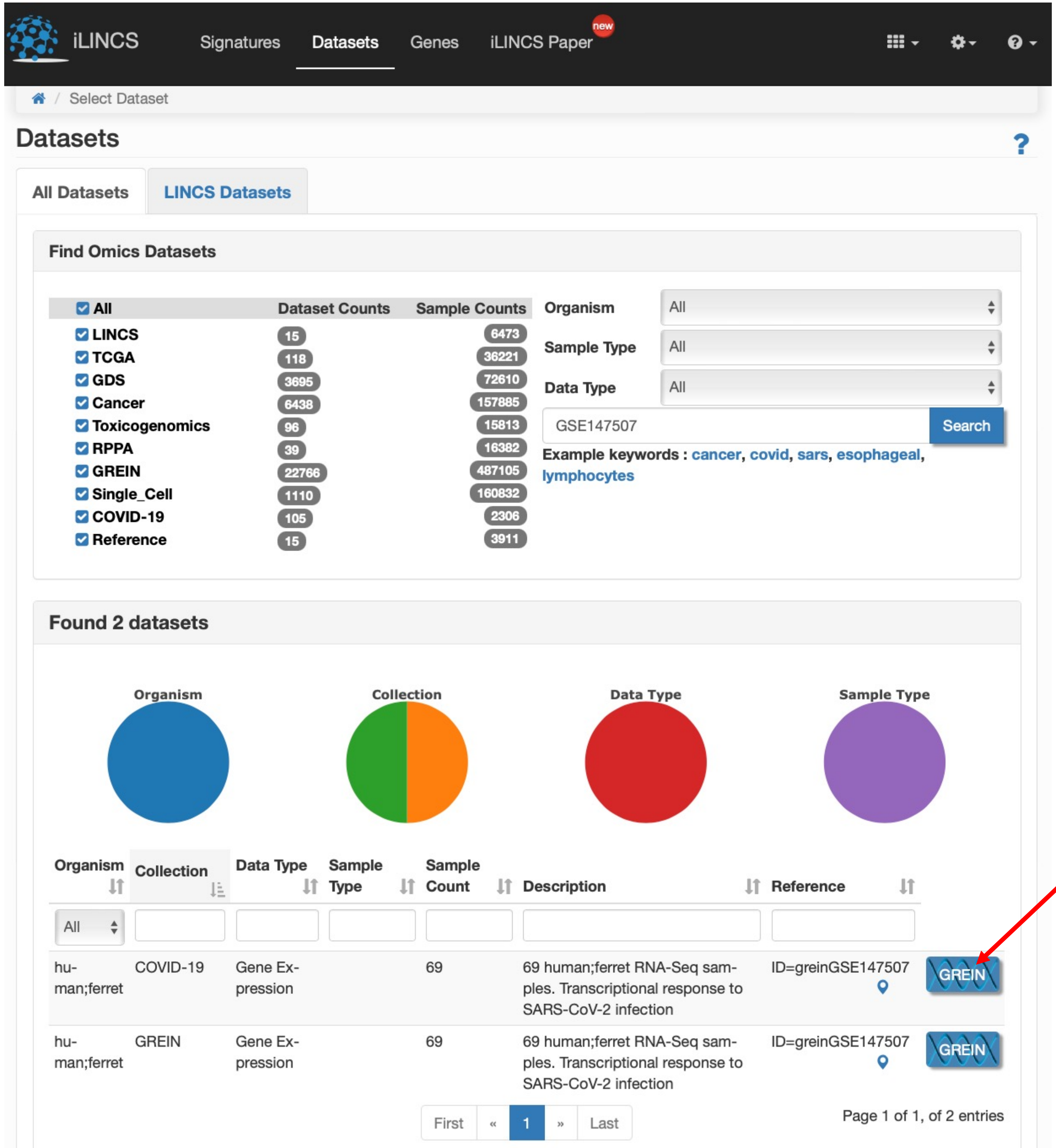

3. Select the dataset for the analysis.

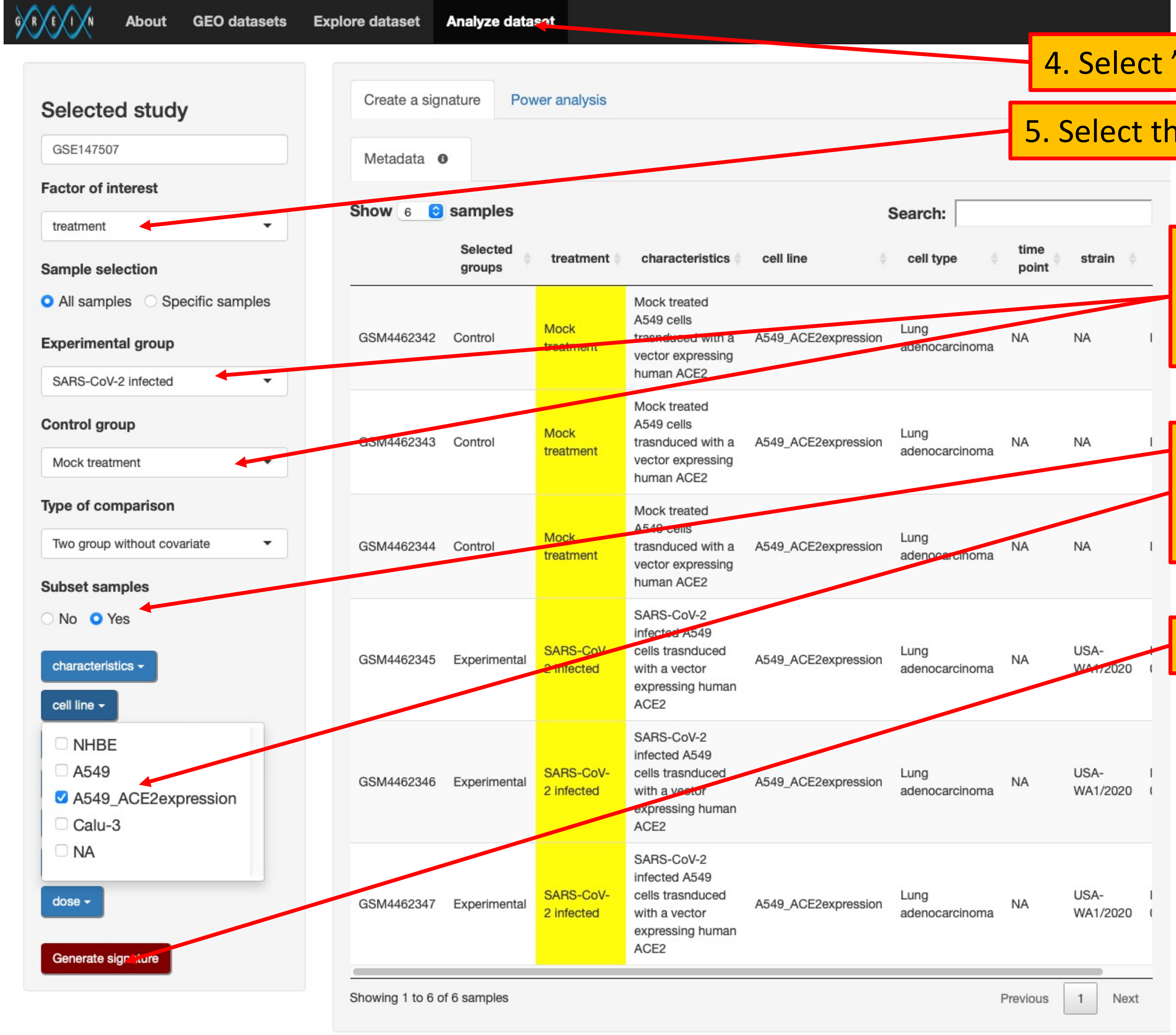

4. Select ”Analyze dataset” workflow

5. Select the “treatment” as factor of interest

6. Select the value of the factor that will indicate treatment and baseline sample groups

7. Choose “Yes” to subset samples to only one cell line and unselect all “cell lines” other than the “A549_ACE2expression”

8. Create signature

Signature in the form of the table of differential expressions and p-values for all 18,230 genes analyzed

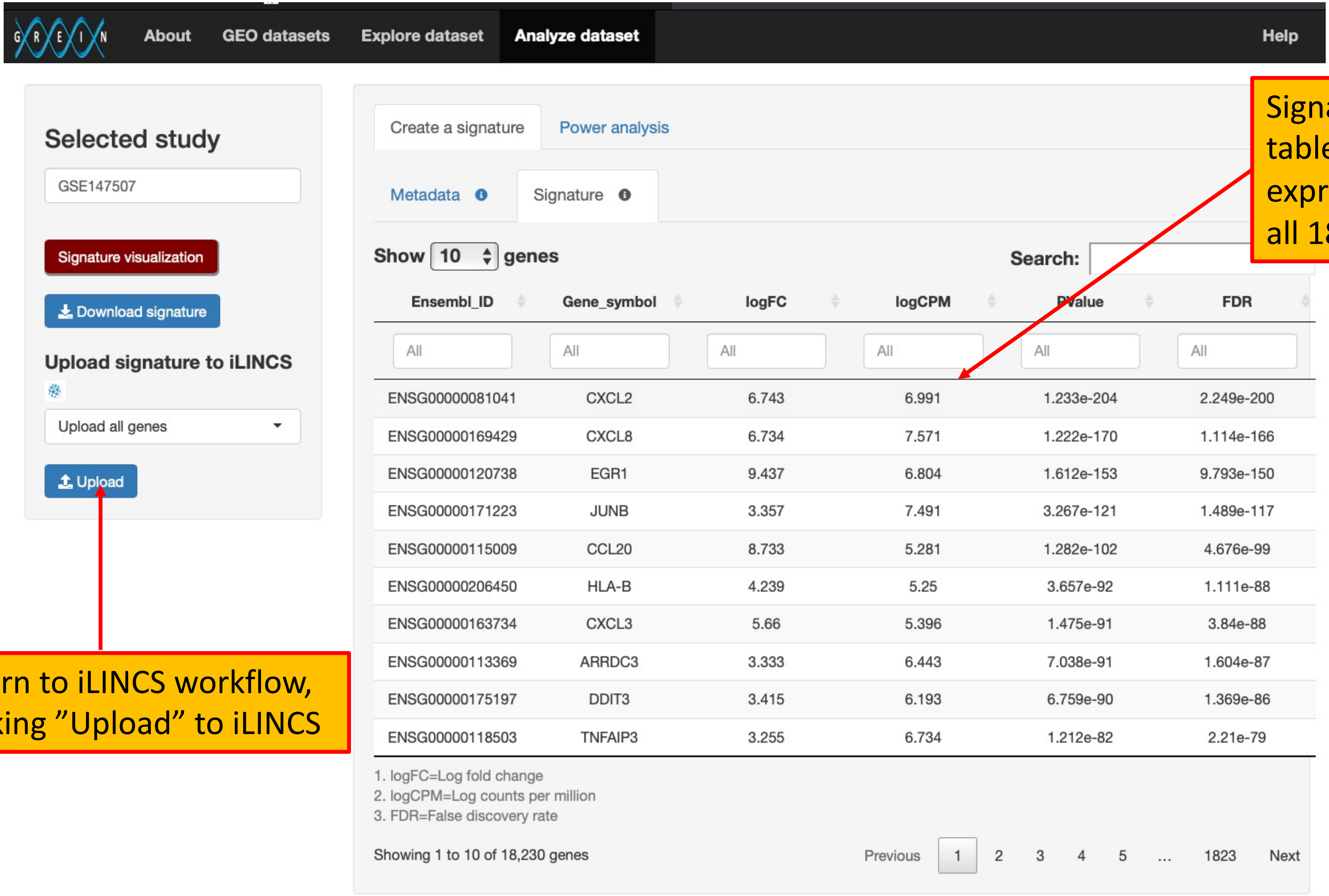

Ensembl_ID	Gene_symbol	logFC	logCPM	PValue	FDR
All	All	All	All	All	All
ENSG00000081041	CXCL2	6.743	6.991	1.233e-204	2.249e-200
ENSG00000169429	CXCL8	6.734	7.571	1.222e-170	1.114e-166
ENSG00000120738	EGR1	9.437	6.804	1.612e-153	9.793e-150
ENSG00000171223	JUNB	3.357	7.491	3.267e-121	1.489e-117
ENSG00000115009	CCL20	8.733	5.281	1.282e-102	4.676e-99
ENSG00000206450	HLA-B	4.239	5.25	3.657e-92	1.111e-88
ENSG00000163734	CXCL3	5.66	5.396	1.475e-91	3.84e-88
ENSG00000113369	ARRDC3	3.333	6.443	7.038e-91	1.604e-87
ENSG00000175197	DDIT3	3.415	6.193	6.759e-90	1.369e-86
ENSG00000118503	TNFAIP3	3.255	6.734	1.212e-82	2.21e-79

1. logFC=Log fold change
2. logCPM=Log counts per million
3. FDR=False discovery rate

9. Return to iLINCS workflow, by clicking ”Upload” to iLINCS

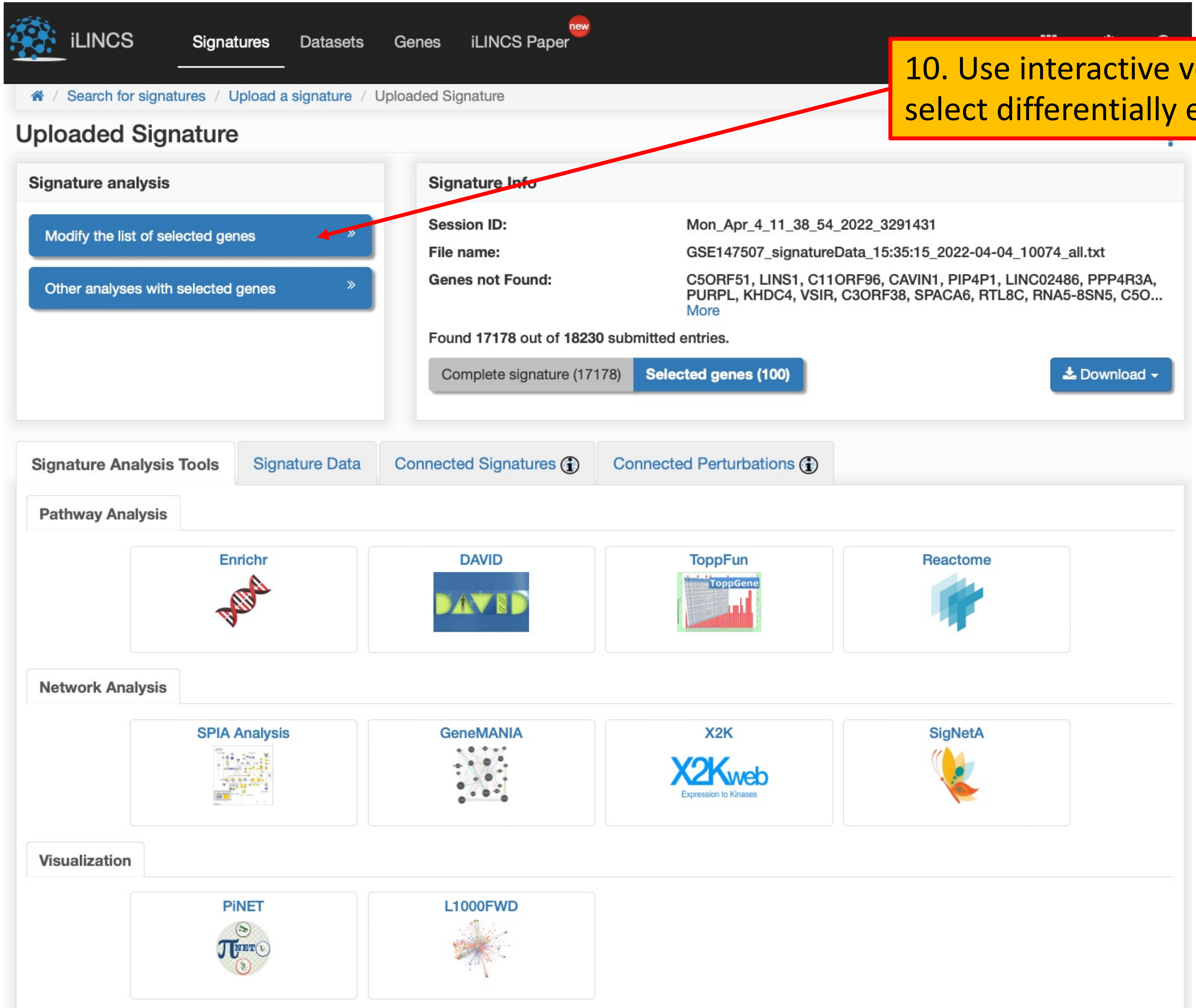

10. Use interactive volcano app to visualize and select differentially expressed genes

11. Use sliders to select genes upregulated after SARS-COV2 infection with p-value<1e-10.

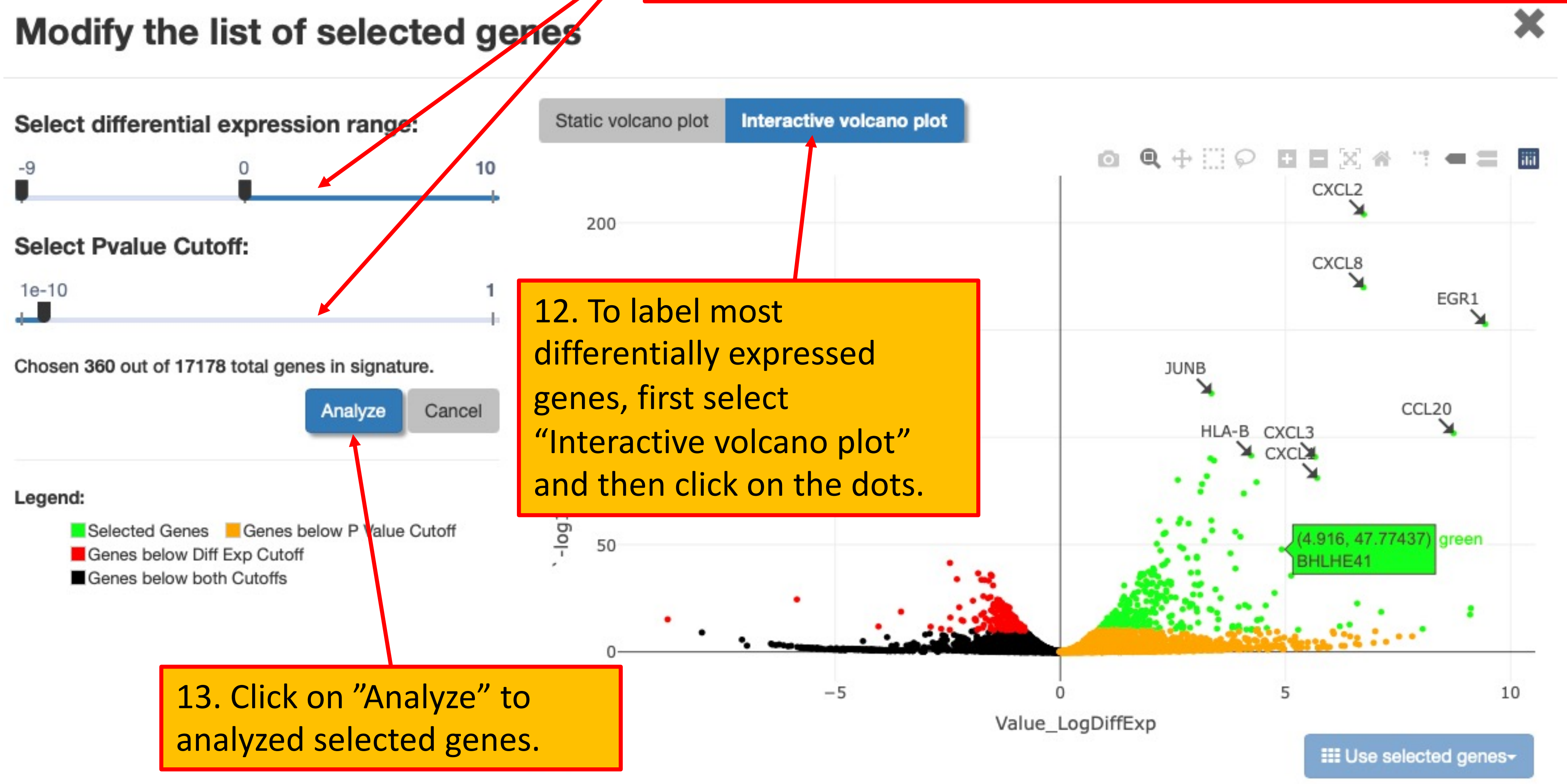

12. To label most differentially expressed genes, first select “Interactive volcano plot” and then click on the dots.

13. Click on ”Analyze” to analyzed selected genes.

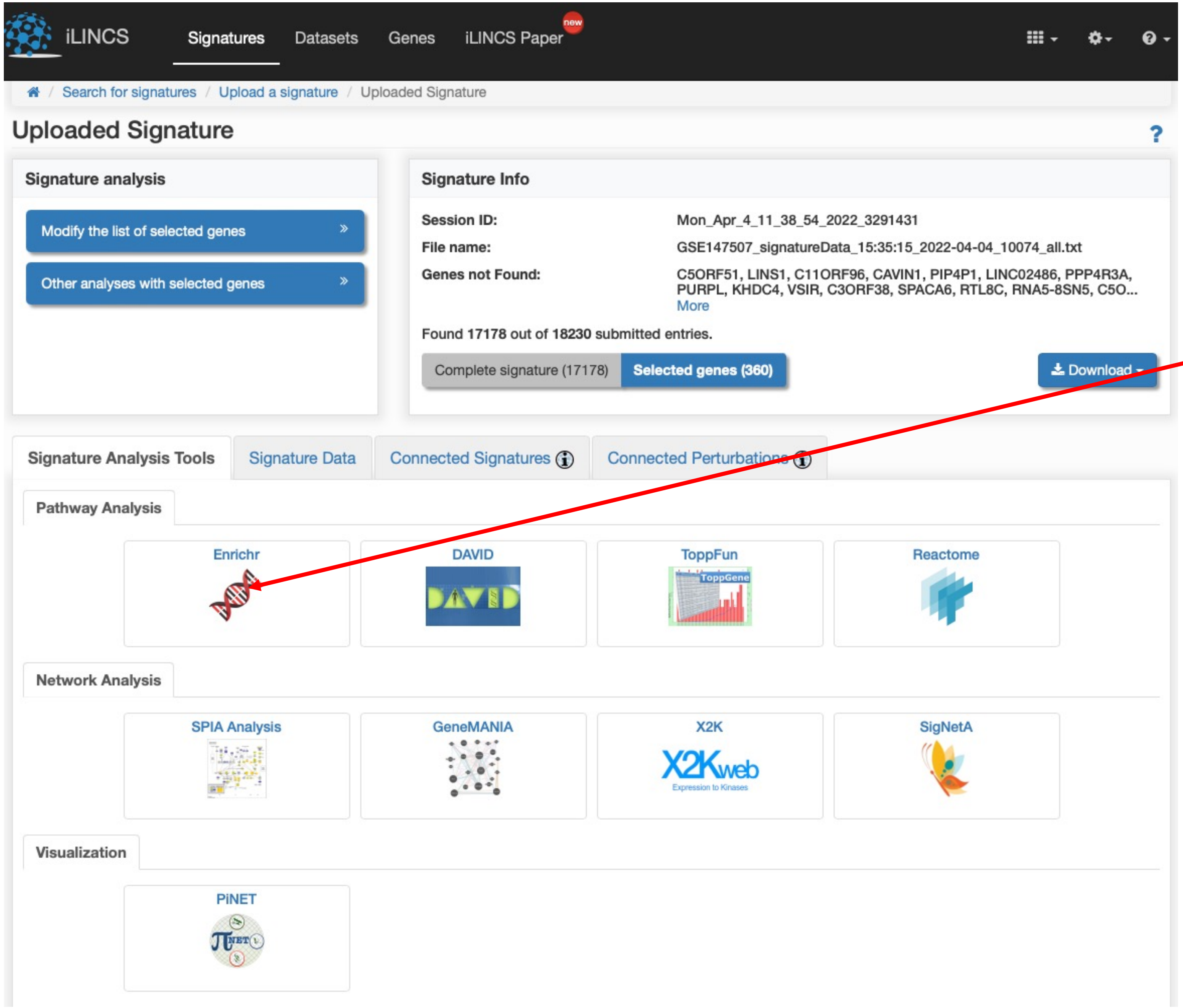

14. Click on Enrich to submit selected upregulated genes for pathway analysis

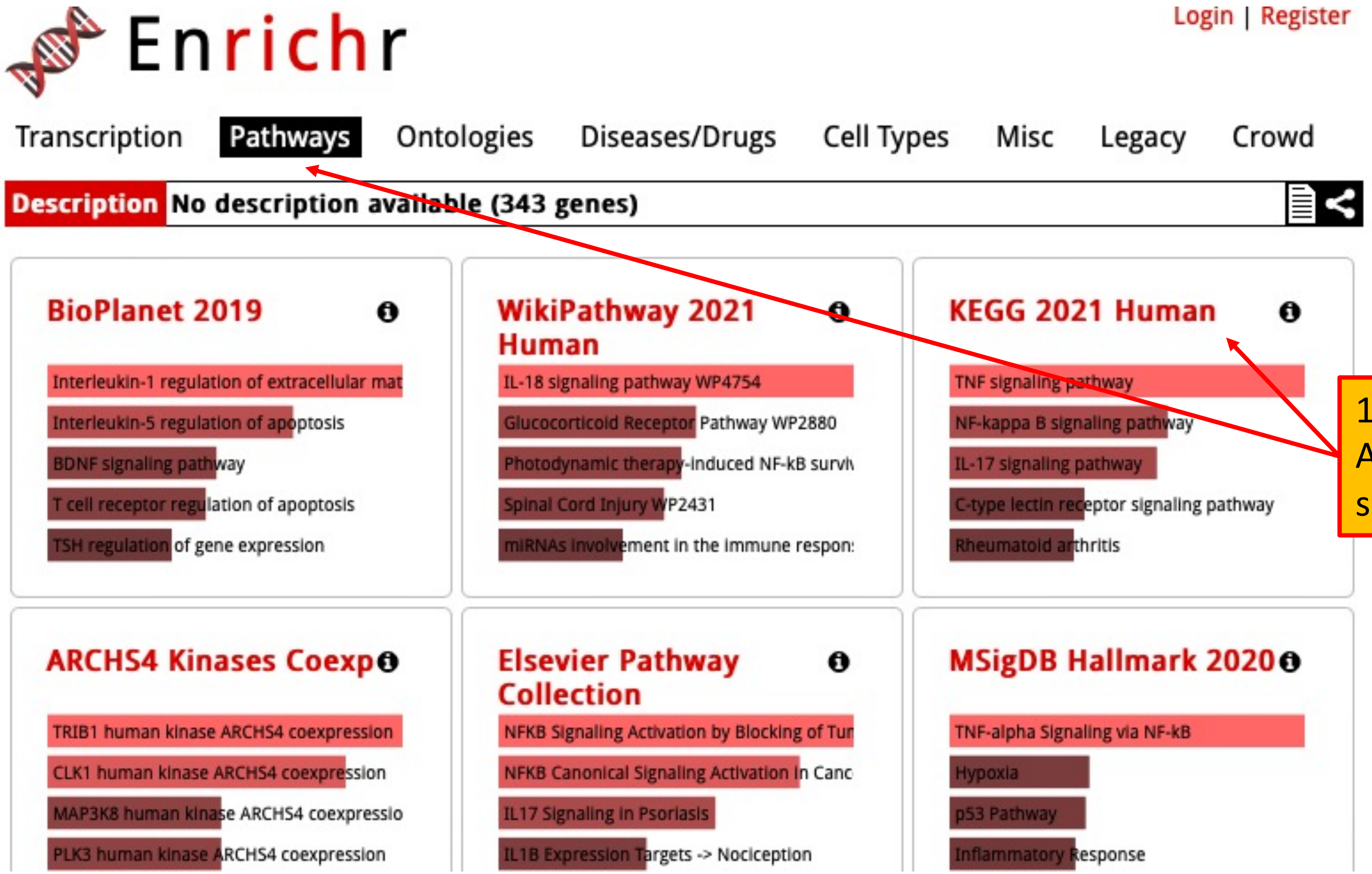

15. Select “Pathways” panel. And observe to most significant KEGG pathways

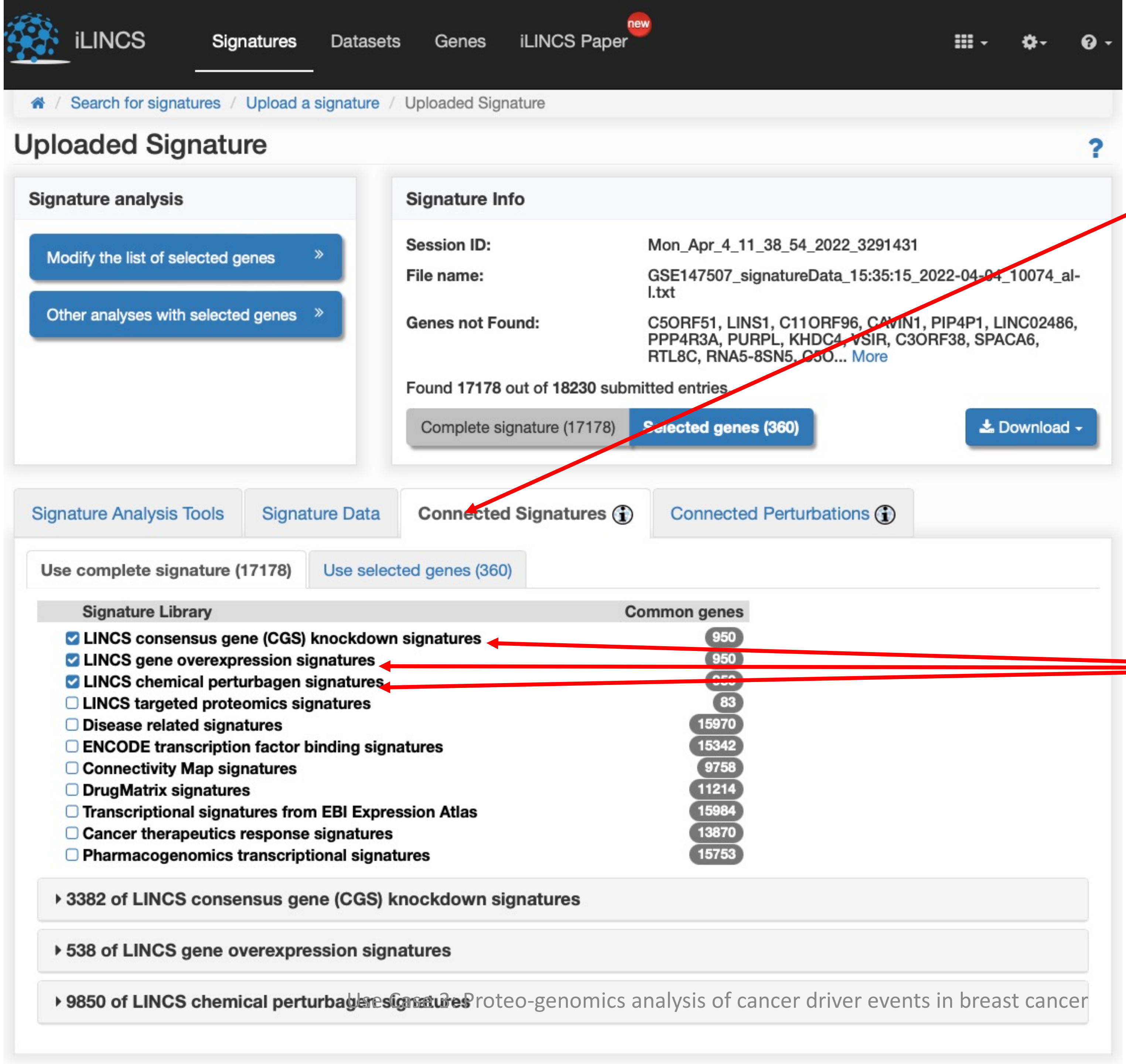

16. Select "Connected Signatures" panels

17. Select three LINCS transcripomic signature libraries for connectivity map analysis

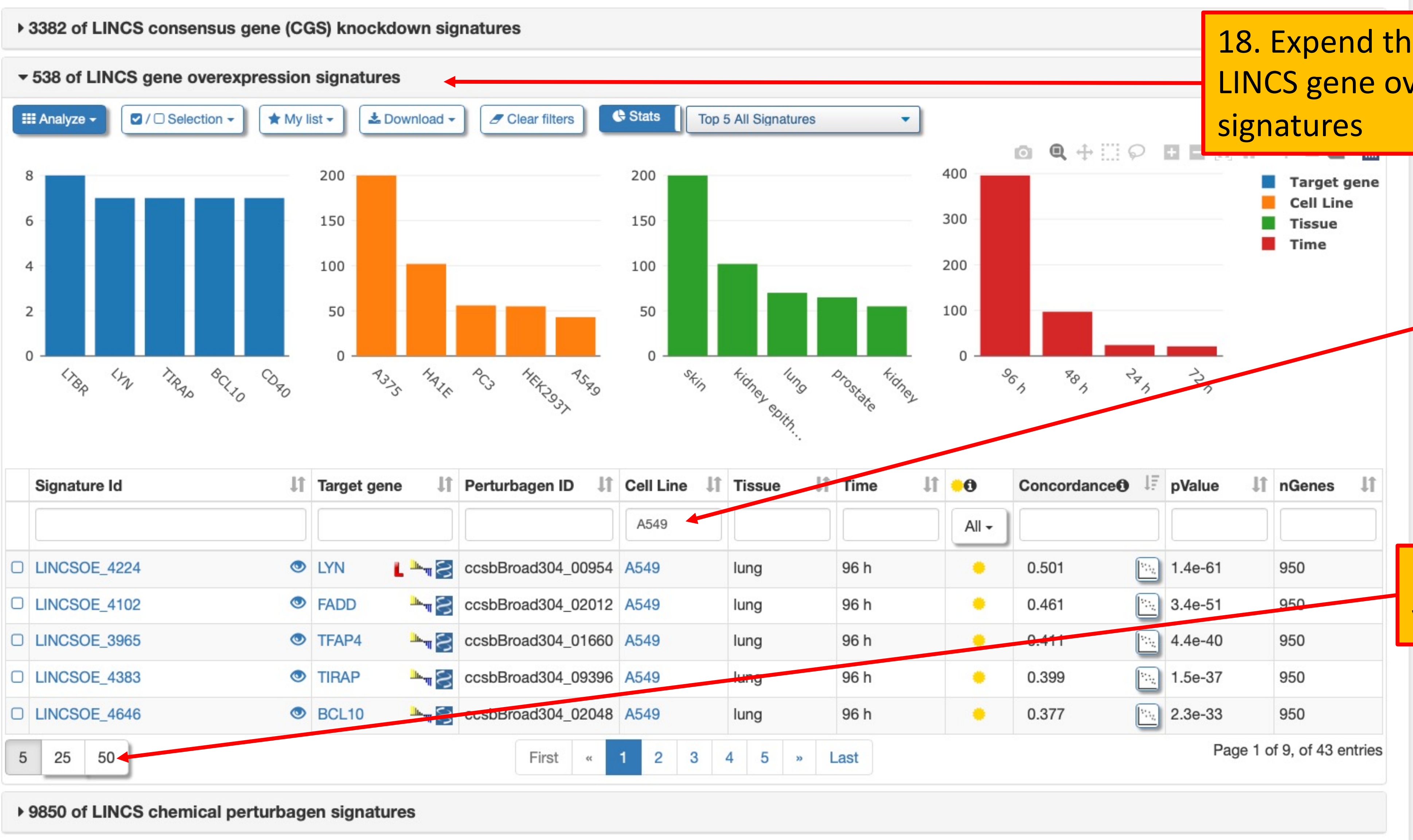

18. Expend the results for LINCS gene overexpression signatures

19. Filter on only A549 signature

20. Expand the list of visible signatures

▸ 3382 of LINCS consensus gene (CGS) knockdown signatures

▾ 538 of LINCS gene overexpression signatures

21. Click on “Select visible” to select all displayed signatures

Analyze ▾ | ☑ / ☐ Selection ▾ | ★ My list ▾ | Download ▾ | Clear filters | Stats | Top 5 All Signatures ▾

- ☑ Select Visible
- ☑ Select First 10 (Select currently displayed signatures)
- ☑ Select First 50
- ☑ Select First 100
- ☑ Select All
- ☐ Unselect All

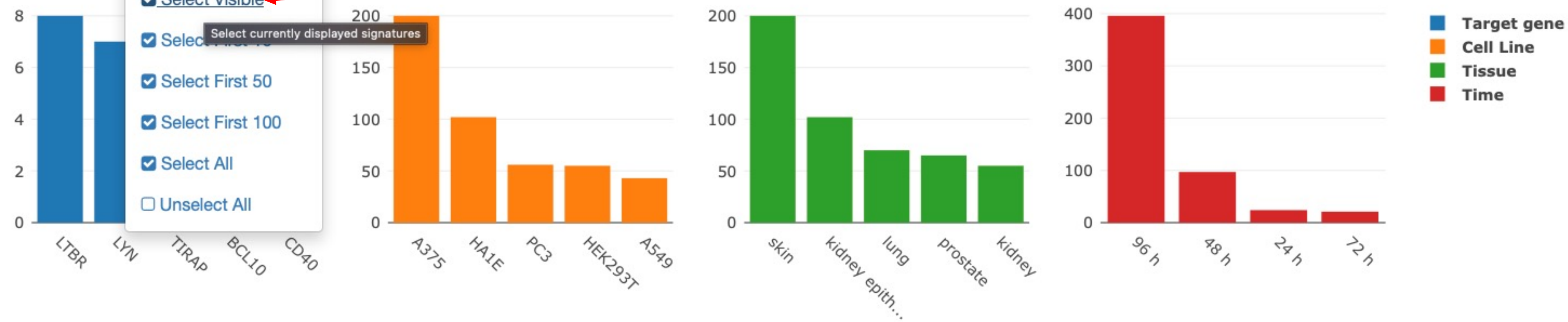

	Signature Id	Target gene	Perturbagen ID	Cell Line	Tissue	Time		Concordance	pValue	nGenes
				A549			All ▾			
☑	LINCSOE_4224	LYN	ccsbBroad304_00954	A549	lung	96 h		0.501	1.4e-61	950
☑	LINCSOE_4102	FADD	ccsbBroad304_02012	A549	lung	96 h		0.461	3.4e-51	950
☑	LINCSOE_3965	TFAP4	ccsbBroad304_01660	A549	lung	96 h		0.411	4.4e-40	950
☑	LINCSOE_4383	TIRAP	ccsbBroad304_09396	A549	lung	96 h		0.399	1.5e-37	950
☑	LINCSOE_4646	BCL10	ccsbBroad304_02048	A549	lung	96 h		0.377	2.3e-33	950
☑	LINCSOE_4707	IFNG	ccsbBroad304_00833	A549	lung	96 h		0.344	9.7e-28	950
☑	LINCSOE_4180	LTBR	ccsbBroad304_06542	A549	lung	96 h		0.344	1.0e-27	950
☑	LINCSOE_3402	OSR2	ccsbBroad304_04691	A549	lung	96 h		0.322	2.3e-24	950
☑	LINCSOE_4785	POU5F1	ccsbBroad304_06753	A549	lung	96 h		0.296	9.9e-21	950
☑	LINCSOE_3982	ACADM	ccsbBroad304_00006	A549	lung	96 h		0.283	6.5e-19	950
☑	LINCSOE_4053	HSPB8	ccsbBroad304_02958	A549	lung	96 h		0.278	2.7e-18	950

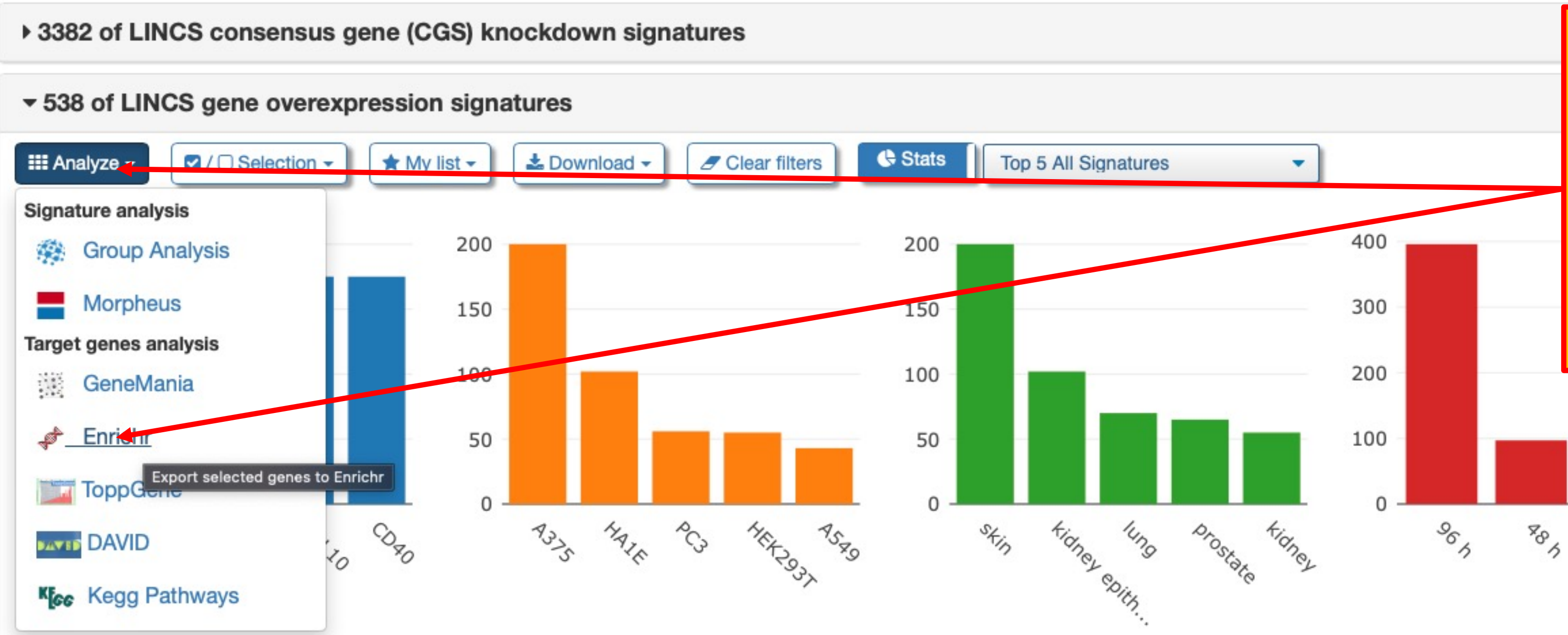

22. Click on "Analyze" drop down list and then click on Enrichr to perform pathway analysis of overexpressed genes corresponding to selected signatures.

	Signature Id		Target gene		Perturbagen ID	Cell Line	Tissue	Time		Concordance		pValue	nGenes
						A549			All				
☑	LINCSOE_4224		LYN		ccsbBroad304_00954	A549	lung	96 h		0.501		1.4e-61	950
☑	LINCSOE_4102		FADD		ccsbBroad304_02012	A549	lung	96 h		0.461		3.4e-51	950
☑	LINCSOE_3965		TFAP4		ccsbBroad304_01660	A549	lung	96 h		0.411		4.4e-40	950
☑	LINCSOE_4383		TIRAP		ccsbBroad304_09396	A549	lung	96 h		0.399		1.5e-37	950
☑	LINCSOE_4646		BCL10		ccsbBroad304_02048	A549	lung	96 h		0.377		2.3e-33	950
☑	LINCSOE_4707		IFNG		ccsbBroad304_00833	A549	lung	96 h		0.344		9.7e-28	950
☑	LINCSOE_4180		LTBR		ccsbBroad304_06542	A549	lung	96 h		0.344		1.0e-27	950
☑	LINCSOE_3402		OSR2		ccsbBroad304_04691	A549	lung	96 h		0.322		2.3e-24	950
☑	LINCSOE_4785		POU5F1		ccsbBroad304_06753	A549	lung	96 h		0.296		9.9e-21	950

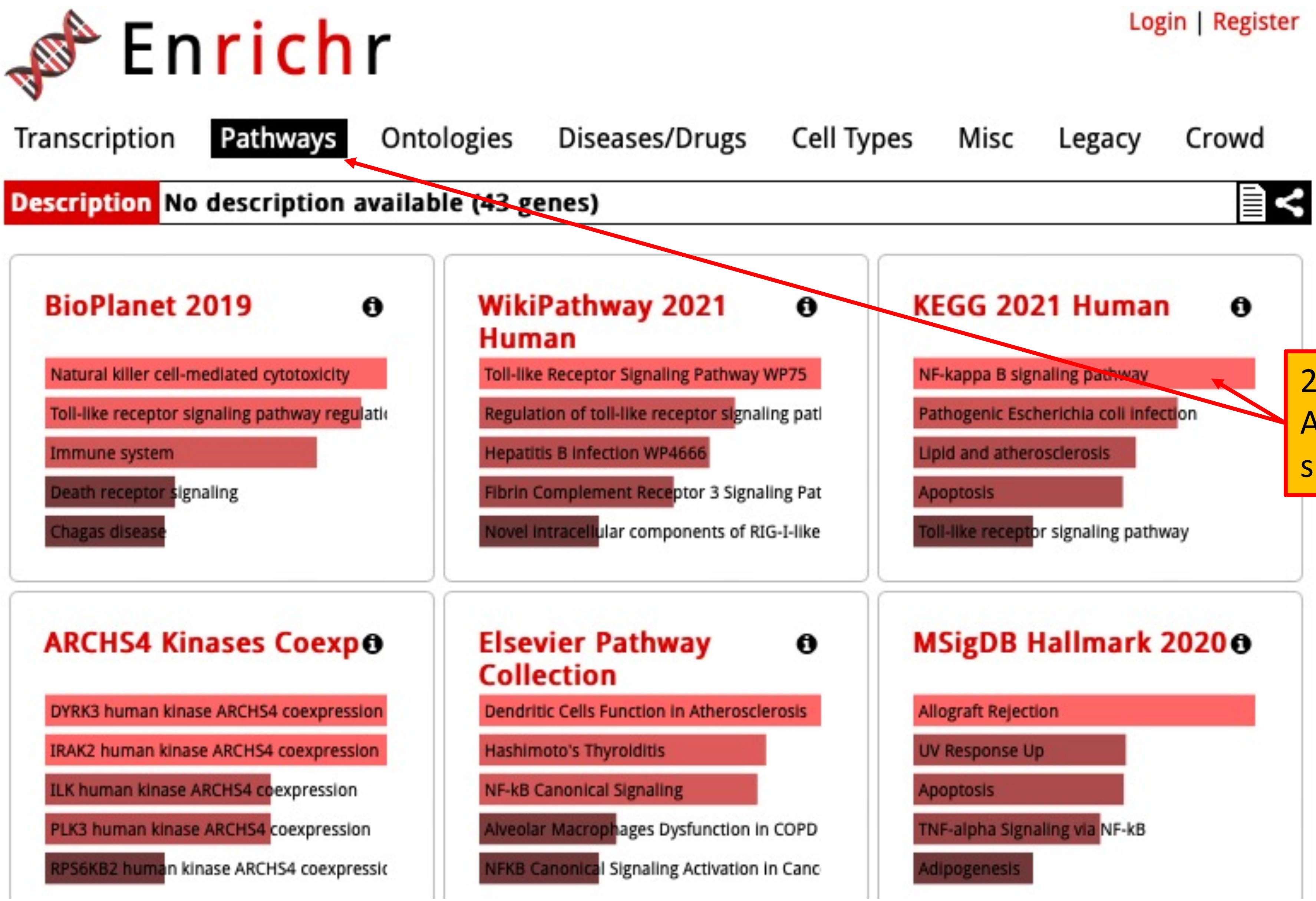

23. Select “Pathways” panel. And observe to most significant KEGG pathway

28. Click to proceed with summaries

▸ 538 of LINCS gene overexpression signatures

▾ 9850 of LINCS chemical perturbagen signatures

Analyze ▾ | ☑ / ☐ Selection ▾ | ★ My list ▾ | Download ▾ | Clear filters | Stats | Top 5 From Selected Signatures ▾

Selection has changed. Please click to calculate the stats again

	Signature Id	Perturbagen	Perturbagen targets	Concentration	Cell Line	Tissue	Time		Concordance	pValue	nGenes
								All ▾			
☑	LINCSCP_131611	CGP 60474	CDK1	3.33uM	MCF10A	breast epithelial	3h		-0.413	2.5e-40	950
☑	LINCSCP_19450	PG 490		0.1uM	HCC515	lung	6h		-0.412	3.3e-40	950
☑	LINCSCP_131493	Alvocidib	CDK1 | CDK2 | ...	0.37uM	MCF10A	breast	3h		-0.397	2.9e-37	950

Final results reported in the paper

▸ 538 of LINCS gene overexpression signatures

▾ 9850 of LINCS chemical perturbagen signatures

Analyze ▾ | ☑ / ☐ Selection ▾ | ★ My list ▾ | Download ▾ | Clear filters | Stats | Top 5 From Selected Signatures ▾

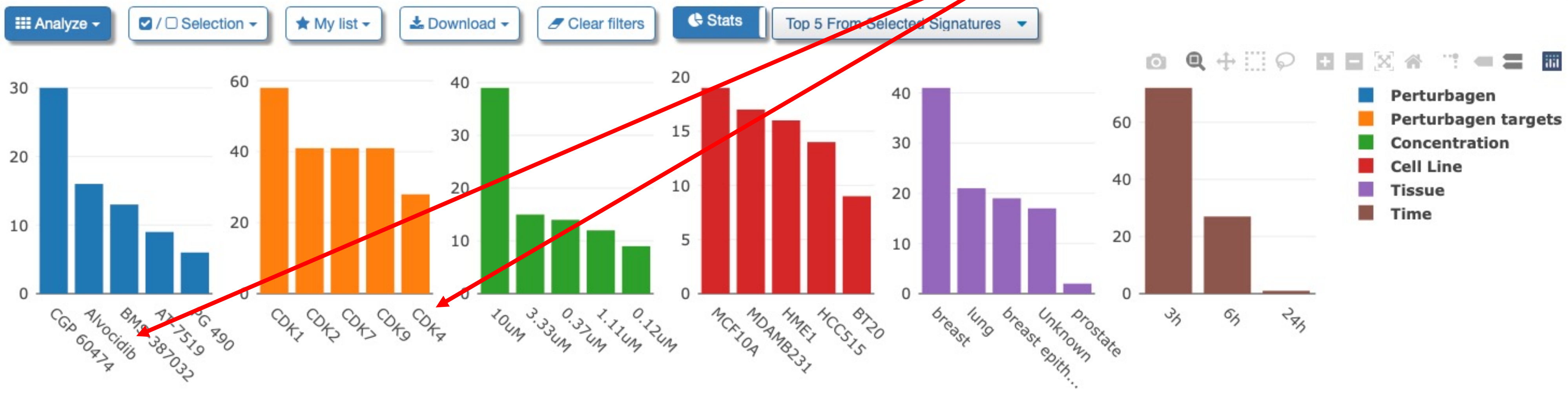

	Signature Id	Perturbagen	Perturbagen targets	Concentration	Cell Line	Tissue	Time		Concordance		pValue	nGenes
								All ▾				
☑	LINCSCP_131611	CGP 60474	CDK1	3.33uM	MCF10A	breast epithelial	3h		-0.413		2.5e-40	950
☑	LINCSCP_19450	PG 490		0.1uM	HCC515	lung	6h		-0.412		3.3e-40	950
☑	LINCSCP_131493	Alvocidib	CDK1 | CDK2 | ...	0.37uM	MCF10A	breast	3h		-0.397		2.9e-37	950
