# Supplementary material for: Connecting omics signatures and revealing biological mechanisms with iLINCS
Source: Nat Commun. 2022 Aug 9;13:4678. doi: 10.1038/s41467-022-32205-3 (PMC9362980; doi:10.1038/s41467-022-32205-3)
Supplement: Supplementary file 2 — Reporting Summary [file 41467_2022_32205_MOESM2_ESM.pdf]

nature portfolio

Corresponding author(s): Mario Medvedovic

Last updated by author(s): Jun 30, 2022

Reporting Summary

Nature Portfolio wishes to improve the reproducibility of the work that we publish. This form provides structure for consistency and transparency in reporting. For further information on Nature Portfolio policies, see our Editorial Policies and the Editorial Policy Checklist.

Statistics

For all statistical analyses, confirm that the following items are present in the figure legend, table legend, main text, or Methods section.

n/a	Confirmed	
☐	☒	The exact sample size (n) for each experimental group/condition, given as a discrete number and unit of measurement
☐	☒	A statement on whether measurements were taken from distinct samples or whether the same sample was measured repeatedly
☐	☒	The statistical test(s) used AND whether they are one- or two-sided<br>*Only common tests should be described solely by name; describe more complex techniques in the Methods section.*
☐	☒	A description of all covariates tested
☐	☒	A description of any assumptions or corrections, such as tests of normality and adjustment for multiple comparisons
☐	☒	A full description of the statistical parameters including central tendency (e.g. means) or other basic estimates (e.g. regression coefficient) AND variation (e.g. standard deviation) or associated estimates of uncertainty (e.g. confidence intervals)
☐	☒	For null hypothesis testing, the test statistic (e.g. F, t, r) with confidence intervals, effect sizes, degrees of freedom and P value noted<br>*Give P values as exact values whenever suitable.*
☒	☐	For Bayesian analysis, information on the choice of priors and Markov chain Monte Carlo settings
☒	☐	For hierarchical and complex designs, identification of the appropriate level for tests and full reporting of outcomes
☐	☒	Estimates of effect sizes (e.g. Cohen's d, Pearson's r), indicating how they were calculated

Our web collection on statistics for biologists contains articles on many of the points above.

Software and code

Policy information about availability of computer code

Data collection	Data collection was performed using R (version 3.6.2) scripts and specialized R packages and software tools for interaction with public data repositories (TCGAlincs version 2.10.5, SRA-toolkit version 2.11.0).
Data analysis	Data analysis was performed using iLINCS GUI and API. The scripts utilizing iLINCS API are provided in the GitHub repository (https://github.com/uc-bd2k/ilincsAPI). The list of all R packages and their versions used by iLINCS analysis engine are provided in the Supplemental Table ST4. The results generated by accessing Enrichr were obtained at several different points in time, most recently on Jun 24, 2022. Each time we performed analysis, we checked and confirmed the consistency of previously generated results. iLINCS deploys the Morpheus 1.0.8.

For manuscripts utilizing custom algorithms or software that are central to the research but not yet described in published literature, software must be made available to editors and reviewers. We strongly encourage code deposition in a community repository (e.g. GitHub). See the Nature Portfolio guidelines for submitting code & software for further information.

Data

Policy information about availability of data

All manuscripts must include a data availability statement. This statement should provide the following information, where applicable:

- Accession codes, unique identifiers, or web links for publicly available datasets
- A description of any restrictions on data availability
- For clinical datasets or third party data, please ensure that the statement adheres to our policy

All data used in the analyses is already in public domain. The datasets can be downloaded from iLINCS, or from the original sources which are provided on the dataset and signature landing pages in iLINCS GUI. The source LINCS L1000 datasets are GSE92742 [https://www.ncbi.nlm.nih.gov/geo/query/acc.cgi?acc=GSE92742] and GSE70138 [https://www.ncbi.nlm.nih.gov/geo/query/acc.cgi?acc=GSE70138], the aging rat rapamycin treatment dataset is GSE108978 [https://

www.ncbi.nlm.nih.gov/geo/query/acc.cgi?acc=GSE108978] and the SARS-Cov-2 infection dataset is GSE147507 [https://www.ncbi.nlm.nih.gov/geo/query/acc.cgi?acc=GSE147507]. The TCGA datasets were downloaded from Genomic Data Commons [https://portal.gdc.cancer.gov/] using the TCGAbiolinks R package [https://bioconductor.org/packages/release/bioc/html/TCGAbiolinks.html]. Supplemental Use Cases describe how to use iLINCS GUI to access all signatures and datasets used in the analyses.

Field-specific reporting

Please select the one below that is the best fit for your research. If you are not sure, read the appropriate sections before making your selection.

[x] Life sciences [ ] Behavioural & social sciences [ ] Ecological, evolutionary & environmental sciences

For a reference copy of the document with all sections, see nature.com/documents/nr-reporting-summary-flat.pdf

Life sciences study design

All studies must disclose on these points even when the disclosure is negative.

Sample size	All data was re-used from the public domain and all available samples for the comparisons made were used in the analysis. No samples size analysis was performed. When contrasting positive and negative results, all sample sizes were comparable indicating that the differences in the statistical significance were due to the effect size and not due to the sample size.
Data exclusions	No data was excluded.
Replication	No new data was created and findings were not experimentally confirmed. Results of the TCGA data analysis were compared the original report, the results of the analysis of LINCS mTOR inhibitors data were verified by performing the analysis of the legacy Connectivity Map signature of sirolimus and in vivo treatment of aging rats, the results in the analysis of SARS Cov 2 infection dataset were cross-referenced with results obtained by other groups in analyzing this and other similar datasets. The steps undertaken to verify reproducibility of the data processing and analysis results produced by iLINCS are detailed in the Supplemental Quality Control document.
Randomization	There was not randomization in the course of the analyses performed. The data that was used has already been generated and randomization at the analysis stage that we could control, would serve no purpose.
Blinding	No blinding was performed in the course of analysis. The data that was used has already been generated and blinding at the analysis stage that we could control, would serve no purpose.

Reporting for specific materials, systems and methods

We require information from authors about some types of materials, experimental systems and methods used in many studies. Here, indicate whether each material, system or method listed is relevant to your study. If you are not sure if a list item applies to your research, read the appropriate section before selecting a response.

Materials & experimental systems

n/a	Involved in the study	
[x]	[ ]	Antibodies
[x]	[ ]	Eukaryotic cell lines
[x]	[ ]	Palaeontology and archaeology
[x]	[ ]	Animals and other organisms
[x]	[ ]	Human research participants
[x]	[ ]	Clinical data
[x]	[ ]	Dual use research of concern

Methods

n/a	Involved in the study	
[x]	[ ]	ChIP-seq
[x]	[ ]	Flow cytometry
[x]	[ ]	MRI-based neuroimaging
