# Supplementary material for: Connecting omics signatures and revealing biological mechanisms with iLINCS
Source: Nat Commun. 2022 Aug 9;13:4678. doi: 10.1038/s41467-022-32205-3 (PMC9362980; doi:10.1038/s41467-022-32205-3)
Supplement: Supplementary file 3 — Description of Additional Supplementary Files [file 41467_2022_32205_MOESM3_ESM.pdf]

Title: Supplementary Data 1:
Description: Results of the CMAP analysis of the Consensus Genes Signature (CGS) of CRISPR mTOR genetic loss of function perturbation in MCF-7 cell line. The CMAP analysis was performed against all nine iLINCS signature libraries. Each spreadsheet in the Excel workbook corresponds to the results for one library.

Title: Supplementary Data 2:
Description: The enriched Hallmark gene sets for genes up-regulated in Luminal A tumors, and genes up-regulated in Her2E tumors.

Title: Supplementary Data 3:
Description: The top 100 connected signatures in the CMAP analysis of the Luminal A vs Her2E RNA-seq signature. The CMAP analysis was run against signatures in five most iLINCS libraries: CP signatures, Disease signatures, Encode signatures, EBI Expression atlas signatures and Cancer Therapeutic Response signatures.

Title: Supplementary Data 4:
Description: The list and versions of all R packages utilized by iLINCS.

Title: Supplementary Software:
Description: The collection of RStudio notebooks demonstrating the use of iLINCS API, and the QC R scripts reproducing the calculations of various connectivity map analysis scores used by iLINCS. All scripts in this collection can also be accessed in GitHub at https://github.com/uc-bd2k/ilincsAPI.
