# Supplementary material for: Connecting omics signatures and revealing biological mechanisms with iLINCS
Source: Nat Commun. 2022 Aug 9;13:4678. doi: 10.1038/s41467-022-32205-3 (PMC9362980; doi:10.1038/s41467-022-32205-3)
Supplement: Supplementary file 8 — Software 1 [file 41467_2022_32205_MOESM8_ESM.zip › ilincsAPI-master/qc/Supplemental Quality Control.pdf]

Connecting omics signatures of diseases, drugs, and mechanisms of actions with iLINCS

Marcin Pilarczyk [1,6§], Michal Kouril [2,6§], Behrouz Shamsaei [1,6§], Juozas Vasiliauskas [1,6], Wen Niu [1,6], Naim Mahi [1,6], Lixia Zhang [1,6] Nicholas Clark [1,6], Yan Ren[1,6], Shana White [1,6], Rashid Karim [1,5,6], Huan Xu[1,6], Jacek Biesiada[1], Mark F. Bennett[1,6], Sarah Davidson[1], John F Reichard [1,7], Kurt Roberts[1], Vasileios Stathias[3,6], Amar Koleti[3,6], Dusica Vidovic[3,6], Daniel J.B. Clarke[4,6], Stephan C. Schurer[3,6], Avi Ma'ayan[4,6], Jarek Meller [1,2,6], Mario Medvedovic [1,6]*

Quality control assessment of iLINCS data and analysis procedures

Table of Contents

Quality Control of Signature Libraries 1

LINCS L1000 signatures 1

Comparison of LINCS signatures hosted by iLINCS to those released by in GEO (GSE92742 and GSE70138).... 2

The accuracy of iLINCS signatures in the backend database and retrieved through iLINCS API 2

Comparison of LINCS signatures hosted by iLINCS to those hosted by clue.io 2

Quality Control of Omics Datasets 2

Microarray datasets 3

RNA-seq datasets 3

The accuracy and the consistence of iLINCS analysis results 4

Accuracy of connectivity analysis.... 4

Accuracy of pre-computed connectivity scores 4

The accuracy and the consistency of connectivity scores calculated for newly created and user-submitted signatures 4

Accuracy of analysis of iLINCS datasets to create a signature 6

Quality Control of Signature Libraries

LINCS L1000 signatures

The vast majority of omics signatures in iLINCS are derived from the LINCS L1000 data. iLINCS signatures were constructed by following the Broad Institute strategy outlined the original publication (details in Methods and Supplemental Methods). The "level 4" z scores data was downloaded from GEO and MODZ "level 5" signatures were calculated as a weighted average across level 4 replicated plates (https://clue.io/connectopedia/data_levels)[1]. In our internal test, connectivity analysis of the same treatments (perturbagen, time, and concentration matched) across different cell lines for level 5 signatures consistently yields the area under the ROC curve of 0.95, or greater (Supplemental Results 2: Benchmarking methods for connectivity analysis and comparisons to other web resources). This indicates that overall precision of LINCS L1000 signatures in iLINCS in correctly connecting biologically similar signatures is very high. Here we perform technical tests of accuracy of the signatures in iLINCS by comparing them to the released Level 5 signatures in GEO. We perform

this comparison by comparing off-line results all signatures produced by our pipeline to signatures released in GEO, and by randomly selecting and downloading 100 signatures via iLINCS API and confirming again that they are identical to the signatures released in GEO. We also perform a direct connectivity analysis of iLICNS with LINCS signatures via Broad clue.io system.

Comparison of LINCS signatures hosted by iLINCS to those released by in GEO (GSE92742 and GSE70138)

To assess the accuracy of our processing and the accuracy of the signatures in the database, we compared iLINCS CP signatures with those released to GEO by the Broad institute. It is important to notice that in our processing we used the latest Broad algorithm to computing the MODZs (https://github.com/cmap/cmapM). As for the datasets released in GEO, a portion of the older dataset (GSE92742) used an older version of the pipeline while the new dataset (GSE70138) used the latest pipeline which is identical to our pipeline. This was reflected in the results of our comparisons. For signatures in the second release (GSE70138), 100% signatures from iLINCS were identical to corresponding Level 5 signatures in GEO. For signatures in the pilot phase data release (GSE92742), 7.5% signatures had the correlation <0.99, which could be considered as slightly different and is consistent with the use of a slightly different processing pipeline.

The accuracy of iLINCS signatures in the backend database and retrieved through iLINCS API

To make sure that the L1000 signatures we computed were accurately represented in the iLINCS backend databases and in the connectivity analysis, we Use API to download signatures from iLINCS and correlate with signatures in the GEO. A random set of 100 signatures was downloaded from iLINCS using APIs and compared to the GEO signatures. The comparison was made using three measures of similarity, Pearson's correlation, total absolute deviation and maximum absolute deviation. If $s_1 = (g_{1,1}, \ldots, g_{978,1})$ and $s_2 = (g_{1,2}, \ldots, g_{978,2})$ are two signatures where $g_{i,k}$ is the MODZ score for jth gene and ith signatures, then the vector of absolute deviation is defined as $\boldsymbol{ad} = (|g_{1,1} - g_{2,1}|, \ldots, |g_{1,978} - g_{2,978}|)$, the total absolute deviation is defined as $adt = \sum_{i=1}^{978} ad_i$ and maximum absolute deviation is defined as $adm = \max(ad_1, \ldots, ad_{978})$. The results for this analysis is provided in the Supplementary QC Table showing perfect correspondence between signatures in iLINCS and signatures released in GEO.

Comparison of LINCS signatures hosted by iLINCS to those hosted by clue.io

To compare LINCS signatures and connectivity methods from iLINCS to clue.io, we selected 100 random iLINCS L1000 CP (chemical perturbagen) signatures and used them to query clue.io. For each iLINCS signature we extracted a list of the 100 most up-regulated and the 100 most down-regulated landmark genes and submitted these gene lists to clue.io via their L1000 query API. We then assessed each signature's "self-connectivity" by the rank of its connectivity score with the corresponding clue.io signature among all clue.io signatures. A rank of 1 indicates that the iLINCS signature was most connected to its corresponding clue.io signature. Of the 100 signatures tested, all except one had virtually perfect association with clue.io signatures (Supplemental QC table). 98 signatures had a perfect "self-connectivity" rank of 1, one (ASG001_PC3_6H:BRD-A19500257-001-04-7:0.08) had rank of 3 (out of >1 million) which is still virtually perfect association. One signature (LJP006_HEPG2_24H:M01) showed poor association with the corresponding clue.io signature. We assessed the quality of this signature in iLINCS by comparing it to the corresponding signature in the released GEO dataset (GSE70138) and verified that iLINCS signature is identical to the GEO signature. Overall, these results indicate that L1000 signatures in iLINCS are accurate and consistent with the L1000 signatures in clue.io.

Quality Control of Omics Datasets

The omics datasets in iLINCS are uploaded by two distinct processes: 1) The batch processing of microarray and RNA-seq data in GEO and 2) By curated processing of individual datasets. The vast majority of the datasets in iLINCS were processed the first, batch processing approach. In the situations when individual datasets are processed and curated (second approach), each such dataset is associated with a unique processing and uploading script, and the data and metadata are inspected and compared with the original source after it is uploaded. Here we report a systematic quality control we performed for more than 11,000 datasets that were processed using batch processing of microarray and RNA-seq datasets. The quality control of these iLINCS datasets was performed by comparing them to results obtained by other

group's independent processing efforts. The QC of microarray GEO GDS datasets data and metadata was performed by comparing them to EBI Expression Atlas[2] and RNA-seq datasets were compared to the recount2 project datasets[3].

Microarray datasets

We assessed systematically the quality and consistency of the GDS datasets in iLINCS by re-creating EBI Expression Atlas signatures[2] using the GDS datasets in iLINCS and iLINCS API. Through semi-automated search and curation, we identified 150 single factor Expression Atlas signatures that could be perfectly matched to a microarray dataset in the iLINCS collection. The signatures, datasets and matching factors and sample labels are provided in the Supplemental QC Table. This allowed us to re-construct automatically the equivalent signatures directly from the microarray datasets using iLICNS API. Each of the newly created signature was submitted for the connectivity analysis against all Expression Atlas signatures in iLINCS and the ability to recover the correct signature was assessed. In all 150 cases, the correct signature was highly connected to the corresponding Expression Atlas signature and the correct signatures was ranked as the number 1, most connected among all 5,646 Expression Atlas signatures in iLINCS. The fact that the signatures created directly from uploaded GDS datasets are perfect match for EBI Expression Atlas signatures which were constructed by curation and independent analysis of the same datasets indicates that both metadata and expression data for corresponding microarray datasets in iLINCS are consistent with the data deposited in GEO and validates our processing and uploading GEO GDS datasets to iLINCS. Furthermore, this results also validates accuracy of the analysis procedures used to create signatures in iLINCS.

RNA-seq datasets

We assessed systematically the consistency of the *GREIN collection* of preprocessed human, mouse and rat RNA-seq data from GEO pre-processed by the GEO RNA-seq Experiments Interactive Navigator (GREIN)[4] by comparing them to datasets pre-processed by the recount2 project [3]. Despite vastly different processing pipelines, the data exhibited very high level of reproducibility across two collections indicating that GREIN collection is accurate representation of the GEO RNA-seq data.

The comparison between GREIN and recount datasets was performed for common human datasets in both collections. Since recount datasets provides quantifications for individual "runs" and aggregating individual runs into sample specific would require additional manipulations, we focus 18,626 samples from 968 GEO datasets which had one run per sample. The consistency of the processed data was assessed by calculating pairwise Pearson's correlations between all sample GREIN profiles with all recount profiles. A pair of GREIN and recount profiles $(\boldsymbol{g}_i, \boldsymbol{r}_j)$ is designated as true positive (TP) if

Fig 1

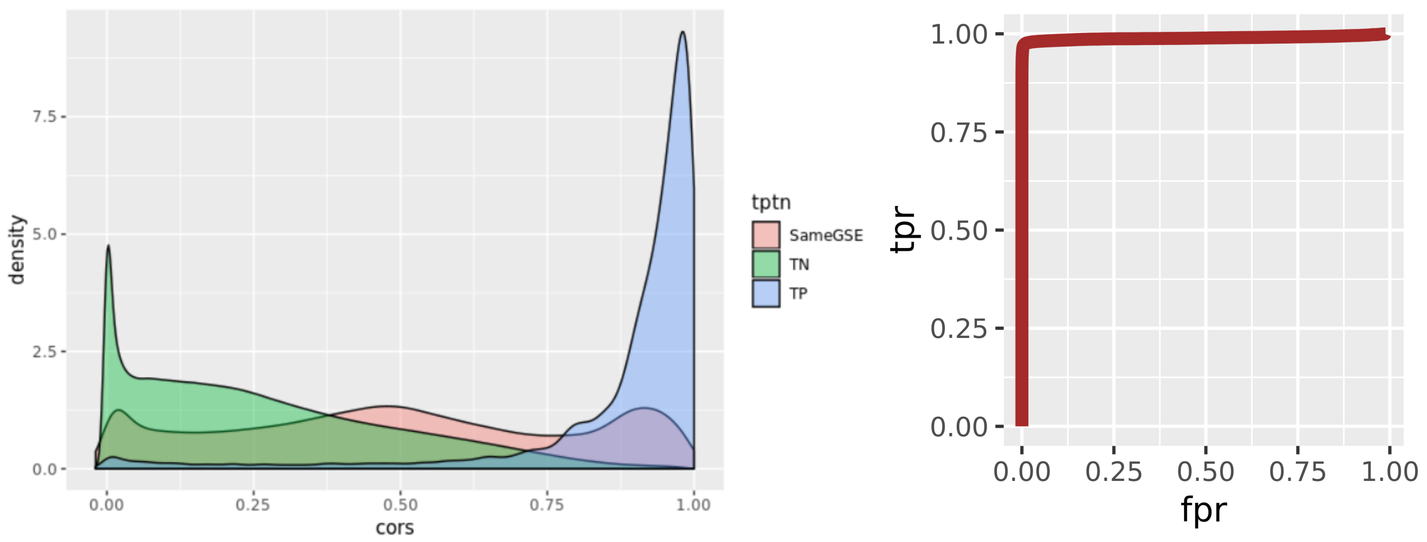

they correspond to the same sample, true negative (TN) if they come from different GEO series, and the pairs corresponding to different samples within the same series as “SameGSE”. The distribution of Person’s correlations is shown in Fig 1. It indicates very strict separation between the correlations between TP and TN samples. This is also clearly indicated in the associated ROC curves for separating TP from TN samples (Fig1 B). False positive rate (fpr) for each TP pair $(\boldsymbol{g}_i, \boldsymbol{r}_i)$ was calculated as the proportion of TN pairs with higher correlation than $cor(\boldsymbol{g}_i, \boldsymbol{r}_i)$. The area under the ROC curve was 0.98 indicating close to perfect separation.

The accuracy and the consistence of iLINCS analysis results

To establish that the accuracy of iLINCS analysis results we implemented the equivalent methodology off-line and cross-references the results produced by iLINCS with results produced by independently implemented methods off-line. We focused on two main aspects of the analyses offered by iLINCS: 1) The connectivity analysis between pre-computed and user submitted signatures; and 2) The methods for creating an omics signature from iLINCS datasets.

Accuracy of connectivity analysis

To systematically and reproducibly assess the accuracy of pre-computed connections between iLINCS signatures and accuracy of the connectivity analysis of newly created and user submitted signatures, we wrote a qulity control (QC) R script (https://github.com/uc-bd2k/ilincsAPI/tree/master/qc) that implements connectivity analysis methods used by iLINCS, performs off-line connectivity analysis, and compares the results to iLINCS results.

Accuracy of pre-computed connectivity scores

The pre-computed connections between all iLINCS signatures are based on the extreme Pearson’s correlation of signed significances (Supplemental Methods). For a randomly selected iLINCS signatures, the QC R script uses iLICNS API to identify connected signatures and the value of the extreme Pearson’s correlation. Then it again uses iLINCS API to download the query and a randomly selected set of connected signatures and calculates extreme Pearson’s correlations off-line. Finally, it compares the correlations returned by the iLINCS query to the off-line calculated correlations. Below is the example output produced by the QC R script for the analysis of iLINCS CP signature LINCSCP_113267 and five randomly selected connected signatures. Results were identical to within what is expected by precision of the data stored in the iLINCS database.

```
[1] For signature: LINCSCP_85346 calculated extreme correlation is:-0.330399839537796 iLINCS query results
is: -0.3304

[1] For signature: LINCSCP_14449 calculated extreme correlation is:-0.308913198637522 iLINCS query results
is: -0.308913

[1] For signature: LINCSCP_85348 calculated extreme correlation is:-0.297887956989956 iLINCS query results
is: -0.297888

[1] For signature: LINCSCP_85349 calculated extreme correlation is:-0.279814638299072 iLINCS query results
is: -0.279815

[1] For signature: LINCSCP_209947 calculated extreme correlation is:-0.277738762299301 iLINCS query results
is: -0.277739
```

The accuracy and the consistency of connectivity scores calculated for newly created and user-submitted signatures

The user-submitted signature can come in the form of a table of log differential gene expressions and pvalues, ($\boldsymbol{d}$,$\boldsymbol{p}$), a table of only log differential gene expressions ($\boldsymbol{d}$), and the lists of up- and down-regulated genes ($\boldsymbol{gl}_{up}$,$\boldsymbol{gl}_{down}$). We used QC R script to submit the same signature (LINCSCP_113267) for the analysis via iLINCS API in these three forms and compared the results produced by iLINCS to off-line computations by QC R script.

*The accuracy of weighted correlations connectivity analysis for a (**d,p**) signature*

If the query signature is created from an iLINCS dataset, or directly uploaded by the user in the form of a table of log differential gene expressions and pvalues, (***d,p***) (Supplemental methods), the connectivity with all iLINCS signatures is calculated as the weighted correlation between the two vectors of log-differential expressions (Supplemental methods Eq 3). For this scenario, we used that same signatures as in previous section (LINCSCP_113267) and submitted it for analysis via iLINCS API as a user submitted signature. The results returned by iLINCS were compared to off-line weighted correlation calculated by QC R script off-line for five randomly selected connected signatures. The weighted correlation coefficient against 5 other signatures is calculated offline and compared with iLINCS results. Results were again identical to within what is expected by precision of the data stored in the iLINCS database.

```
[1] For signature: LINCSCP_85346 calculated weighted correlation is:-0.473201749262842 iLINCS query results is: -0.4732018163

[1] For signature: LINCSCP_85348 calculated weighted correlation is:-0.447326879788148 iLINCS query results is: -0.4473269829

[1] For signature: LINCSCP_14449 calculated weighted correlation is:-0.437788573887979 iLINCS query results is: -0.4377884536

[1] For signature: LINCSCP_11625 calculated weighted correlation is:-0.435372189868698 iLINCS query results is: -0.435372125

[1] For signature: LINCSCP_85349 calculated weighted correlation is:-0.429771138405948 iLINCS query results is: -0.4297711733
```

*The accuracy of weighted correlations connectivity analysis for a (**p**) signature*

If the query signature is directly uploaded by the user in the form of a table of log differential gene expressions (***d***) (Supplemental methods), the connectivity with all iLINCS signatures is calculated as the weighted correlation between the two vectors of log-differential expressions with weights being calculated only using p-values of iLINCS signatures (Supplemental methods). We again used that same signatures as in previous section (LINCSCP_113267) and submitted it for analysis via iLINCS API as a user submitted signature consisting only of log differential expressions. The results returned by iLINCS were compared to off-line weighted correlation calculated by QC R script off-line for five randomly selected connected signatures. The weighted correlation coefficient against 5 other signatures is calculated offline and compared with iLINCS results. Results were again identical to within what is expected by precision of the data stored in the iLINCS database.

```
[1] For signature: LINCSCP_11631 calculated weighted correlation is:-0.507595884030785 iLINCS query results is: -0.5075960534

[1] For signature: LINCSCP_209947 calculated weighted correlation is:-0.500765362989446 iLINCS query results is: -0.5007654212

[1] For signature: LINCSCP_85346 calculated weighted correlation is:-0.495213570689653 iLINCS query results is: -0.4952136282

[1] For signature: LINCSCP_11633 calculated weighted correlation is:-0.490393202955426 iLINCS query results is: -0.4903931816

[1] For signature: LINCSCP_14449 calculated weighted correlation is:-0.481937171739761 iLINCS query results is: -0.4819370293
```

The accuracy of queries with up- and down-regulated gene lists ($\boldsymbol{gl}_{up}, \boldsymbol{gl}_{down}$)

If the query signature is directly uploaded by the user in the form of the lists of up- and down-regulated genes ($\boldsymbol{gl}_{up}, \boldsymbol{gl}_{down}$) (Supplemental methods), the connectivity with all iLINCS signatures is calculated by assigning -1 to down-regulated and +1 to upregulated genes:

$$d_i = \begin{Bmatrix} 1, if\ gene\ i \in \boldsymbol{gl}_{up} \\ 1, if\ gene\ i \in \boldsymbol{gl}_{down} \end{Bmatrix}$$

and calculating weighted Pearson's correlation between such vector and iLINCS signatures in the same way as in the previous section, when user submitted signature ***d*** consisted of only log differential expression levels without p-values. We again used that same signatures as in previous section (LINCSCP_113267), extracted 100 most up- and down-regulated genes and submitted it for analysis via iLINCS API as a user submitted signature. The results returned by iLINCS were compared to off-line weighted correlation calculated by QC R script for five randomly selected connected signatures. Results were again identical to within what is expected by precision of the data stored in the iLINCS database.

```
[1] "For signature: LINCSCP_4770 calculated correlation is:0.594277981689531 iLINCS query results is:
0.5942780464"

[1] "For signature: LINCSCP_19362 calculated correlation is:0.557703722767745 iLINCS query results is:
0.5577038697"

[1] "For signature: LINCSCP_32084 calculated correlation is:0.555662560888544 iLINCS query results is:
0.5556625943"

[1] "For signature: LINCSCP_918 calculated correlation is:0.552018761801338 iLINCS query results is:
0.5520187776"

[1] "For signature: LINCSCP_26496 calculated correlation is:0.542683999823284 iLINCS query results is:
0.5426839885"
```

Accuracy of analysis of iLINCS datasets to create a signature

The accuracy of the differential expression analysis of an iLICNS dataset to create an omics signature is also demonstrated by the results presented in the Microarray datasets qc section. In the process of validating that iLINCS GDS datasets can be used to accurately re-create signatures that were independently created by the EBI Expression Atlas project, we also validated the accuracy of the analysis procedure used by iLINCS to perform the differential expression analysis between

Reference List

1 Subramanian, A. *et al.* A Next Generation Connectivity Map: L1000 Platform and the First 1,000,000 Profiles. *Cell* **171**, 1437-1452 e1417, doi:10.1016/j.cell.2017.10.049 (2017).

2 Papatheodorou, I. *et al.* Expression Atlas: gene and protein expression across multiple studies and organisms. *Nucleic Acids Res* **46**, D246-D251, doi:10.1093/nar/gkx1158 (2018).

3 Collado-Torres, L. *et al.* Reproducible RNA-seq analysis using recount2. *Nat Biotech* **35**, 319-321, doi:10.1038/nbt.3838 (2017).

4 Mahi, N. A., Najafabadi, M. F., Pilarczyk, M., Kouril, M. & Medvedovic, M. GREIN: An Interactive Web Platform for Re-analyzing GEO RNA-seq Data. *Scientific Reports* **9**, 7580, doi:10.1038/s41598-019-43935-8 (2019).
