# Supplementary material for: Connecting omics signatures and revealing biological mechanisms with iLINCS
Source: Nat Commun. 2022 Aug 9;13:4678. doi: 10.1038/s41467-022-32205-3 (PMC9362980; doi:10.1038/s41467-022-32205-3)
Supplement: Supplementary file 8 — Software 1 [file 41467_2022_32205_MOESM8_ESM.zip › ilincsAPI-master/qc/usingIlincsApisExample.html]

iLINCS API R Notebook


# iLINCS API R Notebook

### Prerequisites

### Define a function to download and retreive ilincs signatures

```
get_ilincs_signature <- function(ilincs_signatureId) {
    req <- POST("http://www.ilincs.org/api/ilincsR/downloadSignature", body = list(sigID = paste(ilincs_signatureId), display = FALSE), encode = "json")
    ilincs_sessionId<-unlist(httr::content(req))
    #print(ilincs_sessionId)
    fileUrl=paste("http://www.ilincs.org/tmp/",ilincs_sessionId,".xls",sep="")
    signatureData<-read.table(fileUrl,sep="\t",header=T,stringsAsFactors = F)
    #print(signatureData)
  return(signatureData)
}
```

```
## 
## Attaching package: 'dplyr'
```

```
## The following objects are masked from 'package:stats':
## 
##     filter, lag
```

```
## The following objects are masked from 'package:base':
## 
##     intersect, setdiff, setequal, union
```

```
## Loading required package: Hmisc
```

```
## Loading required package: lattice
```

```
## Loading required package: survival
```

```
## Loading required package: Formula
```

```
## Loading required package: ggplot2
```

```
## 
## Attaching package: 'Hmisc'
```

```
## The following objects are masked from 'package:dplyr':
## 
##     src, summarize
```

```
## The following objects are masked from 'package:base':
## 
##     format.pval, units
```

```
## Loading required package: gdata
```

```
## gdata: read.xls support for 'XLS' (Excel 97-2004) files ENABLED.
```

```
##
```

```
## gdata: read.xls support for 'XLSX' (Excel 2007+) files ENABLED.
```

```
## 
## Attaching package: 'gdata'
```

```
## The following objects are masked from 'package:dplyr':
## 
##     combine, first, last
```

```
## The following object is masked from 'package:stats':
## 
##     nobs
```

```
## The following object is masked from 'package:utils':
## 
##     object.size
```

```
## The following object is masked from 'package:base':
## 
##     startsWith
```

```
## Loading required package: mice
```

```
## 
## Attaching package: 'mice'
```

```
## The following object is masked from 'package:stats':
## 
##     filter
```

```
## The following objects are masked from 'package:base':
## 
##     cbind, rbind
```

```
## Loading required package: data.table
```

```
## 
## Attaching package: 'data.table'
```

```
## The following objects are masked from 'package:gdata':
## 
##     first, last
```

```
## The following objects are masked from 'package:dplyr':
## 
##     between, first, last
```

### Display Signature Libraries

```
apiUrl <- "http://www.ilincs.org/api/SignatureLibraries"
req <- GET(apiUrl)
json <- httr::content(req, as = "text")
ilincs_libraries <- fromJSON(json)
ilincs_libraries[,c("libraryID","libraryName")]
```

#### Select 3 random signature from libraries LIB\_5, LIB\_6 and LIB\_11 and check their connectivity against 5 reported connected signature

```
number_of_random_sample_signatures <- 3
number_of_random_connected_signatures <- 5
```

#### Searching for signatures

```
primes_list <- list("LIB_5", "LIB_6", "LIB_11")

for (p in primes_list) {
  print(p)
 
  ilincs_libId<-p
  apiUrl <- paste("http://www.ilincs.org/api/SignatureMeta/findTermWithSynonyms?library=",ilincs_libId,sep="")
  req <- GET(apiUrl)

  ilincs_result_df<-fromJSON(httr::content(req,type="text"))$data


  print(dim(ilincs_result_df[floor(runif(1000, min=0, max=dim(ilincs_result_df)[1])),c("cellline","compound","concentration","signatureid","is_exemplar")]))
  counter = 0
  
  for (sigloop in ilincs_result_df[floor(runif(number_of_random_sample_signatures, min=0, max=dim(ilincs_result_df)[1])),c("signatureid")]){
    ilincs_signatureId = sigloop
    print(ilincs_signatureId)
    counter <- counter + 1
    
    print("download signature ======================================")
    req <- POST("http://www.ilincs.org/api/ilincsR/downloadSignature", body = list(sigID = paste(ilincs_signatureId), display = FALSE), encode = "json")
    ilincs_sessionId<-unlist(httr::content(req))
    
    fileUrl=paste("http://www.ilincs.org/tmp/",ilincs_sessionId,".xls",sep="")
    signatureData <-read.table(fileUrl,sep="\t",header=T,stringsAsFactors = F)
  
    print("=============================================")
    print("get connected signatures based on signatureid")
    print("=============================================")
    apiUrl <- paste("http://www.ilincs.org/api/SignatureMeta/findConcordantSignatures?sigID=",ilincs_signatureId,"&lib=",ilincs_libId,sep="")
    req <- GET(apiUrl)
    
    ilincs_conn_df<-fromJSON(httr::content(req,type="text"))
    result_of_connected_sigs_from_sigID <- ilincs_conn_df[,c("signatureid","similarity","pValue")]
    result_of_connected_sigs_from_sigID<-result_of_connected_sigs_from_sigID[order(result_of_connected_sigs_from_sigID$similarity),]
    print(head(result_of_connected_sigs_from_sigID))
  
    
    print("========================================================================")
    print("get connected signatures based on the downloaded signature with p value") 
    print("========================================================================")
    #print(ilincs_sessionId[1])
    #print(toString(ilincs_sessionId[1]))
    
    file_name <- paste(ilincs_libId,"_", toString(ilincs_sessionId[1]), ".tsv", sep="")
    write.table(signatureData,file=file_name,sep="\t",row.names=F,col.names = T,quote=F)
    #system(paste("head sigFile.tsv"))
    
    
    apiUrl<-"http://www.ilincs.org/api/SignatureMeta/upload"
    sigFile <- file_name
    in_data<-as.data.frame(fread(file_name))
    in_data_orig<-as.data.frame(fread(file_name))
    #print(in_data)
   
    req <- POST(apiUrl, body=list(file=upload_file(sigFile)))
    #req <- POST(apiUrl, body=list(file=signatureData))
    signatureFile <- httr::content(req)$status$fileName[[1]]
    print(head(signatureFile))

    apiUrl <- "http://www.ilincs.org/api/ilincsR/findConcordances"
    req <- (POST(apiUrl, body = list(file=signatureFile, lib=ilincs_libId), encode = "form"))
    output <- data.table::rbindlist(httr::content(req)$concordanceTable, use.names = TRUE, fill = TRUE)
    result_of_upload_complete_sig <- output[,c("signatureid","similarity","pValue")]
    result_of_upload_complete_sig<-result_of_upload_complete_sig[order(result_of_upload_complete_sig$similarity),]
    print(head(result_of_upload_complete_sig))
    
     #merge(authors, books, by.x="surname", by.y="name")
    #for (sigloop in ilincs_result_df[
    print("===========================================================================================================")
    print("checking the accuracy of results via offline benchmarking on the downloaded complete signature with p-value")  
    print("===========================================================================================================")
      for(varIter in 1:number_of_random_connected_signatures)
         {
          #print(varIter)
          compared_sig = result_of_upload_complete_sig[varIter,"signatureid"]
          #print(compared_sig)
          restart_data <- in_data
          #print("--------------------")
          
          #vardiff <- get_ilincs_signature(toString(compared_sig))
          #varpval <- as.data.frame(lincscpPValues[, toString(compared_sig)])
          #if (ilincs_libId == "LIB_5"){
            #print(as.data.frame(lincscpDiffExp[, "LINCSCP_159326"]))
           # vardiff <- as.data.frame(lincscpDiffExp[, toString(compared_sig)])
          #  varpval <- as.data.frame(lincscpPValues[, toString(compared_sig)])
          #}
         #if (ilincs_libId == "LIB_6"){
          # vardiff <- as.data.frame(lincskdDiffExp[,toString(compared_sig)])
           # varpval <- as.data.frame(lincskdPValues[,toString(compared_sig)])
            
         #}
         #if (ilincs_libId == "LIB_11"){
          # vardiff <- as.data.frame(lincsoeDiffExp[,toString(compared_sig)])
           # varpval <- as.data.frame(lincsoePValues[,toString(compared_sig)])
            
      #}
          com_sig <-get_ilincs_signature(toString(compared_sig))
          rownames(com_sig) <- com_sig$ID_geneid
          vardiff <- as.data.frame(com_sig[,c("Value_LogDiffExp")])
          rownames(vardiff) <- com_sig$ID_geneid
          varpval <- as.data.frame(com_sig[,c("Significance_pvalue")])
          rownames(varpval) <- com_sig$ID_geneid
                   
          colnames(vardiff)<- c("compared_sig_dif")
          colnames(varpval)<- c("compared_sig_pval")

          restart_data<-merge(restart_data, vardiff, by.x="ID_geneid", by.y="row.names")
          restart_data<-merge(restart_data, varpval, by.x="ID_geneid", by.y="row.names")
          weight_vec <- c()
          restart_data$"new_weight"<-(-1.0)*(log10(restart_data$"Significance_pvalue")+log10(restart_data$"compared_sig_pval"))
           w_co <- weightedCorr(y=restart_data$"compared_sig_dif", x=restart_data$"Value_LogDiffExp", method="pearson", weights=restart_data$"new_weight")
           
          print(paste0("For signature: ", compared_sig, " calculated weighted correlation is:",w_co, " ilincs query results is: ", result_of_upload_complete_sig[varIter,"similarity"]))

      }
    
    
    print("==========================================================================")
    print("get connected signatures based on the downloaded signature without p value") 
    print("==========================================================================")
    
    
    file_name <- paste(ilincs_libId,"_withot_pvalue_", toString(ilincs_sessionId[1]), ".tsv", sep="")
    
    signatureData_without_pvalue = subset(signatureData, select = -c(Significance_pvalue) )
    write.table(signatureData_without_pvalue,file=file_name,sep="\t",row.names=F,col.names = T,quote=F)
    
    apiUrl<-"http://www.ilincs.org/api/SignatureMeta/upload"
    sigFile <- file_name
    in_data<-as.data.frame(fread(file_name))
    
   
    req <- POST(apiUrl, body=list(file=upload_file(sigFile)))
    
    signatureFile <- httr::content(req)$status$fileName[[1]]
    print(head(signatureFile))

    apiUrl <- "http://www.ilincs.org/api/ilincsR/findConcordances"
    req <- (POST(apiUrl, body = list(file=signatureFile, lib=ilincs_libId), encode = "form"))
    output <- data.table::rbindlist(httr::content(req)$concordanceTable, use.names = TRUE, fill = TRUE)
    result_of_upload_complete_sig <- output[,c("signatureid","similarity","pValue")]
    result_of_upload_complete_sig<-result_of_upload_complete_sig[order(result_of_upload_complete_sig$similarity),]
    print(head(result_of_upload_complete_sig))
    
    
    print("==============================================================================================================")
    print("checking the accuracy of results via offline benchmarking on the downloaded complete signature without p-value")  
    print("==============================================================================================================")
      for(varIter in 1:5)
         {
          
          compared_sig = result_of_upload_complete_sig[varIter,"signatureid"]
          
          restart_data <- in_data
          
          
          com_sig <-get_ilincs_signature(toString(compared_sig))
          rownames(com_sig) <- com_sig$ID_geneid
          vardiff <- as.data.frame(com_sig[,c("Value_LogDiffExp")])
          rownames(vardiff) <- com_sig$ID_geneid
          varpval <- as.data.frame(com_sig[,c("Significance_pvalue")])
          rownames(varpval) <- com_sig$ID_geneid
         
          colnames(vardiff)<- c("compared_sig_dif")
          colnames(varpval)<- c("compared_sig_pval")

          restart_data<-merge(restart_data, vardiff, by.x="ID_geneid", by.y="row.names")
          restart_data<-merge(restart_data, varpval, by.x="ID_geneid", by.y="row.names")
          weight_vec <- c()
          restart_data$"new_weight"<-(-1.0)*(log10(restart_data$"compared_sig_pval"))
          w_co <- weightedCorr(y=restart_data$"compared_sig_dif", x=restart_data$"Value_LogDiffExp", method="pearson", weights=restart_data$"new_weight")
           
          print(paste0("For signature: ", compared_sig, " calculated weighted correlation is:",w_co, " ilincs query results is: ", result_of_upload_complete_sig[varIter,"similarity"]))

      }
    
    print("========================================================================================================")
    print("checking the accuracy of extreme correlation results via offline benchmarking on the submitted signature")
    print("========================================================================================================")
    print(head(result_of_connected_sigs_from_sigID))
      for(varIter in 1:5)
         {
          
          compared_sig = result_of_connected_sigs_from_sigID[varIter,"signatureid"]
          
          restart_data <- in_data_orig
          
      
          com_sig <-get_ilincs_signature(toString(compared_sig))
          rownames(com_sig) <- com_sig$ID_geneid
          vardiff <- as.data.frame(com_sig[,c("Value_LogDiffExp")])
          rownames(vardiff) <- com_sig$ID_geneid
          varpval <- as.data.frame(com_sig[,c("Significance_pvalue")])
          rownames(varpval) <- com_sig$ID_geneid
          
          colnames(vardiff)<- c("compared_sig_dif")
          colnames(varpval)<- c("compared_sig_pval")
          
          v1<-(-1.0)*sign(vardiff)*log10(varpval)
         
          colnames(v1)<- c("v1")
          rownames(v1) <- as.list(com_sig[,"ID_geneid"])
        
          v2<- as.data.frame((-1.0)*sign(restart_data[,"Value_LogDiffExp"])*log10(restart_data[,"Significance_pvalue"]))
          rownames(v2) <- as.list(restart_data[,"ID_geneid"])
          colnames(v2)<- c("v2")
         

          top100_1<-v1 %>% top_n(100)
          top100_2<-v2 %>% top_n(100)
          
          bot100_1<-v1 %>% top_n(-100)
          bot100_2<-v2 %>% top_n(-100)
         
          row_list_1<-rownames(top100_1)
          row_list_2<-rownames(top100_2)
     
          row_list_1<-append(row_list_1,rownames(bot100_1))
          row_list_2<-append(row_list_2,rownames(bot100_2))
          for (p in rownames(v1)) {
            if (!(p %in% row_list_1)){
              
              v1[p,"v1"] <- 0.0
            }
          }
        
          
          for (p in rownames(v2)) {
           
            if (!(p %in% row_list_2)){
             
           
              v2[p,"v2"] <- 0.0
            
            }
          }

          restart_data<-merge(restart_data, v1, by.x="ID_geneid", by.y="row.names")
          
          
          restart_data<-merge(restart_data, v2, by.x="ID_geneid", by.y="row.names")
          
      
           w_co <- weightedCorr(x=restart_data$"v1", y=restart_data$"v2", method="Pearson")
          
          
          print(paste0("For signature: ", compared_sig, " calculated extreme correlation is:",w_co, " ilincs query results is: ", result_of_connected_sigs_from_sigID[varIter,"similarity"]))
      }
    print("=================================================================")
    print("get connected signatures based on positive and negative gene list") 
    print("=================================================================")
    
    top100signature <- signatureData[order(signatureData$Significance_pvalue)[1:100],]
    
    apiUrl="http://www.ilincs.org/api/ilincsR/findConcordancesSC"

    topUpRegulatedGenes <- list(genesUp=top100signature$Name_GeneSymbol[top100signature$Value_LogDiffExp > 0])
    topDownregulatedGenes <- list(genesDown=top100signature$Name_GeneSymbol[top100signature$Value_LogDiffExp < 0])
    topUpRegulatedGeneIDs <- list(top100signature$ID_geneid[top100signature$Value_LogDiffExp > 0])
    topDownregulatedGeneIDs <- list(top100signature$ID_geneid[top100signature$Value_LogDiffExp < 0])
   

    req <- POST("http://www.ilincs.org/api/ilincsR/findConcordancesSC", body = list(mode="UpDn",metadata=TRUE,signatureProfile = c(topUpRegulatedGenes, topDownregulatedGenes)),encode = "json")

    ilincsUpDnConnectedSignatures <- data.table::rbindlist(httr::content(req)$concordanceTable, use.names = TRUE, fill = TRUE)
  
    print("=====================================================================================================")
    print("checking the accuracy of positive and negative gene list correlation results via offline benchmarking")
    print("=====================================================================================================")
    
    if(TRUE){
      for(varIter in 1:5)
         {
      
          compared_sig = ilincsUpDnConnectedSignatures[varIter,"signatureID"]
         
            com_sig <-get_ilincs_signature(toString(compared_sig))
          rownames(com_sig) <- com_sig$ID_geneid
          vardiff <- as.data.frame(com_sig[,c("Value_LogDiffExp")])
          rownames(vardiff) <- com_sig$ID_geneid
          varpval <- as.data.frame(com_sig[,c("Significance_pvalue")])
          rownames(varpval) <- com_sig$ID_geneid
          
          colnames(vardiff)<- c("compared_sig_dif")
          colnames(varpval)<- c("compared_sig_pval")
      
          v1<-vardiff
         
          colnames(v1)<- c("v1")
          
          v2 <- v1
          colnames(v2)<- c("v2")
          
        
          
          v3 <- data.frame(matrix(ncol=1,nrow=0, dimnames=list(NULL, c("v3"))))
          v4 <- data.frame(matrix(ncol=1,nrow=0, dimnames=list(NULL, c("v4"))))
          
          vvv <- data.frame(matrix(ncol=2,nrow=0, dimnames=list(NULL, c("xx", "yy"))))

          for (p in rownames(v1)) {
            
            if ((p %in% unlist(topUpRegulatedGeneIDs, recursive = FALSE))){
              
              new_row <- c(1.0, v1[p,"v1"]) 
              vvv <- rbind(vvv, new_row)
            
            }
            else if ((p %in% unlist(topDownregulatedGeneIDs, recursive = FALSE))){
              
              new_row <- c(-1.0, v1[p,"v1"]) 
              vvv <- rbind(vvv, new_row)
              
             
            }
           
            
          }
         
          res <- cor(vvv)

         
          print(paste0("For signature: ", compared_sig, " calculated correlation is:",unlist(res[1,2]), " ilincs query results is: ", ilincsUpDnConnectedSignatures[varIter,"similarity"]))
          
      }
    }
  
        
    print("++++++++++++++++++++++ Finished ++++++++++++++++++++++")


  }
}
```

```
## [1] "LIB_5"
```

```
## No encoding supplied: defaulting to UTF-8.
```

```
## [1] 1000    5
## [1] "LINCSCP_113267"
## [1] "download signature ======================================"
## [1] "============================================="
## [1] "get connected signatures based on signatureid"
## [1] "============================================="
```

```
## No encoding supplied: defaulting to UTF-8.
```

```
##        signatureid similarity       pValue
## 275  LINCSCP_85346  -0.330400 1.695648e-31
## 490  LINCSCP_14449  -0.308913 3.170204e-28
## 609  LINCSCP_85348  -0.297888 1.197353e-26
## 882  LINCSCP_85349  -0.279815 3.305750e-24
## 910 LINCSCP_209947  -0.277739 6.143123e-24
## 911  LINCSCP_85820  -0.277652 6.303552e-24
## [1] "========================================================================"
## [1] "get connected signatures based on the downloaded signature with p value"
## [1] "========================================================================"
## [1] "processedSig_Thu_Jan_28_11_49_52_2021_7748992.xls"
##       signatureid similarity       pValue
## 1:  LINCSCP_85346 -0.4732018 1.011372e-55
## 2:  LINCSCP_85348 -0.4473270 2.727487e-49
## 3:  LINCSCP_14449 -0.4377885 4.679781e-47
## 4:  LINCSCP_11625 -0.4353721 1.679015e-46
## 5:  LINCSCP_85349 -0.4297712 3.118833e-45
## 6: LINCSCP_209947 -0.4293959 3.785805e-45
## [1] "==========================================================================================================="
## [1] "checking the accuracy of results via offline benchmarking on the downloaded complete signature with p-value"
## [1] "==========================================================================================================="
## [1] "For signature: LINCSCP_85346 calculated weighted correlation is:-0.473201749262842 ilincs query results is: -0.4732018163"
## [1] "For signature: LINCSCP_85348 calculated weighted correlation is:-0.447326879788148 ilincs query results is: -0.4473269829"
## [1] "For signature: LINCSCP_14449 calculated weighted correlation is:-0.437788573887979 ilincs query results is: -0.4377884536"
## [1] "For signature: LINCSCP_11625 calculated weighted correlation is:-0.435372189868698 ilincs query results is: -0.435372125"
## [1] "For signature: LINCSCP_85349 calculated weighted correlation is:-0.429771138405948 ilincs query results is: -0.4297711733"
## [1] "=========================================================================="
## [1] "get connected signatures based on the downloaded signature without p value"
## [1] "=========================================================================="
## [1] "processedSig_Thu_Jan_28_11_50_30_2021_1653160.xls"
##       signatureid similarity       pValue
## 1:  LINCSCP_11631 -0.5075961 3.599945e-65
## 2: LINCSCP_209947 -0.5007654 3.307346e-63
## 3:  LINCSCP_85346 -0.4952136 1.209647e-61
## 4:  LINCSCP_11633 -0.4903932 2.610825e-60
## 5:  LINCSCP_14449 -0.4819370 5.084650e-58
## 6:  LINCSCP_85349 -0.4700264 6.670729e-55
## [1] "=============================================================================================================="
## [1] "checking the accuracy of results via offline benchmarking on the downloaded complete signature without p-value"
## [1] "=============================================================================================================="
## [1] "For signature: LINCSCP_11631 calculated weighted correlation is:-0.507595884030785 ilincs query results is: -0.5075960534"
## [1] "For signature: LINCSCP_209947 calculated weighted correlation is:-0.500765362989446 ilincs query results is: -0.5007654212"
## [1] "For signature: LINCSCP_85346 calculated weighted correlation is:-0.495213570689653 ilincs query results is: -0.4952136282"
## [1] "For signature: LINCSCP_11633 calculated weighted correlation is:-0.490393202955426 ilincs query results is: -0.4903931816"
## [1] "For signature: LINCSCP_14449 calculated weighted correlation is:-0.481937171739761 ilincs query results is: -0.4819370293"
## [1] "========================================================================================================"
## [1] "checking the accuracy of extreme correlation results via offline benchmarking on the submitted signature"
## [1] "========================================================================================================"
##        signatureid similarity       pValue
## 275  LINCSCP_85346  -0.330400 1.695648e-31
## 490  LINCSCP_14449  -0.308913 3.170204e-28
## 609  LINCSCP_85348  -0.297888 1.197353e-26
## 882  LINCSCP_85349  -0.279815 3.305750e-24
## 910 LINCSCP_209947  -0.277739 6.143123e-24
## 911  LINCSCP_85820  -0.277652 6.303552e-24
```

```
## Selecting by v1
```

```
## Selecting by v2
```

```
## Selecting by v1
```

```
## Selecting by v2
```

```
## [1] "For signature: LINCSCP_85346 calculated extreme correlation is:-0.330399839537796 ilincs query results is: -0.3304"
```

```
## Selecting by v1
## Selecting by v2
```

```
## Selecting by v1
```

```
## Selecting by v2
```

```
## [1] "For signature: LINCSCP_14449 calculated extreme correlation is:-0.308913198637522 ilincs query results is: -0.308913"
```

```
## Selecting by v1
## Selecting by v2
```

```
## Selecting by v1
```

```
## Selecting by v2
```

```
## [1] "For signature: LINCSCP_85348 calculated extreme correlation is:-0.297887956989956 ilincs query results is: -0.297888"
```

```
## Selecting by v1
## Selecting by v2
```

```
## Selecting by v1
```

```
## Selecting by v2
```

```
## [1] "For signature: LINCSCP_85349 calculated extreme correlation is:-0.279814638299072 ilincs query results is: -0.279815"
```

```
## Selecting by v1
## Selecting by v2
```

```
## Selecting by v1
```

```
## Selecting by v2
```

```
## [1] "For signature: LINCSCP_209947 calculated extreme correlation is:-0.277738762299301 ilincs query results is: -0.277739"
## [1] "================================================================="
## [1] "get connected signatures based on positive and negative gene list"
## [1] "================================================================="
## [1] "====================================================================================================="
## [1] "checking the accuracy of positive and negative gene list correlation results via offline benchmarking"
## [1] "====================================================================================================="
## [1] "For signature: LINCSCP_113267 calculated correlation is:0.945732977613948 ilincs query results is: 0.9457329492"
## [1] "For signature: LINCSCP_113269 calculated correlation is:0.924633085725055 ilincs query results is: 0.924633092"
## [1] "For signature: LINCSCP_113266 calculated correlation is:0.915208788067983 ilincs query results is: 0.915208743"
## [1] "For signature: LINCSCP_113268 calculated correlation is:0.896636580393445 ilincs query results is: 0.8966365792"
## [1] "For signature: LINCSCP_113270 calculated correlation is:0.896432905003753 ilincs query results is: 0.8964329023"
## [1] "++++++++++++++++++++++ Finished ++++++++++++++++++++++"
## [1] "LINCSCP_110331"
## [1] "download signature ======================================"
## [1] "============================================="
## [1] "get connected signatures based on signatureid"
## [1] "============================================="
```

```
## No encoding supplied: defaulting to UTF-8.
```

```
##         signatureid similarity       pValue
## 5803  LINCSCP_87253  -0.244290 6.533035e-20
## 5888 LINCSCP_131173  -0.242495 1.035041e-19
## 5944  LINCSCP_88239  -0.241524 1.325860e-19
## 6177  LINCSCP_26724  -0.236467 4.728804e-19
## 6180  LINCSCP_83342  -0.236441 4.758512e-19
## 6263  LINCSCP_53924  -0.234665 7.386208e-19
## [1] "========================================================================"
## [1] "get connected signatures based on the downloaded signature with p value"
## [1] "========================================================================"
## [1] "processedSig_Thu_Jan_28_11_51_42_2021_7491813.xls"
##       signatureid similarity       pValue
## 1: LINCSCP_310240 -0.3831232 1.507158e-35
## 2:    LINCSCP_944 -0.3723046 1.624909e-33
## 3:  LINCSCP_88755 -0.3660801 2.217430e-32
## 4:  LINCSCP_53924 -0.3546742 2.300467e-30
## 5:  LINCSCP_12044 -0.3465831 5.530768e-29
## 6:  LINCSCP_26724 -0.3438364 1.593916e-28
## [1] "==========================================================================================================="
## [1] "checking the accuracy of results via offline benchmarking on the downloaded complete signature with p-value"
## [1] "==========================================================================================================="
## [1] "For signature: LINCSCP_310240 calculated weighted correlation is:-0.383123295246195 ilincs query results is: -0.3831232136"
## [1] "For signature: LINCSCP_944 calculated weighted correlation is:-0.372304725667069 ilincs query results is: -0.37230459"
## [1] "For signature: LINCSCP_88755 calculated weighted correlation is:-0.366080108984052 ilincs query results is: -0.366080115"
## [1] "For signature: LINCSCP_53924 calculated weighted correlation is:-0.354674211863198 ilincs query results is: -0.3546742143"
## [1] "For signature: LINCSCP_12044 calculated weighted correlation is:-0.346582963746496 ilincs query results is: -0.346583076"
## [1] "=========================================================================="
## [1] "get connected signatures based on the downloaded signature without p value"
## [1] "=========================================================================="
## [1] "processedSig_Thu_Jan_28_11_52_25_2021_744006.xls"
##       signatureid similarity       pValue
## 1:  LINCSCP_26724 -0.4190555 7.180876e-43
## 2:    LINCSCP_944 -0.4160253 3.227294e-42
## 3: LINCSCP_310240 -0.4119863 2.335726e-41
## 4:  LINCSCP_88755 -0.4059834 4.211283e-40
## 5:  LINCSCP_83343 -0.4028425 1.868290e-39
## 6:  LINCSCP_11891 -0.4008566 4.753033e-39
## [1] "=============================================================================================================="
## [1] "checking the accuracy of results via offline benchmarking on the downloaded complete signature without p-value"
## [1] "=============================================================================================================="
## [1] "For signature: LINCSCP_26724 calculated weighted correlation is:-0.419055315467304 ilincs query results is: -0.4190554963"
## [1] "For signature: LINCSCP_944 calculated weighted correlation is:-0.416025399162779 ilincs query results is: -0.4160252594"
## [1] "For signature: LINCSCP_310240 calculated weighted correlation is:-0.411986444027985 ilincs query results is: -0.4119863105"
## [1] "For signature: LINCSCP_88755 calculated weighted correlation is:-0.40598340786715 ilincs query results is: -0.4059834076"
## [1] "For signature: LINCSCP_83343 calculated weighted correlation is:-0.402842349405199 ilincs query results is: -0.4028424545"
## [1] "========================================================================================================"
## [1] "checking the accuracy of extreme correlation results via offline benchmarking on the submitted signature"
## [1] "========================================================================================================"
##         signatureid similarity       pValue
## 5803  LINCSCP_87253  -0.244290 6.533035e-20
## 5888 LINCSCP_131173  -0.242495 1.035041e-19
## 5944  LINCSCP_88239  -0.241524 1.325860e-19
## 6177  LINCSCP_26724  -0.236467 4.728804e-19
## 6180  LINCSCP_83342  -0.236441 4.758512e-19
## 6263  LINCSCP_53924  -0.234665 7.386208e-19
```

```
## Selecting by v1
## Selecting by v2
```

```
## Selecting by v1
```

```
## Selecting by v2
```

```
## [1] "For signature: LINCSCP_87253 calculated extreme correlation is:-0.244289517481509 ilincs query results is: -0.24429"
```

```
## Selecting by v1
## Selecting by v2
```

```
## Selecting by v1
```

```
## Selecting by v2
```

```
## [1] "For signature: LINCSCP_131173 calculated extreme correlation is:-0.242495456630676 ilincs query results is: -0.242495"
```

```
## Selecting by v1
## Selecting by v2
```

```
## Selecting by v1
```

```
## Selecting by v2
```

```
## [1] "For signature: LINCSCP_88239 calculated extreme correlation is:-0.24152397997248 ilincs query results is: -0.241524"
```

```
## Selecting by v1
## Selecting by v2
```

```
## Selecting by v1
```

```
## Selecting by v2
```

```
## [1] "For signature: LINCSCP_26724 calculated extreme correlation is:-0.236466617954722 ilincs query results is: -0.236467"
```

```
## Selecting by v1
## Selecting by v2
```

```
## Selecting by v1
```

```
## Selecting by v2
```

```
## [1] "For signature: LINCSCP_83342 calculated extreme correlation is:-0.23644142545828 ilincs query results is: -0.236441"
## [1] "================================================================="
## [1] "get connected signatures based on positive and negative gene list"
## [1] "================================================================="
## [1] "====================================================================================================="
## [1] "checking the accuracy of positive and negative gene list correlation results via offline benchmarking"
## [1] "====================================================================================================="
## [1] "For signature: LINCSCP_107099 calculated correlation is:0.954116320725538 ilincs query results is: 0.9541163164"
## [1] "For signature: LINCSCP_106930 calculated correlation is:0.951552808271607 ilincs query results is: 0.9515528297"
## [1] "For signature: LINCSCP_106931 calculated correlation is:0.951505733883726 ilincs query results is: 0.9515057316"
## [1] "For signature: LINCSCP_107098 calculated correlation is:0.950282201659286 ilincs query results is: 0.9502821915"
## [1] "For signature: LINCSCP_107192 calculated correlation is:0.950067778156498 ilincs query results is: 0.9500677736"
## [1] "++++++++++++++++++++++ Finished ++++++++++++++++++++++"
## [1] "LINCSCP_10628"
## [1] "download signature ======================================"
## [1] "============================================="
## [1] "get connected signatures based on signatureid"
## [1] "============================================="
```

```
## No encoding supplied: defaulting to UTF-8.
```

```
##       signatureid similarity       pValue
## 16  LINCSCP_54613  -0.364322 3.202698e-37
## 22  LINCSCP_55868  -0.362208 7.638012e-37
## 40 LINCSCP_112007  -0.350655 7.875989e-35
## 42  LINCSCP_21331  -0.347514 2.688465e-34
## 54  LINCSCP_25752  -0.343580 1.226536e-33
## 56 LINCSCP_242452  -0.342726 1.700361e-33
## [1] "========================================================================"
## [1] "get connected signatures based on the downloaded signature with p value"
## [1] "========================================================================"
## [1] "processedSig_Thu_Jan_28_11_53_31_2021_1389490.xls"
##      signatureid similarity        pValue
## 1: LINCSCP_58096 -0.6349044 1.747498e-111
## 2: LINCSCP_58219 -0.6333073 9.151464e-111
## 3: LINCSCP_54613 -0.6247614 5.471312e-107
## 4: LINCSCP_58206 -0.6192355 1.312785e-104
## 5: LINCSCP_26094 -0.6166782 1.598199e-103
## 6: LINCSCP_58332 -0.6166361 1.665085e-103
## [1] "==========================================================================================================="
## [1] "checking the accuracy of results via offline benchmarking on the downloaded complete signature with p-value"
## [1] "==========================================================================================================="
## [1] "For signature: LINCSCP_58096 calculated weighted correlation is:-0.634904472710665 ilincs query results is: -0.6349044377"
## [1] "For signature: LINCSCP_58219 calculated weighted correlation is:-0.633307256909334 ilincs query results is: -0.6333072553"
## [1] "For signature: LINCSCP_54613 calculated weighted correlation is:-0.624761456257232 ilincs query results is: -0.6247613817"
## [1] "For signature: LINCSCP_58206 calculated weighted correlation is:-0.619235385923104 ilincs query results is: -0.6192354521"
## [1] "For signature: LINCSCP_26094 calculated weighted correlation is:-0.616678267884164 ilincs query results is: -0.6166781977"
## [1] "=========================================================================="
## [1] "get connected signatures based on the downloaded signature without p value"
## [1] "=========================================================================="
## [1] "processedSig_Thu_Jan_28_11_54_06_2021_5095507.xls"
##      signatureid similarity        pValue
## 1: LINCSCP_58096 -0.6707040 8.653410e-129
## 2: LINCSCP_54613 -0.6638843 2.624641e-125
## 3: LINCSCP_58206 -0.6606417 1.102912e-123
## 4: LINCSCP_26094 -0.6474534 2.765355e-117
## 5: LINCSCP_58332 -0.6435721 1.840709e-115
## 6: LINCSCP_57401 -0.6435710 1.842813e-115
## [1] "=============================================================================================================="
## [1] "checking the accuracy of results via offline benchmarking on the downloaded complete signature without p-value"
## [1] "=============================================================================================================="
## [1] "For signature: LINCSCP_58096 calculated weighted correlation is:-0.670704031188945 ilincs query results is: -0.6707039933"
## [1] "For signature: LINCSCP_54613 calculated weighted correlation is:-0.663884351801467 ilincs query results is: -0.6638842965"
## [1] "For signature: LINCSCP_58206 calculated weighted correlation is:-0.660641659135268 ilincs query results is: -0.6606417404"
## [1] "For signature: LINCSCP_26094 calculated weighted correlation is:-0.647453483587233 ilincs query results is: -0.6474534214"
## [1] "For signature: LINCSCP_58332 calculated weighted correlation is:-0.643572111551641 ilincs query results is: -0.6435720923"
## [1] "========================================================================================================"
## [1] "checking the accuracy of extreme correlation results via offline benchmarking on the submitted signature"
## [1] "========================================================================================================"
##       signatureid similarity       pValue
## 16  LINCSCP_54613  -0.364322 3.202698e-37
## 22  LINCSCP_55868  -0.362208 7.638012e-37
## 40 LINCSCP_112007  -0.350655 7.875989e-35
## 42  LINCSCP_21331  -0.347514 2.688465e-34
## 54  LINCSCP_25752  -0.343580 1.226536e-33
## 56 LINCSCP_242452  -0.342726 1.700361e-33
```

```
## Selecting by v1
## Selecting by v2
```

```
## Selecting by v1
```

```
## Selecting by v2
```

```
## [1] "For signature: LINCSCP_54613 calculated extreme correlation is:-0.36432178798606 ilincs query results is: -0.364322"
```

```
## Selecting by v1
## Selecting by v2
```

```
## Selecting by v1
```

```
## Selecting by v2
```

```
## [1] "For signature: LINCSCP_55868 calculated extreme correlation is:-0.362207888342063 ilincs query results is: -0.362208"
```

```
## Selecting by v1
## Selecting by v2
```

```
## Selecting by v1
```

```
## Selecting by v2
```

```
## [1] "For signature: LINCSCP_112007 calculated extreme correlation is:-0.350655372534115 ilincs query results is: -0.350655"
```

```
## Selecting by v1
## Selecting by v2
```

```
## Selecting by v1
```

```
## Selecting by v2
```

```
## [1] "For signature: LINCSCP_21331 calculated extreme correlation is:-0.347513829571526 ilincs query results is: -0.347514"
```

```
## Selecting by v1
## Selecting by v2
```

```
## Selecting by v1
```

```
## Selecting by v2
```

```
## [1] "For signature: LINCSCP_25752 calculated extreme correlation is:-0.343579959867244 ilincs query results is: -0.34358"
## [1] "================================================================="
## [1] "get connected signatures based on positive and negative gene list"
## [1] "================================================================="
## [1] "====================================================================================================="
## [1] "checking the accuracy of positive and negative gene list correlation results via offline benchmarking"
## [1] "====================================================================================================="
## [1] "For signature: LINCSCP_10628 calculated correlation is:0.972928683101583 ilincs query results is: 0.9729286842"
## [1] "For signature: LINCSCP_10660 calculated correlation is:0.868097972001085 ilincs query results is: 0.8680979707"
## [1] "For signature: LINCSCP_26094 calculated correlation is:-0.809153402723392 ilincs query results is: -0.8091534828"
## [1] "For signature: LINCSCP_58059 calculated correlation is:0.809103348366073 ilincs query results is: 0.8091034156"
## [1] "For signature: LINCSCP_10507 calculated correlation is:0.808842320960912 ilincs query results is: 0.8088423224"
## [1] "++++++++++++++++++++++ Finished ++++++++++++++++++++++"
## [1] "LIB_6"
```

```
## No encoding supplied: defaulting to UTF-8.
```

```
## [1] 1000    5
## [1] "LINCSKD_16281"
## [1] "download signature ======================================"
## [1] "============================================="
## [1] "get connected signatures based on signatureid"
## [1] "============================================="
```

```
## No encoding supplied: defaulting to UTF-8.
```

```
##       signatureid similarity       pValue
## 34  LINCSKD_16044  -0.276017 3.934907e-23
## 99  LINCSKD_14932  -0.253892 2.008799e-20
## 141 LINCSKD_15259  -0.244904 2.144441e-19
## 166 LINCSKD_12435  -0.240720 6.254933e-19
## 248 LINCSKD_20842  -0.230775 7.348772e-18
## 252  LINCSKD_6886  -0.230102 8.645165e-18
## [1] "========================================================================"
## [1] "get connected signatures based on the downloaded signature with p value"
## [1] "========================================================================"
## [1] "processedSig_Thu_Jan_28_11_55_11_2021_272442.xls"
##      signatureid similarity       pValue
## 1: LINCSKD_14932 -0.5364850 5.513271e-74
## 2: LINCSKD_16044 -0.5156581 1.517509e-67
## 3: LINCSKD_15784 -0.4798070 1.874695e-57
## 4: LINCSKD_13642 -0.4788712 3.316000e-57
## 5: LINCSKD_16540 -0.4774263 7.971838e-57
## 6: LINCSKD_10827 -0.4647758 1.445509e-53
## [1] "==========================================================================================================="
## [1] "checking the accuracy of results via offline benchmarking on the downloaded complete signature with p-value"
## [1] "==========================================================================================================="
## [1] "For signature: LINCSKD_14932 calculated weighted correlation is:-0.536485051101379 ilincs query results is: -0.5364849954"
## [1] "For signature: LINCSKD_16044 calculated weighted correlation is:-0.515658098400922 ilincs query results is: -0.5156580893"
## [1] "For signature: LINCSKD_15784 calculated weighted correlation is:-0.479807000823699 ilincs query results is: -0.4798069622"
## [1] "For signature: LINCSKD_13642 calculated weighted correlation is:-0.478871155502169 ilincs query results is: -0.4788711959"
## [1] "For signature: LINCSKD_16540 calculated weighted correlation is:-0.477426303377257 ilincs query results is: -0.4774262793"
## [1] "=========================================================================="
## [1] "get connected signatures based on the downloaded signature without p value"
## [1] "=========================================================================="
## [1] "processedSig_Thu_Jan_28_11_55_39_2021_9447471.xls"
##      signatureid similarity       pValue
## 1: LINCSKD_14932 -0.5683136 9.660538e-85
## 2: LINCSKD_16044 -0.5406655 2.476113e-75
## 3: LINCSKD_15784 -0.5203617 5.830122e-69
## 4:  LINCSKD_7543 -0.5064061 7.971037e-65
## 5: LINCSKD_16165 -0.5034852 5.534917e-64
## 6: LINCSKD_13642 -0.5015887 1.928430e-63
## [1] "=============================================================================================================="
## [1] "checking the accuracy of results via offline benchmarking on the downloaded complete signature without p-value"
## [1] "=============================================================================================================="
## [1] "For signature: LINCSKD_14932 calculated weighted correlation is:-0.568313697493786 ilincs query results is: -0.5683136308"
## [1] "For signature: LINCSKD_16044 calculated weighted correlation is:-0.540665491179002 ilincs query results is: -0.5406654968"
## [1] "For signature: LINCSKD_15784 calculated weighted correlation is:-0.520361755402477 ilincs query results is: -0.5203616724"
## [1] "For signature: LINCSKD_7543 calculated weighted correlation is:-0.506406030334448 ilincs query results is: -0.5064060812"
## [1] "For signature: LINCSKD_16165 calculated weighted correlation is:-0.503485200578451 ilincs query results is: -0.5034852336"
## [1] "========================================================================================================"
## [1] "checking the accuracy of extreme correlation results via offline benchmarking on the submitted signature"
## [1] "========================================================================================================"
##       signatureid similarity       pValue
## 34  LINCSKD_16044  -0.276017 3.934907e-23
## 99  LINCSKD_14932  -0.253892 2.008799e-20
## 141 LINCSKD_15259  -0.244904 2.144441e-19
## 166 LINCSKD_12435  -0.240720 6.254933e-19
## 248 LINCSKD_20842  -0.230775 7.348772e-18
## 252  LINCSKD_6886  -0.230102 8.645165e-18
```

```
## Selecting by v1
## Selecting by v2
```

```
## Selecting by v1
```

```
## Selecting by v2
```

```
## [1] "For signature: LINCSKD_16044 calculated extreme correlation is:-0.276016889989783 ilincs query results is: -0.276017"
```

```
## Selecting by v1
## Selecting by v2
```

```
## Selecting by v1
```

```
## Selecting by v2
```

```
## [1] "For signature: LINCSKD_14932 calculated extreme correlation is:-0.253892434468639 ilincs query results is: -0.253892"
```

```
## Selecting by v1
## Selecting by v2
```

```
## Selecting by v1
```

```
## Selecting by v2
```

```
## [1] "For signature: LINCSKD_15259 calculated extreme correlation is:-0.244904302576221 ilincs query results is: -0.244904"
```

```
## Selecting by v1
## Selecting by v2
```

```
## Selecting by v1
```

```
## Selecting by v2
```

```
## [1] "For signature: LINCSKD_12435 calculated extreme correlation is:-0.240719754189157 ilincs query results is: -0.24072"
```

```
## Selecting by v1
## Selecting by v2
```

```
## Selecting by v1
```

```
## Selecting by v2
```

```
## [1] "For signature: LINCSKD_20842 calculated extreme correlation is:-0.230774629843553 ilincs query results is: -0.230775"
## [1] "================================================================="
## [1] "get connected signatures based on positive and negative gene list"
## [1] "================================================================="
## [1] "====================================================================================================="
## [1] "checking the accuracy of positive and negative gene list correlation results via offline benchmarking"
## [1] "====================================================================================================="
## [1] "For signature: LINCSCP_111630 calculated correlation is:0.7262184875551 ilincs query results is: 0.7262184845"
## [1] "For signature: LINCSCP_111855 calculated correlation is:0.706830801809507 ilincs query results is: 0.7068308285"
## [1] "For signature: LINCSCP_131563 calculated correlation is:0.696231761023878 ilincs query results is: 0.6962318847"
## [1] "For signature: LINCSCP_162777 calculated correlation is:0.689938596828094 ilincs query results is: 0.6899386358"
## [1] "For signature: LINCSCP_111406 calculated correlation is:-0.68860528083592 ilincs query results is: -0.6886053017"
## [1] "++++++++++++++++++++++ Finished ++++++++++++++++++++++"
## [1] "LINCSKD_17213"
## [1] "download signature ======================================"
## [1] "============================================="
## [1] "get connected signatures based on signatureid"
## [1] "============================================="
```

```
## No encoding supplied: defaulting to UTF-8.
```

```
##       signatureid similarity       pValue
## 134 LINCSKD_15831  -0.289013 7.656619e-25
## 165 LINCSKD_15434  -0.283263 4.489863e-24
## 194 LINCSKD_14776  -0.279169 1.542702e-23
## 219 LINCSKD_16165  -0.273333 8.651090e-23
## 235 LINCSKD_15426  -0.272076 1.247161e-22
## 238  LINCSKD_9834  -0.271564 1.446830e-22
## [1] "========================================================================"
## [1] "get connected signatures based on the downloaded signature with p value"
## [1] "========================================================================"
## [1] "processedSig_Thu_Jan_28_11_56_36_2021_9931199.xls"
##      signatureid similarity       pValue
## 1: LINCSKD_15426 -0.5197405 8.992710e-69
## 2: LINCSKD_16165 -0.5096436 9.100768e-66
## 3: LINCSKD_15434 -0.5050314 1.988973e-64
## 4: LINCSKD_15981 -0.4930355 4.876739e-61
## 5: LINCSKD_16197 -0.4861869 3.661188e-59
## 6: LINCSKD_17830 -0.4822297 4.246968e-58
## [1] "==========================================================================================================="
## [1] "checking the accuracy of results via offline benchmarking on the downloaded complete signature with p-value"
## [1] "==========================================================================================================="
## [1] "For signature: LINCSKD_15426 calculated weighted correlation is:-0.519740511879908 ilincs query results is: -0.5197404706"
## [1] "For signature: LINCSKD_16165 calculated weighted correlation is:-0.50964359028906 ilincs query results is: -0.5096436222"
## [1] "For signature: LINCSKD_15434 calculated weighted correlation is:-0.505031379159308 ilincs query results is: -0.5050313844"
## [1] "For signature: LINCSKD_15981 calculated weighted correlation is:-0.493035400950653 ilincs query results is: -0.4930354771"
## [1] "For signature: LINCSKD_16197 calculated weighted correlation is:-0.486186912853063 ilincs query results is: -0.4861869491"
## [1] "=========================================================================="
## [1] "get connected signatures based on the downloaded signature without p value"
## [1] "=========================================================================="
## [1] "processedSig_Thu_Jan_28_11_56_58_2021_4350952.xls"
##      signatureid similarity       pValue
## 1: LINCSKD_15426 -0.5547300 5.222680e-80
## 2: LINCSKD_16165 -0.5482564 7.901905e-78
## 3: LINCSKD_15434 -0.5359756 8.022974e-74
## 4: LINCSKD_16197 -0.5241456 4.080768e-70
## 5: LINCSKD_16143 -0.5205719 5.033703e-69
## 6: LINCSKD_17830 -0.5170839 5.680515e-68
## [1] "=============================================================================================================="
## [1] "checking the accuracy of results via offline benchmarking on the downloaded complete signature without p-value"
## [1] "=============================================================================================================="
## [1] "For signature: LINCSKD_15426 calculated weighted correlation is:-0.554730051133304 ilincs query results is: -0.5547300051"
## [1] "For signature: LINCSKD_16165 calculated weighted correlation is:-0.548256318770304 ilincs query results is: -0.5482563661"
## [1] "For signature: LINCSKD_15434 calculated weighted correlation is:-0.5359756045017 ilincs query results is: -0.5359755821"
## [1] "For signature: LINCSKD_16197 calculated weighted correlation is:-0.524145590777854 ilincs query results is: -0.524145639"
## [1] "For signature: LINCSKD_16143 calculated weighted correlation is:-0.52057187533586 ilincs query results is: -0.5205719217"
## [1] "========================================================================================================"
## [1] "checking the accuracy of extreme correlation results via offline benchmarking on the submitted signature"
## [1] "========================================================================================================"
##       signatureid similarity       pValue
## 134 LINCSKD_15831  -0.289013 7.656619e-25
## 165 LINCSKD_15434  -0.283263 4.489863e-24
## 194 LINCSKD_14776  -0.279169 1.542702e-23
## 219 LINCSKD_16165  -0.273333 8.651090e-23
## 235 LINCSKD_15426  -0.272076 1.247161e-22
## 238  LINCSKD_9834  -0.271564 1.446830e-22
```

```
## Selecting by v1
## Selecting by v2
```

```
## Selecting by v1
```

```
## Selecting by v2
```

```
## [1] "For signature: LINCSKD_15831 calculated extreme correlation is:-0.289013297899273 ilincs query results is: -0.289013"
```

```
## Selecting by v1
## Selecting by v2
```

```
## Selecting by v1
```

```
## Selecting by v2
```

```
## [1] "For signature: LINCSKD_15434 calculated extreme correlation is:-0.283262664107766 ilincs query results is: -0.283263"
```

```
## Selecting by v1
## Selecting by v2
```

```
## Selecting by v1
```

```
## Selecting by v2
```

```
## [1] "For signature: LINCSKD_14776 calculated extreme correlation is:-0.279168908485923 ilincs query results is: -0.279169"
```

```
## Selecting by v1
## Selecting by v2
```

```
## Selecting by v1
```

```
## Selecting by v2
```

```
## [1] "For signature: LINCSKD_16165 calculated extreme correlation is:-0.273332797767788 ilincs query results is: -0.273333"
```

```
## Selecting by v1
## Selecting by v2
```

```
## Selecting by v1
```

```
## Selecting by v2
```

```
## [1] "For signature: LINCSKD_15426 calculated extreme correlation is:-0.272076363156187 ilincs query results is: -0.272076"
## [1] "================================================================="
## [1] "get connected signatures based on positive and negative gene list"
## [1] "================================================================="
## [1] "====================================================================================================="
## [1] "checking the accuracy of positive and negative gene list correlation results via offline benchmarking"
## [1] "====================================================================================================="
## [1] "For signature: LINCSCP_22950 calculated correlation is:0.815337829236837 ilincs query results is: 0.8153378656"
## [1] "For signature: LINCSCP_111517 calculated correlation is:0.8041835817809 ilincs query results is: 0.8041835821"
## [1] "For signature: LINCSCP_21448 calculated correlation is:0.801345451444924 ilincs query results is: 0.8013453693"
## [1] "For signature: LINCSCP_22764 calculated correlation is:0.800604482406323 ilincs query results is: 0.8006045685"
## [1] "For signature: LINCSCP_22880 calculated correlation is:0.796974858638134 ilincs query results is: 0.7969747937"
## [1] "++++++++++++++++++++++ Finished ++++++++++++++++++++++"
## [1] "LINCSKD_12859"
## [1] "download signature ======================================"
## [1] "============================================="
## [1] "get connected signatures based on signatureid"
## [1] "============================================="
```

```
## No encoding supplied: defaulting to UTF-8.
```

```
##       signatureid similarity       pValue
## 53  LINCSKD_12176  -0.337489 4.741677e-32
## 64   LINCSKD_2416  -0.333672 1.961513e-31
## 78  LINCSKD_18002  -0.329930 7.739721e-31
## 82  LINCSKD_33943  -0.328234 1.432918e-30
## 91  LINCSKD_33636  -0.325718 3.547863e-30
## 115 LINCSKD_33954  -0.320883 1.978262e-29
## [1] "========================================================================"
## [1] "get connected signatures based on the downloaded signature with p value"
## [1] "========================================================================"
## [1] "processedSig_Thu_Jan_28_11_57_49_2021_419693.xls"
##      signatureid similarity       pValue
## 1: LINCSKD_33954 -0.5842489 1.393682e-90
## 2: LINCSKD_12932 -0.5794902 8.359912e-89
## 3: LINCSKD_13325 -0.5691318 4.930058e-85
## 4: LINCSKD_13577 -0.5677619 1.518952e-84
## 5: LINCSKD_13822 -0.5605063 5.395658e-82
## 6: LINCSKD_12099 -0.5521766 3.831825e-79
## [1] "==========================================================================================================="
## [1] "checking the accuracy of results via offline benchmarking on the downloaded complete signature with p-value"
## [1] "==========================================================================================================="
## [1] "For signature: LINCSKD_33954 calculated weighted correlation is:-0.584248956483692 ilincs query results is: -0.5842489492"
## [1] "For signature: LINCSKD_12932 calculated weighted correlation is:-0.579490104886752 ilincs query results is: -0.5794901841"
## [1] "For signature: LINCSKD_13325 calculated weighted correlation is:-0.566535062172469 ilincs query results is: -0.5691318105"
## [1] "For signature: LINCSKD_13577 calculated weighted correlation is:-0.56776184710202 ilincs query results is: -0.5677619082"
## [1] "For signature: LINCSKD_13822 calculated weighted correlation is:-0.56050625817766 ilincs query results is: -0.5605062622"
## [1] "=========================================================================="
## [1] "get connected signatures based on the downloaded signature without p value"
## [1] "=========================================================================="
## [1] "processedSig_Thu_Jan_28_11_58_11_2021_4695621.xls"
##      signatureid similarity        pValue
## 1: LINCSKD_13325 -0.6445413 6.488904e-116
## 2: LINCSKD_12986 -0.6444897 6.860346e-116
## 3: LINCSKD_33954 -0.6337353 5.877687e-111
## 4: LINCSKD_12099 -0.6285972 1.141713e-108
## 5: LINCSKD_13577 -0.6276394 3.015828e-108
## 6: LINCSKD_12932 -0.6270840 5.288546e-108
## [1] "=============================================================================================================="
## [1] "checking the accuracy of results via offline benchmarking on the downloaded complete signature without p-value"
## [1] "=============================================================================================================="
## [1] "For signature: LINCSKD_13325 calculated weighted correlation is:-0.643171701761892 ilincs query results is: -0.6445413393"
## [1] "For signature: LINCSKD_12986 calculated weighted correlation is:-0.644489773928139 ilincs query results is: -0.6444896823"
## [1] "For signature: LINCSKD_33954 calculated weighted correlation is:-0.633735271092594 ilincs query results is: -0.633735273"
## [1] "For signature: LINCSKD_12099 calculated weighted correlation is:-0.628597258630344 ilincs query results is: -0.6285972447"
## [1] "For signature: LINCSKD_13577 calculated weighted correlation is:-0.627639313925832 ilincs query results is: -0.6276394034"
## [1] "========================================================================================================"
## [1] "checking the accuracy of extreme correlation results via offline benchmarking on the submitted signature"
## [1] "========================================================================================================"
##       signatureid similarity       pValue
## 53  LINCSKD_12176  -0.337489 4.741677e-32
## 64   LINCSKD_2416  -0.333672 1.961513e-31
## 78  LINCSKD_18002  -0.329930 7.739721e-31
## 82  LINCSKD_33943  -0.328234 1.432918e-30
## 91  LINCSKD_33636  -0.325718 3.547863e-30
## 115 LINCSKD_33954  -0.320883 1.978262e-29
```

```
## Selecting by v1
## Selecting by v2
```

```
## Selecting by v1
```

```
## Selecting by v2
```

```
## [1] "For signature: LINCSKD_12176 calculated extreme correlation is:-0.336791587024924 ilincs query results is: -0.337489"
```

```
## Selecting by v1
## Selecting by v2
```

```
## Selecting by v1
```

```
## Selecting by v2
```

```
## [1] "For signature: LINCSKD_2416 calculated extreme correlation is:-0.33367202584552 ilincs query results is: -0.333672"
```

```
## Selecting by v1
## Selecting by v2
```

```
## Selecting by v1
```

```
## Selecting by v2
```

```
## [1] "For signature: LINCSKD_18002 calculated extreme correlation is:-0.329930424412552 ilincs query results is: -0.32993"
```

```
## Selecting by v1
## Selecting by v2
```

```
## Selecting by v1
```

```
## Selecting by v2
```

```
## [1] "For signature: LINCSKD_33943 calculated extreme correlation is:-0.328234461915239 ilincs query results is: -0.328234"
```

```
## Selecting by v1
## Selecting by v2
```

```
## Selecting by v1
```

```
## Selecting by v2
```

```
## [1] "For signature: LINCSKD_33636 calculated extreme correlation is:-0.325718386748615 ilincs query results is: -0.325718"
## [1] "================================================================="
## [1] "get connected signatures based on positive and negative gene list"
## [1] "================================================================="
## [1] "====================================================================================================="
## [1] "checking the accuracy of positive and negative gene list correlation results via offline benchmarking"
## [1] "====================================================================================================="
## [1] "For signature: LINCSCP_130135 calculated correlation is:-0.835191551365739 ilincs query results is: -0.8351914722"
## [1] "For signature: LINCSCP_100040 calculated correlation is:-0.833061253600668 ilincs query results is: -0.8330612386"
## [1] "For signature: LINCSCP_58144 calculated correlation is:0.831265729868658 ilincs query results is: 0.8312657607"
## [1] "For signature: LINCSCP_106267 calculated correlation is:-0.824692828453626 ilincs query results is: -0.8246927718"
## [1] "For signature: LINCSCP_58219 calculated correlation is:0.823362272736023 ilincs query results is: 0.8233623039"
## [1] "++++++++++++++++++++++ Finished ++++++++++++++++++++++"
## [1] "LIB_11"
```

```
## No encoding supplied: defaulting to UTF-8.
```

```
## [1] 1000    5
## [1] "LINCSOE_11412"
## [1] "download signature ======================================"
## [1] "============================================="
## [1] "get connected signatures based on signatureid"
## [1] "============================================="
```

```
## No encoding supplied: defaulting to UTF-8.
```

```
##      signatureid similarity       pValue
## 14 LINCSOE_11271  -0.336818 2.444829e-31
## 15 LINCSOE_11160  -0.336381 2.878104e-31
## 29 LINCSOE_11374  -0.313684 9.682227e-28
## 33 LINCSOE_11413  -0.311190 2.266632e-27
## 45 LINCSOE_12701  -0.301045 6.637118e-26
## 49 LINCSOE_11588  -0.299784 1.000395e-25
## [1] "========================================================================"
## [1] "get connected signatures based on the downloaded signature with p value"
## [1] "========================================================================"
## [1] "processedSig_Thu_Jan_28_11_59_06_2021_5059130.xls"
##      signatureid similarity       pValue
## 1:  LINCSOE_6575 -0.4717082 2.462243e-55
## 2:  LINCSOE_3685 -0.4646042 1.596984e-53
## 3: LINCSOE_10789 -0.4599639 2.312218e-52
## 4:  LINCSOE_7331 -0.4509406 3.719781e-50
## 5: LINCSOE_12109 -0.4452389 8.531206e-49
## 6:  LINCSOE_6148 -0.4430830 2.746125e-48
## [1] "==========================================================================================================="
## [1] "checking the accuracy of results via offline benchmarking on the downloaded complete signature with p-value"
## [1] "==========================================================================================================="
## [1] "For signature: LINCSOE_6575 calculated weighted correlation is:-0.471708253435572 ilincs query results is: -0.4717082168"
## [1] "For signature: LINCSOE_3685 calculated weighted correlation is:-0.464604145561246 ilincs query results is: -0.4646041861"
## [1] "For signature: LINCSOE_10789 calculated weighted correlation is:-0.459963839083102 ilincs query results is: -0.459963851"
## [1] "For signature: LINCSOE_7331 calculated weighted correlation is:-0.450940496304806 ilincs query results is: -0.4509405966"
## [1] "For signature: LINCSOE_12109 calculated weighted correlation is:-0.445238838128058 ilincs query results is: -0.4452388846"
## [1] "=========================================================================="
## [1] "get connected signatures based on the downloaded signature without p value"
## [1] "=========================================================================="
## [1] "processedSig_Thu_Jan_28_11_59_24_2021_6735654.xls"
##      signatureid similarity       pValue
## 1: LINCSOE_10789 -0.5063562 8.240275e-65
## 2:  LINCSOE_6575 -0.4886842 7.667584e-60
## 3:  LINCSOE_3685 -0.4873910 1.725720e-59
## 4:  LINCSOE_7414 -0.4811451 8.268326e-58
## 5:  LINCSOE_6148 -0.4800451 1.620999e-57
## 6: LINCSOE_12101 -0.4783316 4.603434e-57
## [1] "=============================================================================================================="
## [1] "checking the accuracy of results via offline benchmarking on the downloaded complete signature without p-value"
## [1] "=============================================================================================================="
## [1] "For signature: LINCSOE_10789 calculated weighted correlation is:-0.506356254474475 ilincs query results is: -0.5063562492"
## [1] "For signature: LINCSOE_6575 calculated weighted correlation is:-0.488684266757239 ilincs query results is: -0.4886842275"
## [1] "For signature: LINCSOE_3685 calculated weighted correlation is:-0.487390943024177 ilincs query results is: -0.4873909773"
## [1] "For signature: LINCSOE_7414 calculated weighted correlation is:-0.481145133417887 ilincs query results is: -0.4811450639"
## [1] "For signature: LINCSOE_6148 calculated weighted correlation is:-0.480045166308155 ilincs query results is: -0.4800450752"
## [1] "========================================================================================================"
## [1] "checking the accuracy of extreme correlation results via offline benchmarking on the submitted signature"
## [1] "========================================================================================================"
##      signatureid similarity       pValue
## 14 LINCSOE_11271  -0.336818 2.444829e-31
## 15 LINCSOE_11160  -0.336381 2.878104e-31
## 29 LINCSOE_11374  -0.313684 9.682227e-28
## 33 LINCSOE_11413  -0.311190 2.266632e-27
## 45 LINCSOE_12701  -0.301045 6.637118e-26
## 49 LINCSOE_11588  -0.299784 1.000395e-25
```

```
## Selecting by v1
## Selecting by v2
```

```
## Selecting by v1
```

```
## Selecting by v2
```

```
## [1] "For signature: LINCSOE_11271 calculated extreme correlation is:-0.336818243537825 ilincs query results is: -0.336818"
```

```
## Selecting by v1
## Selecting by v2
```

```
## Selecting by v1
```

```
## Selecting by v2
```

```
## [1] "For signature: LINCSOE_11160 calculated extreme correlation is:-0.336381310381895 ilincs query results is: -0.336381"
```

```
## Selecting by v1
## Selecting by v2
```

```
## Selecting by v1
```

```
## Selecting by v2
```

```
## [1] "For signature: LINCSOE_11374 calculated extreme correlation is:-0.313683546862591 ilincs query results is: -0.313684"
```

```
## Selecting by v1
## Selecting by v2
```

```
## Selecting by v1
```

```
## Selecting by v2
```

```
## [1] "For signature: LINCSOE_11413 calculated extreme correlation is:-0.311189599083612 ilincs query results is: -0.31119"
```

```
## Selecting by v1
## Selecting by v2
```

```
## Selecting by v1
```

```
## Selecting by v2
```

```
## [1] "For signature: LINCSOE_12701 calculated extreme correlation is:-0.301044650584856 ilincs query results is: -0.301045"
## [1] "================================================================="
## [1] "get connected signatures based on positive and negative gene list"
## [1] "================================================================="
## [1] "====================================================================================================="
## [1] "checking the accuracy of positive and negative gene list correlation results via offline benchmarking"
## [1] "====================================================================================================="
## [1] "For signature: LINCSCP_21159 calculated correlation is:0.687235490104146 ilincs query results is: 0.6872354922"
## [1] "For signature: LINCSCP_6683 calculated correlation is:0.670271876863079 ilincs query results is: 0.6702719504"
## [1] "For signature: LINCSCP_40300 calculated correlation is:-0.668876114524641 ilincs query results is: -0.6688761172"
## [1] "For signature: LINCSCP_111665 calculated correlation is:0.664275934485907 ilincs query results is: 0.6642758922"
## [1] "For signature: LINCSCP_6808 calculated correlation is:0.664156380929023 ilincs query results is: 0.6641564024"
## [1] "++++++++++++++++++++++ Finished ++++++++++++++++++++++"
## [1] "LINCSOE_9867"
## [1] "download signature ======================================"
## [1] "============================================="
## [1] "get connected signatures based on signatureid"
## [1] "============================================="
```

```
## No encoding supplied: defaulting to UTF-8.
```

```
##      signatureid similarity       pValue
## 2   LINCSOE_9670  -0.273546 3.261692e-22
## 4  LINCSOE_10496  -0.265009 3.779385e-21
## 6   LINCSOE_9696  -0.258729 2.167040e-20
## 8  LINCSOE_10531  -0.257688 2.882088e-20
## 9  LINCSOE_10488  -0.253250 9.574567e-20
## 10  LINCSOE_9721  -0.250135 2.193535e-19
## [1] "========================================================================"
## [1] "get connected signatures based on the downloaded signature with p value"
## [1] "========================================================================"
## [1] "processedSig_Thu_Jan_28_12_00_16_2021_3182597.xls"
##      signatureid similarity       pValue
## 1: LINCSOE_10496 -0.3153014 5.158399e-24
## 2:  LINCSOE_9670 -0.3075618 7.153452e-23
## 3: LINCSOE_10531 -0.3024907 3.841595e-22
## 4: LINCSOE_10488 -0.2857637 7.793733e-20
## 5:  LINCSOE_9927 -0.2770520 1.079920e-18
## 6: LINCSOE_10519 -0.2713349 5.763638e-18
## [1] "==========================================================================================================="
## [1] "checking the accuracy of results via offline benchmarking on the downloaded complete signature with p-value"
## [1] "==========================================================================================================="
## [1] "For signature: LINCSOE_10496 calculated weighted correlation is:-0.315301395556626 ilincs query results is: -0.3153013584"
## [1] "For signature: LINCSOE_9670 calculated weighted correlation is:-0.307561859335871 ilincs query results is: -0.3075618496"
## [1] "For signature: LINCSOE_10531 calculated weighted correlation is:-0.302490740423512 ilincs query results is: -0.3024906921"
## [1] "For signature: LINCSOE_10488 calculated weighted correlation is:-0.285763642656693 ilincs query results is: -0.2857636692"
## [1] "For signature: LINCSOE_9927 calculated weighted correlation is:-0.277051988500811 ilincs query results is: -0.2770519826"
## [1] "=========================================================================="
## [1] "get connected signatures based on the downloaded signature without p value"
## [1] "=========================================================================="
## [1] "processedSig_Thu_Jan_28_12_00_36_2021_7096242.xls"
##      signatureid similarity       pValue
## 1: LINCSOE_10496 -0.3537427 3.333255e-30
## 2: LINCSOE_10531 -0.3412294 4.310562e-28
## 3:  LINCSOE_9670 -0.3405718 5.531950e-28
## 4: LINCSOE_10488 -0.3311675 1.836394e-26
## 5: LINCSOE_10519 -0.3296852 3.154431e-26
## 6:  LINCSOE_9927 -0.3079501 6.281155e-23
## [1] "=============================================================================================================="
## [1] "checking the accuracy of results via offline benchmarking on the downloaded complete signature without p-value"
## [1] "=============================================================================================================="
## [1] "For signature: LINCSOE_10496 calculated weighted correlation is:-0.353742702576605 ilincs query results is: -0.3537426559"
## [1] "For signature: LINCSOE_10531 calculated weighted correlation is:-0.341229488384105 ilincs query results is: -0.3412294382"
## [1] "For signature: LINCSOE_9670 calculated weighted correlation is:-0.340571827815174 ilincs query results is: -0.3405718139"
## [1] "For signature: LINCSOE_10488 calculated weighted correlation is:-0.331167420167534 ilincs query results is: -0.3311674587"
## [1] "For signature: LINCSOE_10519 calculated weighted correlation is:-0.329685113212314 ilincs query results is: -0.3296852318"
## [1] "========================================================================================================"
## [1] "checking the accuracy of extreme correlation results via offline benchmarking on the submitted signature"
## [1] "========================================================================================================"
##      signatureid similarity       pValue
## 2   LINCSOE_9670  -0.273546 3.261692e-22
## 4  LINCSOE_10496  -0.265009 3.779385e-21
## 6   LINCSOE_9696  -0.258729 2.167040e-20
## 8  LINCSOE_10531  -0.257688 2.882088e-20
## 9  LINCSOE_10488  -0.253250 9.574567e-20
## 10  LINCSOE_9721  -0.250135 2.193535e-19
```

```
## Selecting by v1
## Selecting by v2
```

```
## Selecting by v1
```

```
## Selecting by v2
```

```
## [1] "For signature: LINCSOE_9670 calculated extreme correlation is:-0.273545575851758 ilincs query results is: -0.273546"
```

```
## Selecting by v1
## Selecting by v2
```

```
## Selecting by v1
```

```
## Selecting by v2
```

```
## [1] "For signature: LINCSOE_10496 calculated extreme correlation is:-0.265008825621446 ilincs query results is: -0.265009"
```

```
## Selecting by v1
## Selecting by v2
```

```
## Selecting by v1
```

```
## Selecting by v2
```

```
## [1] "For signature: LINCSOE_9696 calculated extreme correlation is:-0.258729496632758 ilincs query results is: -0.258729"
```

```
## Selecting by v1
## Selecting by v2
```

```
## Selecting by v1
```

```
## Selecting by v2
```

```
## [1] "For signature: LINCSOE_10531 calculated extreme correlation is:-0.257687923362562 ilincs query results is: -0.257688"
```

```
## Selecting by v1
## Selecting by v2
```

```
## Selecting by v1
```

```
## Selecting by v2
```

```
## [1] "For signature: LINCSOE_10488 calculated extreme correlation is:-0.253250327790089 ilincs query results is: -0.25325"
## [1] "================================================================="
## [1] "get connected signatures based on positive and negative gene list"
## [1] "================================================================="
## [1] "====================================================================================================="
## [1] "checking the accuracy of positive and negative gene list correlation results via offline benchmarking"
## [1] "====================================================================================================="
## [1] "For signature: LINCSCP_42620 calculated correlation is:0.4964390998132 ilincs query results is: 0.4964387917"
## [1] "For signature: LINCSCP_41094 calculated correlation is:-0.481285936557922 ilincs query results is: -0.4812859348"
## [1] "For signature: LINCSCP_6788 calculated correlation is:-0.477167950578203 ilincs query results is: -0.4771681179"
## [1] "For signature: LINCSCP_57228 calculated correlation is:-0.471199577764771 ilincs query results is: -0.4711996023"
## [1] "For signature: LINCSCP_292333 calculated correlation is:-0.469034080718435 ilincs query results is: -0.4690340304"
## [1] "++++++++++++++++++++++ Finished ++++++++++++++++++++++"
## [1] "LINCSOE_260"
## [1] "download signature ======================================"
## [1] "============================================="
## [1] "get connected signatures based on signatureid"
## [1] "============================================="
```

```
## No encoding supplied: defaulting to UTF-8.
```

```
##     signatureid similarity       pValue
## 1  LINCSOE_1152  -0.414022 9.302206e-46
## 3    LINCSOE_95  -0.380437 5.272624e-39
## 5  LINCSOE_1396  -0.376384 3.057637e-38
## 7  LINCSOE_1829  -0.371465 2.496476e-37
## 10  LINCSOE_793  -0.368570 8.447482e-37
## 15 LINCSOE_1751  -0.358071 6.337779e-35
## [1] "========================================================================"
## [1] "get connected signatures based on the downloaded signature with p value"
## [1] "========================================================================"
## [1] "processedSig_Thu_Jan_28_12_01_21_2021_3880362.xls"
##      signatureid similarity       pValue
## 1:   LINCSOE_390 -0.4447315 1.124138e-48
## 2: LINCSOE_18685 -0.4100652 5.931449e-41
## 3:  LINCSOE_2133 -0.4078251 1.744981e-40
## 4:   LINCSOE_429 -0.4044708 8.647354e-40
## 5:  LINCSOE_1152 -0.4004620 5.717565e-39
## 6:    LINCSOE_95 -0.3935225 1.415569e-37
## [1] "==========================================================================================================="
## [1] "checking the accuracy of results via offline benchmarking on the downloaded complete signature with p-value"
## [1] "==========================================================================================================="
## [1] "For signature: LINCSOE_390 calculated weighted correlation is:-0.444731466749284 ilincs query results is: -0.4447315464"
## [1] "For signature: LINCSOE_18685 calculated weighted correlation is:-0.410065234880294 ilincs query results is: -0.4100652338"
## [1] "For signature: LINCSOE_2133 calculated weighted correlation is:-0.407825098841262 ilincs query results is: -0.4078251309"
## [1] "For signature: LINCSOE_429 calculated weighted correlation is:-0.404470545622467 ilincs query results is: -0.4044707526"
## [1] "For signature: LINCSOE_1152 calculated weighted correlation is:-0.400462064288603 ilincs query results is: -0.4004620114"
## [1] "=========================================================================="
## [1] "get connected signatures based on the downloaded signature without p value"
## [1] "=========================================================================="
## [1] "processedSig_Thu_Jan_28_12_01_40_2021_1186749.xls"
##     signatureid similarity       pValue
## 1: LINCSOE_1152 -0.4937594 3.071649e-61
## 2: LINCSOE_2133 -0.4788643 3.329854e-57
## 3: LINCSOE_1456 -0.4423942 3.982762e-48
## 4:  LINCSOE_429 -0.4402985 1.227730e-47
## 5:  LINCSOE_390 -0.4392965 2.097339e-47
## 6:  LINCSOE_742 -0.4049977 6.732976e-40
## [1] "=============================================================================================================="
## [1] "checking the accuracy of results via offline benchmarking on the downloaded complete signature without p-value"
## [1] "=============================================================================================================="
## [1] "For signature: LINCSOE_1152 calculated weighted correlation is:-0.493759488901497 ilincs query results is: -0.4937594382"
## [1] "For signature: LINCSOE_2133 calculated weighted correlation is:-0.478864299197088 ilincs query results is: -0.4788643444"
## [1] "For signature: LINCSOE_1456 calculated weighted correlation is:-0.44239435161857 ilincs query results is: -0.4423941741"
## [1] "For signature: LINCSOE_429 calculated weighted correlation is:-0.440298409036581 ilincs query results is: -0.4402985353"
## [1] "For signature: LINCSOE_390 calculated weighted correlation is:-0.439296533688549 ilincs query results is: -0.4392965247"
## [1] "========================================================================================================"
## [1] "checking the accuracy of extreme correlation results via offline benchmarking on the submitted signature"
## [1] "========================================================================================================"
##     signatureid similarity       pValue
## 1  LINCSOE_1152  -0.414022 9.302206e-46
## 3    LINCSOE_95  -0.380437 5.272624e-39
## 5  LINCSOE_1396  -0.376384 3.057637e-38
## 7  LINCSOE_1829  -0.371465 2.496476e-37
## 10  LINCSOE_793  -0.368570 8.447482e-37
## 15 LINCSOE_1751  -0.358071 6.337779e-35
```

```
## Selecting by v1
## Selecting by v2
```

```
## Selecting by v1
```

```
## Selecting by v2
```

```
## [1] "For signature: LINCSOE_1152 calculated extreme correlation is:-0.414022006101451 ilincs query results is: -0.414022"
```

```
## Selecting by v1
## Selecting by v2
```

```
## Selecting by v1
```

```
## Selecting by v2
```

```
## [1] "For signature: LINCSOE_95 calculated extreme correlation is:-0.380436773427994 ilincs query results is: -0.380437"
```

```
## Selecting by v1
## Selecting by v2
```

```
## Selecting by v1
```

```
## Selecting by v2
```

```
## [1] "For signature: LINCSOE_1396 calculated extreme correlation is:-0.376383590492352 ilincs query results is: -0.376384"
```

```
## Selecting by v1
## Selecting by v2
```

```
## Selecting by v1
```

```
## Selecting by v2
```

```
## [1] "For signature: LINCSOE_1829 calculated extreme correlation is:-0.37146496937393 ilincs query results is: -0.371465"
```

```
## Selecting by v1
## Selecting by v2
```

```
## Selecting by v1
```

```
## Selecting by v2
```

```
## [1] "For signature: LINCSOE_793 calculated extreme correlation is:-0.368570112996461 ilincs query results is: -0.36857"
## [1] "================================================================="
## [1] "get connected signatures based on positive and negative gene list"
## [1] "================================================================="
## [1] "====================================================================================================="
## [1] "checking the accuracy of positive and negative gene list correlation results via offline benchmarking"
## [1] "====================================================================================================="
## [1] "For signature: LINCSCP_70374 calculated correlation is:0.78671261950671 ilincs query results is: 0.7867126358"
## [1] "For signature: LINCSCP_179686 calculated correlation is:-0.76945442390021 ilincs query results is: -0.7694544555"
## [1] "For signature: LINCSCP_279015 calculated correlation is:-0.756527551881844 ilincs query results is: -0.7565275534"
## [1] "For signature: LINCSCP_27340 calculated correlation is:0.755734621818846 ilincs query results is: 0.755734584"
## [1] "For signature: LINCSCP_117248 calculated correlation is:0.754415858772133 ilincs query results is: 0.75441587"
## [1] "++++++++++++++++++++++ Finished ++++++++++++++++++++++"
```
