# Supplementary material for: Connecting omics signatures and revealing biological mechanisms with iLINCS
Source: Nat Commun. 2022 Aug 9;13:4678. doi: 10.1038/s41467-022-32205-3 (PMC9362980; doi:10.1038/s41467-022-32205-3)
Supplement: Supplementary file 8 — Software 1 [file 41467_2022_32205_MOESM8_ESM.zip › ilincsAPI-master/qc/usingIlincsApisExample.pdf]

iLINCS API R Notebook

Prerequisites

Define a function to download and retreive ilincs signatures

```
get_ilincs_signature <- function(ilincs_signatureId) {
    req <- POST("http://www.ilincs.org/api/ilincsR/downloadSignature", body = list(sigID = paste(ilincs_
    ilincs_sessionId<-unlist(httr::content(req))
    #print(ilincs_sessionId)
    fileUrl=paste("http://www.ilincs.org/tmp/",ilincs_sessionId,".xls",sep="")
    signatureData<-read.table(fileUrl,sep="\t",header=T,stringsAsFactors = F)
    #print(signatureData)
  return(signatureData)
}
```

```
##
## Attaching package: 'dplyr'

## The following objects are masked from 'package:stats':
##
##     filter, lag

## The following objects are masked from 'package:base':
##
##     intersect, setdiff, setequal, union

## Loading required package: Hmisc

## Loading required package: lattice

## Loading required package: survival

## Loading required package: Formula

## Loading required package: ggplot2

##
## Attaching package: 'Hmisc'

## The following objects are masked from 'package:dplyr':
##
##     src, summarize

## The following objects are masked from 'package:base':
##
##     format.pval, units

## Loading required package: gdata

## gdata: read.xls support for 'XLS' (Excel 97-2004) files ENABLED.

##

## gdata: read.xls support for 'XLSX' (Excel 2007+) files ENABLED.
```

```
##
## Attaching package: 'gdata'
## The following objects are masked from 'package:dplyr':
##
##     combine, first, last
## The following object is masked from 'package:stats':
##
##     nobs
## The following object is masked from 'package:utils':
##
##     object.size
## The following object is masked from 'package:base':
##
##     startsWith
## Loading required package: mice
##
## Attaching package: 'mice'
## The following object is masked from 'package:stats':
##
##     filter
## The following objects are masked from 'package:base':
##
##     cbind, rbind
## Loading required package: data.table
##
## Attaching package: 'data.table'
## The following objects are masked from 'package:gdata':
##
##     first, last
## The following objects are masked from 'package:dplyr':
##
##     between, first, last
```

Display Signature Libraries

```
apiUrl <- "http://www.ilincs.org/api/SignatureLibraries"
req <- GET(apiUrl)
json <- httr::content(req, as = "text")
ilincs_libraries <- fromJSON(json)
ilincs_libraries[,c("libraryID","libraryName")]
```

```
##    libraryID                                          libraryName
## 1      LIB_1                           Disease related signatures
## 2     LIB_10               Cancer therapeutics response signatures
## 3     LIB_11                  LINCS gene overexpression signatures
## 4     LIB_12                                 DrugMatrix signatures
## 5     LIB_13 Transcriptional signatures from EBI Expression Atlas
```

```
## 6     LIB_14         Pharmacogenomics transcriptional signatures
## 7      LIB_2                        Connectivity Map signatures
## 8      LIB_3       ENCODE transcription factor binding signatures
## 9      LIB_5                 LINCS chemical perturbagen signatures
## 10     LIB_6      LINCS consensus gene (CGS) knockdown signatures
## 11     LIB_8                           LINCS RNA-Seq signatures
## 12     LIB_9                  LINCS targeted proteomics signatures
```

Select 3 random signature from libraries LIB_5, LIB_6 and LIB_11 and check their connectivity against 5 reported connected signature

```
number_of_random_sample_signatures <- 3
number_of_random_connected_signatures <- 5
```

Searching for signatures

```
primes_list <- list("LIB_5", "LIB_6", "LIB_11")

for (p in primes_list) {
  print(p)

  ilincs_libId<-p
  apiUrl <- paste("http://www.ilincs.org/api/SignatureMeta/findTermWithSynonyms?library=",ilincs_libId,
  req <- GET(apiUrl)

  ilincs_result_df<-fromJSON(httr::content(req,type="text"))$data


  print(dim(ilincs_result_df[floor(runif(1000, min=0, max=dim(ilincs_result_df)[1])),c("cellline","compo
  counter = 0

  for (sigloop in ilincs_result_df[floor(runif(number_of_random_sample_signatures, min=0, max=dim(ilincs
    ilincs_signatureId = sigloop
    print(ilincs_signatureId)
    counter <- counter + 1

    print("download signature =====================================")
    req <- POST("http://www.ilincs.org/api/ilincsR/downloadSignature", body = list(sigID = paste(ilincs_
    ilincs_sessionId<-unlist(httr::content(req))

    fileUrl=paste("http://www.ilincs.org/tmp/",ilincs_sessionId,".xls",sep="")
    signatureData <-read.table(fileUrl,sep="\t",header=T,stringsAsFactors = F)

    print("==============================================")
    print("get connected signatures based on signatureid")
    print("==============================================")
    apiUrl <- paste("http://www.ilincs.org/api/SignatureMeta/findConcordantSignatures?sigID=",ilincs_sig
    req <- GET(apiUrl)

    ilincs_conn_df<-fromJSON(httr::content(req,type="text"))
    result_of_connected_sigs_from_sigID <- ilincs_conn_df[,c("signatureid","similarity","pValue")]
    result_of_connected_sigs_from_sigID<-result_of_connected_sigs_from_sigID[order(result_of_connected_s
    print(head(result_of_connected_sigs_from_sigID))
```

```r
print("========================================================================")
print("get connected signatures based on the downloaded signature with p value")
print("========================================================================")
#print(ilincs_sessionId[1])
#print(toString(ilincs_sessionId[1]))

file_name <- paste(ilincs_libId,"_", toString(ilincs_sessionId[1]), ".tsv", sep="")
write.table(signatureData,file=file_name,sep="\t",row.names=F,col.names = T,quote=F)
#system(paste("head sigFile.tsv"))


apiUrl<-"http://www.ilincs.org/api/SignatureMeta/upload"
sigFile <- file_name
in_data<-as.data.frame(fread(file_name))
in_data_orig<-as.data.frame(fread(file_name))
#print(in_data)

req <- POST(apiUrl, body=list(file=upload_file(sigFile)))
#req <- POST(apiUrl, body=list(file=signatureData))
signatureFile <- httr::content(req)$status$fileName[[1]]
print(head(signatureFile))

apiUrl <- "http://www.ilincs.org/api/ilincsR/findConcordances"
req <- (POST(apiUrl, body = list(file=signatureFile, lib=ilincs_libId), encode = "form"))
output <- data.table::rbindlist(httr::content(req)$concordanceTable, use.names = TRUE, fill = TRUE)
result_of_upload_complete_sig <- output[,c("signatureid","similarity","pValue")]
result_of_upload_complete_sig<-result_of_upload_complete_sig[order(result_of_upload_complete_sig$si
print(head(result_of_upload_complete_sig))

 #merge(authors, books, by.x="surname", by.y="name")
#for (sigloop in ilincs_result_df[
print("============================================================================================
print("checking the accuracy of results via offline benchmarking on the downloaded complete signatu
print("============================================================================================
  for(varIter in 1:number_of_random_connected_signatures)
     {
      #print(varIter)
      compared_sig = result_of_upload_complete_sig[varIter,"signatureid"]
      #print(compared_sig)
      restart_data <- in_data
      #print("--------------------")

      #vardiff <- get_ilincs_signature(toString(compared_sig))
      #varpval <- as.data.frame(lincscpPValues[, toString(compared_sig)])
      #if (ilincs_libId == "LIB_5"){
        #print(as.data.frame(lincscpDiffExp[, "LINCSCP_159326"]))
       # vardiff <- as.data.frame(lincscpDiffExp[, toString(compared_sig)])
      #  varpval <- as.data.frame(lincscpPValues[, toString(compared_sig)])
      #}
     #if (ilincs_libId == "LIB_6"){
      # vardiff <- as.data.frame(lincskdDiffExp[,toString(compared_sig)])
       # varpval <- as.data.frame(lincskdPValues[,toString(compared_sig)])
```

```r
    #}
    #if (ilincs_libId == "LIB_11"){
     # vardiff <- as.data.frame(lincsoeDiffExp[,toString(compared_sig)])
      # varpval <- as.data.frame(lincsoePValues[,toString(compared_sig)])

  #}
     com_sig <-get_ilincs_signature(toString(compared_sig))
     rownames(com_sig) <- com_sig$ID_geneid
     vardiff <- as.data.frame(com_sig[,c("Value_LogDiffExp")])
     rownames(vardiff) <- com_sig$ID_geneid
     varpval <- as.data.frame(com_sig[,c("Significance_pvalue")])
     rownames(varpval) <- com_sig$ID_geneid

     colnames(vardiff)<- c("compared_sig_dif")
     colnames(varpval)<- c("compared_sig_pval")

     restart_data<-merge(restart_data, vardiff, by.x="ID_geneid", by.y="row.names")
     restart_data<-merge(restart_data, varpval, by.x="ID_geneid", by.y="row.names")
     weight_vec <- c()
     restart_data$"new_weight"<-(-1.0)*(log10(restart_data$"Significance_pvalue")+log10(restart_da
      w_co <- weightedCorr(y=restart_data$"compared_sig_dif", x=restart_data$"Value_LogDiffExp", m

     print(paste0("For signature: ", compared_sig, " calculated weighted correlation is:",w_co, "

  }


print("===========================================================================")
print("get connected signatures based on the downloaded signature without p value")
print("===========================================================================")


file_name <- paste(ilincs_libId,"_withot_pvalue_", toString(ilincs_sessionId[1]), ".tsv", sep="")

signatureData_without_pvalue = subset(signatureData, select = -c(Significance_pvalue) )
write.table(signatureData_without_pvalue,file=file_name,sep="\t",row.names=F,col.names = T,quote=F)

apiUrl<-"http://www.ilincs.org/api/SignatureMeta/upload"
sigFile <- file_name
in_data<-as.data.frame(fread(file_name))


req <- POST(apiUrl, body=list(file=upload_file(sigFile)))

signatureFile <- httr::content(req)$status$fileName[[1]]
print(head(signatureFile))

apiUrl <- "http://www.ilincs.org/api/ilincsR/findConcordances"
req <- (POST(apiUrl, body = list(file=signatureFile, lib=ilincs_libId), encode = "form"))
output <- data.table::rbindlist(httr::content(req)$concordanceTable, use.names = TRUE, fill = TRUE)
result_of_upload_complete_sig <- output[,c("signatureid","similarity","pValue")]
result_of_upload_complete_sig<-result_of_upload_complete_sig[order(result_of_upload_complete_sig$si
print(head(result_of_upload_complete_sig))
```

```
print("==========================================================================================
print("checking the accuracy of results via offline benchmarking on the downloaded complete signatu
print("==========================================================================================
  for(varIter in 1:5)
     {

      compared_sig = result_of_upload_complete_sig[varIter,"signatureid"]

      restart_data <- in_data


      com_sig <-get_ilincs_signature(toString(compared_sig))
      rownames(com_sig) <- com_sig$ID_geneid
      vardiff <- as.data.frame(com_sig[,c("Value_LogDiffExp")])
      rownames(vardiff) <- com_sig$ID_geneid
      varpval <- as.data.frame(com_sig[,c("Significance_pvalue")])
      rownames(varpval) <- com_sig$ID_geneid

      colnames(vardiff)<- c("compared_sig_dif")
      colnames(varpval)<- c("compared_sig_pval")

      restart_data<-merge(restart_data, vardiff, by.x="ID_geneid", by.y="row.names")
      restart_data<-merge(restart_data, varpval, by.x="ID_geneid", by.y="row.names")
      weight_vec <- c()
      restart_data$"new_weight"<-(-1.0)*(log10(restart_data$"compared_sig_pval"))
      w_co <- weightedCorr(y=restart_data$"compared_sig_dif", x=restart_data$"Value_LogDiffExp", me

      print(paste0("For signature: ", compared_sig, " calculated weighted correlation is:",w_co, " 

  }

print("==========================================================================================
print("checking the accuracy of extreme correlation results via offline benchmarking on the submitt
print("==========================================================================================
print(head(result_of_connected_sigs_from_sigID))
  for(varIter in 1:5)
     {

      compared_sig = result_of_connected_sigs_from_sigID[varIter,"signatureid"]

      restart_data <- in_data_orig


      com_sig <-get_ilincs_signature(toString(compared_sig))
      rownames(com_sig) <- com_sig$ID_geneid
      vardiff <- as.data.frame(com_sig[,c("Value_LogDiffExp")])
      rownames(vardiff) <- com_sig$ID_geneid
      varpval <- as.data.frame(com_sig[,c("Significance_pvalue")])
      rownames(varpval) <- com_sig$ID_geneid

      colnames(vardiff)<- c("compared_sig_dif")
      colnames(varpval)<- c("compared_sig_pval")
```

```
        v1<-(-1.0)*sign(vardiff)*log10(varpval)

        colnames(v1)<- c("v1")
        rownames(v1) <- as.list(com_sig[,"ID_geneid"])

        v2<- as.data.frame((-1.0)*sign(restart_data[,"Value_LogDiffExp"])*log10(restart_data[,"Signif
        rownames(v2) <- as.list(restart_data[,"ID_geneid"])
        colnames(v2)<- c("v2")


        top100_1<-v1 %>% top_n(100)
        top100_2<-v2 %>% top_n(100)

        bot100_1<-v1 %>% top_n(-100)
        bot100_2<-v2 %>% top_n(-100)

        row_list_1<-rownames(top100_1)
        row_list_2<-rownames(top100_2)

        row_list_1<-append(row_list_1,rownames(bot100_1))
        row_list_2<-append(row_list_2,rownames(bot100_2))
        for (p in rownames(v1)) {
          if (!(p %in% row_list_1)){

            v1[p,"v1"] <- 0.0
          }
        }


        for (p in rownames(v2)) {

          if (!(p %in% row_list_2)){


            v2[p,"v2"] <- 0.0

          }
        }

        restart_data<-merge(restart_data, v1, by.x="ID_geneid", by.y="row.names")


        restart_data<-merge(restart_data, v2, by.x="ID_geneid", by.y="row.names")


         w_co <- weightedCorr(x=restart_data$"v1", y=restart_data$"v2", method="Pearson")


        print(paste0("For signature: ", compared_sig, " calculated extreme correlation is:",w_co, " i
  }
print("=================================================================")
print("get connected signatures based on positive and negative gene list")
print("=================================================================")
```

```
top100signature <- signatureData[order(signatureData$Significance_pvalue)[1:100],]

apiUrl="http://www.ilincs.org/api/ilincsR/findConcordancesSC"

topUpRegulatedGenes <- list(genesUp=top100signature$Name_GeneSymbol[top100signature$Value_LogDiffExp
topDownregulatedGenes <- list(genesDown=top100signature$Name_GeneSymbol[top100signature$Value_LogDi
topUpRegulatedGeneIDs <- list(top100signature$ID_geneid[top100signature$Value_LogDiffExp > 0])
topDownregulatedGeneIDs <- list(top100signature$ID_geneid[top100signature$Value_LogDiffExp < 0])


req <- POST("http://www.ilincs.org/api/ilincsR/findConcordancesSC", body = list(mode="UpDn",metadata

ilincsUpDnConnectedSignatures <- data.table::rbindlist(httr::content(req)$concordanceTable, use.name

print("=========================================================================================
print("checking the accuracy of positive and negative gene list correlation results via offline benc
print("=========================================================================================

if(TRUE){
  for(varIter in 1:5)
     {

      compared_sig = ilincsUpDnConnectedSignatures[varIter,"signatureID"]

        com_sig <-get_ilincs_signature(toString(compared_sig))
      rownames(com_sig) <- com_sig$ID_geneid
      vardiff <- as.data.frame(com_sig[,c("Value_LogDiffExp")])
      rownames(vardiff) <- com_sig$ID_geneid
      varpval <- as.data.frame(com_sig[,c("Significance_pvalue")])
      rownames(varpval) <- com_sig$ID_geneid

      colnames(vardiff)<- c("compared_sig_dif")
      colnames(varpval)<- c("compared_sig_pval")

      v1<-vardiff

      colnames(v1)<- c("v1")

      v2 <- v1
      colnames(v2)<- c("v2")



      v3 <- data.frame(matrix(ncol=1,nrow=0, dimnames=list(NULL, c("v3"))))
      v4 <- data.frame(matrix(ncol=1,nrow=0, dimnames=list(NULL, c("v4"))))

      vvv <- data.frame(matrix(ncol=2,nrow=0, dimnames=list(NULL, c("xx", "yy"))))

      for (p in rownames(v1)) {

        if ((p %in% unlist(topUpRegulatedGeneIDs, recursive = FALSE))){

          new_row <- c(1.0, v1[p,"v1"])
```

```
            vvv <- rbind(vvv, new_row)

          }
          else if ((p %in% unlist(topDownregulatedGeneIDs, recursive = FALSE))){

            new_row <- c(-1.0, v1[p,"v1"])
            vvv <- rbind(vvv, new_row)


          }


        }

        res <- cor(vvv)


        print(paste0("For signature: ", compared_sig, " calculated correlation is:",unlist(res[1,2]),

      }
    }


    print("++++++++++++++++++++++ Finished ++++++++++++++++++++++")



  }
}
```

```
## [1] "LIB_5"

## No encoding supplied: defaulting to UTF-8.

## [1] 1000    5
## [1] "LINCSCP_100565"
## [1] "download signature ======================================="
## [1] "=============================================="
## [1] "get connected signatures based on signatureid"
## [1] "=============================================="

## No encoding supplied: defaulting to UTF-8.

##        signatureid similarity       pValue
## 75  LINCSCP_100009  -0.343628 1.204267e-33
## 246 LINCSCP_100105  -0.278812 4.462067e-24
## 274  LINCSCP_99657  -0.273476 2.156795e-23
## 276  LINCSCP_99611  -0.273270 2.290700e-23
## 280  LINCSCP_99970  -0.272597 2.787306e-23
## 309  LINCSCP_99733  -0.268607 8.813850e-23
## [1] "========================================================================="
## [1] "get connected signatures based on the downloaded signature with p value"
## [1] "========================================================================="
## [1] "processedSig_Thu_Jan_28_11_35_04_2021_6592757.xls"
##       signatureid similarity       pValue
## 1: LINCSCP_100103 -0.3853837 5.542190e-36
```

```
## 2: LINCSCP_100157 -0.3368827 2.217421e-27
## 3:  LINCSCP_99657 -0.3306965 2.181553e-26
## 4:  LINCSCP_99730 -0.3214693 5.997403e-25
## 5:  LINCSCP_99611 -0.3183347 1.801554e-24
## 6: LINCSCP_100009 -0.3179461 2.062865e-24
## [1] "=============================================================================
## [1] "checking the accuracy of results via offline benchmarking on the downloaded complete signature w
## [1] "=============================================================================
## [1] "For signature: LINCSCP_100103 calculated weighted correlation is:-0.38538378378164 ilincs query
## [1] "For signature: LINCSCP_100157 calculated weighted correlation is:-0.336882660623484 ilincs query
## [1] "For signature: LINCSCP_99657 calculated weighted correlation is:-0.330696569088013 ilincs query
## [1] "For signature: LINCSCP_99730 calculated weighted correlation is:-0.321469394760238 ilincs query
## [1] "For signature: LINCSCP_99611 calculated weighted correlation is:-0.318334615504363 ilincs query
## [1] "======================================================================="
## [1] "get connected signatures based on the downloaded signature without p value"
## [1] "======================================================================="
## [1] "processedSig_Thu_Jan_28_11_35_46_2021_5764155.xls"
##       signatureid similarity       pValue
## 1:  LINCSCP_81480 -0.3915535 3.470370e-37
## 2: LINCSCP_100103 -0.3868863 2.837841e-36
## 3:  LINCSCP_83344 -0.3504977 1.201388e-29
## 4:  LINCSCP_83342 -0.3499755 1.474597e-29
## 5:  LINCSCP_99723 -0.3477191 3.558931e-29
## 6:  LINCSCP_99659 -0.3347465 4.912234e-27
## [1] "=============================================================================
## [1] "checking the accuracy of results via offline benchmarking on the downloaded complete signature w
## [1] "=============================================================================
## [1] "For signature: LINCSCP_81480 calculated weighted correlation is:-0.391553598044763 ilincs query
## [1] "For signature: LINCSCP_100103 calculated weighted correlation is:-0.386886393239925 ilincs query
## [1] "For signature: LINCSCP_83344 calculated weighted correlation is:-0.350497751996947 ilincs query
## [1] "For signature: LINCSCP_83342 calculated weighted correlation is:-0.349975612759529 ilincs query
## [1] "For signature: LINCSCP_99723 calculated weighted correlation is:-0.347719116083672 ilincs query
## [1] "=============================================================================
## [1] "checking the accuracy of extreme correlation results via offline benchmarking on the submitted s
## [1] "=============================================================================
##        signatureid similarity       pValue
## 75  LINCSCP_100009  -0.343628 1.204267e-33
## 246 LINCSCP_100105  -0.278812 4.462067e-24
## 274  LINCSCP_99657  -0.273476 2.156795e-23
## 276  LINCSCP_99611  -0.273270 2.290700e-23
## 280  LINCSCP_99970  -0.272597 2.787306e-23
## 309  LINCSCP_99733  -0.268607 8.813850e-23

## Selecting by v1

## Selecting by v2

## Selecting by v1

## Selecting by v2

## [1] "For signature: LINCSCP_100009 calculated extreme correlation is:-0.343627777234235 ilincs query

## Selecting by v1
## Selecting by v2

## Selecting by v1
```

```
## Selecting by v2

## [1] "For signature: LINCSCP_100105 calculated extreme correlation is:-0.278812018224326 ilincs query

## Selecting by v1
## Selecting by v2

## Selecting by v1

## Selecting by v2

## [1] "For signature: LINCSCP_99657 calculated extreme correlation is:-0.273476405574651 ilincs query

## Selecting by v1
## Selecting by v2

## Selecting by v1

## Selecting by v2

## [1] "For signature: LINCSCP_99611 calculated extreme correlation is:-0.273270079025426 ilincs query

## Selecting by v1
## Selecting by v2

## Selecting by v1

## Selecting by v2

## [1] "For signature: LINCSCP_99970 calculated extreme correlation is:-0.272596700324695 ilincs query
## [1] "============================================================"
## [1] "get connected signatures based on positive and negative gene list"
## [1] "============================================================"
## [1] "==============================================================================
## [1] "checking the accuracy of positive and negative gene list correlation results via offline benchm
## [1] "==============================================================================
## [1] "For signature: LINCSCP_100565 calculated correlation is:0.915106356864751 ilincs query results
## [1] "For signature: LINCSCP_100564 calculated correlation is:0.837902864047339 ilincs query results
## [1] "For signature: LINCSCP_99882 calculated correlation is:0.79523629651884 ilincs query results is
## [1] "For signature: LINCSCP_100563 calculated correlation is:0.790066542107856 ilincs query results
## [1] "For signature: LINCSCP_99416 calculated correlation is:0.787021465222495 ilincs query results i
## [1] "+++++++++++++++++++++ Finished +++++++++++++++++++++"
## [1] "LINCSCP_103649"
## [1] "download signature ========================================"
## [1] "=============================================="
## [1] "get connected signatures based on signatureid"
## [1] "=============================================="

## No encoding supplied: defaulting to UTF-8.

##         signatureid similarity       pValue
## 3600  LINCSCP_30486  -0.246987 3.248226e-20
## 4104 LINCSCP_236585  -0.240475 1.730230e-19
## 4586  LINCSCP_30548  -0.234930 6.918950e-19
## 4710 LINCSCP_177128  -0.233483 9.877825e-19
## 5123  LINCSCP_30501  -0.229448 2.631297e-18
## 5188  LINCSCP_57109  -0.228833 3.050488e-18
## [1] "======================================================================"
## [1] "get connected signatures based on the downloaded signature with p value"
## [1] "======================================================================"
## [1] "processedSig_Thu_Jan_28_11_37_06_2021_605943.xls"
```

```
##       signatureid similarity       pValue
## 1:  LINCSCP_24690 -0.3559235 1.396346e-30
## 2:  LINCSCP_11555 -0.3306363 2.230005e-26
## 3:  LINCSCP_30486 -0.3248069 1.832800e-25
## 4: LINCSCP_108469 -0.3169915 2.874787e-24
## 5:   LINCSCP_1053 -0.3142160 7.493940e-24
## 6:    LINCSCP_687 -0.3103625 2.787226e-23
## [1] "==============================================================================
## [1] "checking the accuracy of results via offline benchmarking on the downloaded complete signature w
## [1] "==============================================================================
## [1] "For signature: LINCSCP_24690 calculated weighted correlation is:-0.355923517764813 ilincs query
## [1] "For signature: LINCSCP_11555 calculated weighted correlation is:-0.330636349774949 ilincs query
## [1] "For signature: LINCSCP_30486 calculated weighted correlation is:-0.324806800411753 ilincs query
## [1] "For signature: LINCSCP_108469 calculated weighted correlation is:-0.316991435460184 ilincs query
## [1] "For signature: LINCSCP_1053 calculated weighted correlation is:-0.31421604849011 ilincs query re
## [1] "======================================================================"
## [1] "get connected signatures based on the downloaded signature without p value"
## [1] "======================================================================"
## [1] "processedSig_Thu_Jan_28_11_37_43_2021_4014373.xls"
##      signatureid similarity       pValue
## 1: LINCSCP_24690 -0.4003577 6.003540e-39
## 2: LINCSCP_43473 -0.3938104 1.241016e-37
## 3:  LINCSCP_1053 -0.3817598 2.745177e-35
## 4: LINCSCP_11555 -0.3794390 7.568548e-35
## 5: LINCSCP_30486 -0.3769653 2.210800e-34
## 6: LINCSCP_19237 -0.3733405 1.045960e-33
## [1] "==============================================================================
## [1] "checking the accuracy of results via offline benchmarking on the downloaded complete signature w
## [1] "==============================================================================
## [1] "For signature: LINCSCP_24690 calculated weighted correlation is:-0.400357790909989 ilincs query
## [1] "For signature: LINCSCP_43473 calculated weighted correlation is:-0.39381038722749 ilincs query r
## [1] "For signature: LINCSCP_1053 calculated weighted correlation is:-0.381759810715179 ilincs query r
## [1] "For signature: LINCSCP_11555 calculated weighted correlation is:-0.379438995753714 ilincs query
## [1] "For signature: LINCSCP_30486 calculated weighted correlation is:-0.376965244264841 ilincs query
## [1] "==============================================================================
## [1] "checking the accuracy of extreme correlation results via offline benchmarking on the submitted s
## [1] "==============================================================================
##         signatureid similarity       pValue
## 3600  LINCSCP_30486  -0.246987 3.248226e-20
## 4104 LINCSCP_236585  -0.240475 1.730230e-19
## 4586  LINCSCP_30548  -0.234930 6.918950e-19
## 4710 LINCSCP_177128  -0.233483 9.877825e-19
## 5123  LINCSCP_30501  -0.229448 2.631297e-18
## 5188  LINCSCP_57109  -0.228833 3.050488e-18

## Selecting by v1
## Selecting by v2

## Selecting by v1

## Selecting by v2

## [1] "For signature: LINCSCP_30486 calculated extreme correlation is:-0.246986605290159 ilincs query r

## Selecting by v1
## Selecting by v2
```

```
## Selecting by v1

## Selecting by v2

## [1] "For signature: LINCSCP_236585 calculated extreme correlation is:-0.240474874107508 ilincs query

## Selecting by v1
## Selecting by v2

## Selecting by v1

## Selecting by v2

## [1] "For signature: LINCSCP_30548 calculated extreme correlation is:-0.234929952495473 ilincs query r

## Selecting by v1
## Selecting by v2

## Selecting by v1

## Selecting by v2

## [1] "For signature: LINCSCP_177128 calculated extreme correlation is:-0.233482529680165 ilincs query

## Selecting by v1
## Selecting by v2

## Selecting by v1

## Selecting by v2

## [1] "For signature: LINCSCP_30501 calculated extreme correlation is:-0.229448377711779 ilincs query r
## [1] "================================================================"
## [1] "get connected signatures based on positive and negative gene list"
## [1] "================================================================"
## [1] "===============================================================================================
## [1] "checking the accuracy of positive and negative gene list correlation results via offline benchma
## [1] "===============================================================================================
## [1] "For signature: LINCSCP_103649 calculated correlation is:0.94283518978144 ilincs query results is
## [1] "For signature: LINCSCP_103650 calculated correlation is:0.862875151742906 ilincs query results r
## [1] "For signature: LINCSCP_109437 calculated correlation is:0.853753678597397 ilincs query results r
## [1] "For signature: LINCSCP_106615 calculated correlation is:0.805553852229115 ilincs query results r
## [1] "For signature: LINCSCP_107205 calculated correlation is:0.786926000617315 ilincs query results r
## [1] "+++++++++++++++++++++ Finished ++++++++++++++++++++++"
## [1] "LINCSCP_10933"
## [1] "download signature ======================================"
## [1] "============================================="
## [1] "get connected signatures based on signatureid"
## [1] "============================================="

## No encoding supplied: defaulting to UTF-8.

##       signatureid similarity       pValue
## 19  LINCSCP_10940  -0.393354 1.066184e-42
## 24 LINCSCP_203408  -0.379284 5.647669e-40
## 26 LINCSCP_204302  -0.377513 1.217135e-39
## 27  LINCSCP_11307  -0.376318 2.038411e-39
## 33 LINCSCP_283893  -0.371062 1.918016e-38
## 34 LINCSCP_203448  -0.370556 2.375543e-38
## [1] "======================================================================"
## [1] "get connected signatures based on the downloaded signature with p value"
## [1] "======================================================================"
```

```
## [1] "processedSig_Thu_Jan_28_11_38_58_2021_934176.xls"
##       signatureid similarity       pValue
## 1: LINCSCP_164361 -0.5242163 3.881783e-70
## 2: LINCSCP_164551 -0.5209663 3.820461e-69
## 3:  LINCSCP_84830 -0.5119675 1.889407e-66
## 4:  LINCSCP_84808 -0.5115754 2.465488e-66
## 5: LINCSCP_165891 -0.5109424 3.785761e-66
## 6: LINCSCP_164759 -0.5044238 2.975585e-64
## [1] "===================================================================================
## [1] "checking the accuracy of results via offline benchmarking on the downloaded complete signature w
## [1] "===================================================================================
## [1] "For signature: LINCSCP_164361 calculated weighted correlation is:-0.524216289054621 ilincs query
## [1] "For signature: LINCSCP_164551 calculated weighted correlation is:-0.520966274975338 ilincs query
## [1] "For signature: LINCSCP_84830 calculated weighted correlation is:-0.511967467367204 ilincs query
## [1] "For signature: LINCSCP_84808 calculated weighted correlation is:-0.511575440886524 ilincs query
## [1] "For signature: LINCSCP_165891 calculated weighted correlation is:-0.510942345948274 ilincs query
## [1] "=========================================================================="
## [1] "get connected signatures based on the downloaded signature without p value"
## [1] "=========================================================================="
## [1] "processedSig_Thu_Jan_28_11_39_38_2021_8031895.xls"
##       signatureid similarity       pValue
## 1: LINCSCP_164361 -0.5274733 3.827676e-71
## 2: LINCSCP_164385 -0.5206082 4.907667e-69
## 3: LINCSCP_164551 -0.5105850 4.820873e-66
## 4:  LINCSCP_82972 -0.5100607 6.869462e-66
## 5: LINCSCP_164759 -0.5090182 1.386477e-65
## 6:  LINCSCP_84115 -0.5004319 4.113467e-63
## [1] "===================================================================================
## [1] "checking the accuracy of results via offline benchmarking on the downloaded complete signature w
## [1] "===================================================================================
## [1] "For signature: LINCSCP_164361 calculated weighted correlation is:-0.527473309203476 ilincs query
## [1] "For signature: LINCSCP_164385 calculated weighted correlation is:-0.520608146638985 ilincs query
## [1] "For signature: LINCSCP_164551 calculated weighted correlation is:-0.510585031761957 ilincs query
## [1] "For signature: LINCSCP_82972 calculated weighted correlation is:-0.510060706544786 ilincs query
## [1] "For signature: LINCSCP_164759 calculated weighted correlation is:-0.509018326192993 ilincs query
## [1] "===================================================================================
## [1] "checking the accuracy of extreme correlation results via offline benchmarking on the submitted s
## [1] "===================================================================================
##       signatureid similarity       pValue
## 19  LINCSCP_10940  -0.393354 1.066184e-42
## 24 LINCSCP_203408  -0.379284 5.647669e-40
## 26 LINCSCP_204302  -0.377513 1.217135e-39
## 27  LINCSCP_11307  -0.376318 2.038411e-39
## 33 LINCSCP_283893  -0.371062 1.918016e-38
## 34 LINCSCP_203448  -0.370556 2.375543e-38

## Selecting by v1
## Selecting by v2

## Selecting by v1

## Selecting by v2

## [1] "For signature: LINCSCP_10940 calculated extreme correlation is:-0.393354341312693 ilincs query i

## Selecting by v1
```

```
## Selecting by v2

## Selecting by v1

## Selecting by v2

## [1] "For signature: LINCSCP_203408 calculated extreme correlation is:-0.379283763176388 ilincs query

## Selecting by v1
## Selecting by v2

## Selecting by v1

## Selecting by v2

## [1] "For signature: LINCSCP_204302 calculated extreme correlation is:-0.377513074457502 ilincs query

## Selecting by v1
## Selecting by v2

## Selecting by v1

## Selecting by v2

## [1] "For signature: LINCSCP_11307 calculated extreme correlation is:-0.376317729801599 ilincs query r

## Selecting by v1
## Selecting by v2

## Selecting by v1

## Selecting by v2

## [1] "For signature: LINCSCP_283893 calculated extreme correlation is:-0.371062445095888 ilincs query
## [1] "================================================================"
## [1] "get connected signatures based on positive and negative gene list"
## [1] "================================================================"
## [1] "==========================================================================================
## [1] "checking the accuracy of positive and negative gene list correlation results via offline benchma
## [1] "==========================================================================================
## [1] "For signature: LINCSCP_10933 calculated correlation is:0.960329231711995 ilincs query results is
## [1] "For signature: LINCSCP_10940 calculated correlation is:-0.826572699668606 ilincs query results i
## [1] "For signature: LINCSCP_10941 calculated correlation is:0.815146123589356 ilincs query results is
## [1] "For signature: LINCSCP_84830 calculated correlation is:-0.812595228090349 ilincs query results i
## [1] "For signature: LINCSCP_10889 calculated correlation is:0.811830547252762 ilincs query results is
## [1] "+++++++++++++++++++++ Finished ++++++++++++++++++++++"
## [1] "LIB_6"

## No encoding supplied: defaulting to UTF-8.

## [1] 1000    5
## [1] "LINCSKD_10602"
## [1] "download signature ====================================="
## [1] "=========================================="
## [1] "get connected signatures based on signatureid"
## [1] "=========================================="

## No encoding supplied: defaulting to UTF-8.

##      signatureid similarity       pValue
## 5  LINCSKD_13280  -0.306661 2.593197e-27
## 12  LINCSKD_7857  -0.286886 1.480109e-24
## 13 LINCSKD_11934  -0.285104 2.559907e-24
```

```
## 14  LINCSKD_7774  -0.284733 2.867657e-24
## 22  LINCSKD_4126  -0.273998 7.122626e-23
## 47 LINCSKD_13776  -0.258656 5.511818e-21
## [1] "=========================================================================="
## [1] "get connected signatures based on the downloaded signature with p value"
## [1] "=========================================================================="
## [1] "processedSig_Thu_Jan_28_11_40_49_2021_1027944.xls"
##      signatureid similarity       pValue
## 1: LINCSKD_18702 -0.4827054 3.168648e-58
## 2: LINCSKD_25927 -0.4617121 8.488524e-53
## 3:  LINCSKD_4126 -0.4499204 6.544019e-50
## 4: LINCSKD_17120 -0.4474539 2.544164e-49
## 5:  LINCSKD_7857 -0.4471871 2.944722e-49
## 6: LINCSKD_15047 -0.4457823 6.345492e-49
## [1] "==============================================================================================
## [1] "checking the accuracy of results via offline benchmarking on the downloaded complete signature w
## [1] "==============================================================================================
## [1] "For signature: LINCSKD_18702 calculated weighted correlation is:-0.482705400842952 ilincs query
## [1] "For signature: LINCSKD_25927 calculated weighted correlation is:-0.461712016849786 ilincs query
## [1] "For signature: LINCSKD_4126 calculated weighted correlation is:-0.449920405718028 ilincs query i
## [1] "For signature: LINCSKD_17120 calculated weighted correlation is:-0.447453815100572 ilincs query
## [1] "For signature: LINCSKD_7857 calculated weighted correlation is:-0.447187122814391 ilincs query i
## [1] "============================================================================"
## [1] "get connected signatures based on the downloaded signature without p value"
## [1] "============================================================================"
## [1] "processedSig_Thu_Jan_28_11_41_13_2021_4470521.xls"
##      signatureid similarity       pValue
## 1: LINCSKD_18702 -0.4794678 2.305672e-57
## 2: LINCSKD_25927 -0.4781845 5.033750e-57
## 3: LINCSKD_22143 -0.4660782 6.773860e-54
## 4: LINCSKD_17120 -0.4647114 1.500648e-53
## 5: LINCSKD_20424 -0.4610282 1.257124e-52
## 6: LINCSKD_15047 -0.4558229 2.425746e-51
## [1] "==============================================================================================
## [1] "checking the accuracy of results via offline benchmarking on the downloaded complete signature w
## [1] "==============================================================================================
## [1] "For signature: LINCSKD_18702 calculated weighted correlation is:-0.479467870069167 ilincs query
## [1] "For signature: LINCSKD_25927 calculated weighted correlation is:-0.478184473754809 ilincs query
## [1] "For signature: LINCSKD_22143 calculated weighted correlation is:-0.466078157765135 ilincs query
## [1] "For signature: LINCSKD_17120 calculated weighted correlation is:-0.464711274879586 ilincs query
## [1] "For signature: LINCSKD_20424 calculated weighted correlation is:-0.461028132861668 ilincs query
## [1] "==============================================================================================
## [1] "checking the accuracy of extreme correlation results via offline benchmarking on the submitted s
## [1] "==============================================================================================
##      signatureid similarity       pValue
## 5  LINCSKD_13280  -0.306661 2.593197e-27
## 12  LINCSKD_7857  -0.286886 1.480109e-24
## 13 LINCSKD_11934  -0.285104 2.559907e-24
## 14  LINCSKD_7774  -0.284733 2.867657e-24
## 22  LINCSKD_4126  -0.273998 7.122626e-23
## 47 LINCSKD_13776  -0.258656 5.511818e-21

## Selecting by v1
## Selecting by v2
```

```
## Selecting by v1

## Selecting by v2

## [1] "For signature: LINCSKD_13280 calculated extreme correlation is:-0.306660941928778 ilincs query r

## Selecting by v1
## Selecting by v2

## Selecting by v1

## Selecting by v2

## [1] "For signature: LINCSKD_7857 calculated extreme correlation is:-0.286885971237325 ilincs query re

## Selecting by v1
## Selecting by v2

## Selecting by v1

## Selecting by v2

## [1] "For signature: LINCSKD_11934 calculated extreme correlation is:-0.285103806760826 ilincs query r

## Selecting by v1
## Selecting by v2

## Selecting by v1

## Selecting by v2

## [1] "For signature: LINCSKD_7774 calculated extreme correlation is:-0.284732906232042 ilincs query re

## Selecting by v1
## Selecting by v2

## Selecting by v1

## Selecting by v2

## [1] "For signature: LINCSKD_4126 calculated extreme correlation is:-0.273997946319758 ilincs query re
## [1] "=============================================================="
## [1] "get connected signatures based on positive and negative gene list"
## [1] "=============================================================="
## [1] "===========================================================================================
## [1] "checking the accuracy of positive and negative gene list correlation results via offline benchma
## [1] "===========================================================================================
## [1] "For signature: LINCSCP_18029 calculated correlation is:-0.768856875452157 ilincs query results r
## [1] "For signature: LINCSCP_132203 calculated correlation is:-0.750787401258136 ilincs query results
## [1] "For signature: LINCSCP_17986 calculated correlation is:-0.744721847290952 ilincs query results r
## [1] "For signature: LINCSCP_17902 calculated correlation is:-0.742031108319138 ilincs query results r
## [1] "For signature: LINCSCP_238255 calculated correlation is:-0.740840649985959 ilincs query results
## [1] "++++++++++++++++++++ Finished ++++++++++++++++++++"
## [1] "LINCSKD_14875"
## [1] "download signature ====================================="
## [1] "============================================="
## [1] "get connected signatures based on signatureid"
## [1] "============================================="

## No encoding supplied: defaulting to UTF-8.

##      signatureid similarity       pValue
## 15 LINCSKD_15984  -0.324698 5.111401e-30
## 54 LINCSKD_15957  -0.299203 3.010983e-26
```

```
## 55 LINCSKD_14882  -0.299097 3.116106e-26
## 64 LINCSKD_16098  -0.295349 1.039711e-25
## 71 LINCSKD_16570  -0.290464 4.868585e-25
## 72  LINCSKD_6712  -0.290221 5.252701e-25
## [1] "======================================================================"
## [1] "get connected signatures based on the downloaded signature with p value"
## [1] "======================================================================"
## [1] "processedSig_Thu_Jan_28_11_42_05_2021_2002516.xls"
##      signatureid similarity       pValue
## 1: LINCSKD_16185 -0.5597403 9.945901e-82
## 2: LINCSKD_16570 -0.5591578 1.581802e-81
## 3: LINCSKD_16165 -0.5467669 2.469173e-77
## 4: LINCSKD_15984 -0.5287907 1.488926e-71
## 5: LINCSKD_15957 -0.5258831 1.189931e-70
## 6:  LINCSKD_2752 -0.5243055 3.644463e-70
## [1] "==============================================================================================
## [1] "checking the accuracy of results via offline benchmarking on the downloaded complete signature w
## [1] "==============================================================================================
## [1] "For signature: LINCSKD_16185 calculated weighted correlation is:-0.559740270575908 ilincs query
## [1] "For signature: LINCSKD_16570 calculated weighted correlation is:-0.559157699731835 ilincs query
## [1] "For signature: LINCSKD_16165 calculated weighted correlation is:-0.546766962938159 ilincs query
## [1] "For signature: LINCSKD_15984 calculated weighted correlation is:-0.528790672434877 ilincs query
## [1] "For signature: LINCSKD_15957 calculated weighted correlation is:-0.525883207511507 ilincs query
## [1] "========================================================================="
## [1] "get connected signatures based on the downloaded signature without p value"
## [1] "========================================================================="
## [1] "processedSig_Thu_Jan_28_11_42_29_2021_4853931.xls"
##      signatureid similarity       pValue
## 1: LINCSKD_16570 -0.6023641 1.255740e-97
## 2: LINCSKD_16185 -0.5941538 2.226096e-94
## 3: LINCSKD_16165 -0.5634470 5.080625e-83
## 4: LINCSKD_15984 -0.5587435 2.198901e-81
## 5:  LINCSKD_6712 -0.5555283 2.790891e-80
## 6:  LINCSKD_8623 -0.5548613 4.711937e-80
## [1] "==============================================================================================
## [1] "checking the accuracy of results via offline benchmarking on the downloaded complete signature w
## [1] "==============================================================================================
## [1] "For signature: LINCSKD_16570 calculated weighted correlation is:-0.602364064202627 ilincs query
## [1] "For signature: LINCSKD_16185 calculated weighted correlation is:-0.594153814027098 ilincs query
## [1] "For signature: LINCSKD_16165 calculated weighted correlation is:-0.563447077046229 ilincs query
## [1] "For signature: LINCSKD_15984 calculated weighted correlation is:-0.558743473284907 ilincs query
## [1] "For signature: LINCSKD_6712 calculated weighted correlation is:-0.555528358029356 ilincs query i
## [1] "==============================================================================================
## [1] "checking the accuracy of extreme correlation results via offline benchmarking on the submitted s
## [1] "==============================================================================================
##      signatureid similarity       pValue
## 15 LINCSKD_15984  -0.324698 5.111401e-30
## 54 LINCSKD_15957  -0.299203 3.010983e-26
## 55 LINCSKD_14882  -0.299097 3.116106e-26
## 64 LINCSKD_16098  -0.295349 1.039711e-25
## 71 LINCSKD_16570  -0.290464 4.868585e-25
## 72  LINCSKD_6712  -0.290221 5.252701e-25

## Selecting by v1
```

```
## Selecting by v2

## Selecting by v1

## Selecting by v2

## [1] "For signature: LINCSKD_15984 calculated extreme correlation is:-0.324698360421918 ilincs query r

## Selecting by v1
## Selecting by v2

## Selecting by v1

## Selecting by v2

## [1] "For signature: LINCSKD_15957 calculated extreme correlation is:-0.299203002998539 ilincs query r

## Selecting by v1
## Selecting by v2

## Selecting by v1

## Selecting by v2

## [1] "For signature: LINCSKD_14882 calculated extreme correlation is:-0.299097067590012 ilincs query r

## Selecting by v1
## Selecting by v2

## Selecting by v1

## Selecting by v2

## [1] "For signature: LINCSKD_16098 calculated extreme correlation is:-0.295349091729216 ilincs query r

## Selecting by v1
## Selecting by v2

## Selecting by v1

## Selecting by v2

## [1] "For signature: LINCSKD_16570 calculated extreme correlation is:-0.29046414443324 ilincs query re
## [1] "=================================================================="
## [1] "get connected signatures based on positive and negative gene list"
## [1] "=================================================================="
## [1] "===================================================================================================
## [1] "checking the accuracy of positive and negative gene list correlation results via offline benchma
## [1] "===================================================================================================
## [1] "For signature: LINCSCP_111517 calculated correlation is:0.803926451945614 ilincs query results i
## [1] "For signature: LINCSCP_21615 calculated correlation is:0.803378125556444 ilincs query results is
## [1] "For signature: LINCSCP_111313 calculated correlation is:0.802043286781822 ilincs query results i
## [1] "For signature: LINCSCP_162831 calculated correlation is:0.778224022275842 ilincs query results i
## [1] "For signature: LINCSCP_163535 calculated correlation is:0.777769035891647 ilincs query results i
## [1] "+++++++++++++++++++++ Finished +++++++++++++++++++++"
## [1] "LINCSKD_16669"
## [1] "download signature ======================================="
## [1] "============================================="
## [1] "get connected signatures based on signatureid"
## [1] "============================================="

## No encoding supplied: defaulting to UTF-8.
```

```
##      signatureid similarity       pValue
## 14 LINCSKD_19917  -0.177944 5.668044e-13
## 66 LINCSKD_16367   0.153877 3.574639e-11
## 65 LINCSKD_11461   0.153943 3.536965e-11
## 64 LINCSKD_16512   0.154086 3.457196e-11
## 63  LINCSKD_7546   0.154181 3.404512e-11
## 62 LINCSKD_31284   0.154635 3.165494e-11
## [1] "======================================================================"
## [1] "get connected signatures based on the downloaded signature with p value"
## [1] "======================================================================"
## [1] "processedSig_Thu_Jan_28_11_43_23_2021_8230496.xls"
##      signatureid similarity       pValue
## 1: LINCSKD_28849 -0.2852211 9.205370e-20
## 2: LINCSKD_17900 -0.2849553 9.985937e-20
## 3: LINCSKD_27045 -0.2617483 8.746621e-17
## 4:  LINCSKD_1657 -0.2447595 8.297904e-15
## 5: LINCSKD_19595 -0.2379419 4.692902e-14
## 6:  LINCSKD_3411 -0.2363440 6.989577e-14
## [1] "=========================================================================================
## [1] "checking the accuracy of results via offline benchmarking on the downloaded complete signature w
## [1] "=========================================================================================
## [1] "For signature: LINCSKD_28849 calculated weighted correlation is:-0.34156012842032 ilincs query r
## [1] "For signature: LINCSKD_17900 calculated weighted correlation is:-0.284955328445373 ilincs query
## [1] "For signature: LINCSKD_27045 calculated weighted correlation is:-0.261748389239326 ilincs query
## [1] "For signature: LINCSKD_1657 calculated weighted correlation is:-0.244759466396629 ilincs query r
## [1] "For signature: LINCSKD_19595 calculated weighted correlation is:-0.237941900409615 ilincs query
## [1] "========================================================================="
## [1] "get connected signatures based on the downloaded signature without p value"
## [1] "========================================================================="
## [1] "processedSig_Thu_Jan_28_11_43_49_2021_3211352.xls"
##      signatureid similarity       pValue
## 1: LINCSKD_28849 -0.4148207 5.840206e-42
## 2: LINCSKD_27045 -0.2988697 1.250262e-21
## 3: LINCSKD_17900 -0.2928353 8.609756e-21
## 4:  LINCSKD_1657 -0.2912411 1.422505e-20
## 5: LINCSKD_21000 -0.2799408 4.563614e-19
## 6: LINCSKD_18785 -0.2636726 5.111868e-17
## [1] "=========================================================================================
## [1] "checking the accuracy of results via offline benchmarking on the downloaded complete signature w
## [1] "=========================================================================================
## [1] "For signature: LINCSKD_28849 calculated weighted correlation is:-0.503934059467431 ilincs query
## [1] "For signature: LINCSKD_27045 calculated weighted correlation is:-0.298869727649913 ilincs query
## [1] "For signature: LINCSKD_17900 calculated weighted correlation is:-0.292835254174552 ilincs query
## [1] "For signature: LINCSKD_1657 calculated weighted correlation is:-0.291241028873985 ilincs query r
## [1] "For signature: LINCSKD_21000 calculated weighted correlation is:-0.280042544484763 ilincs query
## [1] "=========================================================================================
## [1] "checking the accuracy of extreme correlation results via offline benchmarking on the submitted s
## [1] "=========================================================================================
##      signatureid similarity       pValue
## 14 LINCSKD_19917  -0.177944 5.668044e-13
## 66 LINCSKD_16367   0.153877 3.574639e-11
## 65 LINCSKD_11461   0.153943 3.536965e-11
## 64 LINCSKD_16512   0.154086 3.457196e-11
## 63  LINCSKD_7546   0.154181 3.404512e-11
```

```
## 62 LINCSKD_31284   0.154635 3.165494e-11

## Selecting by v1
## Selecting by v2

## Selecting by v1

## Selecting by v2

## [1] "For signature: LINCSKD_19917 calculated extreme correlation is:-0.177944317866885 ilincs query r

## Selecting by v1
## Selecting by v2

## Selecting by v1

## Selecting by v2

## [1] "For signature: LINCSKD_16367 calculated extreme correlation is:0.153876671108612 ilincs query re

## Selecting by v1
## Selecting by v2

## Selecting by v1

## Selecting by v2

## [1] "For signature: LINCSKD_11461 calculated extreme correlation is:0.153942926215902 ilincs query re

## Selecting by v1
## Selecting by v2

## Selecting by v1

## Selecting by v2

## [1] "For signature: LINCSKD_16512 calculated extreme correlation is:0.154085506919031 ilincs query re

## Selecting by v1
## Selecting by v2

## Selecting by v1

## Selecting by v2

## [1] "For signature: LINCSKD_7546 calculated extreme correlation is:0.154181407796622 ilincs query res
## [1] "=================================================================="
## [1] "get connected signatures based on positive and negative gene list"
## [1] "=================================================================="
## [1] "===================================================================================================
## [1] "checking the accuracy of positive and negative gene list correlation results via offline benchma
## [1] "===================================================================================================
## [1] "For signature: LINCSCP_30921 calculated correlation is:0.499935050910346 ilincs query results is
## [1] "For signature: LINCSCP_171583 calculated correlation is:0.487461526348127 ilincs query results i
## [1] "For signature: LINCSCP_4325 calculated correlation is:-0.486545278398794 ilincs query results is
## [1] "For signature: LINCSCP_78786 calculated correlation is:0.483394291678813 ilincs query results is
## [1] "For signature: LINCSCP_22700 calculated correlation is:0.481910815181552 ilincs query results is
## [1] "+++++++++++++++++++++ Finished ++++++++++++++++++++++"
## [1] "LIB_11"

## No encoding supplied: defaulting to UTF-8.

## [1] 1000    5
## [1] "LINCSOE_1562"
## [1] "download signature ======================================"
```

```
## [1] "=============================================="
## [1] "get connected signatures based on signatureid"
## [1] "=============================================="
## No encoding supplied: defaulting to UTF-8.
##     signatureid similarity       pValue
## 2   LINCSOE_616  -0.308367 5.877230e-27
## 14 LINCSOE_1676  -0.279130 6.261310e-23
## 19 LINCSOE_1689  -0.271340 6.194272e-22
## 22  LINCSOE_570  -0.270754 7.337678e-22
## 23 LINCSOE_5505  -0.270146 8.745024e-22
## 24 LINCSOE_2058  -0.268673 1.334910e-21
## [1] "=========================================================================="
## [1] "get connected signatures based on the downloaded signature with p value"
## [1] "=========================================================================="
## [1] "processedSig_Thu_Jan_28_11_44_51_2021_5330131.xls"
##      signatureid similarity       pValue
## 1:  LINCSOE_2058 -0.3834884 1.282940e-35
## 2:  LINCSOE_1676 -0.3801199 5.625394e-35
## 3:  LINCSOE_2919 -0.3798765 6.255392e-35
## 4:  LINCSOE_1413 -0.3597296 3.008756e-31
## 5: LINCSOE_14268 -0.3557628 1.489153e-30
## 6:   LINCSOE_616 -0.3535071 3.660247e-30
## [1] "===================================================================================================
## [1] "checking the accuracy of results via offline benchmarking on the downloaded complete signature w
## [1] "===================================================================================================
## [1] "For signature: LINCSOE_2058 calculated weighted correlation is:-0.383488410520575 ilincs query r
## [1] "For signature: LINCSOE_1676 calculated weighted correlation is:-0.380119961523665 ilincs query r
## [1] "For signature: LINCSOE_2919 calculated weighted correlation is:-0.37987645980425 ilincs query re
## [1] "For signature: LINCSOE_1413 calculated weighted correlation is:-0.359729766910328 ilincs query r
## [1] "For signature: LINCSOE_14268 calculated weighted correlation is:-0.355762685543508 ilincs query 
## [1] "============================================================================="
## [1] "get connected signatures based on the downloaded signature without p value"
## [1] "============================================================================="
## [1] "processedSig_Thu_Jan_28_11_45_12_2021_6275762.xls"
##      signatureid similarity       pValue
## 1:  LINCSOE_2919 -0.4184215 9.846274e-43
## 2:  LINCSOE_2058 -0.4071363 2.427621e-40
## 3:  LINCSOE_1676 -0.3949057 7.512492e-38
## 4: LINCSOE_14268 -0.3889790 1.110743e-36
## 5:  LINCSOE_1413 -0.3815768 2.974558e-35
## 6:  LINCSOE_1782 -0.3788590 9.739014e-35
## [1] "===================================================================================================
## [1] "checking the accuracy of results via offline benchmarking on the downloaded complete signature w
## [1] "===================================================================================================
## [1] "For signature: LINCSOE_2919 calculated weighted correlation is:-0.41842148769901 ilincs query re
## [1] "For signature: LINCSOE_2058 calculated weighted correlation is:-0.407136324828597 ilincs query r
## [1] "For signature: LINCSOE_1676 calculated weighted correlation is:-0.394905731864118 ilincs query r
## [1] "For signature: LINCSOE_14268 calculated weighted correlation is:-0.388978846285307 ilincs query 
## [1] "For signature: LINCSOE_1413 calculated weighted correlation is:-0.38157700244662 ilincs query re
## [1] "===================================================================================================
## [1] "checking the accuracy of extreme correlation results via offline benchmarking on the submitted s
## [1] "===================================================================================================
##     signatureid similarity       pValue
```

```
## 2   LINCSOE_616  -0.308367 5.877230e-27
## 14 LINCSOE_1676  -0.279130 6.261310e-23
## 19 LINCSOE_1689  -0.271340 6.194272e-22
## 22  LINCSOE_570  -0.270754 7.337678e-22
## 23 LINCSOE_5505  -0.270146 8.745024e-22
## 24 LINCSOE_2058  -0.268673 1.334910e-21

## Selecting by v1
## Selecting by v2

## Selecting by v1

## Selecting by v2

## [1] "For signature: LINCSOE_616 calculated extreme correlation is:-0.308367387335526 ilincs query res

## Selecting by v1
## Selecting by v2

## Selecting by v1

## Selecting by v2

## [1] "For signature: LINCSOE_1676 calculated extreme correlation is:-0.279130197726132 ilincs query re

## Selecting by v1
## Selecting by v2

## Selecting by v1

## Selecting by v2

## [1] "For signature: LINCSOE_1689 calculated extreme correlation is:-0.271340007646399 ilincs query re

## Selecting by v1
## Selecting by v2

## Selecting by v1

## Selecting by v2

## [1] "For signature: LINCSOE_570 calculated extreme correlation is:-0.270754113428551 ilincs query res

## Selecting by v1
## Selecting by v2

## Selecting by v1

## Selecting by v2

## [1] "For signature: LINCSOE_5505 calculated extreme correlation is:-0.270145646819855 ilincs query re
## [1] "================================================================"
## [1] "get connected signatures based on positive and negative gene list"
## [1] "================================================================"
## [1] "==========================================================================================
## [1] "checking the accuracy of positive and negative gene list correlation results via offline benchma
## [1] "==========================================================================================
## [1] "For signature: LINCSCP_2857 calculated correlation is:0.727314553903618 ilincs query results is
## [1] "For signature: LINCSCP_88617 calculated correlation is:0.667094622028069 ilincs query results is
## [1] "For signature: LINCSCP_176805 calculated correlation is:0.652344975486778 ilincs query results i
## [1] "For signature: LINCSCP_69017 calculated correlation is:0.646640134425506 ilincs query results is
## [1] "For signature: LINCSCP_70317 calculated correlation is:-0.645290118471407 ilincs query results i
## [1] "+++++++++++++++++++++ Finished ++++++++++++++++++++++"
## [1] "LINCSOE_14495"
```

```
## [1] "download signature ====================================="
## [1] "=============================================="
## [1] "get connected signatures based on signatureid"
## [1] "=============================================="

## No encoding supplied: defaulting to UTF-8.

##      signatureid similarity       pValue
## 14 LINCSOE_14566  -0.465418 1.070886e-57
## 17 LINCSOE_14609  -0.456741 1.554891e-55
## 26 LINCSOE_14459  -0.445713 7.092317e-53
## 29 LINCSOE_14354  -0.440385 1.261888e-51
## 31 LINCSOE_14501  -0.438328 3.782158e-51
## 33 LINCSOE_14510  -0.436162 1.191284e-50
## [1] "======================================================================="
## [1] "get connected signatures based on the downloaded signature with p value"
## [1] "======================================================================="
## [1] "processedSig_Thu_Jan_28_11_45_58_2021_2202707.xls"
##      signatureid similarity        pValue
## 1: LINCSOE_14566 -0.7196098 8.864559e-157
## 2:  LINCSOE_7361 -0.7148543 8.471502e-154
## 3: LINCSOE_14501 -0.7027834 1.679831e-146
## 4: LINCSOE_14332 -0.6970422 3.697131e-143
## 5:  LINCSOE_6236 -0.6856315 9.570525e-137
## 6: LINCSOE_14681 -0.6853320 1.397275e-136
## [1] "=====================================================================================
## [1] "checking the accuracy of results via offline benchmarking on the downloaded complete signature w
## [1] "=====================================================================================
## [1] "For signature: LINCSOE_14566 calculated weighted correlation is:-0.719609807554303 ilincs query
## [1] "For signature: LINCSOE_7361 calculated weighted correlation is:-0.714854321463374 ilincs query r
## [1] "For signature: LINCSOE_14501 calculated weighted correlation is:-0.70278340919591 ilincs query r
## [1] "For signature: LINCSOE_14332 calculated weighted correlation is:-0.697042208686588 ilincs query
## [1] "For signature: LINCSOE_6236 calculated weighted correlation is:-0.68563146282067 ilincs query re
## [1] "=========================================================================="
## [1] "get connected signatures based on the downloaded signature without p value"
## [1] "=========================================================================="
## [1] "processedSig_Thu_Jan_28_11_46_16_2021_2741752.xls"
##      signatureid similarity        pValue
## 1: LINCSOE_14566 -0.7361330 1.236436e-167
## 2: LINCSOE_14332 -0.7258677 8.513198e-161
## 3:  LINCSOE_7361 -0.7053448 5.103558e-148
## 4: LINCSOE_14501 -0.7042154 2.393238e-147
## 5:  LINCSOE_6236 -0.6865381 3.035397e-137
## 6: LINCSOE_14354 -0.6844070 4.483207e-136
## [1] "=====================================================================================
## [1] "checking the accuracy of results via offline benchmarking on the downloaded complete signature w
## [1] "=====================================================================================
## [1] "For signature: LINCSOE_14566 calculated weighted correlation is:-0.736133018042196 ilincs query
## [1] "For signature: LINCSOE_14332 calculated weighted correlation is:-0.725867665908864 ilincs query
## [1] "For signature: LINCSOE_7361 calculated weighted correlation is:-0.705344844354455 ilincs query r
## [1] "For signature: LINCSOE_14501 calculated weighted correlation is:-0.704215381168739 ilincs query
## [1] "For signature: LINCSOE_6236 calculated weighted correlation is:-0.686538091300572 ilincs query r
## [1] "=====================================================================================
## [1] "checking the accuracy of extreme correlation results via offline benchmarking on the submitted s
## [1] "=====================================================================================
```

```
##      signatureid similarity       pValue
## 14 LINCSOE_14566  -0.465418 1.070886e-57
## 17 LINCSOE_14609  -0.456741 1.554891e-55
## 26 LINCSOE_14459  -0.445713 7.092317e-53
## 29 LINCSOE_14354  -0.440385 1.261888e-51
## 31 LINCSOE_14501  -0.438328 3.782158e-51
## 33 LINCSOE_14510  -0.436162 1.191284e-50

## Selecting by v1
## Selecting by v2

## Selecting by v1

## Selecting by v2

## [1] "For signature: LINCSOE_14566 calculated extreme correlation is:-0.465418341250644 ilincs query r

## Selecting by v1
## Selecting by v2

## Selecting by v1

## Selecting by v2

## [1] "For signature: LINCSOE_14609 calculated extreme correlation is:-0.456740871487361 ilincs query r

## Selecting by v1
## Selecting by v2

## Selecting by v1

## Selecting by v2

## [1] "For signature: LINCSOE_14459 calculated extreme correlation is:-0.445713058681 ilincs query resu

## Selecting by v1
## Selecting by v2

## Selecting by v1

## Selecting by v2

## [1] "For signature: LINCSOE_14354 calculated extreme correlation is:-0.440384645021 ilincs query resu

## Selecting by v1
## Selecting by v2

## Selecting by v1

## Selecting by v2

## [1] "For signature: LINCSOE_14501 calculated extreme correlation is:-0.438327669276549 ilincs query r
## [1] "================================================================"
## [1] "get connected signatures based on positive and negative gene list"
## [1] "================================================================"
## [1] "=====================================================================================================
## [1] "checking the accuracy of positive and negative gene list correlation results via offline benchma
## [1] "=====================================================================================================
## [1] "For signature: LINCSCP_226446 calculated correlation is:0.922165180692982 ilincs query results r
## [1] "For signature: LINCSCP_116767 calculated correlation is:-0.916754438583011 ilincs query results
## [1] "For signature: LINCSCP_218419 calculated correlation is:0.915779793612196 ilincs query results r
## [1] "For signature: LINCSCP_225178 calculated correlation is:-0.912452260708974 ilincs query results
## [1] "For signature: LINCSCP_225252 calculated correlation is:-0.91238268705273 ilincs query results r
## [1] "+++++++++++++++++++++ Finished +++++++++++++++++++++"
```

```
## [1] "LINCSOE_2412"
## [1] "download signature ======================================"
## [1] "============================================="
## [1] "get connected signatures based on signatureid"
## [1] "============================================="

## No encoding supplied: defaulting to UTF-8.

##      signatureid similarity       pValue
## 4   LINCSOE_2308  -0.375948 3.688621e-38
## 7   LINCSOE_2332  -0.324840 1.949763e-29
## 9   LINCSOE_2366  -0.322762 4.086119e-29
## 14  LINCSOE_2209  -0.284247 1.334676e-23
## 21  LINCSOE_2416  -0.250404 2.042785e-19
## 22 LINCSOE_19749  -0.243907 1.112536e-18
## [1] "=============================================================================="
## [1] "get connected signatures based on the downloaded signature with p value"
## [1] "=============================================================================="
## [1] "processedSig_Thu_Jan_28_11_47_01_2021_6117843.xls"
##      signatureid similarity       pValue
## 1:  LINCSOE_2308 -0.4993890 8.123119e-63
## 2:  LINCSOE_2366 -0.4787053 3.668075e-57
## 3:  LINCSOE_2416 -0.3917117 3.229937e-37
## 4: LINCSOE_19749 -0.3628268 8.496418e-32
## 5:  LINCSOE_3085 -0.3437366 1.656081e-28
## 6: LINCSOE_20074 -0.3430669 2.140078e-28
## [1] "==============================================================================================
## [1] "checking the accuracy of results via offline benchmarking on the downloaded complete signature w
## [1] "==============================================================================================
## [1] "For signature: LINCSOE_2308 calculated weighted correlation is:-0.499388952379253 ilincs query i
## [1] "For signature: LINCSOE_2366 calculated weighted correlation is:-0.478705296779514 ilincs query i
## [1] "For signature: LINCSOE_2416 calculated weighted correlation is:-0.391711682078742 ilincs query i
## [1] "For signature: LINCSOE_19749 calculated weighted correlation is:-0.362826782461146 ilincs query
## [1] "For signature: LINCSOE_3085 calculated weighted correlation is:-0.343736592462957 ilincs query i
## [1] "================================================================================="
## [1] "get connected signatures based on the downloaded signature without p value"
## [1] "================================================================================="
## [1] "processedSig_Thu_Jan_28_11_47_20_2021_7391107.xls"
##      signatureid similarity       pValue
## 1:  LINCSOE_2308 -0.5071566 4.830079e-65
## 2:  LINCSOE_2366 -0.4783492 4.554654e-57
## 3: LINCSOE_19749 -0.4131607 1.317358e-41
## 4:  LINCSOE_2416 -0.3997218 8.081060e-39
## 5:  LINCSOE_2317 -0.3555089 1.648386e-30
## 6:  LINCSOE_5469 -0.3511174 9.415677e-30
## [1] "==============================================================================================
## [1] "checking the accuracy of results via offline benchmarking on the downloaded complete signature w
## [1] "==============================================================================================
## [1] "For signature: LINCSOE_2308 calculated weighted correlation is:-0.507156514660143 ilincs query i
## [1] "For signature: LINCSOE_2366 calculated weighted correlation is:-0.478349113504682 ilincs query i
## [1] "For signature: LINCSOE_19749 calculated weighted correlation is:-0.41316067165358 ilincs query i
## [1] "For signature: LINCSOE_2416 calculated weighted correlation is:-0.399721754533265 ilincs query i
## [1] "For signature: LINCSOE_2317 calculated weighted correlation is:-0.355508917090654 ilincs query i
## [1] "==============================================================================================
## [1] "checking the accuracy of extreme correlation results via offline benchmarking on the submitted s
```

```
## [1] "==========================================================================================
##      signatureid similarity       pValue
## 4   LINCSOE_2308  -0.375948 3.688621e-38
## 7   LINCSOE_2332  -0.324840 1.949763e-29
## 9   LINCSOE_2366  -0.322762 4.086119e-29
## 14  LINCSOE_2209  -0.284247 1.334676e-23
## 21  LINCSOE_2416  -0.250404 2.042785e-19
## 22 LINCSOE_19749  -0.243907 1.112536e-18

## Selecting by v1
## Selecting by v2

## Selecting by v1

## Selecting by v2

## [1] "For signature: LINCSOE_2308 calculated extreme correlation is:-0.375947582784689 ilincs query re

## Selecting by v1
## Selecting by v2

## Selecting by v1

## Selecting by v2

## [1] "For signature: LINCSOE_2332 calculated extreme correlation is:-0.324839562831783 ilincs query re

## Selecting by v1
## Selecting by v2

## Selecting by v1

## Selecting by v2

## [1] "For signature: LINCSOE_2366 calculated extreme correlation is:-0.322761662968741 ilincs query re

## Selecting by v1
## Selecting by v2

## Selecting by v1

## Selecting by v2

## [1] "For signature: LINCSOE_2209 calculated extreme correlation is:-0.284246891188749 ilincs query re

## Selecting by v1
## Selecting by v2

## Selecting by v1

## Selecting by v2

## [1] "For signature: LINCSOE_2416 calculated extreme correlation is:-0.250404448376141 ilincs query re
## [1] "================================================================"
## [1] "get connected signatures based on positive and negative gene list"
## [1] "================================================================"
## [1] "==========================================================================================
## [1] "checking the accuracy of positive and negative gene list correlation results via offline benchma
## [1] "==========================================================================================
## [1] "For signature: LINCSCP_1906 calculated correlation is:-0.702302139740545 ilincs query results is
## [1] "For signature: LINCSCP_46568 calculated correlation is:-0.690776387614032 ilincs query results i
## [1] "For signature: LINCSCP_68961 calculated correlation is:-0.686072608554865 ilincs query results i
## [1] "For signature: LINCSCP_43829 calculated correlation is:-0.684227924624064 ilincs query results i
## [1] "For signature: LINCSCP_52629 calculated correlation is:-0.67804032252688 ilincs query results is
```

```
## [1] "++++++++++++++++++++++ Finished ++++++++++++++++++++++"
```
