# Supplementary material for: Connecting omics signatures and revealing biological mechanisms with iLINCS
Source: Nat Commun. 2022 Aug 9;13:4678. doi: 10.1038/s41467-022-32205-3 (PMC9362980; doi:10.1038/s41467-022-32205-3)

iLINCS API R Notebook

#Loading packages

#Setting default width option

Display Signature Libraries

```
apiUrl <- "http://www.ilincs.org/api/SignatureLibraries"
req <- GET(apiUrl)
json <- httr::content(req, as = "text")
ilincs_libraries <- fromJSON(json)
ilincs_libraries[,c("libraryID","libraryName")]
```

```
##    libraryID                                         libraryName
## 1      LIB_1                           Disease related signatures
## 2     LIB_10                Cancer therapeutics response signatures
## 3     LIB_11                  LINCS gene overexpression signatures
## 4     LIB_12                                DrugMatrix signatures
## 5     LIB_13 Transcriptional signatures from EBI Expression Atlas
## 6     LIB_14           Pharmacogenomics transcriptional signatures
## 7      LIB_2                          Connectivity Map signatures
## 8      LIB_3         ENCODE transcription factor binding signatures
## 9      LIB_5                LINCS chemical perturbagen signatures
## 10     LIB_6       LINCS consensus gene (CGS) knockdown signatures
## 11     LIB_8                             LINCS RNA-Seq signatures
## 12     LIB_9                  LINCS targeted proteomics signatures
```

Searching for MTOR CRIPR genetic loss of function (CGS) perturbation signatures in the MCF7 cell line

```
term <- "MTOR"
ilincs_libId<-"LIB_6"
apiUrl <- paste("http://www.ilincs.org/api/SignatureMeta/findTermWithSynonyms?term=",term,"&library=",il
req <- GET(apiUrl)

mtorCgs<-fromJSON(httr::content(req,type="text"))$data
```

```
## No encoding supplied: defaulting to UTF-8.
```

```
mtorCgsMcf7<-mtorCgs[intersect(grep("MCF7",mtorCgs$cellline),grep("trt_xpr.cgs",mtorCgs$pert_type)),]
mtorCgsMcf7[,c("cellline","time","treatment","signatureid","pert_type")]
```

```
##    cellline time treatment   signatureid   pert_type
## 26 MCF7.101 96 h      MTOR LINCSKD_33763 trt_xpr.cgs
## 27 MCF7.311 96 h      MTOR LINCSKD_33816 trt_xpr.cgs
```

Finding and summarizing connected CGSes to the first MTOR CRISPR CGS (LINCSKD_33763)

Finding connected CGSes

```
ilincs_signatureId <- mtorCgsMcf7$signatureid[1]

apiUrl <- paste("http://www.ilincs.org/api/SignatureMeta/findConcordantSignatures?sigID=",ilincs_signatu
req <- GET(apiUrl)

connectedCgs<-fromJSON(httr::content(req,type="text"))
```

```
## No encoding supplied: defaulting to UTF-8.
```

```
head(connectedCgs[,c("signatureid","similarity","pValue","cellline","treatment")])
```

```
##     signatureid similarity       pValue cellline treatment
## 1 LINCSKD_33768   0.361049 4.719053e-36 MCF7.101    PIK3CA
## 2 LINCSKD_33816   0.356419 3.071392e-35 MCF7.311      MTOR
## 3 LINCSKD_33710   0.289951 5.716644e-25 HT29.311      MTOR
## 4 LINCSKD_33821   0.270321 2.072215e-22 MCF7.311    PIK3CA
## 5 LINCSKD_33445   0.260006 3.801609e-21 A375.311      MTOR
## 6 LINCSKD_33657   0.251621 3.687196e-20 HELA.311      MTOR
```

Summary boxplot, Figure 2A in the manusript

```
geneFreq <- sort(table(connectedCgs$treatment[1:100]),decreasing=TRUE)[5:1]
geneFreqDf<-data.frame(gene=factor(names(geneFreq),levels=names(geneFreq),ordered=T),geneFreq=as.vector

tp100cgs<-ggplot(data=geneFreqDf,aes(x=gene,y=geneFreq)) +
  geom_bar(stat="identity",fill="steelblue") +
  coord_flip() + theme_bw() +
  theme(axis.ticks=element_line(color="grey80"),text=element_text(size=20),legend.key.size=unit(1,"cm")
  labs(x="",y="Number of Signatures")
print(tp100cgs)
```

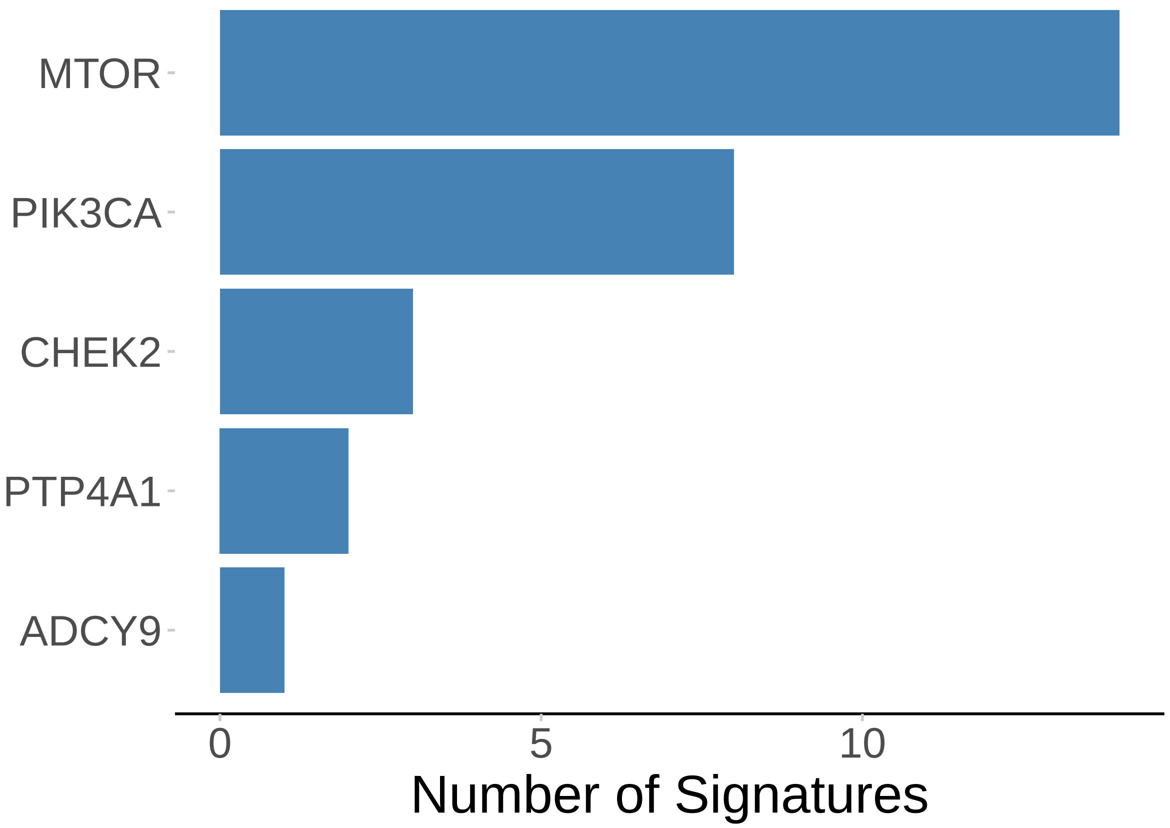

Finding and summarizing connected chemical perturbage (CP) signatures to MTOR CRISPR CGS (LINCSKD_33763)

Finding connected CPs

```
ilincs_libId<-"LIB_5"

apiUrl <- paste("http://www.ilincs.org/api/SignatureMeta/findConcordantSignatures?sigID=",ilincs_signatu
req <- GET(apiUrl)

connectedCps<-fromJSON(httr::content(req,type="text"))
```

```
## No encoding supplied: defaulting to UTF-8.
```

```
head(connectedCps)
```

```
##      signatureid similarity significance       pValue nGenes factor        compound concentration cell
## 1 LINCSCP_137891   0.319918      29.1413 7.222069e-30    978     NA      Everolimus        0.04uM
## 2 LINCSCP_141783   0.315187      28.4269 3.741927e-29    978     NA SCHEMBL6851809        0.12uM
## 3 LINCSCP_137889   0.314463      28.3185 4.802370e-29    978     NA      Everolimus        0.37uM
## 4 LINCSCP_141782   0.300157      26.2408 5.744227e-27    978     NA SCHEMBL6851809        0.37uM
## 5  LINCSCP_38560   0.295942      25.6504 2.236856e-26    978     NA       Sirolimus        3.33uM
## 6 LINCSCP_143289   0.293372      25.2952 5.067947e-26    978     NA         AZD2014        0.12uM
##                        lincsSigID is_exemplar GeneTargets
## 1              REP.A010_MCF7_24H:H06          NA FKBP1A|MTOR
```

```
## 2                 REP.A021_MCF7_24H:E05          NA          MTOR
## 3                 REP.A010_MCF7_24H:H04          NA FKBP1A|MTOR
## 4                 REP.A021_MCF7_24H:E04          NA          MTOR
## 5 PCLB002_MCF7_24H:BRD-K84937637:3.33           0 FKBP1A|MTOR
## 6                 REP.A025_MCF7_24H:H05          NA          MTOR
```

Summary boxplot, Figure 2B in the manusript

```
top100GeneTargets<-unlist(strsplit(connectedCps$GeneTargets[1:100],split="\\|"))
geneFreq <- sort(table(top100GeneTargets),decreasing=TRUE)[5:1]
geneFreqDf<-data.frame(gene=factor(names(geneFreq),levels=names(geneFreq),ordered=T),geneFreq=as.vector

tp100cps<-ggplot(data=geneFreqDf,aes(x=gene,y=geneFreq)) +
  geom_bar(stat="identity",fill="steelblue") +
  coord_flip() + theme_bw() +
  theme(axis.ticks=element_line(color="grey80"),text=element_text(size=20),legend.key.size=unit(1,"cm")
  labs(x="",y="Number of Signatures")
tp100cps
```

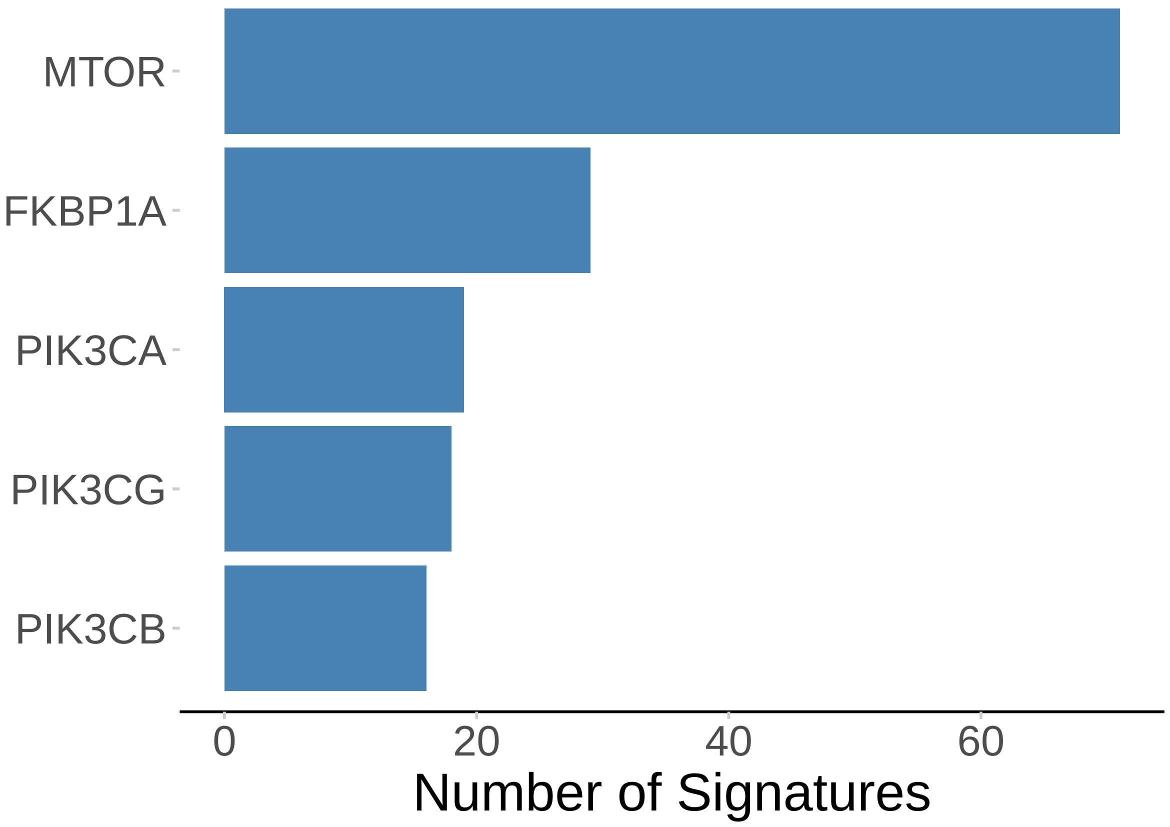

Supplement: Supplementary file 8 — Software 1 [file 41467_2022_32205_MOESM8_ESM.zip › ilincsAPI-master/useCases/useCase1a.pdf]
