# Supplementary material for: Connecting omics signatures and revealing biological mechanisms with iLINCS
Source: Nat Commun. 2022 Aug 9;13:4678. doi: 10.1038/s41467-022-32205-3 (PMC9362980; doi:10.1038/s41467-022-32205-3)
Supplement: Supplementary file 8 — Software 1 [file 41467_2022_32205_MOESM8_ESM.zip › ilincsAPI-master/useCases/useCase1b.html]

iLINCS API R Notebook


# iLINCS API R Notebook

#Loading packages

#Setting default width option

# Display Signature Libraries

```
apiUrl <- "http://www.ilincs.org/api/SignatureLibraries"
req <- GET(apiUrl)
json <- httr::content(req, as = "text")
ilincs_libraries <- fromJSON(json)
ilincs_libraries[,c("libraryID","libraryName")]
```

# Searching for Everolimus chemical perturbage (CP) perturbation signatures in the MCF7 cell line

```
term <- "Everolimus"
ilincs_libId<-"LIB_5"
apiUrl <- paste("http://www.ilincs.org/api/SignatureMeta/findTermWithSynonyms?term=",term,"&library=",ilincs_libId,sep="")
req <- GET(apiUrl)

everolimusCps<-fromJSON(httr::content(req,type="text"))$data
```

```
## No encoding supplied: defaulting to UTF-8.
```

```
everolimusCpsMcf7<-everolimusCps[intersect(grep("Everolimus",everolimusCps$compound),grep("MCF7",everolimusCps$cellline)),]
everolimusCpsMcf7[,c("compound","time","concentration","signatureid","cellline")]
```

# Enrichr analysis of everolimus signature at 0.04uM concentration (LINCSCP\_137891) (Fig 2C)

```
ilincs_signatureId <- "LINCSCP_137891"

req <- POST("http://www.ilincs.org/api/ilincsR/downloadSignature", body = list(sigID = ilincs_signatureId, display = FALSE), encode = "json")
ilincs_sessionId<-unlist(httr::content(req))
ilincs_sessionId
```

```
##                                 data 
## "sig_Sat_May__7_22_13_30_2022_27789"
```

```
signatureFileUrl=paste("http://www.ilincs.org/tmp/",ilincs_sessionId,".xls",sep="")
everolimusSignatureData<-read.table(signatureFileUrl,sep="\t",header=T,stringsAsFactors = F)
sigGenes100<-everolimusSignatureData$Name_GeneSymbol[order(everolimusSignatureData$Significance_pvalue)[1:100]]

setEnrichrSite("Enrichr")
```

```
## Connection changed to https://maayanlab.cloud/Enrichr/
```

```
## Connection is Live!
```

```
enriched <- enrichr(genes=sigGenes100, databases="KEGG_2019_Human")
```

```
## Uploading data to Enrichr... Done.
##   Querying KEGG_2019_Human... Done.
## Parsing results... Done.
```

```
enriched[["KEGG_2019_Human"]][1:5,]
```

# Finding and summarizing connected CGSes with the Everolimus signature at 0.04uM concentration (LINCSCP\_137891)

## Finding connected CGSes

```
ilincs_libId<-"LIB_6"

apiUrl <- paste("http://www.ilincs.org/api/SignatureMeta/findConcordantSignatures?sigID=",ilincs_signatureId,"&lib=",ilincs_libId,sep="")
req <- GET(apiUrl)

connectedCgs<-fromJSON(httr::content(req,type="text"))
```

```
## No encoding supplied: defaulting to UTF-8.
```

```
head(connectedCgs[,c("signatureid","similarity","pValue","cellline","treatment")])
```

## Summary boxplot, Figure 2D in the manuscript

```
geneFreq <- sort(table(connectedCgs$treatment[1:100]),decreasing=TRUE)[5:1]
geneFreqDf<-data.frame(gene=factor(names(geneFreq),levels=names(geneFreq),ordered=T),geneFreq=as.vector(geneFreq))

tp100cgs<-ggplot(data=geneFreqDf,aes(x=gene,y=geneFreq)) + 
  geom_bar(stat="identity",fill="steelblue") + 
  coord_flip() + theme_bw() +
  theme(axis.ticks=element_line(color="grey80"),text=element_text(size=20),legend.key.size=unit(1,"cm"),axis.line.x=element_line(colour="black"),panel.grid.major=element_blank(),panel.grid.minor=element_blank(),panel.border=element_blank(),panel.background=element_blank()) +
  labs(x="",y="Number of Signatures")
tp100cgs
```

## Enrichr analysis of connected CGSes (Fig 2D)

```
setEnrichrSite("Enrichr")
```

```
## Connection changed to https://maayanlab.cloud/Enrichr/
```

```
## Connection is Live!
```

```
enrichedCgsTargets <- enrichr(genes=unique(connectedCgs$treatment), databases="KEGG_2019_Human")
```

```
## Uploading data to Enrichr... Done.
##   Querying KEGG_2019_Human... Done.
## Parsing results... Done.
```

```
enrichedCgsTargets[["KEGG_2019_Human"]][1:5,]
```

# Finding and summarizing connected chemical perturbage (CP) signatures with the Everolimus signature at 0.04uM concentration (LINCSCP\_137891)

## Finding connected CPs

```
ilincs_libId<-"LIB_5"

apiUrl <- paste("http://www.ilincs.org/api/SignatureMeta/findConcordantSignatures?sigID=",ilincs_signatureId,"&lib=",ilincs_libId,sep="")
req <- GET(apiUrl)

connectedCps<-fromJSON(httr::content(req,type="text"))
```

```
## No encoding supplied: defaulting to UTF-8.
```

```
head(connectedCps)
```

## Summary boxplot, Figure 2E in the manusript

```
top100GeneTargets<-unlist(strsplit(connectedCps$GeneTargets[1:100],split="\\|"))
geneFreq <- sort(table(top100GeneTargets),decreasing=TRUE)[5:1]
geneFreqDf<-data.frame(gene=factor(names(geneFreq),levels=names(geneFreq),ordered=T),geneFreq=as.vector(geneFreq))

tp100cp<-ggplot(data=geneFreqDf,aes(x=gene,y=geneFreq)) + 
  geom_bar(stat="identity",fill="steelblue") + 
  coord_flip() + theme_bw() +
  theme(axis.ticks=element_line(color="grey80"),text=element_text(size=20),legend.key.size=unit(1,"cm"),axis.line.x=element_line(colour="black"),panel.grid.major=element_blank(),panel.grid.minor=element_blank(),panel.border=element_blank(),panel.background=element_blank()) +
  labs(x="",y="Number of Signatures")
tp100cp
```

## Enrichr analysis of connected CGSes (Fig 2E)

```
setEnrichrSite("Enrichr")
```

```
## Connection changed to https://maayanlab.cloud/Enrichr/
```

```
## Connection is Live!
```

```
enrichedCgsTargets <- enrichr(genes=unique(top100GeneTargets), databases="KEGG_2019_Human")
```

```
## Uploading data to Enrichr... Done.
##   Querying KEGG_2019_Human... Done.
## Parsing results... Done.
```

```
enrichedCgsTargets[["KEGG_2019_Human"]][1:5,]
```
