# Supplementary material for: Connecting omics signatures and revealing biological mechanisms with iLINCS
Source: Nat Commun. 2022 Aug 9;13:4678. doi: 10.1038/s41467-022-32205-3 (PMC9362980; doi:10.1038/s41467-022-32205-3)
Supplement: Supplementary file 8 — Software 1 [file 41467_2022_32205_MOESM8_ESM.zip › ilincsAPI-master/useCases/useCase1b.pdf]

iLINCS API R Notebook

#Loading packages

#Setting default width option

Display Signature Libraries

```
apiUrl <- "http://www.ilincs.org/api/SignatureLibraries"
req <- GET(apiUrl)
json <- httr::content(req, as = "text")
ilincs_libraries <- fromJSON(json)
ilincs_libraries[,c("libraryID","libraryName")]
```

```
##    libraryID                                  libraryName
## 1      LIB_1                   Disease related signatures
## 2     LIB_10           Cancer therapeutics response signatures
## 3     LIB_11              LINCS gene overexpression signatures
## 4     LIB_12                         DrugMatrix signatures
## 5     LIB_13 Transcriptional signatures from EBI Expression Atlas
## 6     LIB_14       Pharmacogenomics transcriptional signatures
## 7      LIB_2                    Connectivity Map signatures
## 8      LIB_3        ENCODE transcription factor binding signatures
## 9      LIB_5             LINCS chemical perturbagen signatures
## 10     LIB_6       LINCS consensus gene (CGS) knockdown signatures
## 11     LIB_8                        LINCS RNA-Seq signatures
## 12     LIB_9              LINCS targeted proteomics signatures
```

Searching for Everolimus chemical perturbage (CP) perturbation signatures in the MCF7 cell line

```
term <- "Everolimus"
ilincs_libId<-"LIB_5"
apiUrl <- paste("http://www.ilincs.org/api/SignatureMeta/findTermWithSynonyms?term=",term,"&library=",i[illegible]
req <- GET(apiUrl)

everolimusCps<-fromJSON(httr::content(req,type="text"))$data
```

```
## No encoding supplied: defaulting to UTF-8.
```

```
everolimusCpsMcf7<-everolimusCps[intersect(grep("Everolimus",everolimusCps$compound),grep("MCF7",everol[illegible]
everolimusCpsMcf7[,c("compound","time","concentration","signatureid","cellline")]
```

```
##      compound time concentration    signatureid cellline
## 61  Everolimus  24h          10uM LINCSCP_137886     MCF7
## 62  Everolimus  24h        3.33uM LINCSCP_137887     MCF7
## 63  Everolimus  24h        1.11uM LINCSCP_137888     MCF7
## 64  Everolimus  24h        0.37uM LINCSCP_137889     MCF7
```

```
## 65  Everolimus  24h        0.12uM LINCSCP_137890     MCF7
## 66  Everolimus  24h        0.04uM LINCSCP_137891     MCF7
## 132 Everolimus  24h          10uM  LINCSCP_32458     MCF7
## 133 Everolimus   6h          10uM  LINCSCP_32655     MCF7
```

Enrichr analysis of everolimus signature at 0.04uM concentration (LINCSCP_137891) (Fig 2C)

```
ilincs_signatureId <- "LINCSCP_137891"

req <- POST("http://www.ilincs.org/api/ilincsR/downloadSignature", body = list(sigID = ilincs_signatureI
ilincs_sessionId<-unlist(httr::content(req))
ilincs_sessionId
```

```
##                                   data
## "sig_Sat_May__7_22_13_30_2022_27789"
```

```
signatureFileUrl=paste("http://www.ilincs.org/tmp/",ilincs_sessionId,".xls",sep="")
everolimusSignatureData<-read.table(signatureFileUrl,sep="\t",header=T,stringsAsFactors = F)
sigGenes100<-everolimusSignatureData$Name_GeneSymbol[order(everolimusSignatureData$Significance_pvalue)

setEnrichrSite("Enrichr")
```

```
## Connection changed to https://maayanlab.cloud/Enrichr/
```

```
## Connection is Live!
```

```
enriched <- enrichr(genes=sigGenes100, databases="KEGG_2019_Human")
```

```
## Uploading data to Enrichr... Done.
##   Querying KEGG_2019_Human... Done.
## Parsing results... Done.
```

```
enriched[["KEGG_2019_Human"]][1:5,]
```

```
##                                         Term Overlap      P.value Adjusted.P.value Old.P.value Old.Ad
## 1                          Colorectal cancer    6/86 4.570116e-06     0.0007677795           0
## 2 Valine, leucine and isoleucine degradation    4/48 9.668983e-05     0.0081219461           0
## 3              Terpenoid backbone biosynthesis    3/22 1.743100e-04     0.0085224485           0
## 4                         Endometrial cancer    4/58 2.029154e-04     0.0085224485           0
## 5                                 Cell cycle   5/124 3.974780e-04     0.0096239910           0
```

Finding and summarizing connected CGSes with the Everolimus signature at 0.04uM concentration (LINCSCP_137891)

Finding connected CGSes

```
ilincs_libId<-"LIB_6"

apiUrl <- paste("http://www.ilincs.org/api/SignatureMeta/findConcordantSignatures?sigID=",ilincs_signatu
req <- GET(apiUrl)

connectedCgs<-fromJSON(httr::content(req,type="text"))
```

```
## No encoding supplied: defaulting to UTF-8.
```

```r
head(connectedCgs[,c("signatureid","similarity","pValue","cellline","treatment")])
```

```
##     signatureid similarity       pValue cellline treatment
## 1 LINCSKD_33816   0.355679 1.074249e-35 MCF7.311      MTOR
## 2 LINCSKD_33763   0.319918 7.222069e-30 MCF7.101      MTOR
## 3 LINCSKD_33922   0.304985 1.177066e-27  PC3.311      MTOR
## 4 LINCSKD_33869   0.297908 1.189836e-26  PC3.101      MTOR
## 5 LINCSKD_33710   0.297193 1.497679e-26 HT29.311      MTOR
## 6 LINCSKD_33975   0.296891 1.649948e-26 YAPC.311      MTOR
```

Summary boxplot, Figure 2D in the manuscript

```r
geneFreq <- sort(table(connectedCgs$treatment[1:100]),decreasing=TRUE)[5:1]
geneFreqDf<-data.frame(gene=factor(names(geneFreq),levels=names(geneFreq),ordered=T),geneFreq=as.vector

tp100cgs<-ggplot(data=geneFreqDf,aes(x=gene,y=geneFreq)) +
  geom_bar(stat="identity",fill="steelblue") +
  coord_flip() + theme_bw() +
  theme(axis.ticks=element_line(color="grey80"),text=element_text(size=20),legend.key.size=unit(1,"cm")
  labs(x="",y="Number of Signatures")
tp100cgs
```

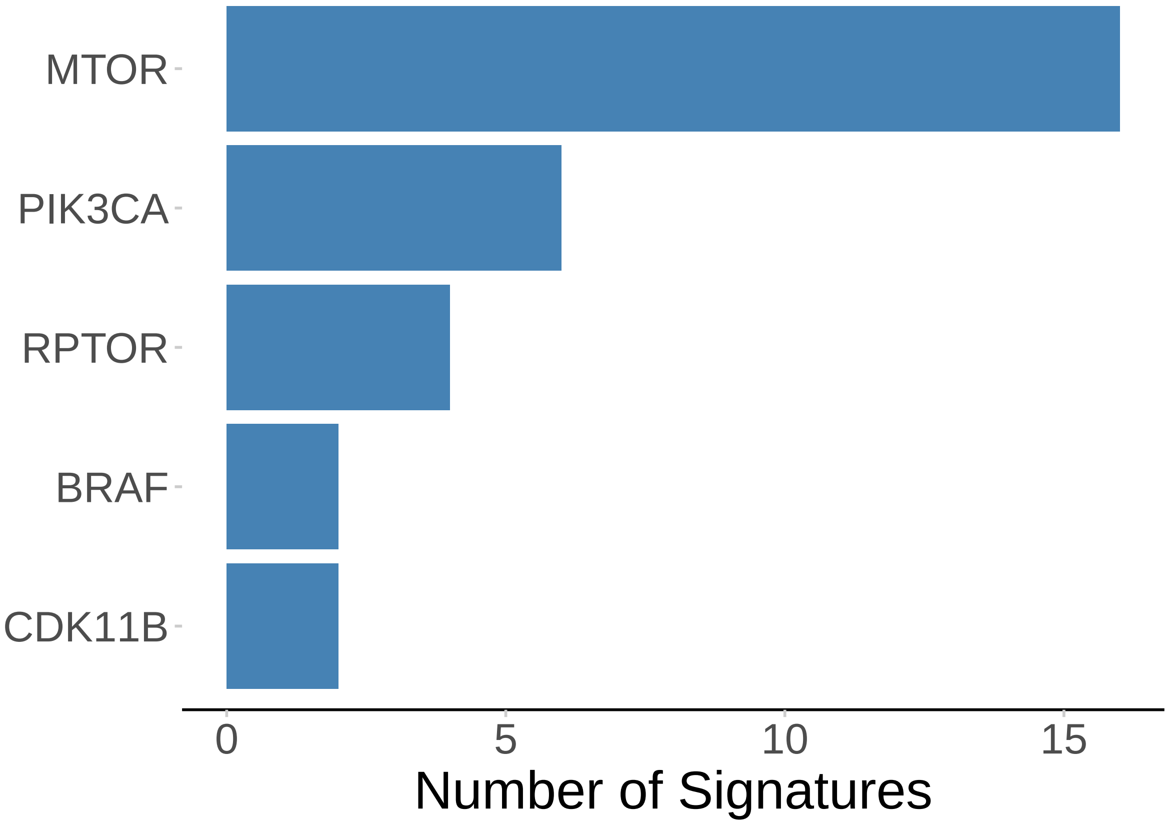

Enrichr analysis of connected CGSes (Fig 2D)

```r
setEnrichrSite("Enrichr")
```

```
## Connection changed to https://maayanlab.cloud/Enrichr/
```

```
## Connection is Live!
```

```r
enrichedCgsTargets <- enrichr(genes=unique(connectedCgs$treatment), databases="KEGG_2019_Human")
```

```
## Uploading data to Enrichr... Done.
##   Querying KEGG_2019_Human... Done.
## Parsing results... Done.
```

```r
enrichedCgsTargets[["KEGG_2019_Human"]][1:5,]
```

```
##                                 Term Overlap      P.value Adjusted.P.value Old.P.value Old.Adjusted.P
## 1            mTOR signaling pathway  14/152 3.917992e-15     7.169925e-13           0
## 2                 Colorectal cancer    9/86 1.276902e-10     9.035277e-09           0
## 3               Endometrial cancer    8/58 1.481193e-10     9.035277e-09           0
## 4                        Autophagy  10/128 2.129479e-10     9.742369e-09           0
## 5 Thyroid hormone signaling pathway   9/116 1.904802e-09     6.971575e-08           0
##                                                                   Genes
## 1 MAP2K1;FZD5;PDPK1;FZD4;STRADB;MAPKAP1;BRAF;MTOR;RPTOR;PIK3CA;RHEB;AKT1;RICTOR;EIF4E
## 2                                   MAP2K1;PIK3CA;MYC;AKT1;PMAIP1;BRAF;TP53;MTOR;BBC3
## 3                                          MAP2K1;PIK3CA;PDPK1;MYC;ILK;AKT1;BRAF;TP53
## 4                           RPTOR;BECN1;MAP2K1;PIK3CA;PDPK1;RHEB;AKT1;PIK3C3;ATG7;MTOR
## 5                                  MAP2K1;PIK3CA;PDPK1;RHEB;MYC;AKT1;ATP1A3;TP53;MTOR
```

Finding and summarizing connected chemical perturbage (CP) signatures with the Everolimus signature at 0.04uM concentration (LINCSCP_137891)

Finding connected CPs

```r
ilincs_libId<-"LIB_5"

apiUrl <- paste("http://www.ilincs.org/api/SignatureMeta/findConcordantSignatures?sigID=",ilincs_signatu
req <- GET(apiUrl)

connectedCps<-fromJSON(httr::content(req,type="text"))
```

```
## No encoding supplied: defaulting to UTF-8.
```

```r
head(connectedCps)
```

```
##      signatureid similarity significance        pValue nGenes factor   compound concentration cellli
## 1 LINCSCP_137889   0.691327     144.3390 4.580566e-145    978     NA Everolimus        0.37uM     MC
## 2 LINCSCP_137888   0.671113     133.4310 3.705507e-134    978     NA Everolimus        1.11uM     MC
## 3 LINCSCP_137886   0.619745     109.2560 5.550003e-110    978     NA Everolimus          10uM     MC
## 4 LINCSCP_143132   0.606843     103.8700 1.348646e-104    978     NA  Sirolimus        0.37uM     MC
## 5 LINCSCP_143130   0.594678      99.0135 9.692821e-100    978     NA  Sirolimus        3.33uM     MC
## 6 LINCSCP_143133   0.574759      91.4952  3.197302e-92    978     NA  Sirolimus        0.12uM     MC
##   is_exemplar GeneTargets
## 1          NA FKBP1A|MTOR
```

```
## 2          NA FKBP1A|MTOR
## 3          NA FKBP1A|MTOR
## 4          NA FKBP1A|MTOR
## 5          NA FKBP1A|MTOR
## 6          NA FKBP1A|MTOR
```

Summary boxplot, Figure 2E in the manusript

```
top100GeneTargets<-unlist(strsplit(connectedCps$GeneTargets[1:100],split="\\|"))
geneFreq <- sort(table(top100GeneTargets),decreasing=TRUE)[5:1]
geneFreqDf<-data.frame(gene=factor(names(geneFreq),levels=names(geneFreq),ordered=T),geneFreq=as.vector

tp100cp<-ggplot(data=geneFreqDf,aes(x=gene,y=geneFreq)) +
  geom_bar(stat="identity",fill="steelblue") +
  coord_flip() + theme_bw() +
  theme(axis.ticks=element_line(color="grey80"),text=element_text(size=20),legend.key.size=unit(1,"cm")
  labs(x="",y="Number of Signatures")
tp100cp
```

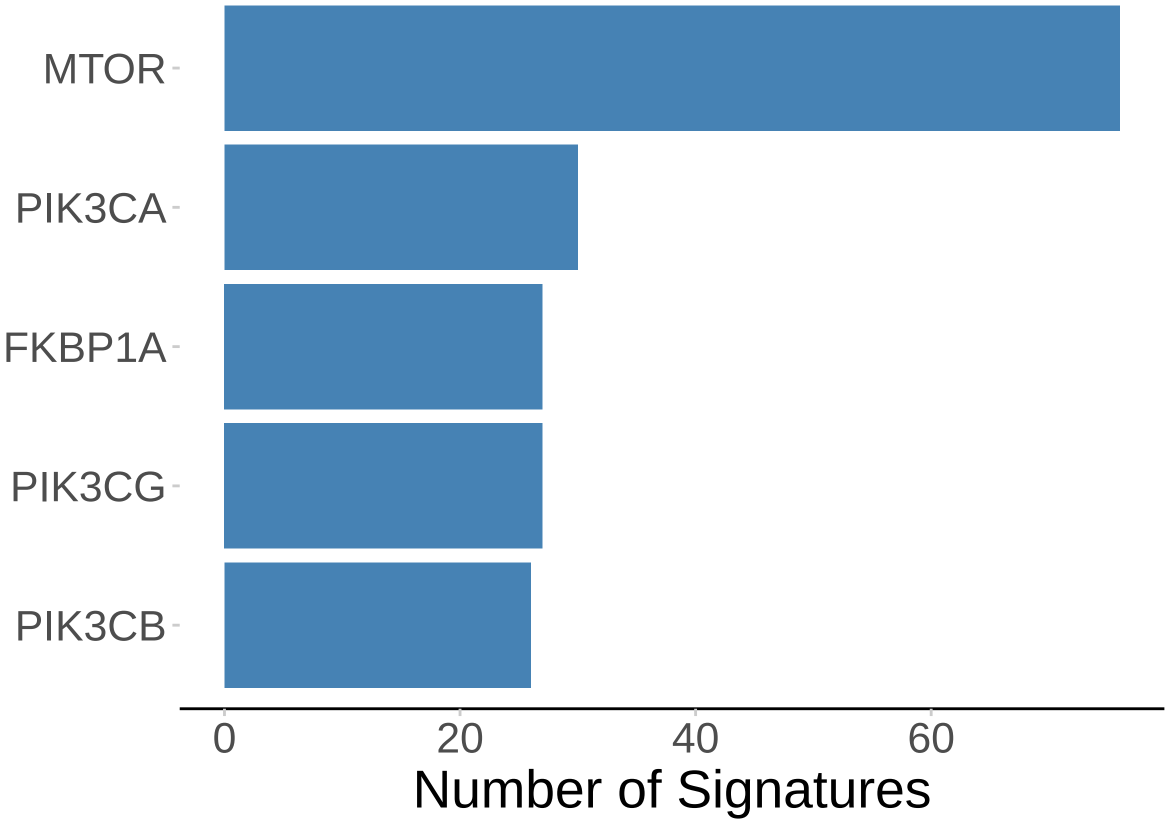

Enrichr analysis of connected CGSes (Fig 2E)

```
setEnrichrSite("Enrichr")
```

```
## Connection changed to https://maayanlab.cloud/Enrichr/
```

```
## Connection is Live!
```

```r
enrichedCgsTargets <- enrichr(genes=unique(top100GeneTargets), databases="KEGG_2019_Human")
```

```
## Uploading data to Enrichr... Done.
##   Querying KEGG_2019_Human... Done.
## Parsing results... Done.
```

```r
enrichedCgsTargets[["KEGG_2019_Human"]][1:5,]
```

```
##                                 Term Overlap      P.value Adjusted.P.value Old.P.value Old.Adjusted.P
## 1           HIF-1 signaling pathway  14/100 1.858086e-27     1.497395e-25           0
## 2 Phospholipase D signaling pathway  15/148 2.454745e-27     1.497395e-25           0
## 3                            Glioma   13/75 1.072013e-26     4.359521e-25           0
## 4       PI3K-Akt signaling pathway  17/354 1.024631e-25     2.772415e-24           0
## 5           FoxO signaling pathway  14/132 1.136236e-25     2.772415e-24           0
##                                                                                                  Ge
## 1                    INSR;PIK3CD;PIK3R3;PIK3R2;PIK3CB;PIK3R1;MTOR;EGFR;IGF1R;PIK3CA;AKT2;AKT3;MKNK2;A
## 2             INSR;PIK3CD;PIK3R3;PIK3R2;PIK3CB;PIK3R1;MTOR;PIK3CG;EGFR;PIK3R5;PIK3CA;AKT2;AKT3;AKT1;R
## 3                          PIK3CD;PIK3R3;PIK3R2;PIK3CB;PIK3R1;MTOR;EGFR;IGF1R;PIK3CA;AKT2;AKT3;AKT1;R
## 4 INSR;PIK3CD;PIK3R3;PIK3R2;PIK3CB;PIK3R1;MTOR;PIK3CG;EGFR;PIK3R5;IGF1R;PIK3CA;AKT2;AKT3;AKT1;RAF1;J
## 5                    INSR;PLK1;PIK3CD;PIK3R3;PIK3R2;PIK3CB;PIK3R1;EGFR;IGF1R;PIK3CA;AKT2;AKT3;AKT1;R
```
