# Supplementary material for: Connecting omics signatures and revealing biological mechanisms with iLINCS
Source: Nat Commun. 2022 Aug 9;13:4678. doi: 10.1038/s41467-022-32205-3 (PMC9362980; doi:10.1038/s41467-022-32205-3)
Supplement: Supplementary file 8 — Software 1 [file 41467_2022_32205_MOESM8_ESM.zip › ilincsAPI-master/useCases/useCase2.html]

iLINCS API R Notebook


# iLINCS API R Notebook

# Analyzing TCGA breast cancer proteomics RPPA dataset

## Searching for the dataset in the TCGA collection (ie portal)

```
portal<-"TCGA"
searchTerm<-"BRCA"
dataType<-"Proteomics"
apiUrl <- paste0("http://www.ilincs.org/api/PublicDatasets/findDatasets?term=",searchTerm,"&portal=",portal,"&dataType=",dataType)
req <- GET(apiUrl)
json <- httr::content(req, as = "text")
breastProteomics <- fromJSON(json)
breastProteomics[,c("experiment","assay","nsamples","description")]
```

## Getting metadata for the TCGA breast cancer proteomics RPPA dataset (TCGA\_BRCA\_RPPA\_2019)

```
experiment <- "TCGA_BRCA_RPPA_2019"

apiUrl <- paste("http://www.ilincs.org/api/ilincsR/getSamples?id=",experiment,sep="")
req <- GET(apiUrl)
json <- httr::content(req, as = "text")
sampleMeta <- fromJSON(json)
sampleMetaData <- data.frame(sampleMeta$data$rows)
head(sampleMetaData)
```

## Summary of the PAM50\_mRNA factor

```
table(sampleMetaData$PAM50_mRNA)
```

```
## 
##          <NA>    Basal-like HER2-enriched     Luminal A     Luminal B   Normal-like 
##           521            85            50           174           102             5
```

## Creating signature by comparing “Luminal A” and “HER2-enriched”

```
filter<-"PAM50_mRNA:Luminal A,,,PAM50_mRNA:HER2-enriched"
property <- "PAM50_mRNA"
treatment <- "Luminal A"
baseline <- "HER2-enriched"

apiUrl <- "http://www.ilincs.org/api/ilincsR/LincsDataAnalysis"
req <- POST(apiUrl, body = list(exp =experiment,prop = property,treatment=treatment,baseline=baseline, filterchk=filter,includeORexclude=1), encode = "json")

createdProteomicSignaturesSessionID <- httr::content(req)$sessionID
proteinSignatureFileUrl=paste0("http://www.ilincs.org/tmp/completeSig_",createdProteomicSignaturesSessionID,".xls")

diffProteinExpSignature<-read.table(proteinSignatureFileUrl,header=T,sep="\t",stringsAsFactors = F)[,c("PROBE","ID_geneid","Name_GeneSymbol","Value_LogDiffExp","Significance_pvalue")]
head(diffProteinExpSignature)
```

## Retrieving Top 12 Differentially expressed proteins (p-value<1e-10) (results in Fig 3A)

```
top12Proteins<-diffProteinExpSignature[diffProteinExpSignature$Significance_pvalue<1e-10,]
top12Proteins
```

## Heatmap of protein to top 12 proteins (Fig 3A)

```
f1 = colorRamp2(c(0,1), c("green", "red"))
f2 = colorRamp2(c(-1, 0,1), c("blue", "black", "yellow"), space = "RGB")

load(url(paste("http://www.ilincs.org/tmp/",experiment,".RData",sep="")),verbose=T)
```

```
## Loading objects:
##   eset
```

```
proteinExpressionEset<-get("eset")
proteinExpressionEset<-proteinExpressionEset[fData(proteinExpressionEset)$PROBE %in% top12Proteins$PROBE,pData(proteinExpressionEset)$PAM50_mRNA %in% c("Luminal A","HER2-enriched")]
proteinExpressionEset<-proteinExpressionEset[,order(pData(proteinExpressionEset)$PAM50_mRNA)]

meanPE<-apply(exprs(proteinExpressionEset),1,mean)
proteinExpressionDataTable<-sweep(exprs(proteinExpressionEset),1,meanPE,"-")


pam50ColumnAnnotation = HeatmapAnnotation(PAM50_mRNA=pData(proteinExpressionEset)$PAM50_mRNA,col = list(PAM50_mRNA = c("Luminal A"="red","HER2-enriched"="green")),annotation_height=unit(20,"mm"), annotation_name_gp = gpar(fontsize = 8), show_annotation_name =T, annotation_name_side="left")

  Heatmap(proteinExpressionDataTable, col = f2, cluster_columns = F, cluster_rows=T, show_column_names = F,top_annotation = pam50ColumnAnnotation, show_row_names = T,row_names_gp = gpar(fontsize = 12), heatmap_legend_param = list(title = "Log2 \nDifferential \nExpression", color_bar = "discrete"),rect_gp = gpar(col = "white", lty = 1, lwd = 0.1),show_heatmap_legend = T)
```

# Analyzing TCGA breast cancer transcriptom RNA-seq dataset

## Searching for the dataset in the TCGA collection (ie portal)

```
portal<-"TCGA"
searchTerm<-"BRCA"
assay<-"RNA-seq"
apiUrl <- paste0("http://www.ilincs.org/api/PublicDatasets/findDatasets?term=",searchTerm,"&portal=",portal,"&assay=",assay)
req <- GET(apiUrl)
json <- httr::content(req, as = "text")
breastTranscriptomics <- fromJSON(json)
breastTranscriptomics[breastTranscriptomics$dataType=="Gene Expression",c("experiment","assay","nsamples","description")]
```

## Getting metadata for the newest TCGA breast cancer transcriptomics RNA-seq dataset (TCGA\_BRCA\_RNASeqV2\_2019)

```
experiment <- "TCGA_BRCA_RNASeqV2_2019"

apiUrl <- paste("http://www.ilincs.org/api/ilincsR/getSamples?id=",experiment,sep="")
req <- GET(apiUrl)
json <- httr::content(req, as = "text")
sampleMeta <- fromJSON(json)
sampleMetaData <- data.frame(sampleMeta$data$rows)
head(sampleMetaData)
```

## Summary of the PAM50\_mRNA factor

```
table(sampleMetaData$PAM50_mRNA)
```

```
## 
##          <NA>    Basal-like HER2-enriched     Luminal A     Luminal B   Normal-like 
##           694            97            58           231           127             8
```

## Creating signature by comparing “Luminal A” and “HER2-enriched”

```
filter<-"PAM50_mRNA:Luminal A,,,PAM50_mRNA:HER2-enriched"
property <- "PAM50_mRNA"
treatment <- "Luminal A"
baseline <- "HER2-enriched"

apiUrl <- "http://www.ilincs.org/api/ilincsR/LincsDataAnalysis"
req <- POST(apiUrl, body = list(exp =experiment,prop = property,treatment=treatment,baseline=baseline, filterchk=filter,includeORexclude=1), encode = "json")

createdSignaturesSessionID <- httr::content(req)$sessionID
signatureFileUrl=paste0("http://www.ilincs.org/tmp/completeSig_",createdSignaturesSessionID,".xls")

diffGeneExpSignature<-read.table(signatureFileUrl,header=T,sep="\t",stringsAsFactors = F)[,c("ID_geneid","Name_GeneSymbol","Value_LogDiffExp","Significance_pvalue")]
head(diffGeneExpSignature)
```

## Retrieving results for top 12 Differentially expressed proteins (results in Fig 3B)

```
diffGeneExpTop12Proteins<-diffGeneExpSignature[which(diffGeneExpSignature$Name_GeneSymbol %in% top12Proteins$Name_GeneSymbol),]
diffGeneExpTop12Proteins
```

## Heatmap of gene expression data for top 12 proteins (Fig 3B)

```
f1 = colorRamp2(c(0,1), c("green", "red"))
f2 = colorRamp2(c(-1, 0,1), c("blue", "black", "yellow"), space = "RGB")

load(url(paste("http://www.ilincs.org/tmp/",experiment,".RData",sep="")),verbose=T)
```

```
## Loading objects:
##   eset
```

```
geneExpressionEset<-get("eset")
geneExpressionEset<-geneExpressionEset[fData(geneExpressionEset)$ID_geneid %in% diffGeneExpTop12Proteins$ID_geneid,pData(geneExpressionEset)$PAM50_mRNA %in% c("Luminal A","HER2-enriched")]
geneExpressionEset<-geneExpressionEset[,order(pData(geneExpressionEset)$PAM50_mRNA)]

meanPE<-apply(exprs(geneExpressionEset),1,mean)
geneExpressionDataTable<-sweep(exprs(geneExpressionEset),1,meanPE,"-")


pam50ColumnAnnotation = HeatmapAnnotation(PAM50_mRNA=pData(geneExpressionEset)$PAM50_mRNA,col = list(PAM50_mRNA = c("Luminal A"="red","HER2-enriched"="green")),annotation_height=unit(20,"mm"), annotation_name_gp = gpar(fontsize = 8), show_annotation_name =T, annotation_name_side="left")

  Heatmap(geneExpressionDataTable, col = f2, cluster_columns = F, cluster_rows=T, show_column_names = F,top_annotation = pam50ColumnAnnotation, show_row_names = T,row_labels=fData(geneExpressionEset)$Name_GeneSymbol,row_names_gp = gpar(fontsize = 12), heatmap_legend_param = list(title = "Log2 \nDifferential \nExpression", color_bar = "discrete"),rect_gp = gpar(col = "white", lty = 1, lwd = 0.1),show_heatmap_legend = T)
```

# Connectivity analysis of the transcriptional “Luminal A” vs “HER2-enriched” signature

## Upload the signature file that was previousl downloaded

```
sigFilename<-paste0("sigFileForUpload_",createdSignaturesSessionID,".tsv")
write.table(diffGeneExpSignature,file=sigFilename,col.names = T,row.names = F,sep="\t",quote = F)
apiUrl<-"http://www.ilincs.org/api/SignatureMeta/upload"
req <- POST(apiUrl, body=list(file=upload_file(sigFilename)))
uploadedFileName <- httr::content(req)$status$fileName[[1]]
uploadedFileName
```

```
## [1] "processedSig_Sat_May_7_22_14_25_2022_7940542.xls"
```

## Find connected CP signatures

```
apiUrl <- "http://www.ilincs.org/api/ilincsR/findConcordances"
req <- (POST(apiUrl, body = list(file=uploadedFileName, lib="LIB_5"), encode = "form"))
connectedCpSignatures <- data.table::rbindlist(httr::content(req)$concordanceTable, use.names = TRUE, fill = TRUE)
head(connectedCpSignatures)
```

## Group analysis of top 100 most connected signatures with signature of interest

```
signatureGroup <-connectedCpSignatures$signatureid[1:100]
apiUrl<-"http://www.ilincs.org/api/ilincsR/GroupLincsAnalysis"
req<-POST(apiUrl, body = list(idList = signatureGroup,noOfGenes = 50), encode = "json")                   
groupAnalysisSessionID <- httr::content(req)$data[[2]]
groupAnalysisSessionID
```

```
## [1] "Sat_May__22_14_50_7_7185586"
```

## Load r ExpressionSet from the signature group analysis

```
load(url(paste("http://www.ilincs.org/tmp/filteredeset_",groupAnalysisSessionID,".RData",sep="")),verbose=T)
```

```
## Loading objects:
##   filteredeset_Sat_May__22_14_50_7_7185586
```

```
groupAnalysisEset<-get(paste("filteredeset_",groupAnalysisSessionID,sep=""))
groupAnalysisEset
```

```
## ExpressionSet (storageMode: lockedEnvironment)
## assayData: 559 features, 100 samples 
##   element names: exprs 
## protocolData: none
## phenoData
##   sampleNames: LINCSCP_135677 LINCSCP_139907 ... LINCSCP_66347 (100 total)
##   varLabels: signatureID compound ... treatment (6 total)
##   varMetadata: labelDescription
## featureData
##   featureNames: 7153::TOP2A::DNA topoisomerase II alpha 9961::MVP::major vault protein ... 80349::WDR61::WD repeat domain 61 (559 total)
##   fvarLabels: ID_geneid Name_GeneSymbol DESCRIPTION
##   fvarMetadata: labelDescription
## experimentData: use 'experimentData(object)'
## Annotation:
```

## Creating heatmap of top 100 connected signatures (Fig 3C)

```
f2 = colorRamp2(c(-1, 0,1), c("blue", "black", "yellow"), space = "RGB")

  Heatmap(exprs(groupAnalysisEset), col = f2, cluster_columns = T, cluster_rows=T, column_names_gp = gpar(fontsize = 8),top_annotation = NULL, show_row_dend=F, show_column_dend=F,show_column_names = F, show_row_names = F, heatmap_legend_param = list(title = "MODZ", color_bar = "discrete"),rect_gp = gpar(col = "white", lty = 1, lwd = 0.1),show_heatmap_legend = T)
```
