# Supplementary material for: Connecting omics signatures and revealing biological mechanisms with iLINCS
Source: Nat Commun. 2022 Aug 9;13:4678. doi: 10.1038/s41467-022-32205-3 (PMC9362980; doi:10.1038/s41467-022-32205-3)
Supplement: Supplementary file 8 — Software 1 [file 41467_2022_32205_MOESM8_ESM.zip › ilincsAPI-master/useCases/useCase2.pdf]

iLINCS API R Notebook

Analyzing TCGA breast cancer proteomics RPPA dataset

Searching for the dataset in the TCGA collection (ie portal)

```
portal<-"TCGA"
searchTerm<-"BRCA"
dataType<-"Proteomics"
apiUrl <- paste0("http://www.ilincs.org/api/PublicDatasets/findDatasets?term=",searchTerm,"&portal=",po
req <- GET(apiUrl)
json <- httr::content(req, as = "text")
breastProteomics <- fromJSON(json)
breastProteomics[,c("experiment","assay","nsamples","description")]
```

```
##           experiment assay nsamples
## 1 TCGA_BRCA_RPPA_2019  RPPA      937
## 2   TCGA_OV_RPPA_2019  RPPA      443
##
## 1     937 RPPA breast invasive carcinoma (BRCA) samples from TCGA project. Data were downloaded via
## 2 443 RPPA ovarian serous cystadenocarcinoma (OV) samples from TCGA project. Data were downloaded vi
```

Getting metadata for the TCGA breast cancer proteomics RPPA dataset (TCGA_BRCA_RPPA_2019)

```
experiment <- "TCGA_BRCA_RPPA_2019"

apiUrl <- paste("http://www.ilincs.org/api/ilincsR/getSamples?id=",experiment,sep="")
req <- GET(apiUrl)
json <- httr::content(req, as = "text")
sampleMeta <- fromJSON(json)
sampleMetaData <- data.frame(sampleMeta$data$rows)
head(sampleMetaData)
```

```
##               MeasurementName age_quartile         sample_type primary_diagnosis morphology gender
## 1 TCGA-3C-AALI-01A-21-A43F-20        49-57 Primary solid Tumor             C50.9     8500/3 female b
## 2 TCGA-3C-AALK-01A-21-A43F-20        49-57 Primary solid Tumor             C50.9     8500/3 female b
## 3 TCGA-4H-AAAK-01A-21-A43F-20        49-57 Primary solid Tumor             C50.9     8520/3 female
## 4 TCGA-5L-AAT1-01A-21-A43F-20        58-66 Primary solid Tumor             C50.9     8520/3 female
## 5 TCGA-5T-A9QA-01A-21-A43F-20        49-57 Primary solid Tumor             C50.9     8523/3 female b
## 6 TCGA-A1-A0SF-01A-21-A17I-20        49-57 Primary solid Tumor             C50.9     8500/3 female
##   RPPA_Clusters neoplasm_cancer_status             histological_type
## 1          <NA>             TUMOR FREE  Infiltrating Ductal Carcinoma            Post (prior bilater
## 2          <NA>             TUMOR FREE  Infiltrating Ductal Carcinoma
## 3          <NA>             TUMOR FREE Infiltrating Lobular Carcinoma            Post (prior bilater
## 4          <NA>             WITH TUMOR Infiltrating Lobular Carcinoma            Post (prior bilater
## 5          <NA>                   <NA>                 Other, specify
## 6          <NA>             TUMOR FREE  Infiltrating Ductal Carcinoma Pre (<6 months since LMP AND n
```

```
##   PR_status PR_level_cell_percentage HER2_level HER2_status HER2_FISH margin_status distant_metastasi
## 1  Positive                     <10%       <NA>    Positive      <NA>      Negative              <N
## 2  Positive                   80-89%       <NA>    Positive      <NA>         Close              <N
## 3  Positive                   70-79%         2+   Equivocal      <NA>      Negative              <N
## 4  Positive                   10-19%         2+   Equivocal      <NA>      Negative              <N
## 5  Negative                     <NA>         2+   Equivocal  Negative          <NA>              <N
## 6  Positive                   90-99%       <NA>    Negative      <NA>      Negative                N
##   mut_impact_BRCA1 mut_deleterious_BRCA1 mut_impact_CASP8 mut_deleterious_CASP8 mut_impact_CBFB mut_
## 1               NO                    NO               NO                    NO              NO
## 2               NO                    NO               NO                    NO              NO
## 3               NO                    NO               NO                    NO              NO
## 4               NO                    NO         MODERATE                    NO              NO
## 5             <NA>                  <NA>             <NA>                  <NA>            <NA>
## 6               NO                    NO               NO                    NO              NO
##   mut_impact_CHD4 mut_deleterious_CHD4 mut_impact_CTCF mut_deleterious_CTCF mut_impact_ERBB2 mut_dele
## 1              NO                   NO        MODERATE                   NO               NO
## 2              NO                   NO              NO                   NO               NO
## 3              NO                   NO              NO                   NO         MODERATE
## 4        MODERATE                   NO              NO                   NO               NO
## 5            <NA>                 <NA>            <NA>                 <NA>             <NA>
## 6              NO                   NO              NO                   NO               NO
##   mut_impact_GATA3 mut_deleterious_GATA3 mut_impact_GPS2 mut_deleterious_GPS2 mut_impact_KMT2C mut_in
## 1               NO                    NO              NO                   NO         MODERATE
## 2               NO                    NO              NO                   NO               NO
## 3               NO                    NO              NO                   NO               NO
## 4         MODERATE                    NO        MODERATE                   NO               NO
## 5             <NA>                  <NA>            <NA>                 <NA>             <NA>
## 6               NO                    NO              NO                   NO         MODERATE
##   mut_deleterious_MAP3K1 mut_impact_NCOR1 mut_deleterious_NCOR1 mut_impact_NF1 mut_deleterious_NF1 mu
## 1                     NO               NO                    NO             NO                  NO
## 2                     NO         MODERATE                    NO             NO                  NO
## 3                     NO               NO                    NO             NO                  NO
## 4                     NO               NO                    NO           HIGH                  NO
## 5                   <NA>             <NA>                  <NA>           <NA>                <NA>
## 6                     NO               NO                    NO             NO                  NO
##   mut_deleterious_PTEN mut_impact_PTPRD mut_deleterious_PTPRD mut_impact_RB1 mut_deleterious_RB1 mut_
## 1                   NO             HIGH                    NO             NO                  NO
## 2                   NO               NO                    NO             NO                  NO
## 3                   NO               NO                    NO             NO                  NO
## 4                   NO               NO                    NO             NO                  NO
## 5                 <NA>             <NA>                  <NA>           <NA>                <NA>
## 6                   NO              LOW                    NO             NO                  NO
##   mut_deleterious_TBX3 mut_impact_TP53 mut_deleterious_TP53
## 1                   NO            HIGH                   NO
## 2                   NO              NO                   NO
## 3                   NO              NO                   NO
## 4                   NO              NO                   NO
## 5                 <NA>            <NA>                 <NA>
## 6                   NO              NO                   NO
```

Summary of the PAM50_mRNA factor

```r
table(sampleMetaData$PAM50_mRNA)
```

```
##
##          <NA>    Basal-like HER2-enriched     Luminal A     Luminal B   Normal-like
##           521            85            50           174           102             5
```

Creating signature by comparing "Luminal A" and "HER2-enriched"

```
filter<-"PAM50_mRNA:Luminal A,,,PAM50_mRNA:HER2-enriched"
property <- "PAM50_mRNA"
treatment <- "Luminal A"
baseline <- "HER2-enriched"

apiUrl <- "http://www.ilincs.org/api/ilincsR/LincsDataAnalysis"
req <- POST(apiUrl, body = list(exp =experiment,prop = property,treatment=treatment,baseline=baseline, 

createdProteomicSignaturesSessionID <- httr::content(req)$sessionID
proteinSignatureFileUrl=paste0("http://www.ilincs.org/tmp/completeSig_",createdProteomicSignaturesSessi

diffProteinExpSignature<-read.table(proteinSignatureFileUrl,header=T,sep="\t",stringsAsFactors = F)[,c(
head(diffProteinExpSignature)
```

```
##         PROBE ID_geneid Name_GeneSymbol Value_LogDiffExp Significance_pvalue
## 1    ER-alpha      2099            ESR1         2.835721        1.320409e-25
## 2 HER2_pY1248      2064           ERBB2        -1.300406        3.561584e-23
## 3        HER2      2064           ERBB2        -1.598668        2.861979e-22
## 4       Bcl-2       596            BCL2         1.255441        2.495637e-20
## 5          PR      5241             PGR         2.887453        8.200612e-20
## 6 EGFR_pY1068      1956            EGFR        -1.152552        2.058972e-19
```

Retrieving Top 12 Differentially expressed proteins (p-value<1e-10) (results in Fig 3A)

```
top12Proteins<-diffProteinExpSignature[diffProteinExpSignature$Significance_pvalue<1e-10,]
top12Proteins
```

```
##          PROBE ID_geneid Name_GeneSymbol Value_LogDiffExp Significance_pvalue
## 1     ER-alpha      2099            ESR1        2.8357212        1.320409e-25
## 2  HER2_pY1248      2064           ERBB2       -1.3004059        3.561584e-23
## 3         HER2      2064           ERBB2       -1.5986681        2.861979e-22
## 4        Bcl-2       596            BCL2        1.2554406        2.495637e-20
## 5           PR      5241             PGR        2.8874526        8.200612e-20
## 6  EGFR_pY1068      1956            EGFR       -1.1525515        2.058972e-19
## 7         ASNS       440            ASNS       -0.6198126        1.002036e-16
## 8        FoxM1      2305           FOXM1       -0.5444826        8.617266e-16
## 9    Cyclin_B1       891           CCNB1       -0.9719865        5.824394e-15
## 10       GATA3      2625           GATA3        0.9602510        4.811066e-12
## 11        G6PD      2539            G6PD       -0.5373067        1.266588e-11
## 12 4E-BP1_pS65      1978         EIF4EBP1       -0.2793193        9.949816e-11
```

Heatmap of protein to top 12 proteins (Fig 3A)

```
f1 = colorRamp2(c(0,1), c("green", "red"))
f2 = colorRamp2(c(-1, 0,1), c("blue", "black", "yellow"), space = "RGB")
```

```r
load(url(paste("http://www.ilincs.org/tmp/",experiment,".RData",sep="")),verbose=T)
```

```
## Loading objects:
##   eset
```

```r
proteinExpressionEset<-get("eset")
proteinExpressionEset<-proteinExpressionEset[fData(proteinExpressionEset)$PROBE %in% top12Proteins$PROBE
proteinExpressionEset<-proteinExpressionEset[,order(pData(proteinExpressionEset)$PAM50_mRNA)]

meanPE<-apply(exprs(proteinExpressionEset),1,mean)
proteinExpressionDataTable<-sweep(exprs(proteinExpressionEset),1,meanPE,"-")


pam50ColumnAnnotation = HeatmapAnnotation(PAM50_mRNA=pData(proteinExpressionEset)$PAM50_mRNA,col = list

  Heatmap(proteinExpressionDataTable, col = f2, cluster_columns = F, cluster_rows=T, show_column_names =
```

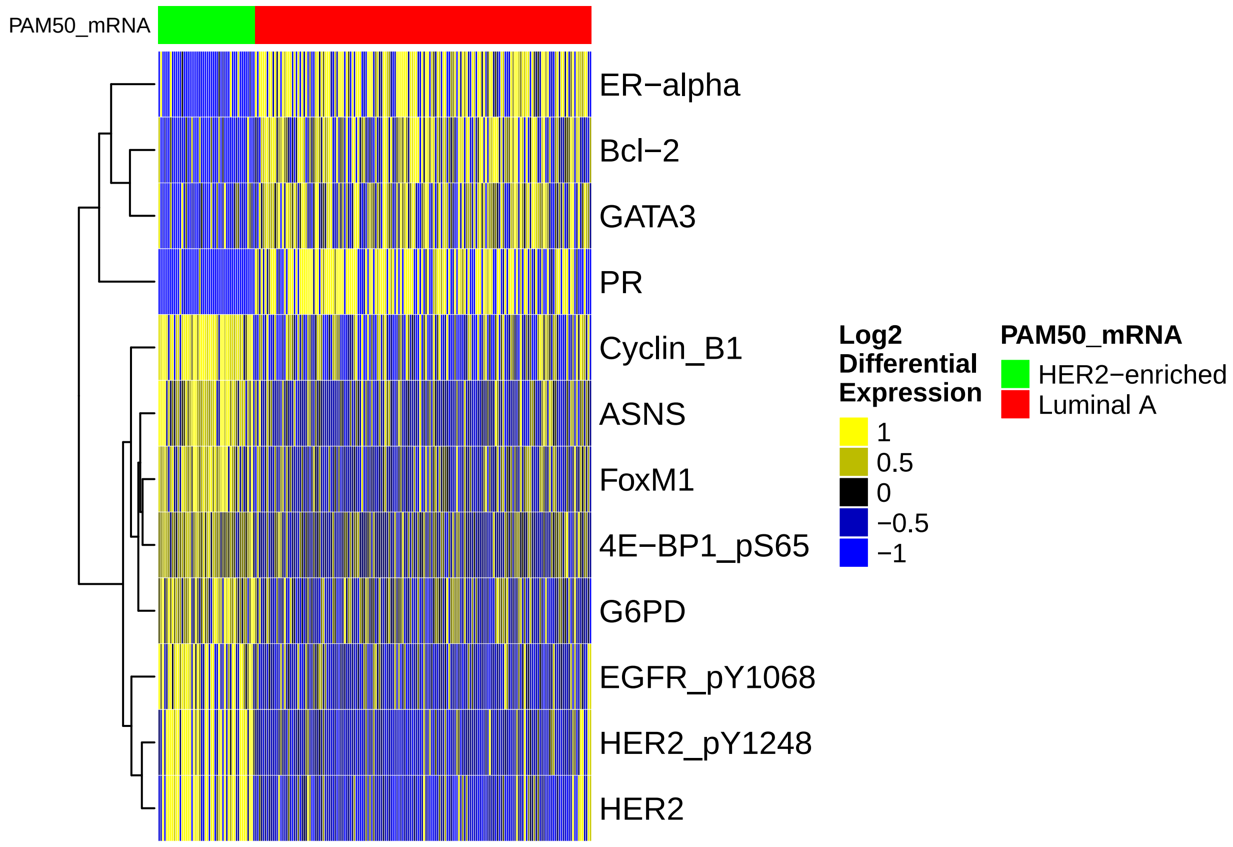

Analyzing TCGA breast cancer transcriptom RNA-seq dataset

Searching for the dataset in the TCGA collection (ie portal)

```r
portal<-"TCGA"
searchTerm<-"BRCA"
assay<-"RNA-seq"
apiUrl <- paste0("http://www.ilincs.org/api/PublicDatasets/findDatasets?term=",searchTerm,"&portal=",po
```

```r
req <- GET(apiUrl)
json <- httr::content(req, as = "text")
breastTranscriptomics <- fromJSON(json)
breastTranscriptomics[breastTranscriptomics$dataType=="Gene Expression",c("experiment","assay","nsamples
```

```
##                 experiment   assay nsamples
## 5      TCGA_BRCA_RNASeqV2 RNA-seq      919
## 6 TCGA_BRCA_RNASeqV2_2019 RNA-seq     1215
## 9   TCGA_OV_RNASeqV2_2019 RNA-seq      309
##
## 5                                                    919 RNA-seq breast invasive carcinoma (BRCA) sam
## 6    1215 RNA-seq breast invasive carcinoma (BRCA) samples from TCGA project. Data were downloaded vi
## 9 309 RNA-seq ovarian serous cystadenocarcinoma (OV) samples from TCGA project. Data were downloaded
```

Getting metadata for the newest TCGA breast cancer transcriptomics RNA-seq dataset (TCGA_BRCA_RNASeqV2_2019)

```r
experiment <- "TCGA_BRCA_RNASeqV2_2019"

apiUrl <- paste("http://www.ilincs.org/api/ilincsR/getSamples?id=",experiment,sep="")
req <- GET(apiUrl)
json <- httr::content(req, as = "text")
sampleMeta <- fromJSON(json)
sampleMetaData <- data.frame(sampleMeta$data$rows)
head(sampleMetaData)
```

```
##                 MeasurementName age_quartile         sample_type primary_diagnosis morphology gender
## 1 TCGA-3C-AAAU-01A-11R-A41B-07        49-57 Primary solid Tumor             C50.9     8520/3 female
## 2 TCGA-3C-AALI-01A-11R-A41B-07        49-57 Primary solid Tumor             C50.9     8500/3 female
## 3 TCGA-3C-AALJ-01A-31R-A41B-07        58-66 Primary solid Tumor             C50.9     8500/3 female
## 4 TCGA-3C-AALK-01A-11R-A41B-07        49-57 Primary solid Tumor             C50.9     8500/3 female
## 5 TCGA-4H-AAAK-01A-12R-A41B-07        49-57 Primary solid Tumor             C50.9     8520/3 female
## 6 TCGA-5L-AAT0-01A-12R-A41B-07        26-48 Primary solid Tumor             C50.9     8520/3 female
##   RPPA_Clusters neoplasm_cancer_status              histological_type
## 1          <NA>             WITH TUMOR Infiltrating Lobular Carcinoma Pre (<6 months since LMP AND no
## 2          <NA>             TUMOR FREE  Infiltrating Ductal Carcinoma            Post (prior bilatera
## 3          <NA>             TUMOR FREE  Infiltrating Ductal Carcinoma            Post (prior bilatera
## 4          <NA>             TUMOR FREE  Infiltrating Ductal Carcinoma
## 5          <NA>             TUMOR FREE Infiltrating Lobular Carcinoma            Post (prior bilatera
## 6          <NA>             TUMOR FREE Infiltrating Lobular Carcinoma            Post (prior bilatera
##   PR_status PR_level_cell_percentage HER2_level   HER2_status HER2_FISH margin_status distant_metasta
## 1  Positive                   50-59%       <NA>      Negative      <NA>      Negative
## 2  Positive                     <10%       <NA>      Positive      <NA>      Negative
## 3  Positive                   30-39%       <NA> Indeterminate      <NA>      Negative
## 4  Positive                   80-89%       <NA>      Positive      <NA>         Close
## 5  Positive                   70-79%         2+     Equivocal      <NA>      Negative
## 6  Positive                   50-59%         1+      Negative      <NA>      Positive
##   mut_impact_BRCA1 mut_deleterious_BRCA1 mut_impact_CASP8 mut_deleterious_CASP8 mut_impact_CBFB mut_d
## 1             <NA>                  <NA>             <NA>                  <NA>            <NA>
## 2               NO                    NO               NO                    NO              NO
## 3             <NA>                  <NA>             <NA>                  <NA>            <NA>
## 4               NO                    NO               NO                    NO              NO
## 5               NO                    NO               NO                    NO              NO
```

```
## 6              NO                     NO               NO                     NO              NO
##   mut_impact_CHD4 mut_deleterious_CHD4 mut_impact_CTCF mut_deleterious_CTCF mut_impact_ERBB2 mut_dele
## 1            <NA>                 <NA>            <NA>                 <NA>             <NA>
## 2              NO                   NO        MODERATE                   NO               NO
## 3            <NA>                 <NA>            <NA>                 <NA>             <NA>
## 4              NO                   NO              NO                   NO               NO
## 5              NO                   NO              NO                   NO         MODERATE
## 6              NO                   NO              NO                   NO               NO
##   mut_impact_GATA3 mut_deleterious_GATA3 mut_impact_GPS2 mut_deleterious_GPS2 mut_impact_KMT2C mut_in
## 1             <NA>                  <NA>            <NA>                 <NA>             <NA>
## 2               NO                    NO              NO                   NO         MODERATE
## 3             <NA>                  <NA>            <NA>                 <NA>             <NA>
## 4               NO                    NO              NO                   NO               NO
## 5               NO                    NO              NO                   NO               NO
## 6               NO                    NO              NO                   NO               NO
##   mut_deleterious_MAP3K1 mut_impact_NCOR1 mut_deleterious_NCOR1 mut_impact_NF1 mut_deleterious_NF1 mu
## 1                   <NA>             <NA>                  <NA>           <NA>                <NA>
## 2                     NO               NO                    NO             NO                  NO
## 3                   <NA>             <NA>                  <NA>           <NA>                <NA>
## 4                     NO         MODERATE                    NO             NO                  NO
## 5                     NO               NO                    NO             NO                  NO
## 6                     NO               NO                    NO             NO                  NO
##   mut_deleterious_PTEN mut_impact_PTPRD mut_deleterious_PTPRD mut_impact_RB1 mut_deleterious_RB1 mut_
## 1                 <NA>             <NA>                  <NA>           <NA>                <NA>
## 2                   NO             HIGH                    NO             NO                  NO
## 3                 <NA>             <NA>                  <NA>           <NA>                <NA>
## 4                   NO               NO                    NO             NO                  NO
## 5                   NO               NO                    NO             NO                  NO
## 6                   NO               NO                    NO             NO                  NO
##   mut_deleterious_TBX3 mut_impact_TP53 mut_deleterious_TP53
## 1                 <NA>            <NA>                 <NA>
## 2                   NO            HIGH                   NO
## 3                 <NA>            <NA>                 <NA>
## 4                   NO              NO                   NO
## 5                   NO              NO                   NO
## 6                   NO              NO                   NO
```

Summary of the PAM50_mRNA factor

```r
table(sampleMetaData$PAM50_mRNA)
```

```
##
##          <NA>    Basal-like HER2-enriched     Luminal A     Luminal B   Normal-like
##           694            97            58           231           127             8
```

Creating signature by comparing “Luminal A” and “HER2-enriched”

```r
filter<-"PAM50_mRNA:Luminal A,,,PAM50_mRNA:HER2-enriched"
property <- "PAM50_mRNA"
treatment <- "Luminal A"
baseline <- "HER2-enriched"

apiUrl <- "http://www.ilincs.org/api/ilincsR/LincsDataAnalysis"
```

```r
req <- POST(apiUrl, body = list(exp =experiment,prop = property,treatment=treatment,baseline=baseline, i

createdSignaturesSessionID <- httr::content(req)$sessionID
signatureFileUrl=paste0("http://www.ilincs.org/tmp/completeSig_",createdSignaturesSessionID,".xls")

diffGeneExpSignature<-read.table(signatureFileUrl,header=T,sep="\t",stringsAsFactors = F)[,c("ID_geneid
head(diffGeneExpSignature)
```

```
##   ID_geneid Name_GeneSymbol Value_LogDiffExp Significance_pvalue
## 1      2099            ESR1         3.966901        1.246425e-43
## 2      2886            GRB7        -2.680763        7.551647e-43
## 3     10948          STARD3        -2.124142        8.181878e-43
## 4       596            BCL2         2.378674        2.223119e-38
## 5     80129         CCDC170         2.612447        1.178032e-37
## 6      2064           ERBB2        -2.487254        1.457141e-37
```

Retrieving results for top 12 Differentially expressed proteins (results in Fig 3B)

```r
diffGeneExpTop12Proteins<-diffGeneExpSignature[which(diffGeneExpSignature$Name_GeneSymbol %in% top12Prot
diffGeneExpTop12Proteins
```

```
##       ID_geneid Name_GeneSymbol Value_LogDiffExp Significance_pvalue
## 1          2099            ESR1        3.9669005        1.246425e-43
## 4           596            BCL2        2.3786737        2.223119e-38
## 6          2064           ERBB2       -2.4872541        1.457141e-37
## 12         5241             PGR        4.7020572        2.802427e-34
## 101        2539            G6PD       -1.1821240        2.614818e-24
## 105        2305           FOXM1       -1.5630961        2.822134e-24
## 146        2625           GATA3        1.4899675        2.436816e-22
## 194         891           CCNB1       -1.1241082        3.479925e-21
## 1812        440            ASNS       -0.6974389        2.196442e-09
## 3956       1978        EIF4EBP1       -0.6832938        8.336803e-06
## 11192      1956            EGFR       -0.4563731        6.009345e-02
```

Heatmap of gene expression data for top 12 proteins (Fig 3B)

```r
f1 = colorRamp2(c(0,1), c("green", "red"))
f2 = colorRamp2(c(-1, 0,1), c("blue", "black", "yellow"), space = "RGB")

load(url(paste("http://www.ilincs.org/tmp/",experiment,".RData",sep="")),verbose=T)
```

```
## Loading objects:
##   eset
```

```r
geneExpressionEset<-get("eset")
geneExpressionEset<-geneExpressionEset[fData(geneExpressionEset)$ID_geneid %in% diffGeneExpTop12Protein
geneExpressionEset<-geneExpressionEset[,order(pData(geneExpressionEset)$PAM50_mRNA)]

meanPE<-apply(exprs(geneExpressionEset),1,mean)
geneExpressionDataTable<-sweep(exprs(geneExpressionEset),1,meanPE,"-")


pam50ColumnAnnotation = HeatmapAnnotation(PAM50_mRNA=pData(geneExpressionEset)$PAM50_mRNA,col = list(PA
```

```
Heatmap(geneExpressionDataTable, col = f2, cluster_columns = F, cluster_rows=T, show_column_names = F
```

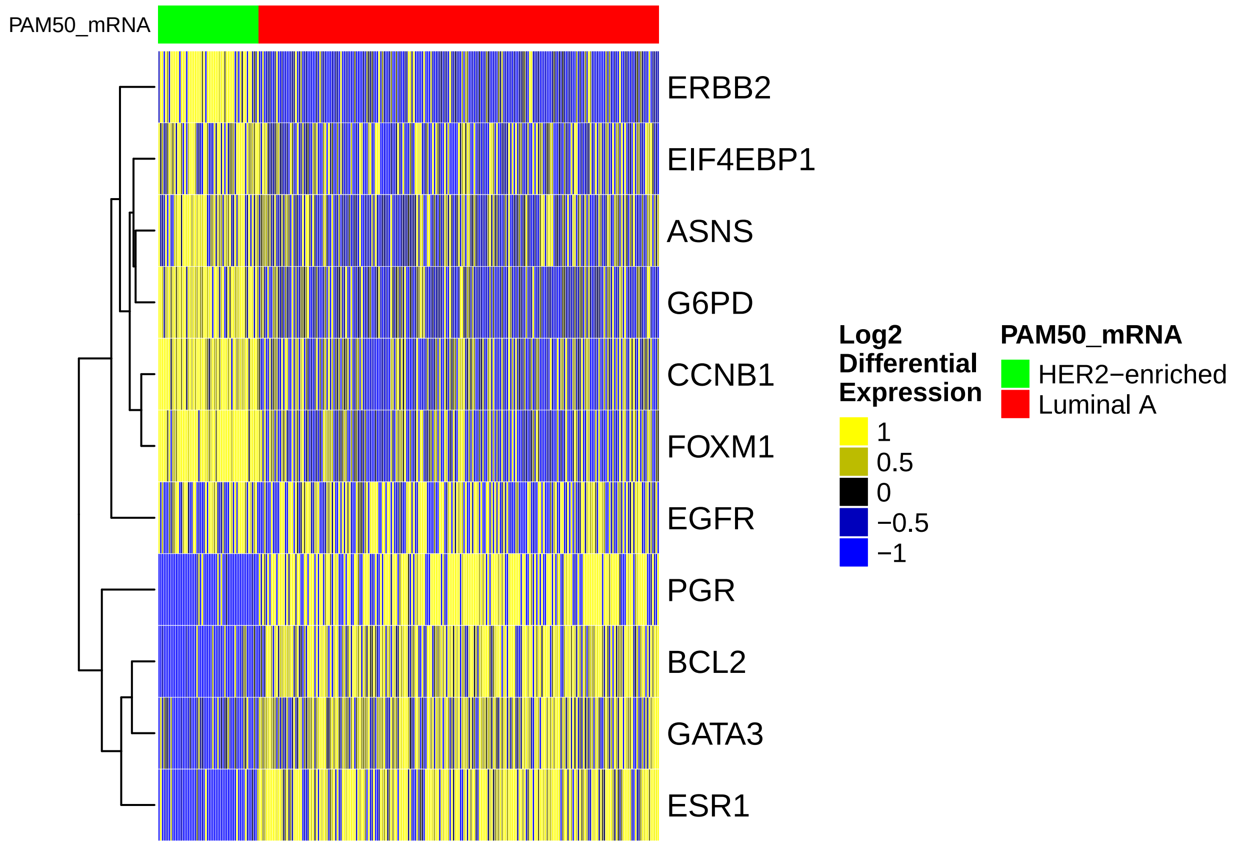

Connectivity analysis of the transcriptional "Luminal A" vs "HER2-enriched" signature

Upload the signature file that was previousl downloaded

```
sigFilename<-paste0("sigFileForUpload_",createdSignaturesSessionID,".tsv")
write.table(diffGeneExpSignature,file=sigFilename,col.names = T,row.names = F,sep="\t",quote = F)
apiUrl<-"http://www.ilincs.org/api/SignatureMeta/upload"
req <- POST(apiUrl, body=list(file=upload_file(sigFilename)))
uploadedFileName <- httr::content(req)$status$fileName[[1]]
uploadedFileName
```

```
## [1] "processedSig_Sat_May_7_22_14_25_2022_7940542.xls"
```

Find connected CP signatures

```
apiUrl <- "http://www.ilincs.org/api/ilincsR/findConcordances"
req <- (POST(apiUrl, body = list(file=uploadedFileName, lib="LIB_5"), encode = "form"))
connectedCpSignatures <- data.table::rbindlist(httr::content(req)$concordanceTable, use.names = TRUE, f
head(connectedCpSignatures)
```

```
##    similarity       pValue nGenes    compound lincsPertID     GeneTargets concentration time
```

```
## 1:  0.5366747 1.123590e-73    973      KPT-330   LSM-45842            XPO1        0.37uM  24h LINCSCP_
## 2:  0.5323634 2.597747e-72    973 Palbociclib    LSM-1071 CCND1|CDK4|CDK6        10uM  24h LINCSCP_
## 3:  0.5223302 3.263468e-69    973  BMS-536924    LSM-1210           IGF1R        10uM  24h  LINCSCP
## 4:  0.5179384 6.885138e-68    973 Palbociclib    LSM-1071 CCND1|CDK4|CDK6       3.33uM  24h LINCSCP_
## 5:  0.5160682 2.488524e-67    973      WZ 3146    LSM-5809            EGFR        10uM  24h  LINCSCP
## 6:  0.5143386 8.108895e-67    973 Palbociclib    LSM-1071 CCND1|CDK4|CDK6       1.11uM  24h LINCSCP_
```

Group analysis of top 100 most connected signatures with signature of interest

```
signatureGroup <-connectedCpSignatures$signatureid[1:100]
apiUrl<-"http://www.ilincs.org/api/ilincsR/GroupLincsAnalysis"
req<-POST(apiUrl, body = list(idList = signatureGroup,noOfGenes = 50), encode = "json")
groupAnalysisSessionID <- httr::content(req)$data[[2]]
groupAnalysisSessionID
```

```
## [1] "Sat_May__22_14_50_7_7185586"
```

Load r ExpressionSet from the signature group analysis

```
load(url(paste("http://www.ilincs.org/tmp/filteredeset_",groupAnalysisSessionID,".RData",sep="")),verbo
```

```
## Loading objects:
##   filteredeset_Sat_May__22_14_50_7_7185586
```

```
groupAnalysisEset<-get(paste("filteredeset_",groupAnalysisSessionID,sep=""))
groupAnalysisEset
```

```
## ExpressionSet (storageMode: lockedEnvironment)
## assayData: 559 features, 100 samples
##   element names: exprs
## protocolData: none
## phenoData
##   sampleNames: LINCSCP_135677 LINCSCP_139907 ... LINCSCP_66347 (100 total)
##   varLabels: signatureID compound ... treatment (6 total)
##   varMetadata: labelDescription
## featureData
##   featureNames: 7153::TOP2A::DNA topoisomerase II alpha 9961::MVP::major vault protein ... 80349::WDL
##   fvarLabels: ID_geneid Name_GeneSymbol DESCRIPTION
##   fvarMetadata: labelDescription
## experimentData: use 'experimentData(object)'
## Annotation:
```

Creating heatmap of top 100 connected signatures (Fig 3C)

```
f2 = colorRamp2(c(-1, 0,1), c("blue", "black", "yellow"), space = "RGB")

  Heatmap(exprs(groupAnalysisEset), col = f2, cluster_columns = T, cluster_rows=T, column_names_gp = gpa
```

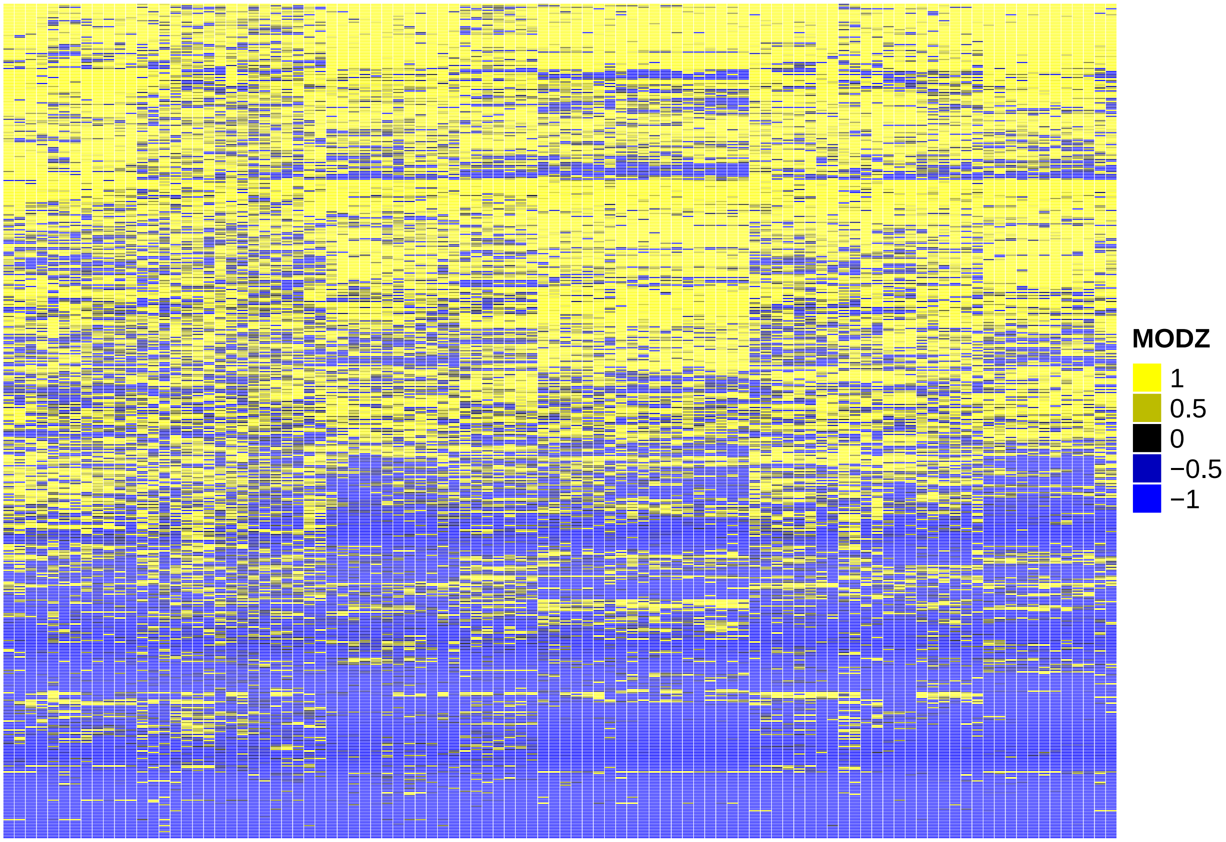
MODZ
1
0.5
0
−0.5
−1
