# Supplementary material for: Connecting omics signatures and revealing biological mechanisms with iLINCS
Source: Nat Commun. 2022 Aug 9;13:4678. doi: 10.1038/s41467-022-32205-3 (PMC9362980; doi:10.1038/s41467-022-32205-3)
Supplement: Supplementary file 8 — Software 1 [file 41467_2022_32205_MOESM8_ESM.zip › ilincsAPI-master/usingIlincsApis.html]

iLINCS API R Notebook


# iLINCS API R Notebook

## Display Signature Libraries

```
apiUrl <- "http://www.ilincs.org/api/SignatureLibraries"
req <- GET(apiUrl)
json <- httr::content(req, as = "text")
ilincs_libraries <- fromJSON(json)
ilincs_libraries[,c("libraryID","libraryName")]
```

## Searching for signature using BROAD ID

##### Searching for signature for given term. In this example **“Diclofenac”** coumpound will be shown as an example.

```
term <- "Diclofenac"
ilincs_libId<-"LIB_5"
apiUrl <- paste("http://www.ilincs.org/api/SignatureMeta/findTermWithSynonyms?term=",term,"&library=",ilincs_libId,sep="")
req <- GET(apiUrl)

ilincs_result_df<-fromJSON(httr::content(req,type="text"))$data
```

```
## No encoding supplied: defaulting to UTF-8.
```

```
head(ilincs_result_df[,c("cellline","compound","concentration","signatureid","is_exemplar")])
```

### Selecting a signature to analyze

```
ilincs_signatureId <- ilincs_result_df[1,"signatureid"]
ilincs_signatureId
```

```
## [1] "LINCSCP_100"
```

## Getting signature data and vulcano plot

##### The first step is to retrieve the session id for creation of the signature data table

```
req <- POST("http://www.ilincs.org/api/ilincsR/downloadSignature", body = list(sigID = paste(ilincs_signatureId), display = FALSE), encode = "json")
ilincs_sessionId<-unlist(httr::content(req))
ilincs_sessionId
```

```
##                                   data 
## "sig_Thu_Apr_14_00_18_03_2022_4145405"
```

### Retrieving data

```
fileUrl=paste("http://www.ilincs.org/tmp/",ilincs_sessionId,".xls",sep="")
signatureData<-read.table(fileUrl,sep="\t",header=T,stringsAsFactors = F)
head(signatureData)
```

### Insert volcano plot

```
apiUrl <- paste("http://www.ilincs.org/api/ilincsR/volcanoPlot?file=",ilincs_sessionId,".xls",sep="")
req <- GET(apiUrl)
ilincs_volcanoUrl<-paste("http://www.ilincs.org",httr::content(req)$url,sep="")
include_graphics(ilincs_volcanoUrl)
```

## Get connected signatures

```
apiUrl <- paste("http://www.ilincs.org/api/SignatureMeta/findConcordantSignatures?sigID=",ilincs_signatureId,"&lib=",ilincs_libId,sep="")
req <- GET(apiUrl)
# prettify(httr::content(req,type="text"))
ilincs_conn_df<-fromJSON(httr::content(req,type="text"))
```

```
## No encoding supplied: defaulting to UTF-8.
```

```
head(ilincs_conn_df)
```

## Find LINCS signatures connected to user a submitted signature

##### Creating user submitted signature in the form of a gene list

```
# top100signature <- signatureData[order(signatureData$Significance_pvalue)[1:100],c("Name_GeneSymbol","Value_LogDiffExp","Significance_pvalue")]
top100signature <- signatureData[order(signatureData$Significance_pvalue)[1:100],]
head(top100signature)
```

### Uploading signature as a list genes for enrichment

```
apiUrl="http://www.ilincs.org/api/ilincsR/findConcordancesSC"
req <- POST(apiUrl, body = list(mode="geneList",metadata=TRUE,signatureProfile = list(genes=top100signature$Name_GeneSymbol)),encode = "json")
ilincsEnrichedSignatures <- data.table::rbindlist(httr::content(req)$sigScores, use.names = TRUE, fill = TRUE)
head(ilincsEnrichedSignatures)
```

## Get GSEA plot

```
apiUrl <- "http://www.ilincs.org/api/ilincsR/plotGSEA"
req <- POST(apiUrl, body = list(signatureId = ilincs_signatureId,genes = top100signature$ID_geneid, output="png"), encode = "json")

gseaPlotPng <- httr::content(req)$fileName
gseaPlotUrl <- paste("http://www.ilincs.org/tmp/",gseaPlotPng,".png",sep="")
include_graphics(gseaPlotUrl)
```

## Signature as a list of up and down genes

```
apiUrl="http://www.ilincs.org/api/ilincsR/findConcordancesSC"

topUpRegulatedGenes <- list(genesUp=top100signature$Name_GeneSymbol[top100signature$Value_LogDiffExp > 0])
topDownregulatedGenes <- list(genesDown=top100signature$Name_GeneSymbol[top100signature$Value_LogDiffExp < 0])

req <- POST("http://www.ilincs.org/api/ilincsR/findConcordancesSC", body = list(mode="UpDn",metadata=TRUE,signatureProfile = c(topUpRegulatedGenes, topDownregulatedGenes)),encode = "json")

ilincsUpDnConnectedSignatures <- data.table::rbindlist(httr::content(req)$concordanceTable, use.names = TRUE, fill = TRUE)
head(ilincsUpDnConnectedSignatures)
```

## Group analysis of top 20 most connected signatures with signature of interest

```
signatureGroup <-ilincsUpDnConnectedSignatures$signatureID[1:20]
apiUrl<-"http://www.ilincs.org/api/ilincsR/GroupLincsAnalysis"
req<-POST(apiUrl, body = list(idList = signatureGroup,noOfGenes = 50), encode = "json")                   
groupAnalysisSessionID <- httr::content(req)$data[[2]]
groupAnalysisSessionID
```

```
## [1] "Thu_Apr_14_2022_00_24_47_5867221"
```

### Signature group heatmap on iLINCS

```
heatmap_url<-paste("http://www.ilincs.org/apps/heatmap/?sessionID=",groupAnalysisSessionID,"&property=signatureID",sep = "")
# browseURL(heatmap_url)  ##uncoment to launch the browser
# include_url(heatmap_url, height = "900px")  ##uncomment to embed in iframe
```

### Signature group PCA and tSNE app on iLINCS

```
pca_url<-paste("http://www.ilincs.org/apps/pca/?sessionID=",groupAnalysisSessionID,"&property=signatureID",sep = "")
# browseURL(pca_url)  ##uncoment to launch the browser
# include_url(pca_url, height = "900px")  ##uncomment to embed in iframe
```

### Load r ExpressionSet from the signature analysis

```
load(url(paste("http://www.ilincs.org/tmp/filteredeset_",groupAnalysisSessionID,".RData",sep="")),verbose=T)
```

```
## Loading objects:
##   filteredeset_Thu_Apr_14_2022_00_24_47_5867221
```

```
eset<-get(paste("filteredeset_",groupAnalysisSessionID,sep=""))
eset
```

```
## Loading required package: Biobase
```

```
## Loading required package: BiocGenerics
```

```
## 
## Attaching package: 'BiocGenerics'
```

```
## The following objects are masked from 'package:stats':
## 
##     IQR, mad, sd, var, xtabs
```

```
## The following objects are masked from 'package:base':
## 
##     anyDuplicated, append, as.data.frame, basename, cbind, colnames, dirname, do.call, duplicated, eval, evalq, Filter, Find, get, grep, grepl, intersect, is.unsorted, lapply,
##     Map, mapply, match, mget, order, paste, pmax, pmax.int, pmin, pmin.int, Position, rank, rbind, Reduce, rownames, sapply, setdiff, sort, table, tapply, union, unique, unsplit,
##     which.max, which.min
```

```
## Welcome to Bioconductor
## 
##     Vignettes contain introductory material; view with 'browseVignettes()'. To cite Bioconductor, see 'citation("Biobase")', and for packages 'citation("pkgname")'.
```

```
## 
## Attaching package: 'Biobase'
```

```
## The following object is masked from 'package:httr':
## 
##     content
```

```
## ExpressionSet (storageMode: lockedEnvironment)
## assayData: 453 features, 20 samples 
##   element names: exprs 
## protocolData: none
## phenoData
##   sampleNames: LINCSCP_100 LINCSCP_99 ... LINCSCP_176754 (20 total)
##   varLabels: signatureID compound ... treatment (6 total)
##   varMetadata: labelDescription
## featureData
##   featureNames: 5993::RFX5::regulatory factor X5 9134::CCNE2::cyclin E2 ... 1277::COL1A1::collagen type I alpha 1 chain (453 total)
##   fvarLabels: ID_geneid Name_GeneSymbol DESCRIPTION
##   fvarMetadata: labelDescription
## experimentData: use 'experimentData(object)'
## Annotation:
```

### Download gct from the signature analysis

```
download.file(paste("http://www.ilincs.org/tmp/filteredeset_",groupAnalysisSessionID,".gct",sep=""),destfile="sigs.gct")
sigs <- readLines(con <- file("sigs.gct"))
head(sigs,n=10)
```

```
##  [1] "#1.3"                                                                                                                                                                                                                                                                                                                                        
##  [2] "453\t20\t3\t6"                                                                                                                                                                                                                                                                                                                               
##  [3] "id\tID_geneid\tName_GeneSymbol\tDESCRIPTION\tLINCSCP_100\tLINCSCP_99\tLINCSCP_160\tLINCSCP_176580\tLINCSCP_176698\tLINCSCP_1829\tLINCSCP_65\tLINCSCP_176748\tLINCSCP_178725\tLINCSCP_176625\tLINCSCP_176560\tLINCSCP_124\tLINCSCP_138\tLINCSCP_176578\tLINCSCP_109\tLINCSCP_2105\tLINCSCP_176623\tLINCSCP_2321\tLINCSCP_2160\tLINCSCP_176754"
##  [4] "signatureID\tna\tna\tna\tLINCSCP_100\tLINCSCP_99\tLINCSCP_160\tLINCSCP_176580\tLINCSCP_176698\tLINCSCP_1829\tLINCSCP_65\tLINCSCP_176748\tLINCSCP_178725\tLINCSCP_176625\tLINCSCP_176560\tLINCSCP_124\tLINCSCP_138\tLINCSCP_176578\tLINCSCP_109\tLINCSCP_2105\tLINCSCP_176623\tLINCSCP_2321\tLINCSCP_2160\tLINCSCP_176754"                    
##  [5] "compound\tna\tna\tna\tDiclofenac\tYM90709\tTubocurarine\tAmidate\tTOCRIS-0930\tTozasertib\tChlorthalidone\tTrazodone\tClotrimazole\tPiperidolate\tOxfendazole\tErythromycin Estolate\tCilostazol\tVenlafaxine\tRS-39604\tPK04_181029\tDexamethasone Acetate\tChenodiol\tAC1M5YQH\tStaurosporine Aglycon"                                     
##  [6] "concentration\tna\tna\tna\t10uM\t10uM\t10uM\t10uM\t10uM\t10uM\t10uM\t10uM\t10uM\t10uM\t10uM\t10uM\t10uM\t10uM\t10uM\t10uM\t10uM\t10uM\t10uM\t10uM"                                                                                                                                                                                           
##  [7] "cellLine\tna\tna\tna\tA375\tA375\tA375\tA375\tA375\tA375\tA375\tA375\tA375\tA375\tA375\tA375\tA375\tA375\tA375\tA375\tA375\tA375\tA375\tA375"                                                                                                                                                                                                
##  [8] "time\tna\tna\tna\t6h\t6h\t6h\t6h\t6h\t6h\t6h\t6h\t6h\t6h\t6h\t6h\t6h\t6h\t6h\t6h\t6h\t6h\t6h\t6h"                                                                                                                                                                                                                                            
##  [9] "treatment\tna\tna\tna\tDiclofenac\tYM90709\tTubocurarine\tAmidate\tTOCRIS-0930\tTozasertib\tChlorthalidone\tTrazodone\tClotrimazole\tPiperidolate\tOxfendazole\tErythromycin Estolate\tCilostazol\tVenlafaxine\tRS-39604\tPK04_181029\tDexamethasone Acetate\tChenodiol\tAC1M5YQH\tStaurosporine Aglycon"                                    
## [10] "5993::RFX5::regulatory factor X5\t5993\tRFX5\tregulatory factor X5\t-10\t-4.47623\t-9.54631\t1.40607\t1.01019\t-4.40532\t1.54067\t-4.43403\t1.94129\t1.75126\t-2.14805\t-4.2685\t-8.68012\t2.87324\t2.45352\t-7.18714\t1.20953\t-5.25076\t-3.35107\t-3.80186"
```

## Find connected signatures based on user submitted full signature

##### Creating a file to upload from previouslsy downloaded signature file

```
write.table(signatureData,file="sigFile.tsv",sep="\t",row.names=F,col.names = T,quote=F)
system(paste("head sigFile.tsv"))
```

### Upload the file

```
apiUrl<-"http://www.ilincs.org/api/SignatureMeta/upload"
sigFile <- "sigFile.tsv"
req <- POST(apiUrl, body=list(file=upload_file("sigFile.tsv")))
signatureFile <- httr::content(req)$status$fileName[[1]]
signatureFile
```

```
## [1] "processedSig_Thu_Apr_14_00_29_35_2022_978050.xls"
```

### Find connected signatures

```
apiUrl <- "http://www.ilincs.org/api/ilincsR/findConcordances"
req <- (POST(apiUrl, body = list(file=signatureFile, lib="LIB_5"), encode = "form"))
output <- data.table::rbindlist(httr::content(req)$concordanceTable, use.names = TRUE, fill = TRUE)
head(output)
```

## Find connected compound perturbations

```
apiUrl <- paste("http://www.ilincs.org/api/ilincsR/signatureEnrichment?sigFile=",signatureFile,"&library=LIB_5&metadata=TRUE",sep="")
req <- GET(apiUrl)
json <- httr::content(req, as = "text")
iLincsConnectedCompoundPerturbations <- fromJSON(json)$enrichment
head(iLincsConnectedCompoundPerturbations)
```

## Get connected genetic perturbations

```
apiUrl <- paste("http://www.ilincs.org/api/ilincsR/signatureEnrichment?sigFile=",signatureFile,"&library=LIB_6",sep="")
req <- GET(apiUrl)
json <- httr::content(req, as = "text")
iLincsConnectedGeneticPerturbations <- fromJSON(json)$enrichment
head(iLincsConnectedGeneticPerturbations)
```

## Retriving Signature Metadata

```
apiUrl <- paste("http://www.ilincs.org/api/SignatureMeta/",ilincs_signatureId,sep="")
req <- GET(apiUrl)
ilincsJSON<-httr::content(req,type="text")
```

```
## No encoding supplied: defaulting to UTF-8.
```

```
prettify(ilincsJSON)
```

```
## {
##     "antibodytarget": null,
##     "cellline": "A375",
##     "tissue": "skin",
##     "cid": null,
##     "compound": "Diclofenac",
##     "concentration": "10uM",
##     "concordancetable": "concordanceLib5",
##     "datasetid": null,
##     "factor": null,
##     "level1": null,
##     "level2": null,
##     "libraryid": "LIB_5",
##     "lincspertid": "LSM-2160",
##     "nCtrSamples": null,
##     "nTrtSamples": null,
##     "peaktype": null,
##     "platform": "L1000",
##     "signatureid": "LINCSCP_100",
##     "lincsSigID": "CPC004_A375_6H:BRD-K08252256-236-17-1:10",
##     "organism": null,
##     "clueIoCompound": "diclofenac",
##     "integratedMoas": "Cyclooxygenase inhibitor",
##     "GeneTargets": "PTGS1|PTGS2",
##     "time": "6h",
##     "treatment": "Diclofenac",
##     "perturbagenID": "BRD-K08252256",
##     "stitchID": "CID3033",
##     "pubChemID": "3033",
##     "is_exemplar": 1,
##     "pert_type": "trt_cp"
## }
##
```

```
ilincsSigMetaData<-fromJSON(ilincsJSON)
head(ilincsSigMetaData)
```

```
## $antibodytarget
## NULL
## 
## $cellline
## [1] "A375"
## 
## $tissue
## [1] "skin"
## 
## $cid
## NULL
## 
## $compound
## [1] "Diclofenac"
## 
## $concentration
## [1] "10uM"
```

### List of LINCS Datasets

```
apiUrl <- "http://www.ilincs.org/api/PublicDatasets/getDatasets?lincs=true"
req <- GET(apiUrl)
json <- httr::content(req, as = "text")
lincs_datasets <- fromJSON(json)
lincsDatasets <- data.frame(lincs_datasets$data)
lincsDatasets[c("experiment","assay","dataType")]
```

### Dataset metadata

**Example:** EDS-1014

```
experiment <- "EDS-1014"

apiUrl <- paste("http://www.ilincs.org/api/PublicDatasets/",experiment,sep="")
req <- GET(apiUrl)
json <- httr::content(req, as = "text")
datasetMetaData <- fromJSON(json)
datasetMetaData
```

```
## $platform
## [1] "GPL10999syn2347004"
## 
## $description
## [1] "54 mRNA-seq samples from baseline, unperturbed breast cancer cell lines were profiled using Illumina Genome Analyzer IIx to identify patterns of gene expression associated with subtype and response to therapeutic compounds. Data were generated by Microenvironment Perturbagen (MEP) LINCS Center at Oregon Health and Science University."
## 
## $summary
## NULL
## 
## $geolink
## [1] "https://www.synapse.org/#!Synapse:syn2347004"
## 
## $lincsDsgc
## [1] "ohsu"
## 
## $publink
## [1] "http://www.ncbi.nlm.nih.gov/pubmed/24176112"
## 
## $pubmeddescription
## [1] "Daemen A, Griffith OL, Heiser LM, Wang NJ et al. Modeling precision treatment of breast cancer. Genome Biol 2013;14(10):R110."
## 
## $experiment
## [1] "EDS-1014"
## 
## $assay
## [1] "RNA-seq"
## 
## $dataFormat
## [1] "MaxD"
## 
## $sampleType
## [1] "cell line"
## 
## $dataType
## [1] "Gene Expression"
## 
## $organism
## [1] "human"
## 
## $portal
## [1] "LINCS"
## 
## $SourceID
## [1] "EDS-1014"
## 
## $nsamples
## [1] 54
```

Heatmap for dataset EDS-1014 on iLINCS

PCA and tSNE for dataset EDS-1014 on iLINCS

### Dataset sample metadata

```
apiUrl <- paste("http://www.ilincs.org/api/ilincsR/getSamples?id=",experiment,sep="")
req <- GET(apiUrl)
json <- httr::content(req, as = "text")
sampleMeta <- fromJSON(json)
sampleMetaData <- data.frame(sampleMeta$data$rows)
head(sampleMetaData)
```

## Creating signature using iLINCS

Create signature from dataset EDS-1014 on iLINCS

## Creating signature using iLINCS API

```
property <- "ER"
level1 <- "ER:+"
level2 <- "ER:-"
apiUrl <- "http://www.ilincs.org/api/ilincsR/LincsDataAnalysis"
req <- POST(apiUrl, body = list(exp = paste(experiment),prop = property,filterchk=paste(level1,level2,sep = ",,,"),includeORexclude=1), encode = "json")
createdSignaturesSessionID <- httr::content(req)$sessionID
createdSignaturesSessionID
```

```
## [1] "Thu_Apr_14_00_30_39_2022_2085786"
```

## Top 100 genes in created signature

```
l <- lapply(httr::content(req)$geneData, function(x) unlist(x))
ilincs_result <- data.frame(t(sapply(l,c)))
top100signatureData <- ilincs_result[1:100,c("Name_GeneSymbol","Value_LogDiffExp","Significance_pvalue")]
head(top100signatureData)
```

### Heatmap of data used in signature creation

### Signature group heatmap on iLINCS

```
heatmap_url2<-paste("http://www.ilincs.org/apps/heatmap/?sessionID=",createdSignaturesSessionID,"&property=",property,"&geneCount=100",sep = "")
# browseURL(heatmap_url2)  ##uncomment to launch in a browser
# include_url(heatmap_url2, height = "900px")  ##uncomment to embed in an iframe
```
